# Ligand non-innocence and an unusual σ-bond metathesis step enables catalytic borylation using 9-borabicyclo-[3.3.1]-nonane†

**DOI:** 10.1039/d5sc02085a

**Published:** 2025-04-15

**Authors:** Milan Kumar Bisai, Justyna Łosiewicz, Gary S. Nichol, Andrew P. Dominey, Stephen P. Thomas, Stuart A. Macgregor, Michael J. Ingleson

**Affiliations:** a EaStCHEM School of Chemistry, University of Edinburgh Edinburgh EH9 3FJ UK mingleso@ed.ac.uk; b GSK Medicines Research Centre Gunnels Wood Road, Stevenage Hertfordshire SG1 2NY UK; c EaStCHEM School of Chemistry, University of St Andrews KY16 9ST UK

## Abstract

The metal-catalyzed intermolecular C–H borylation of arenes is an extremely powerful C–H functionalization methodology. However, to date it is effectively restricted to forming organo-boronate esters (Aryl–B(OR)_2_) with its application to form other organoboranes rarely explored. Herein, we report a catalytic intermolecular heteroarene C–H borylation method using the commercial hydroborane 9-borabicyclo-[3.3.1]-nonane, (H–BBN)_2_. This process is effective for mono- and di-borylation to form a range of heteroaryl–BBN compounds using either NacNacAl or NacNacZn (NacNac = {(2,6-^i^Pr_2_C_6_H_3_)N(CH_3_)C}_2_CH) based catalysts. Notably, mechanistic studies indicated a highly unusual σ-bond metathesis process between NacNacZn–Aryl and the dimeric hydroborane, with first order kinetics in the hydroborane dimer ((H–BBN)_2_). Our calculated metathesis pathway involves ligand non-innocence and addition of both H–BBN units in (H–BBN)_2_ to the NacNacZn–heteroaryl complex. This is in contrast to the conventional σ-bond metathesis mechanism using other hydroboranes which invariably proceeds by reaction of one equivalent of a monomeric hydroborane (*e.g.*, H–B(OR)_2_) with a M–C unit. Overall, this work demonstrates the potential of extending catalytic arene C–H borylation beyond boronate esters, while highlighting that the σ-bond metathesis reaction can be mechanistically more complex when utilizing dimeric hydroboranes such as (H–BBN)_2_.

## Abstract 2

Herein, we report a Zn/Al catalysed heteroarene C–H borylation using 9-borabicyclo-[3.3.1]-nonane. DFT studies indicated an unusual σ-bond metathesis process between NacNacZn–Aryl and the dimeric hydroborane, involving ligand non-innocence.

## Graphical abstract

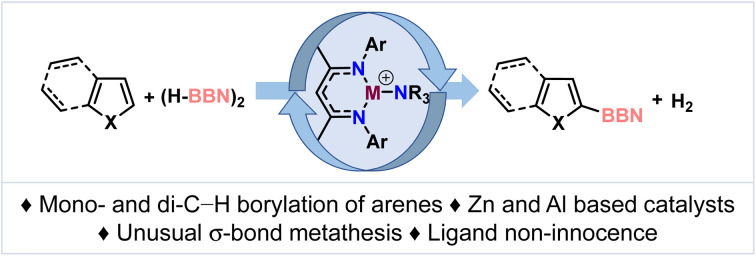

## Introduction

**Fig. 1:**
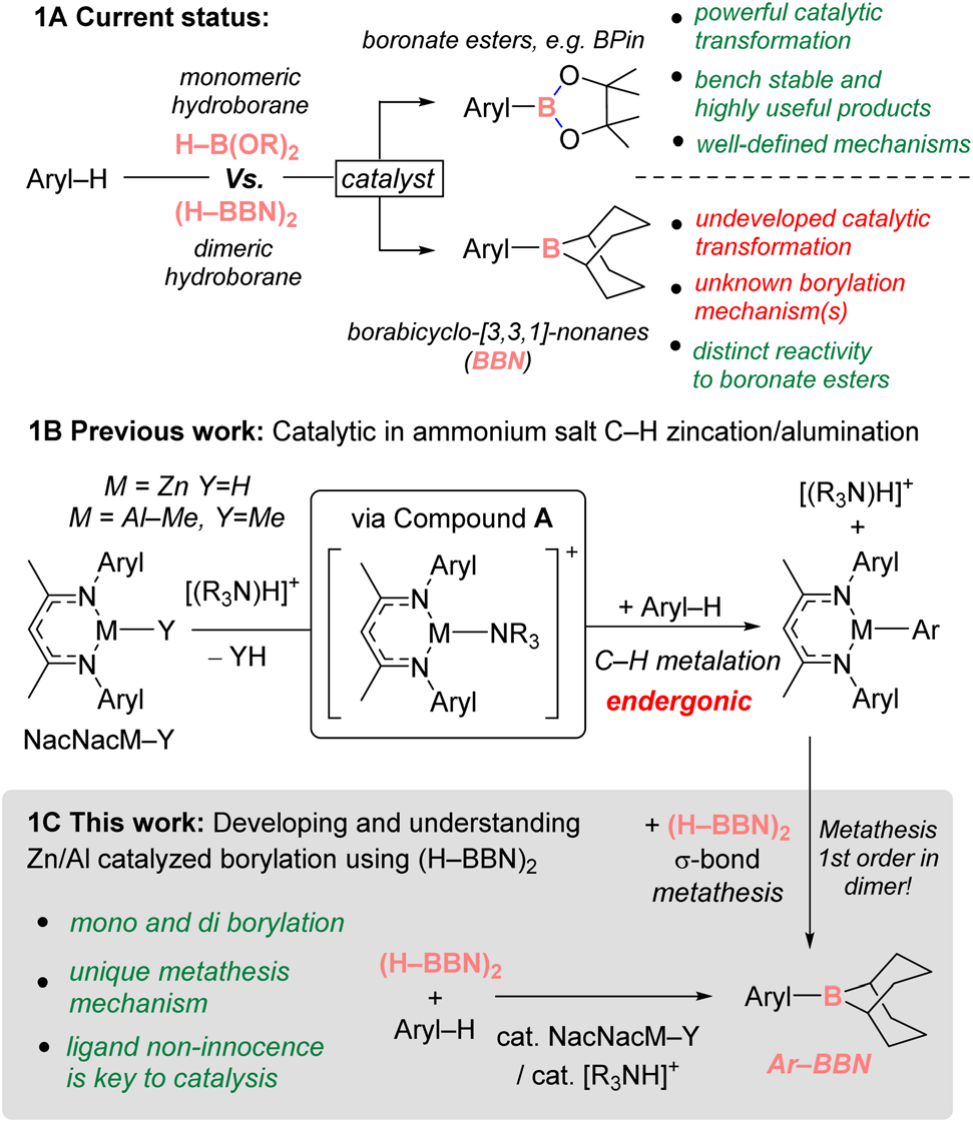
(A): Comparison of the current status of catalytic C–H borylation using monomeric H–B(OR)_2_ and dimeric (H–BBN)_2_. (B): Previous catalyzed C–H zincation and alumination. (C): This work.

The catalytic borylation of arenes is well established as an extremely useful C–H functionalization methodology.^1–3^ However, catalytic intermolecular C–H borylation processes are almost exclusively limited to forming heteroaryl- and aryl-boronate esters (collectively termed herein Ar–B(OR)_2_), particularly pinacol derivatives (Ar–BPin, Fig. 1A).^1–3^ This is due to the commercially available and bench-stable precursors HBPin/B_2_Pin_2_ reacting appropriately with transition metal complexes to enable catalysis to form Ar–BPin products that have considerable synthetic utility. In contrast, catalytic intermolecular borylation to access other aryl boranes (*i.e.*, non Ar–B(OR)_2_) is extremely rare despite the fact that many of these boranes display distinct reactivity profiles compared to the organo-boronate ester analogues. In this area a notable exception are the reports using H–BDan (1,8-naphthalenediaminatoborane) in catalytic C–H borylation.^4–6^ However, the Ar–BDan products and Aryl–BPin compounds are both weakly Lewis acidic at boron. In contrast, *B*-Ar-9-borabicyclo-[3.3.1]-nonane compounds (Ar–BBN) are more Lewis acidic at boron. While this results in lower bench stability, it enables these organoboranes to effect transformations that are not possible using Ar–B(OR)_2_/Ar–BDan.^7–9^ Furthermore, the hydroborane, 9-borabicyclo-[3.3.1]-nonane, (H–BBN)_2_, is commercially available, inexpensive, and widely used in synthesis.^10^ However, to date the use of (H–BBN)_2_ in catalytic intermolecular C–H borylation to form a range of Ar–BBN compounds is not reported to our knowledge (Fig. 1A),^11–13^

Previously, we and others have reported zinc-catalyzed C–H borylations using the monomeric boranes H–BPin, H–BCat, (Cat = *o*-C_6_H_4_O_2_^2−^) and H–BDan.^5,14–20^ The coordination of these monomeric boranes through O/N to zinc electrophiles was proposed to be key for borylation.^14,15^ This is consistent with catalytic borylation not proceeding in these systems when using (H–BBN)_2_ as this borane does not contain a Lewis basic O or N unit.^15^ Furthermore, (H–BBN)_2_ is dimeric and thus often reacts differently to the monomeric dioxaborolanes. More recently, we have used NacNacM–Y (M = Zn, Y=H or M = Al–Me, Y = Me) and sub-stoichiometric ammonium salts to form compound A that effects the C–H zincation and C–H alumination of heteroarenes (Fig. 1B, NacNac = {(2,6-^i^Pr_2_C_6_H_3_)N(CH_3_)C}_2_CH).^21^ Given that NacNacM–R complexes have been reported that undergo σ-bond metathesis with monomeric hydroboranes (*e.g.* H–BPin) to form organoboranes and NacNacM–H,^17,19,22–24^ we wondered if performing arene C–H zincation/alumination in the presence of (H–BBN)_2_ could enable catalytic C–H borylation to form Ar–BBN compounds. This would require the product from C–H metalation, NacNacM–Ar, to undergo σ-bond metathesis with (H–BBN)_2_ dimer to produce Aryl–BBN and NacNacM–H. NacNacM–H would then react rapidly with [(R_3_N)H]^+^ salts to reform compound A.^21^ However, the feasibility of this catalytic borylation process is contingent upon (i) the dimeric (H–BBN)_2_ undergoing σ-bond metathesis with NacNacM–Aryl *via* a low barrier process, and (ii) the Lewis acidic Y–BBN species (Y=H or Ar) not quenching any on-cycle species. A low barrier metathesis step is essential given the endergonic nature of the first step in the putative catalytic cycle, C–H metalation using compound A. This results in an increase in the effective transition state energies for all steps after the C–H metalation. Notably, there are no previously reported studies into the mechanism and associated barriers of the σ-bond metathesis between dimeric hydroboranes, such as (H–BBN)_2_, and (main group element)–Y species (Y=R, OR, NR_2_).^22,25–29^ Note, the dissociation of (H–BBN)_2_ into two equivalents of monomer is significantly endergonic,^26*b*^ therefore it will react in a distinct way to the monomeric boranes widely used to date in catalytic borylation, *e.g.* HBPin/HBCat/HBDan, which could significantly impact the mechanism of the σ-bond metathesis, and other, step(s) in the catalytic cycle.

Herein, we report the Zn/Al-catalyzed C–H borylation of a range of heteroarenes using (H–BBN)_2_. Notably, mechanistic studies indicate a highly unusual σ-bond metathesis process proceeding by the addition of both H–BBN units in dimeric (H–BBN)_2_ to the metal complex which is enabled by NacNac ligand non-innocence.

## Results and discussion

### C–H borylation studies

In our ammonium salt catalyzed aryl C–H zincation,^21^ the highest yields were observed using [(Et_3_N)H]^+^ containing salts and after that [(DMT)H]^+^ (DMT = *N*,*N*-dimethyl-*p*-toluidine). Therefore, these salts were explored in combination with NacNacZnH, 1, for the C–H borylation of 2-methyl-thiophene using (H–BBN)_2_ (Table 1). From this it was found that [(DMT)H][B(C_6_F_5_)_4_] significantly outperformed the [(Et_3_N)H]^+^ congener (entries 1–2), suggesting that a stronger Brønsted acid is vital (pK_a_ [(DMT)H]^+^ = 10.8, [(Et_3_N)H]^+^ = 18.5).^30^ This is distinct to performance in C–H zincation. An anion effect also was observed, with [B(C_6_F_5_)_4_]^−^ being more effective than the more coordinating anion [OTf]^−^ (entry 3). In the absence of 1 or the ammonium salt no C–H borylation was observed (entries 4–5). Ultimately, 5 mol% of 1 and [(DMT)H][B(C_6_F_5_)_4_] was identified as optimal (entry 7, for full optimization see Table S1†).

**Table 1:** Select optimization reactions for 2-methyl-thiophene borylation

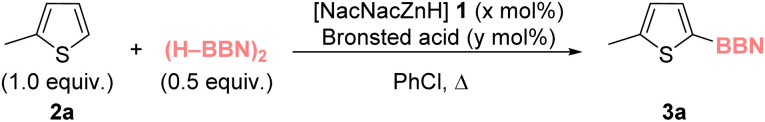					
	Brønsted acid	*x*/*y* mol%	*T* (°C)	*t* (h)	Yield^a^ (%)
1	[(Et_3_N)H][B(C_6_F_5_)_4_]	10/10	60	18	<5
2	[(DMT)H][B(C_6_F_5_)_4_]	10/10	60	18	55
3	[(DMT)H][OTf]	10/10	60	18	< 5
4	[(DMT)H][B(C_6_F_5_)_4_]	0/10	60	18	0
5	—	10/0	60	18	0
6^b^	[(DMT)H][B(C_6_F_5_)_4_]	10/10	80	24	98
7^b^	**[(DMT)H][B(C**_**6**_ **F**_**5**_ **)**_**4**_ **]**	**5/5**	**80**	**24**	**94**
8^b^	[(DMT)H][B(C_6_F_5_)_4_]	2.5/2.5	80	24	84

a Yield relative to (H–BBN)_2_ using an internal standard.

b Using 1.15 equiv. 2a.

**Chart 1:**
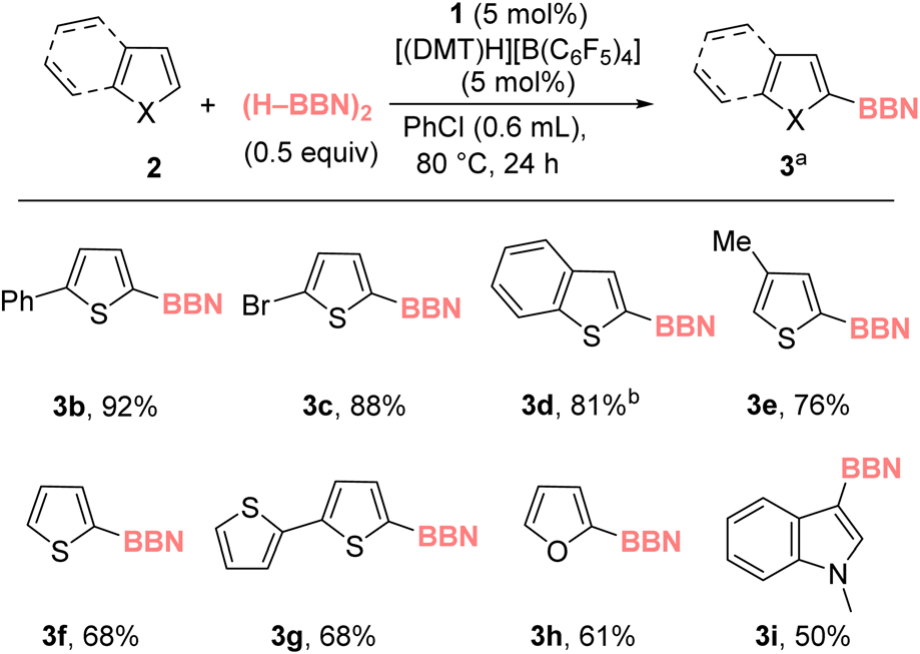
Substrate scope for zinc-catalyzed borylation. ^*a*^ Yield relative to (H–BBN)_2_ using an internal standard. ^*b*^ at 100 °C.

With optimized conditions in hand the scope and limitations of the C–H borylation were explored (Chart 1). Other C2 substituted thiophenes were amenable and produced 3b and 3c in good yield, while the less nucleophilic heteroarene benzothiophene was converted to 3d in 81% yield. 3-Me-thiophene was functionalized predominantly at the C5 position to form 3e (with only a minor amount of C2-functionalized product formed), while thiophene and bithiophene also were borylated to form 3f and 3g, respectively, in good yield. Other heteroarenes, such as furan and N–Me-indole, also were amenable to C–H borylation to form 3h and 3i, respectively, in moderate yield. Note, the products from these reactions are all consistent with S_E_Ar type selectivity, while less activated heteroaromatics (*e.g.*, benzofuran) and activated arenes such as N–Me-carbazole, anisole and anthracene did not undergo borylation, analogous to the outcome from the C–H zincation (see ESI, Section 3.4†). Furthermore, attempts using a chiral dimeric R_2_BH borane, diisopinocampheylborane, under optimized conditions resulted in no C–H borylation of 2-methylthiophene (after 24 h at 80 °C).

**Chart 2:**
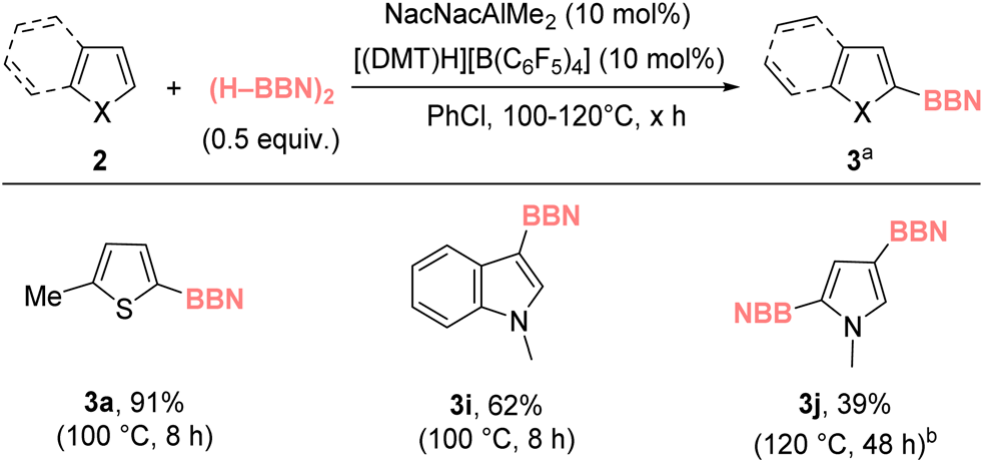
Aluminium catalyzed C–H borylation. ^*a*^ Yield *versus* an internal standard. ^*b*^ 1.5 equiv. (H–BBN)_2_.

Next, we assessed if aluminium-catalyzed borylation was possible. It was found that 1 could be replaced with NacNacAlMe_2_4 in the catalytic borylation using (H–BBN)_2_ (Chart 2). Aluminium-catalyzed borylation also was applied to N–Me-pyrrole to form diborylated 3j (attempts to form mono-borylated N–Me-pyrrole resulted in mono and diborylated products).

**Fig. 2:**
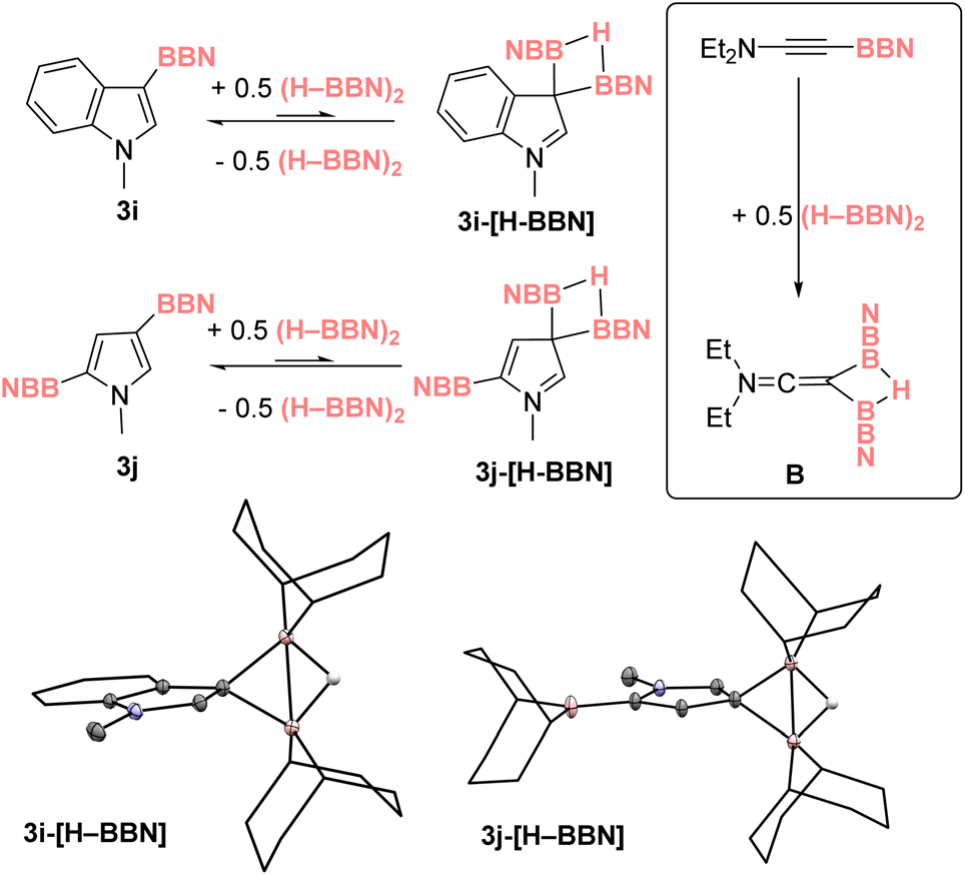
Reaction of 3i/3j with (H–BBN)_2_. Bottom, solid-state structures of 3i/3j-(H–BBN), ellipsoids at 50% probability and most hydrogens omitted for clarity. Inset right the formation of compound B also containing a CB_2_H core.

From the reactions producing 3i and 3j a small quantity of crystals suitable for X-ray diffraction analysis were formed. Notably, this analysis showed these were not 3i/3j but instead were the H–BBN adducts of 3i and 3j, termed 3i-(H–BBN) and 3j-(H–BBN) (Fig. 2). *In situ* analysis of the Zn/Al catalyzed reactions that form 3i/3j revealed that while the major resonance is due to 3i/3j (*δ*^11^_B_ ≈ 72), an additional minor ^11^B resonance at *ca.* 5 ppm was present consistent with 3i/3j-(H–BBN).^31^ Indeed, dissolution of crystals of 3i-(H–BBN) or 3j-(H–BBN) led to formation of 3i/3j and (H–BBN)_2_ as the major product (by NMR spectroscopy), indicating a solution equilibrium favoring 3i/3j and (H–BBN)_2_. Note, no resonances for thienyl analogues of 3i-/3j-(H–BBN) were observed in any of the reactions, presumably due to the lower nucleophilicity of thiophenes relative to indoles/pyrroles^32^ and consistent with the computed trend observed in our DFT calculations (see Table S5†). The formation of 3i/3j-(H–BBN) is related to the reaction of Et_2_N–C≡C–BBN with (H–BBN)_2_ which forms a compound also containing a cyclic CB_2_H unit (Fig. 2 inset, compound B).^31^

**Chart 3:**
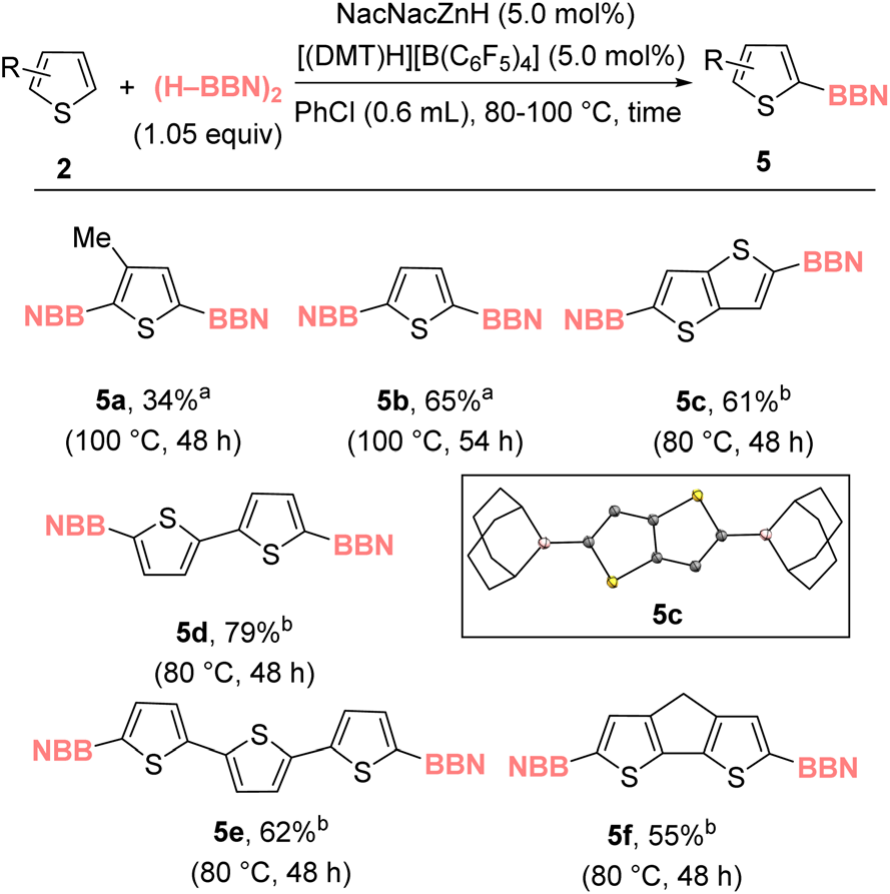
Zinc catalyzed diborylation of thiophenes. ^*a*^ Yield *versus* an internal standard. ^*b*^ Isolated yield.

With the mono-borylation scope assessed, our attention turned to the diborylation of thiophenes given the importance of diborylated precursors in accessing organic materials.^33^ Note, to date the catalytic C–H diborylation of thiophenes *via* an S_E_Ar type process (*i.e.* transition metal free) is limited to only the most highly nucleophilic thiophenes, such as 3,4-dialkoxy-substituted thiophenes.^34^ Diborylated thienyl products 5a–5f proved accessible through zinc catalysis simply by increasing the equivalents of (H–BBN)_2_ used (Chart 3). Notably, most of the diborylated products were poorly soluble in the reaction solvent, chlorobenzene, facilitating their facile isolation in good yield. X-ray diffraction studies on a number confirmed their formation (inset Chart 3 for 5c, for 5d and 5e see ESI†).

### Synthetic utility

**Scheme 1:**
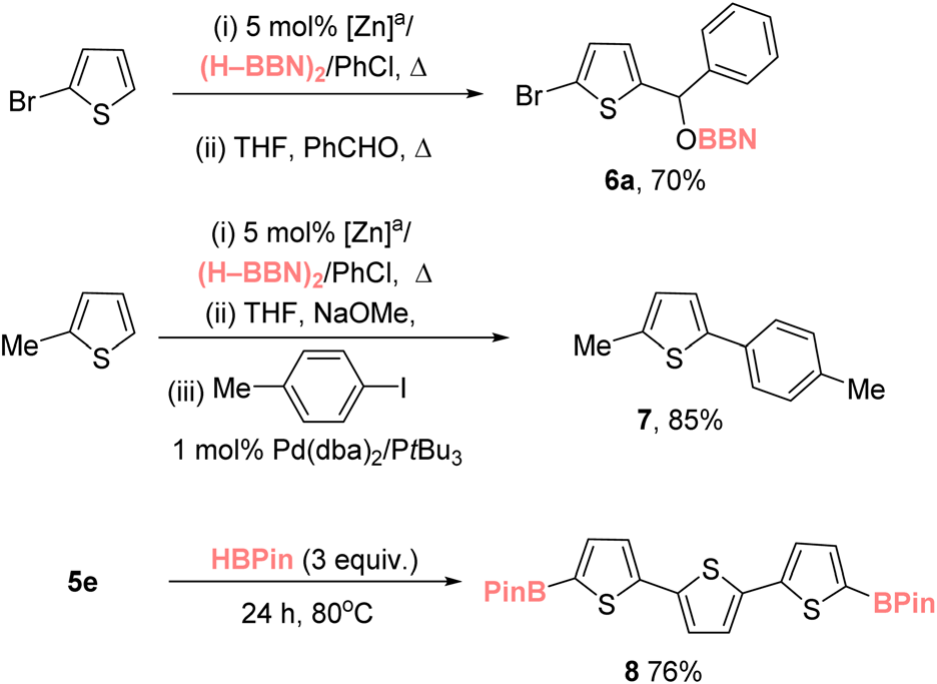
Utilization of thienyl–BBN products. ^*a*^ Borylation conditions from Table 1.

With a range of mono- and di-borylated products accessible their utility was explored. Given the importance of thienyl–C(H)(OH)Ph units in active pharmaceutical ingredients, *e.g.*, tiemonium salts,^35^ a one-pot route to these motifs from a thiophene precursor was targeted. The reaction of crude (*i.e.*, made *in situ* and taken forward with no purification) Br–thienylBBN (3c) with benzaldehyde proceeded to form 6a in 70% yield (Scheme 1 top) with the alcohol 6b then formed using the previously reported O–BBN hydrolysis conditions (see Fig. S76†).^8^ Importantly, thienyl–BPin compounds do not react with benzaldehyde under identical conditions, presumably due to their lower Lewis acidity at boron. This demonstrates that this catalytic C–H borylation approach can be harnessed in tandem with the distinct reactivity profile of Ar–BBN derivatives to access value-added products in one-pot from the starting heteroarene. The crude products from zinc-catalyzed C–H borylation also can be telescoped into a Suzuki–Miyaura cross-coupling reaction (Scheme 1, middle), which led to compound 7 in excellent yield for a multi-step process. Bench stable borylated derivatives also were prepared by *trans*-borylation of thienyl–BBN species with HBPin to generate the Aryl–Bpin congeners (*e.g.*, to form 8).^36^

### Mechanistic studies

**Scheme 2:**
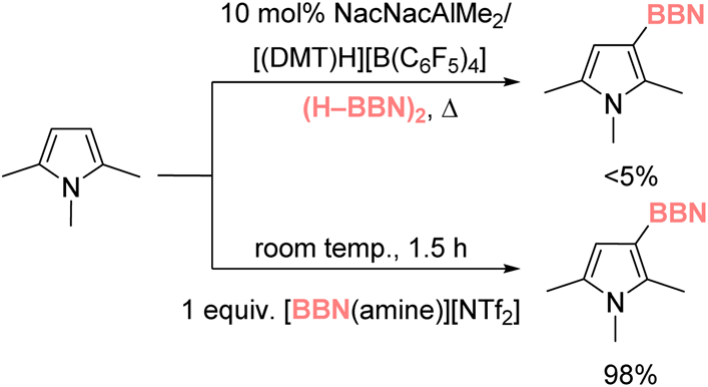
Disparate outcomes from the borylation of 2,5-*N*-trimethylpyrrole.

With the scope and utility of the process assessed our attention turned to the mechanism. For this catalytic borylation a NacNacM–Y complex (*e.g.*1), and [(DMT)H][B(C_6_F_5_)_4_] were both required. This is consistent with the observation that (H–BBN)_2_ and [(DMT)H][B(C_6_F_5_)_4_] do not react (even on heating to 80 °C) to form a [(DMT)–BBN]^+^ salt (with related salts known to be effective borylating agents,^37^ including in catalytic processes when using [CatB(amine)]^+^ derivatives).^38,39^ To further preclude the possibility of [(DMT)–BBN]^+^ mediated borylation occurring in this process, the borylation of 2,5-*N*-trimethylpyrrole was attempted under the optimized aluminium-catalyzed conditions. This substrate was chosen as it undergoes C–H borylation using stoichiometric [(amine)–BBN]^+^ in high yield at room temperature,^37^ while it has a sterically hindered borylation position located alpha to a methyl (C–H alumination is highly sensitive to steric environment).^21^ Under our optimized aluminium-catalyzed conditions < 5% C–H borylation of 2,5-*N*-trimethylpyrrole was observed at 100 °C, further disfavoring a [(DMT)–BBN]^+^ mediated catalytic borylation process (Scheme 2). Furthermore, DFT calculations (*vide infra* and Fig. S115†) disfavored a process proceeding through the borenium equivalent [NacNacZn–(μ-H)–BBN]^+^. Therefore, our mechanistic studies focused on the hypothesis that heteroarene C–H metalation followed by σ-bond metathesis leads to Aryl–BBN.

Given the greater substrate scope of the zinc catalyzed metalation/borylation^21^ all subsequent work focused on this system. The zinc-catalyzed reactions use NacNacZnH (1) and [(DMT)H][B(C_6_F_5_)_4_]; and these react to form [NacNacZn(DMT)][B(C_6_F_5_)_4_] ([9][B(C_6_F_5_)]_4_) rapidly through a low-energy transition state.^21^ It was proposed that C–H borylation then would proceed by: (i) [9]^+^ reacting with a heteroarene to form NacNacZn–Aryl and [(DMT)H]^+^; (ii) the Zn–Aryl species would then undergo σ-bond metathesis with (H–BBN)_2_ to form the Ar–BBN product, and regenerate the zinc-hydride 1; (iii) 1 would then react with [(DMT)H]^+^ to form [9]^+^ and H_2_. At this point it is important to highlight that 2-methyl-thiophene C–H zincation using [9]^+^ is endergonic by +15 kcal mol^−1^.^21^ Therefore, to have a feasible Δ*G*^‡^_span_ for this catalytic C–H borylation cycle the σ-bond metathesis step involving dimeric (H–BBN)_2_ has to proceed *via* a low barrier process.

**Fig. 3:**
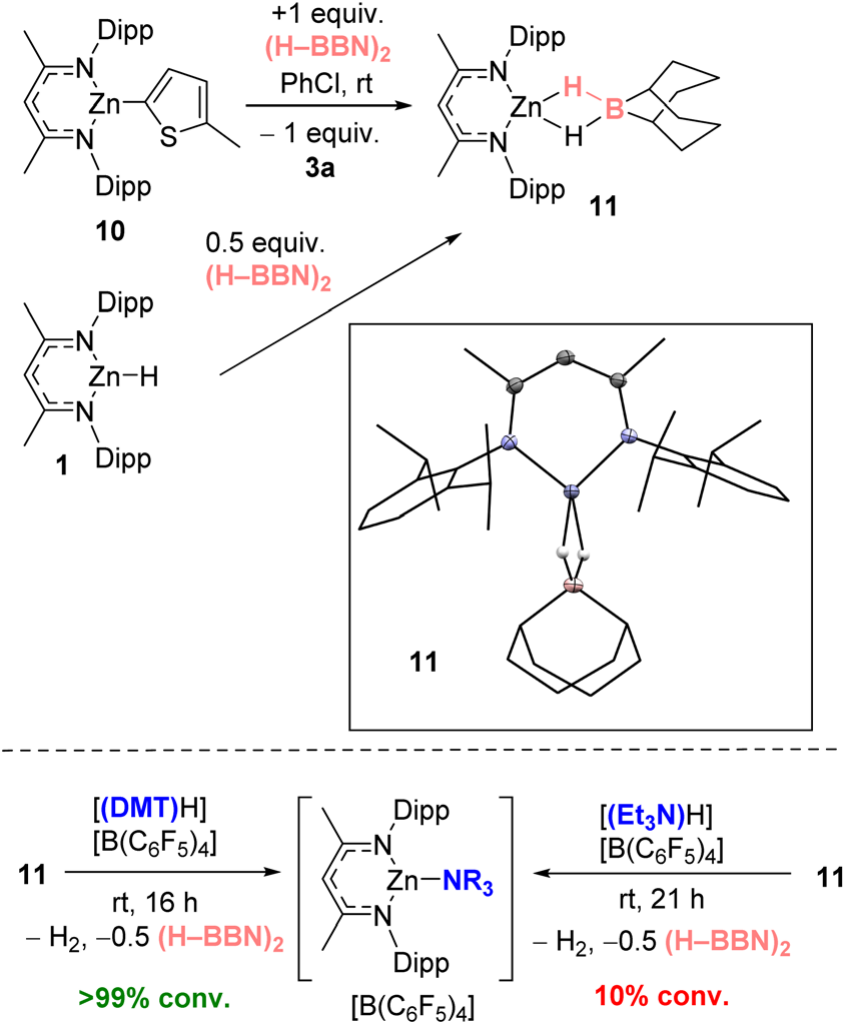
Top, the formation of 11 by metathesis of 10 with (H–BBN)_2_ (also forming 3a) or by direct addition of 0.5 equiv. (H–BBN)_2_ to 1. Inset, the solid-state structure of 11. Bottom, the disparate outcome from protonolysis of 11.

To probe the metathesis process NacNacZn(thienyl) complex 10 was synthesized and reacted with 0.5 equivalent of (H–BBN)_2_. While the metathesis did proceed at room temperature to form thienyl–BBN, 3a, only 50% of 10 was consumed to form a single new NacNacZn product that was not compound 1. Instead, the zinc complex displayed NMR data consistent with formation of NacNacZn–(μ-H)_2_–BBN, 11 (Fig. 3). This included a ^11^B NMR resonance (at −13.5 ppm) comparable with other M–(μ-H)_2_–BBN complexes.^25,26a,40^ The use of 1 equiv. of (H–BBN)_2_ resulted in the full conversion of 10 into 3a and 11. The formation of 11 was confirmed by single crystal X-ray diffraction studies (inset Fig. 3). The structure of 11 contains a Zn⋯B distance of 2.179 Å consistent with a borohydride unit bound to zinc *via* two bridging hydrogens, with 11 having comparable metrics to NacNacZn–(μ-H)_2_–BH_2_.^41^ Complex 11 also can be synthesized from NacNacZnH 1 by addition of 0.5 equivalent of (H–BBN)_2_. Note, while the boron center in 11 is structurally similar to the active boron electrophile in Wang's borocation-mediated catalytic C–H borylation,^42^ complex 11 is neutral and thus is a weak electrophile at boron which is not active in C–H borylation (Table 1, entry 5).

The formation of 11 instead of 1 is significant as it demonstrates a key difference in reactivity using (H–BBN)_2_*versus* H–BPin.^17^ Furthermore, to close the C–H borylation cycle the Brønsted acid, [(DMT)H]^+^, produced during the arene C–H zincation step has to react with a hydridic species to reform 9 (and concomitantly H_2_). A protonolysis step starting from 11 is distinct to the protonolysis of NacNacZnH 1, which is facile with a range of [(R_3_N)H]^+^ salts.^21^ Therefore, the reaction of [(DMT)H][B(C_6_F_5_)_4_] and 11 was investigated. This revealed a rapid reaction at room temperature to form [9][B(C_6_F_5_)_4_], H_2_ and 0.5 equiv. (H–BBN)_2_ (Fig. 3, bottom). In contrast, the reaction between [(Et_3_N)H][B(C_6_F_5_)_4_] and 11 proceeds very slowly at room temperature indicating a higher barrier protonolysis step starting from 11 than starting from 1 (Fig. 3, bottom). This may explain the poor outcomes using [(Et_3_N)H][B(C_6_F_5_)_4_] in the catalytic borylation compared to that using [(DMT)H][B(C_6_F_5_)_4_].

**Fig. 4:**
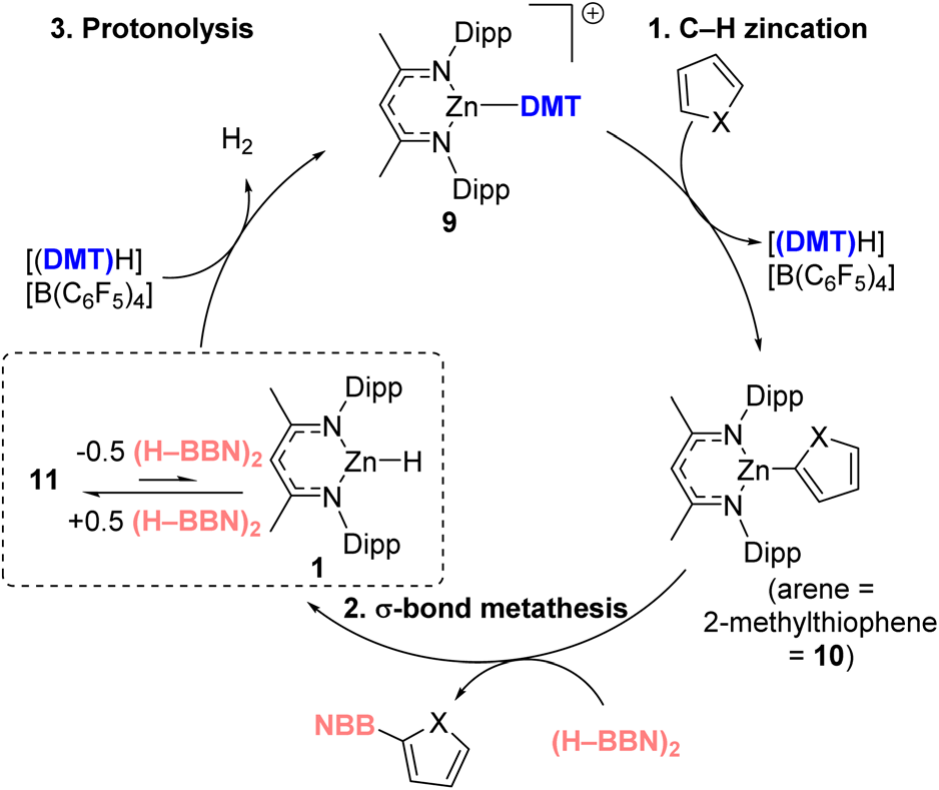
Proposed catalytic cycle for C–H borylation.

Overall, the proposed cycle is outlined in Fig. 4, proceeding *via* 1. Arene C–H zincation, 2. σ-bond metathesis and 3. Protonolysis. The latter could potentially occur directly from 11 or by endergonic conversion of 11 into 1 which would then undergo protonolysis. While the stoichiometric experiments confirmed that both the σ-bond metathesis and protonolysis of 11 using [(DMT)H][B(C_6_F_5_)_4_] proceed at room temperature, how they proceed remained a key question. The σ-bond metathesis process was of particular interest, as the C–H borylation reactions used (H–BBN)_2_ which strongly favors its dimeric form in contrast to the monomeric hydroboranes (*e.g.*, H–BPin) previously studied.

Cognizant that hidden catalysis can facilitate transformations in organoboron chemistry,^43^ several species that could be present at low concentration during the catalysis were tested for their effect on the metathesis step. The addition of 5 mol% of DMT, [(DMT)H][B(C_6_F_5_)_4_] and Me_2_S–BH_3_ separately to the reaction between Zn–Aryl 10 and (H–BBN)_2_ led to no change in the rate of the metathesis reaction, indicating that these species do not catalyze this metathesis reaction. With the positive effect of catalyst loading on borylation rate determined (Table 1 and Fig. S107 and S108†), variable time normalization analysis (VTNA)^44^ was used to determine the order with respect to (H–BBN)_2_. This analysis gave data that was consistent with a first order in (H–BBN)_2_ dimer for the metathesis step (Fig. S100†). Some alkene hydroboration reactions using (H–BBN)_2_ are first order with respect to the dimer,^45^ which indicates the dissociation of the (H–BBN)_2_ dimer into two equivalents of (H–BBN) monomer is rate limiting. In these cases, the hydroboration reactions are zero order with respect to the other component(s). The situation here is different, as the rate is also affected by the concentration of the zinc complex and the thienyl substrate (see Tables 1, S1 and Fig. S107 and S108†). This precludes (H–BBN)_2_ dimer dissociation into two monomers being the turnover limiting step in this zinc catalyzed arene borylation. The standard metathesis mechanism involves a monomeric hydroborane species reacting *via* a four membered transition state (*e.g.*, involving M–C and H–B). However, such a process is not consistent with the above data as when one equivalent of monomeric H–BBN reacts with a substrate in the rate limiting step the reaction is 0.5 order with respect to (H–BBN)_2_ dimer.^45^ Therefore, computational analysis was performed to provide further insight into the mechanism.

### Computational studies

All calculations discussed herein were performed at the B3PW91(def2-TZVP, D3(BJ), PhCl)//B3PW91(Zn: SDD; S: SDD(d); other atoms: 6-31G**) level of theory which was chosen based on its performance in our work on the related catalyzed C–H zincation.^21^ Note, in the solvent used for the borylation reactions, PhCl, H–BBN exists as a dimer, (H–BBN)_2_, and at this level of theory +9.9 kcal mol^−1^ (Δ*G*) per monomer unit is required for dissociation of the dimer. Therefore, calculations involving association/dissociation of H–BBN use 0.5 equiv. of (H–BBN)_2_ for determining the energy change.

**Scheme 3:**
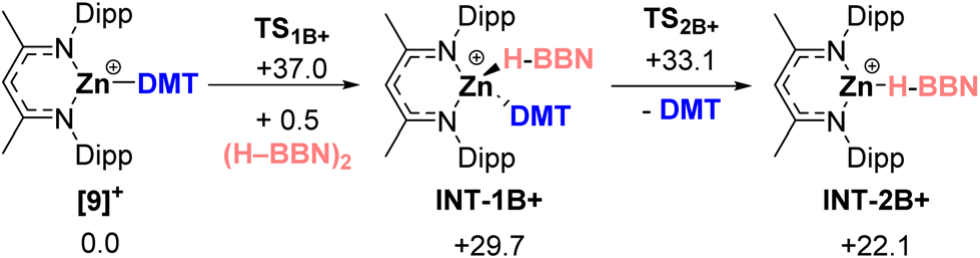
Calculated free energies (kcal mol^−1^) to form the borenium equivalent INT-2B+.

As mentioned above a borylation mechanism involving borenium equivalents *e.g.*, INT-1B+ or INT-2B+ was disfavored based on DFT calculations (Scheme 3). Specifically, the displacement of DMT by H–BBN proceeds *via* formation of INT-1B+ which is formed in a significantly endergonic step involving a high energy transition state (Δ*G*^‡^ = +37.0 kcal mol^−1^) which precludes this mechanism. Notably, the formation of INT-2B+ from [9]^+^ is much more endergonic than the displacement of DMT in [9]^+^ by HBCat (which coordinates through an oxo group to zinc).^14^ This significant difference presumably is why there is no C–H borylation using (H–BBN)_2_ when catalyzed by zinc electrophiles that do not effect C–H metalation.^15^

**Fig. 5:**
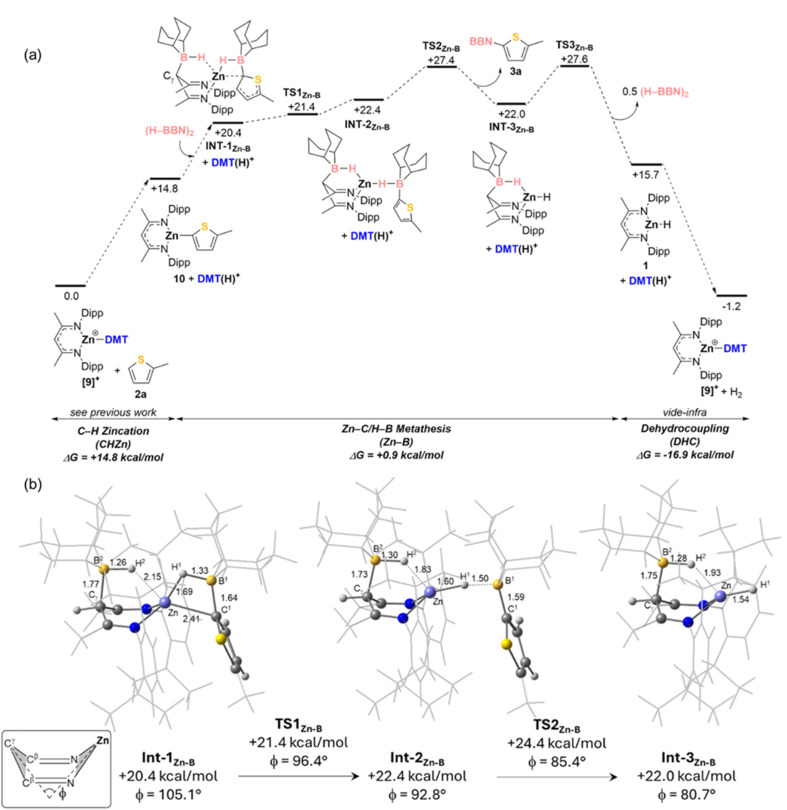
(a) Computed free energy reaction profile (kcal mol^−1^) for the catalytic C–H borylation of 2-methyl-thiophene focusing on the σ-bond metathesis phase. (Method: B3PW91(def2-TZVP, D3(BJ), PhCl)//B3PW91(Zn: SDD; S: SDD (d); other atoms: 6-31G**)). (b) Details of key intermediates in the σ-bond metathesis process (distances in Å; inset defines the metallacycle folding angle, *ϕ*;^21^ NacNac, BBN and 2-methyl-thiophene substituents shown in wireframe for clarity).

A range of mechanisms then were explored for the catalytic borylation of 2-methyl-thiophene that proceed *via* C–H zincation, σ-bond metathesis and protonolysis and a computed profile that is consistent with the VTNA analysis (1st order in (H–BBN)_2_ dimer) is shown in Fig. 5(a). Here [NacNacZn(DMT)]^+^ ([9]^+^) and 2-methyl-thiophene are taken as the starting point of the cycle. Overall, the reaction proceeds as postulated, with an endergonic C–H zincation to form 10 (+14.8 kcal mol^−1^) proceeding as outlined in our previous report (*via* a transition state at +21.7 kcal mol^−1^).^21^ This is followed by a σ-bond metathesis phase that is effectively thermoneutral (+0.9 kcal mol^−1^) with the overall process driven forward by exergonic dehydrocoupling to reform [9]^+^ with loss of H_2_ (Δ*G* = −16.9 kcal mol^−1^). The overall process is therefore exergonic (Δ*G* = −1.2 kcal mol^−1^).

The σ-bond metathesis phase proceeds through addition of the (H–BBN)_2_ dimer to 10 to form INT-1_Zn-B_ at +20.4 kcal mol^−1^. This involves the C_γ_ position of the NacNac ligand acting as a Lewis base towards one H–BBN unit, with the other H–BBN unit interacting with the Zn–thienyl moiety.^46^INT-1_Zn-B_ therefore contains two B–H⋯Zn interactions, with that bridging the Zn–thienyl unit being stronger (Zn⋯H^1^–B^1^ = 1.69 Å; B^1^–C^1^ = 1.64 Å *cf.* Zn⋯H^2^–B^2^ = 2.15 Å; C_γ_–B^2^ = 1.77 Å, see Fig. 5(b)). This also induces significant elongation of the Zn–C^1^ bond (2.41 Å *vs.* 1.93 Å in 10) such that its cleavage has a barrier of only 1 kcal mol^−1^*via*TS1_Zn-B_. This forms INT-2_Zn-B_ in which the Zn–H^1^⋯B^1^ moiety approaches linearity (165°) and from which dissociation of the thienyl–BBN product proceeds *via*TS2_Zn-B_ with concomitant contraction of the B^2^–H^2^⋯Zn distance to 1.83 Å. Thus, the non-innocence of the NacNac ligand^47^ not only assists the cleavage of the (H–BBN)_2_ dimer by C_γ_–B bond formation, but the resultant C_γ_-bound hydroborane provides flexible ligation of the zinc centre during the metathesis phase.

This is also reflected in the variation of the Zn–NacNac metallacycle folding angle, ϕ, as previously discussed (inset Fig. 5(b))^21^ and is also seen in INT-3_Zn-B_, formed after product dissociation (B^2^–H^2^⋯Zn = 1.93 Å; *ϕ* = 80.7°). To complete the σ-bond metathesis phase, dissociation of H–BBN occurs *via*TS3_Zn-B_ at +27.6 kcal mol^−1^.

**Scheme 4:**
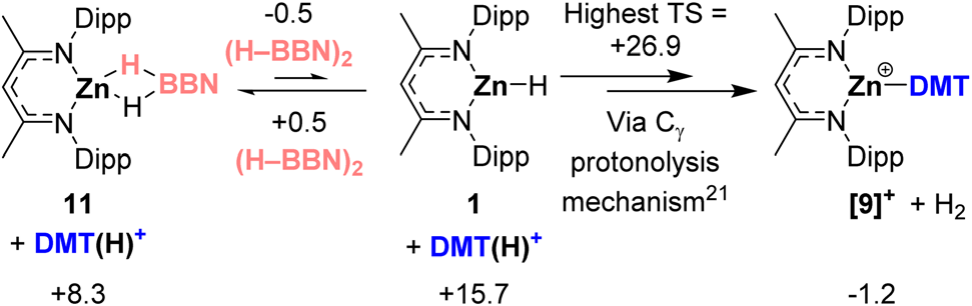
Free energies (kcal mol^−1^) for the protonolysis phase that proceeds *via*1 with only the highest transition state energy shown. Energies are in kcal mol^−1^ relative to the zero energy defined in Fig. 5.

Details of the final phase, protonolysis, are shown in Scheme 4. No pathway involving direct protonolysis of 11 by [(DMT)H]^+^ to form [9]^+^ was found with feasible barriers (see Fig. S111†). However, dissociation of 0.5 equiv. of (H–BBN)_2_ from 11 to form NacNacZnH, 1, is endergonic by only 7.4 kcal mol^−1^. Complex 1 can then react with [(DMT)H]^+^*via* the mechanism described in our previous work which proceeds by protonation of the C_γ_ position of NacNac.^21^ Note, the highest-lying transition state for this process lies at +26.9 kcal mol^−1^ and so is competitive with TS3_Zn-B_ at +27.6 kcal mol^−1^ in the σ-bond metathesis phase. However, we disfavor this protonolysis step being rate limiting as that scenario would result in an inverse dependence on [(H–BBN)]_2_,^26^ not the first order dependence observed. Finally, as with the σ-bond metathesis phase, we highlight that NacNac ligand non-innocence again appears essential for accessing sufficiently low barrier mechanisms for the protonolysis phase.

## Conclusions

A catalytic intermolecular arene C–H borylation process using (H–BBN)_2_ to make a range of mono- and di-borylated heteroarenes is reported for the first time to our knowledge. Due to the endergonic nature of the C–H metalation step, for this C–H borylation process to be energetically feasible it requires low barrier σ-bond metathesis and protonolysis steps. Kinetic studies revealed that the σ-bond metathesis process proceeds *via* addition of both H–BBN units in the (H–BBN)_2_ dimer to the NacNacZn–Aryl complex – *i.e.*, the order of reaction with respect to (H–BBN)_2_ was found to be 1. This is distinct to conventional σ-bond metatheses which involve one equivalent of a monomeric hydroborane reacting with a M–Y unit of a metal complex. The involvement of both H–BBN units in the turnover limiting process is crucial as this offsets the significant energetic cost of cleaving dimeric (H–BBN)_2_. This highlights the importance of considering the correct speciation of the hydroborane when designing borylation processes using (H–BBN)_2_, as this has a considerable impact on the energy profile for a catalytic cycle. In this case, the ability of the metal complex to interact with two H–BBN units is enabled by ligand non-innocence, specifically interaction of the NacNac C_γ_ position with one of the H–BBN units derived from (H–BBN)_2_. The second H–BBN unit then effects the σ-bond metathesis with the M–Aryl unit. Thus, NacNac ligand non-innocence is essential for low barrier σ-bond metathesis and protonolysis phases, which is crucial for enabling borylation catalysis given the endergonic nature of the C–H metalation phase. Thus, this study showcases the benefits of ligating main group metals with non-innocent NacNac ligands, the dramatic impact (H–BBN)_2_ has on the mechanism relative to monomeric boronate esters (*e.g.*, HBPin), and the utility of extending catalytic C–H borylation beyond the synthesis of the aryl-boronate esters that currently dominate this field.

## Author contributions

MKB, JL, SPT, MI and SAM conceived the research concept and aims and analyzed all data. MKB and JL performed the majority of the synthetic work and the majority of the analytical components of this project. GN collected and solved all the crystal structures. JL performed all the calculations. Combined, all authors drafted, reviewed and edited the manuscript.

## Conflicts of interest

There are no conflicts to declare.

## Supplementary Material

SC-016-D5SC02085A-s001

SC-016-D5SC02085A-s002

SC-016-D5SC02085A-s003

## Data Availability

The data supporting this article has been uploaded as part of the ESI.† This includes NMR spectra for all new compounds, *in situ* NMR spectra for catalytic and mechanistic reactions and Cartesian coordinates for all calculated structures.
