# Supplementary material for: Ligand non-innocence and an unusual σ-bond metathesis step enables catalytic borylation using 9-borabicyclo-[3.3.1]-nonane
Source: Chem Sci. 2025 Apr 15;16(21):9255–63. doi: 10.1039/d5sc02085a (PMC12019295; doi:10.1039/d5sc02085a)
Supplement: SC-016-D5SC02085A-s001 [file SC-016-D5SC02085A-s001.pdf]

Supplementary Information (SI) for Chemical Science.
This journal is © The Royal Society of Chemistry 2025

Supporting Information for:

Ligand non-innocence and an unusual σ-bond metathesis step enables catalytic borylation using 9-borabicyclo-[3.3.1]-nonane.

Milan Kumar Bisai[a‡], Justyna Łosiewicz[a‡], Gary S. Nichol[a], Andrew P. Dominey[b], Stephen P. Thomas[a], Stuart A. Macgregor[c]* and Michael J. Ingleson[a]*

‡ = equal contribution

[a]EaStCHEM School of Chemistry, University of Edinburgh, Edinburgh, EH9 3FJ, UK.

[b]GSK Medicines Research Centre, Gunnels Wood Road, Stevenage, Hertfordshire SG1 2NY, UK.

[c]EaStCHEM School of Chemistry, University of St Andrews, St. Andrews, KY16 9ST, UK.

Contents

S1. General considerations………………………………………………………………...S2

S2. Synthesis of [(DMT)H][NTf_2]……………………………………………………......S3-S5

S3. Synthesis of aryl-BBN compounds……………………………………………......S5-S56

S4. Synthetic utility of aryl-BBN compounds…………………………………………S56-S65

S5. Mechanistic studies………………………………………………………...………S66-S88

S6. Crystallographic data………………………………………………………..……......S89-S94

S7. Computational details…………………………...……………………………S95-S163

S8. References…..……………………………………………….……………….…S164-S165

S1. General Considerations

Unless otherwise mentioned, all the experiments were carried out under an inert atmosphere using either standard Schlenk techniques or in a MBraun glovebox (<0.1 ppm H_2O/O_2). Chlorobenzene, C_6D_6 and 1,2-difluorobenzene were distilled over CaH_2 and stored over activated 3 Å molecular sieves. C_6D_5Br and CD_2Cl_2 was over dried over 3 Å molecular sieves. All other solvents were obtained from an Inert PureSolv MD5 SPS and further dried over activated 3 Å molecular sieves. Unless otherwise stated all chemicals were purchased from commercial sources and used as received. ^{Dipp}NacNacH,[1] [where ^{Dipp}NacNac = {2,6-iPr$_2$C$_6$H$_3$NC(Me)}$_2$CH], ^{Dipp}NacNacZnH,[2] ^{Dipp}NacNacAlMe$_2$,[3] [(DMT)H][B(C$_6$F$_5$)$_4$],[4] [(DMT)H][B{C$_6$H$_3$(CF$_3$)$_2$}$_4$],[5] (where, DMT = *N,N*-dimethyl-4-toluidine), [(2,4-Br$_2$C$_6$H$_3$-NMe$_2$H][B(C$_6$F$_5$)$_4$],[5] [(Et$_3$N)H][B(C$_6$F$_5$)$_4$],[5] [(Ph$_3$P)H][B(C$_6$F$_5$)$_4$],[5] [(Et$_3$N)H][OTf],[5] [(DMT)H][OTf],[5] [(Et$_3$N)H][B{C$_6$H$_3$(CF$_3$)$_2$}$_4$],[6] [(DET)H][B(C$_6$F$_5$)$_4$],[5] (where, DET = *N,N*-diethyl-4-toluidine), [^{Dipp}NacNacZn-DMT][B(C$_6$F$_5$)$_4$] (**9**),[5] [^{Dipp}NacNacZn-NEt$_3$][B(C$_6$F$_5$)$_4$],[5] and [^{Dipp}NacNacZn-NEt$_3$][B{C$_6$H$_3$(CF$_3$)$_2$}$_4$][5] were prepared as per reported literature procedures.

^{1}H, ^{13}C{^{1}H}, ^{11}B, and ^{19}F NMR spectra were recorded on Bruker Advance III 500MHz or Bruker PRO 500 MHz spectrometers and referenced to the solvent in use for ^{1}H and ^{13}C{^{1}H}, while ^{11}B and ^{19}F shifts are referenced relative to external $BF_3{\cdot}Et_2O$ and C_6F_6, respectively. Chemical shifts are reported as dimensionless δ values in ppm, coupling constants J are given in Hertz (Hz). The multiplicity of the signals is indicated as "s", "d", "t" "q" "pent", "sept" or "m" for singlet, doublet, triplet, quartet, pentet, septet or multiplet, respectively. Background signals in ^{11}B NMR spectra arise to a significant degree from glass components of the NMR tubes used as well as probes used in our spectrometers. Unless otherwise stated NMR spectroscopy was undertaken at room temperature (~27°C). Carbon atoms directly bonded to B are not observed in ^{13}C{^{1}H} NMR spectra due to quadrupolar broadening.

Mass spectrometry was performed by the Scottish Instrumentation and Resource Centre for Advanced Mass Spectrometry (SIRCAMS) at the University of Edinburgh. Mass spectrometry for [(DMT)H][NTf$_2$] was performed using impact (EI) and electrospray ionisation (ESI) techniques. Samples of C−H borylated products and zinc compounds were prepared in either toluene or chloroform (~2 mg in 0.5 mL) and ionised from an adapted glovebox using an atmospheric pressure photoionization (APPI) source connected to a 12T FT-ICR Solarix (Bruker) in positive mode. Data analysis was carried out using Data Analysis (Bruker).

S2. Synthesis of [(DMT)H][NTf$_2$]

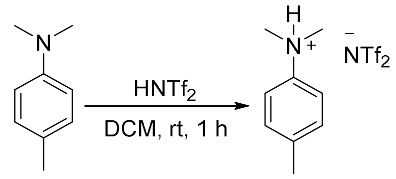

Bis(trifluoromethane)sulfonimide (100 mg, 0.35 mmol, 1.0 equivalent) charged in a J. Young's ampule was dissolved in DCM (5 mL). *N,N*-dimethyl-4-toluidine (52µL, 0.36 mmol, 1.02 equiv.) was slowly added to the reaction mixture at room temperature and stirred for 1 h before all the volatiles were removed *in vacuo* affording a colourless oil. Yield: 92% (137 mg).

^{1}H NMR (500 MHz, CD$_2$Cl$_2$): δ 9.16 (br., 1H, N*H*), 7.39 (br., 4H, ^{DMT}*Ar*), 3.28 (s, 6H, N*Me*$_2$), 2.42 (s, 3H, ^{DMT}*Me*).

^{13}C{^{1}H} NMR (126 MHz, CD$_2$Cl$_2$): δ 141.9, 139.8, 131.7, 120.2 (q, $^1J_{C\text{-}F}$ = 320.5 Hz, *C*F$_3$), 120.0, 48.4, 21.2.

^{19}F NMR (471 MHz, CD$_2$Cl$_2$): δ -77.3.

Mass Spectrometry: Calculated [M$^+$] = 136.11208, Observed [M$^+$] = 136.1120; Calculated [M$^-$] = 279.91784, Observed [M$^-$] = 279.9176.

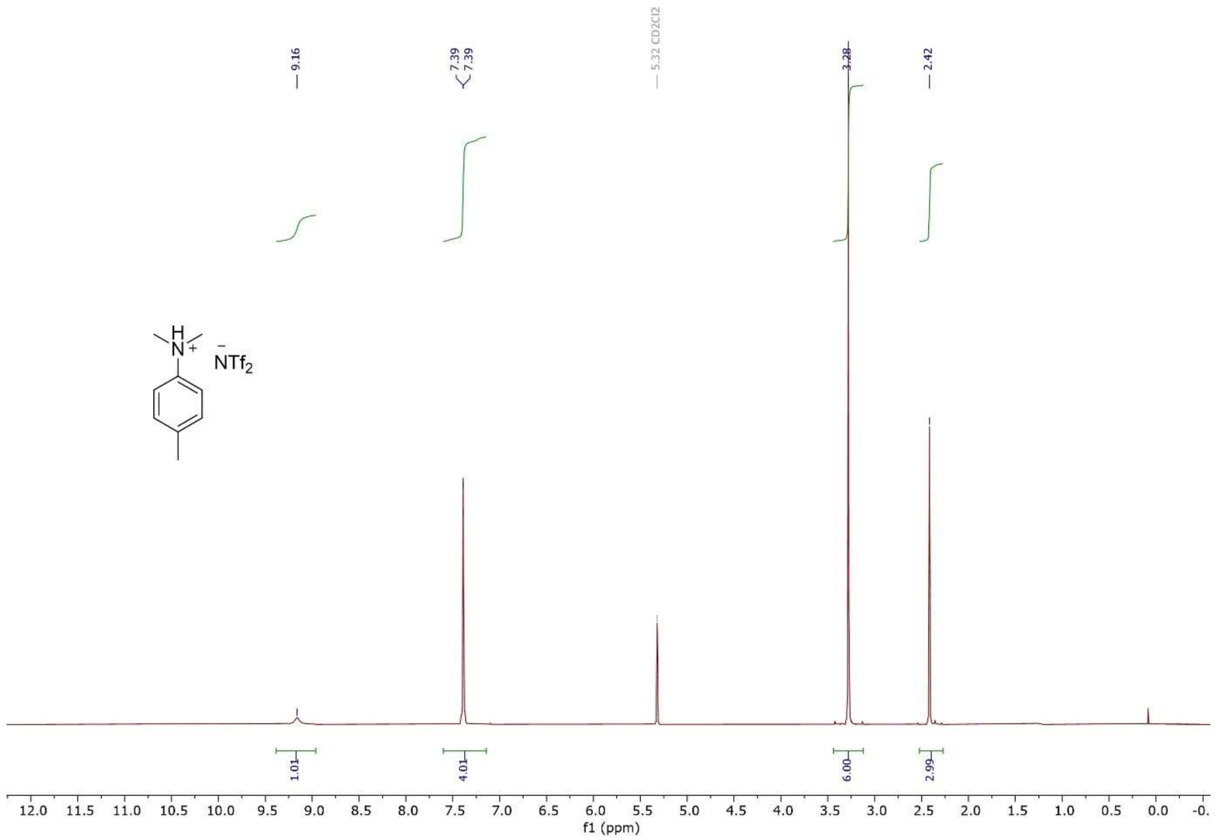

Figure S1: ^{1}H NMR spectrum of [(DMT)H][NTf_2] in CD_2Cl_2.

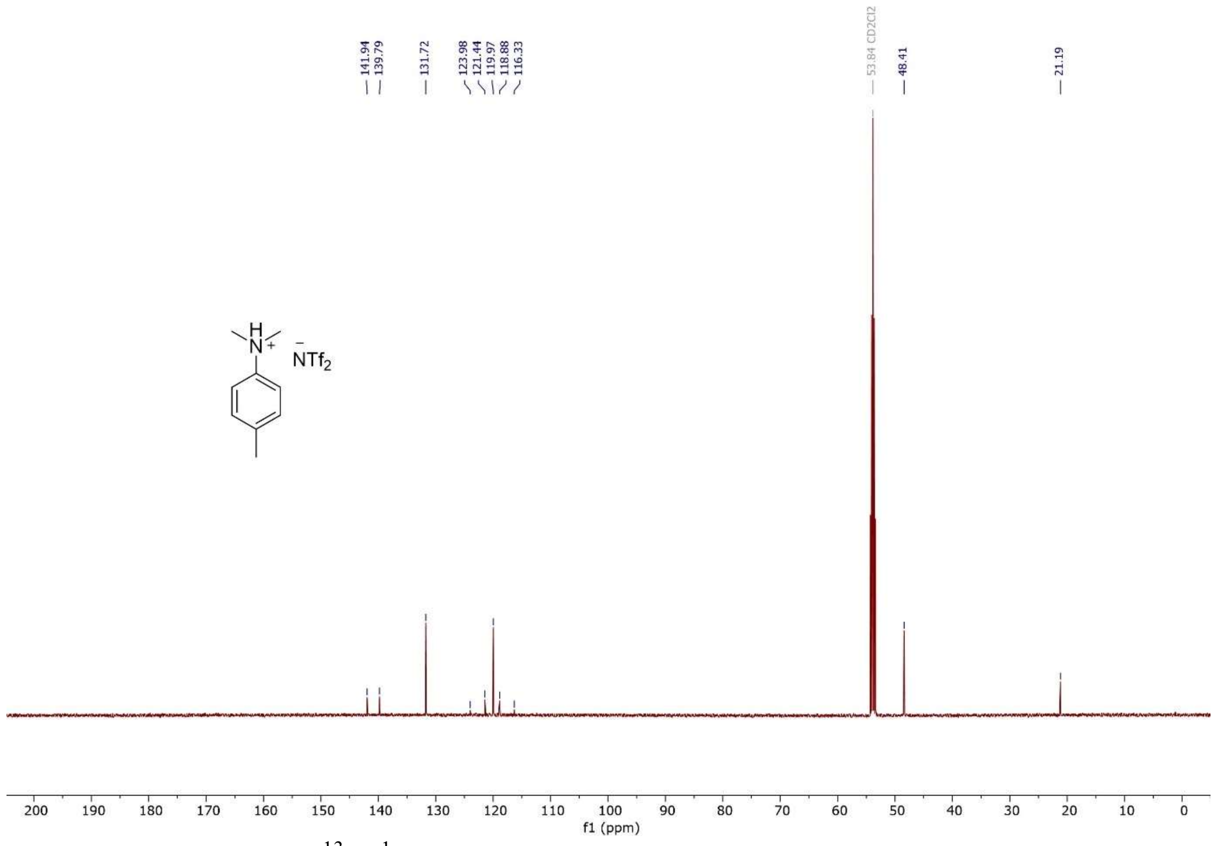

Figure S2: $^{13}C\{^{1}H\}$ NMR spectrum of [(DMT)H][NTf_2] in CD_2Cl_2.

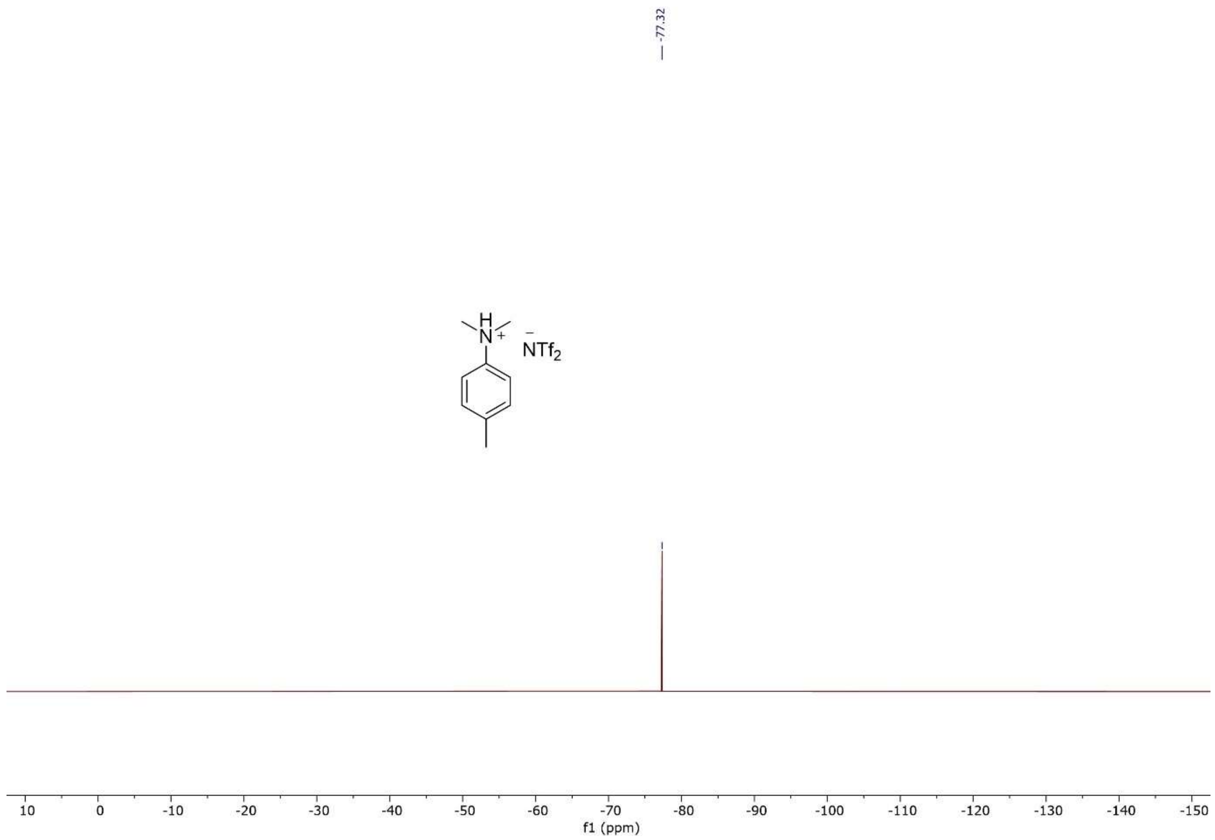

Figure S3: ^{19}F NMR spectrum of [(DMT)H][NTf_2] in CD_2Cl_2.

S3. Synthesis of aryl-BBN compounds

S3.1. General procedure 1: Optimisation for the zinc catalysed C−H borylation of 2-methyl-thiophene

In a glovebox, $[H{-}BBN]_2$ (30.5 mg, 0.125 mmol of dimer), ^{Dipp}NacNacZnH (0.0125-0.0250 mmol) and Brønsted acid (0.0125-0.0250 mmol) charged in a J. Young's NMR tube were dissolved in PhCl (0.6 mL). Subsequently, 2-methyl-thiophene (0.250-0.375 mmol, 1.0-1.5 equiv.) was added to the reaction mixture and heated at a specified temperature for a specified time. Upon completion, dibromomethane (17.5 µL, 0.250 mmol, 1.0 equivalent w.r.t. H−BBN monomer) was added to the reaction mixture as an internal standard to determine in situ yield by the integration of diagnostic ^{1}H (*Me*-thienyl-BBN) resonances.

Table S1: Zinc catalysed C−H borylation of 2-methyl-thiophene[a]

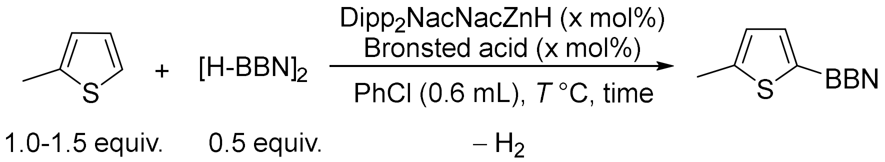

Entry	[Zn] (mol%)	Brønsted Acid	Acid (mol%)	T (°C)	Time (h)	Yield (%)[b]
**1**	-	$[(DMT)H][B(C_6F_5)_4]$	10	60	18	0.0
**2**	10	-	-	60	18	0.0
**3**	10	$[(DMT)H][B(C_6F_5)_4]$	10	rt	18	3.0
**4**	10	$[(DMT)H][B(C_6F_5)_4]$	10	60	18	55.0
**5**	10	$[(DMT)H][B\{C_6H_3(CF_3)_2\}_4]$	10	60	18	39.0
**6**	10	$[(Et_3N)H][B\{C_6H_3(CF_3)_2\}_4]$	10	60	18	6.0
**7**	10	$[(Et_3N)H][B(C_6F_5)_4]$	10	60	18	3.0
**8**	10	$[(DET)H][B(C_6F_5)_4]$	10	60	18	10.0
**9**	10	$[(Ph_3P)H][B(C_6F_5)_4]$	10	60	18	0.0
**10**	10	$[(Et_3N)H][OTf]$	10	60	18	1.0
**11**	10	[(DMT)H][OTf]	10	60	18	4.0
**12**	10	$[(DMT)H][NTf_2]$	10	60	18	1.0
**13**	10	$[(DMT)H][B(C_6F_5)_4]$	15	60	18	52.0
**14**	15	$[(DMT)H][B(C_6F_5)_4]$	10	60	18	57.0
**15**	10	$[(DMT)H][B(C_6F_5)_4]$	10	70	18	70.0
**16**	10	$[(DMT)H][B(C_6F_5)_4]$	10	80	18	87.0
**17**	10	$[(DMT)H][B(C_6F_5)_4]$	10	80	24	91.0
**18**	5	$[(DMT)H][B(C_6F_5)_4]$	5	80	24	89.0
**19**[c]	5	$[(DMT)H][B(C_6F_5)_4]$	5	80	24	87.0
**20**[d]	5	$[(DMT)H][B(C_6F_5)_4]$	5	80	24	87.0
**21**[e]	5	$[(DMT)H][B(C_6F_5)_4]$	5	80	24	99.0
**22**[e]	5	$[(DMT)H][B(C_6F_5)_4]$	5	80	18	99.0
**23**[f]	5	$[(DMT)H][B(C_6F_5)_4]$	5	80	16	83.0
**24**[f]	5	$[(DMT)H][B(C_6F_5)_4]$	5	80	24	94.0
**25**[f]	2.5	$[(DMT)H][B(C_6F_5)_4]$	2.5	80	24	84.0

[a] 2-methyl-thiophene (1.0-1.5 equiv.), [H−BBN]$_2$ (0.5 equiv.), ^{Dipp}NacNacZnH (0.05-0.1 equiv), and Brønsted acid (0.05-0.1 equiv) in PhCl (0.6 mL). [b] Yield by ^{1}H NMR spectroscopy versus CH_2Br_2 added as internal standard at the end. [c] reaction carried out in C_6D_5Br. [d] reaction carried out in 1,2-difluorobenzene. [e] reaction using 1.5 equiv. 2-methyl-thiophene. [f] reaction using 1.15 equiv. 2-methyl-thiophene.

S3.2. General procedure 2: Zinc catalysed C–H mono-borylation of (hetero)arenes

In a glovebox, [H−BBN]$_2$ (30.5 mg, 0.125 mmol of dimer, 0.5 equiv.), ^{Dipp}NacNacZnH (6.0 mg, 0.0125 mmol) and [(DMT)H][B(C_6F_5)$_4$] (10.5 mg, 0.0125 mmol) charged in a J. Young's NMR tube were dissolved in PhCl (0.6 mL). Subsequently, the corresponding heteroarene (0.287 mmol, 1.15 equiv.) was added and the reaction mixture heated at 80-100 °C for a specified time. Upon completion, dibromomethane (17.5 µL, 0.250 mmol) was added to the reaction mixture as an internal standard to determine in situ yield by the integration of diagnostic ^{1}H resonances. In cases where the diagnostic peak in ^{1}H NMR spectrum is obscured by chlorobenzene solvent, the reaction mixture was dried and redissolved in C_6D_6 to determine in situ yield upon addition of dibromomethane (17.5 µL, 0.250 mmol) as an internal standard.

For the obtaining the C−H borylated products sufficiently clean for unambiguous characterisation, volatiles were removed in vacuo and the residue was extracted in dry *n*-hexane (ca. 2 mL). The solution was filtered, dried under vacuo and submitted for NMR characterisation.

Please note, formation of O(BBN)$_2$ was observed in minor amounts due to the moisture sensitivity of [H−BBN]$_2$ and borylated heteroarenes.

S3.2.1. Synthesis of 2-(9-borabicyclo[3.3.1]nonan-9-yl)-5-methyl-thiophene, **3a**

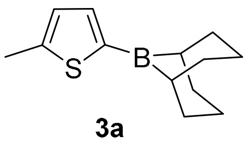

3a

As per general procedure 2, using 2-methyl-thiophene (27.5 µL, 0.287 mmol, 1.15 equiv.) and heating at 80 °C for 24 h. In situ yield by integration of diagnostic ^{1}H resonances (94% yield by ^{1}H NMR spectroscopy).

^{1}H NMR (500 MHz, C_6D_6): δ 7.65 (d, J =3.5 Hz, 1H, ^{Thienyl}C*H*), 6.75 (d, J = 3.5 Hz, 1H, ^{Thienyl}C*H*), 2.23-2.20 (m, 2H, BBN), 2.20 (d, J = 0.9 Hz, 3H, ^{Thienyl}C*H*$_3$), 2.02-1.85 (m, 12H, BBN).

^{13}C{^{1}H} NMR (126 MHz, C_6D_6): δ 152.7, 139.4, 128.9, 34.5, 30.0, 23.9, 15.7.

^{11}B NMR (160 MHz, PhCl): δ 72.1.

Note, several attempts were made to perform mass spectrometry on this compound, but these all did not show the $[M]^+$ or $[M+H]^+$.

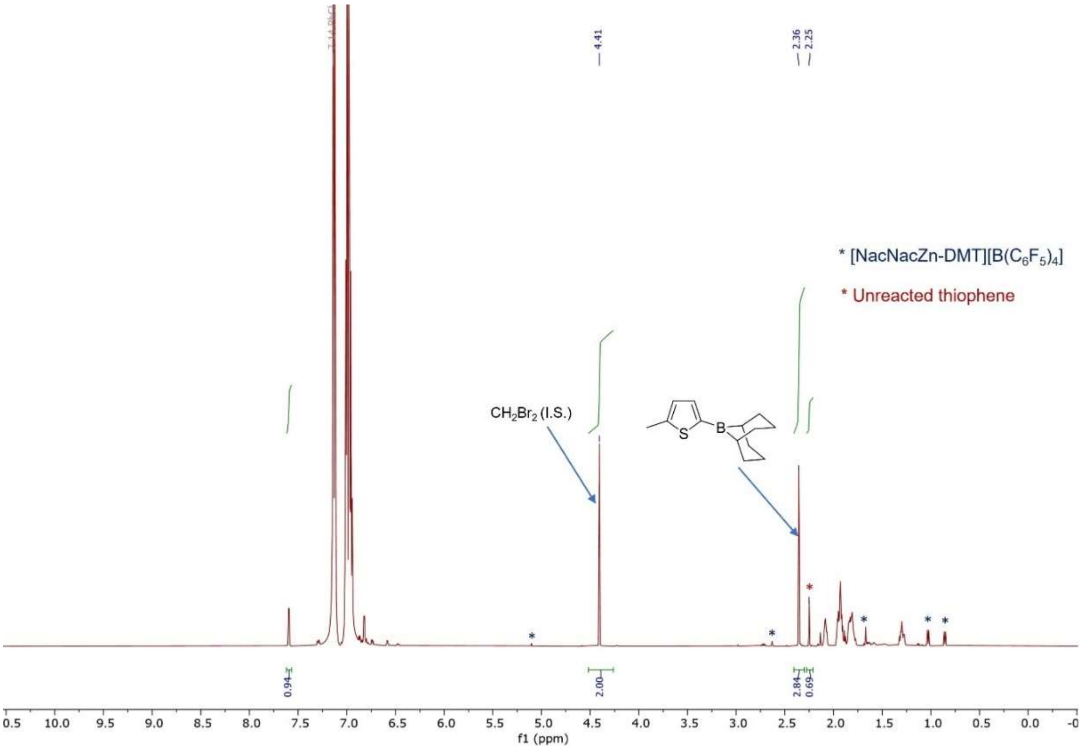

Figure S4: C–H borylation of 2-methylthiophene in PhCl by in situ ^{1}H NMR spectroscopy.

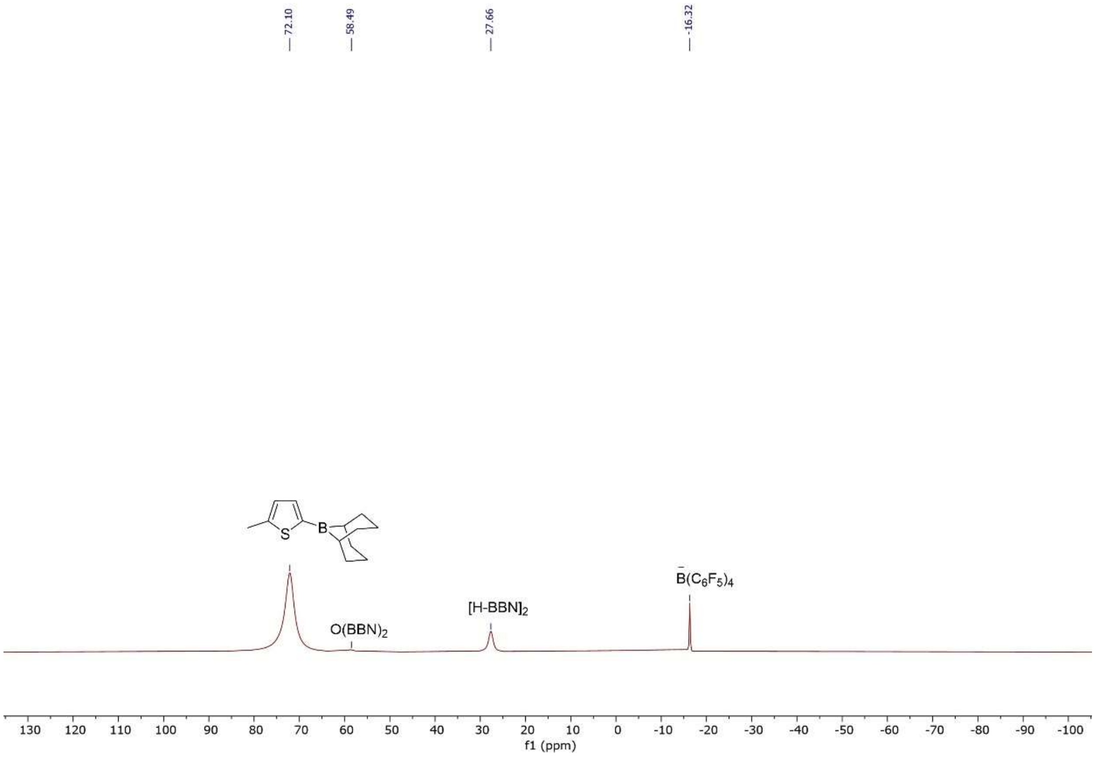

Figure S5: ^{11}B NMR spectroscopy from the crude reaction mixture in PhCl.

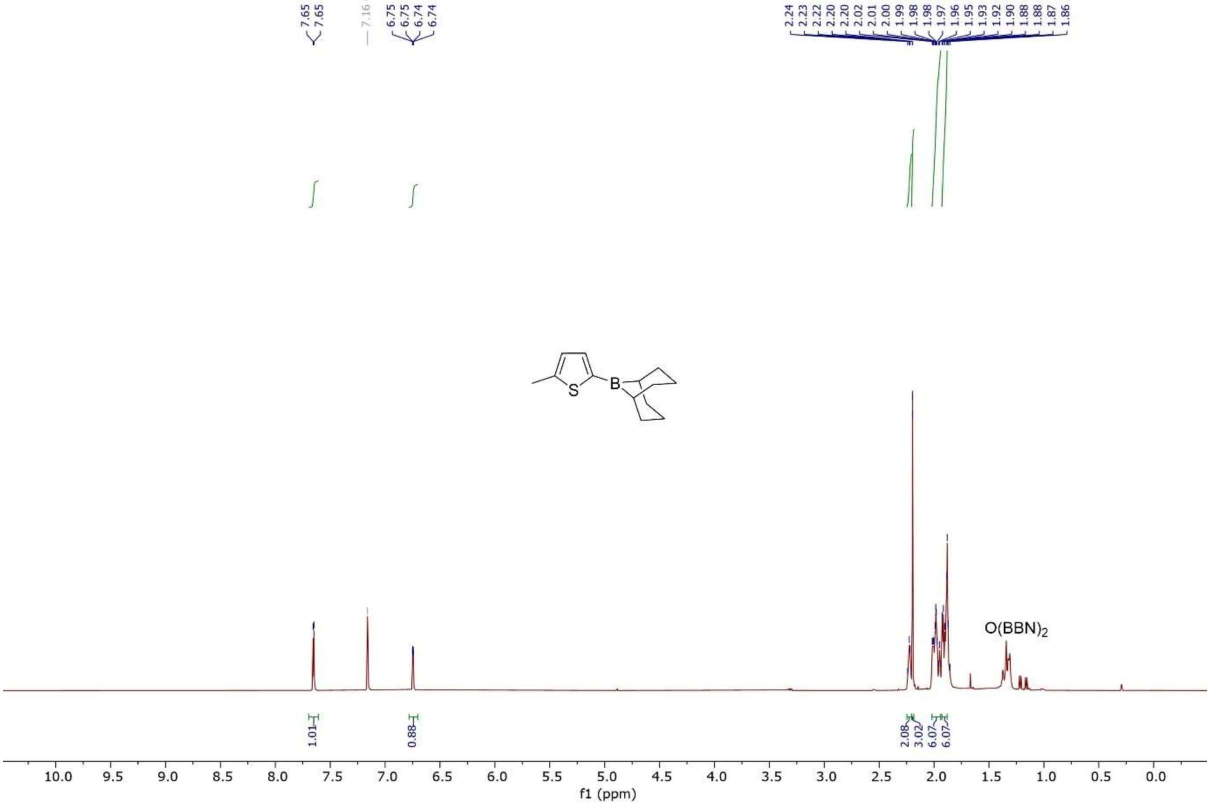

Figure S6: ^{1}H NMR spectrum of compound **3a** in C_6D_6.

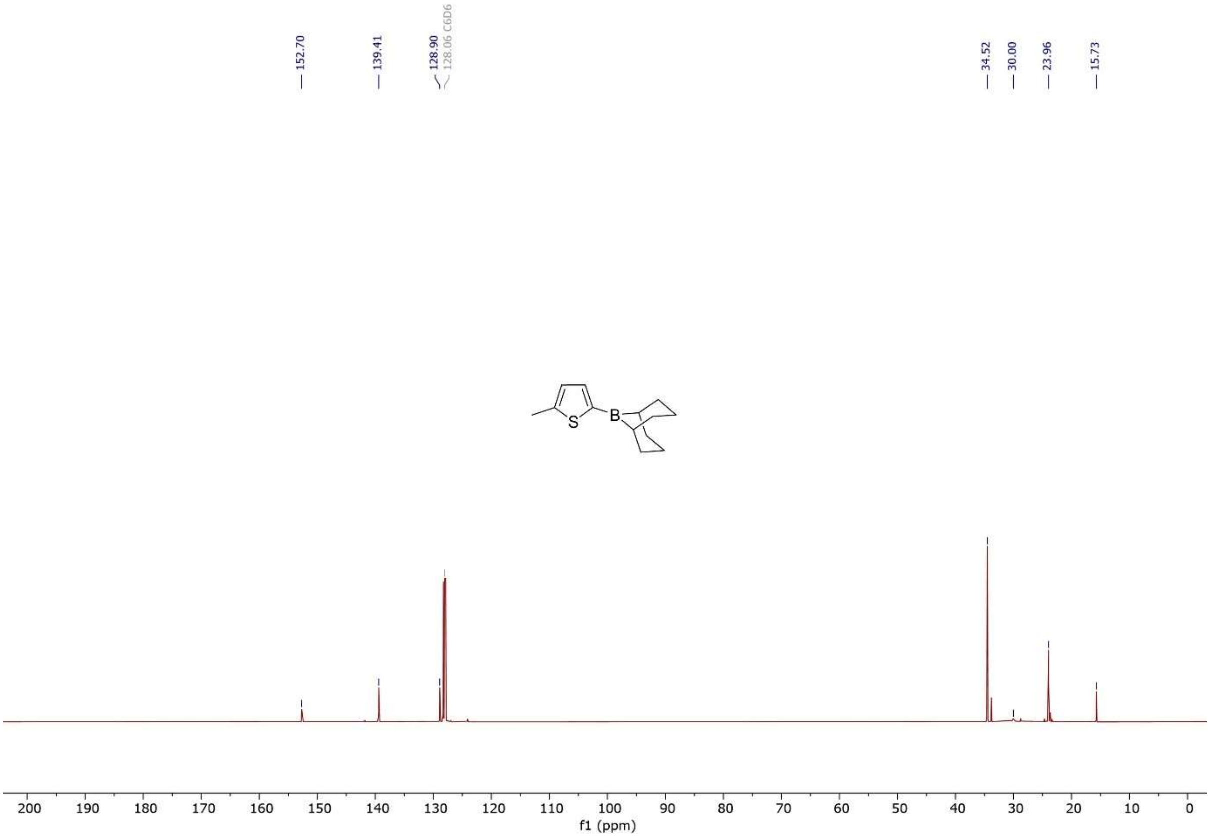

Figure S7: $^{13}C\{^{1}H\}$ NMR spectrum of compound **3a** in C_6D_6.

S3.2.2. Synthesis of 2-(9-borabicyclo[3.3.1]nonan-9-yl)-5-phenyl-thiophene, **3b**

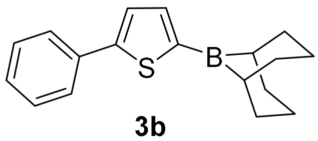

3b

As per general procedure 2, using 2-phenylthiophene (46.0 mg, 0.287 mmol, 1.15 equiv.) and heating at 80 °C for 24 h. In situ yield by integration of diagnostic ^{1}H resonances (92% yield by ^{1}H NMR spectroscopy).

^{1}H NMR (500 MHz, C_6D_6): δ 7.71 (d, J = 3.8 Hz, 1H, ^{Thienyl}C*H*), 7.60-7.57 (m, 2H, *Ph*), 7.29 (d, J = 3.6 Hz, 1H, ^{Thienyl}C*H*), 7.12-7.09 (m, 2H, *Ph*), 7.06-7.02 (m, 1H, *Ph*), 2.25-2.24 (m, 2H, BBN), 2.06-1.82 (m, 12H, BBN).

^{13}C{^{1}H} NMR (126 MHz, C_6D_6): δ 156.1, 139.8, 134.8, 129.3, 128.6, 126.7, 126.2, 34.6, 30.2, 23.9.

^{11}B NMR (160 MHz, C_6D_6): δ 73.2.

Mass spectrometry: Calculated for $[C_{18}H_{21}BS]^+$: 280.14516, found 280.14567.

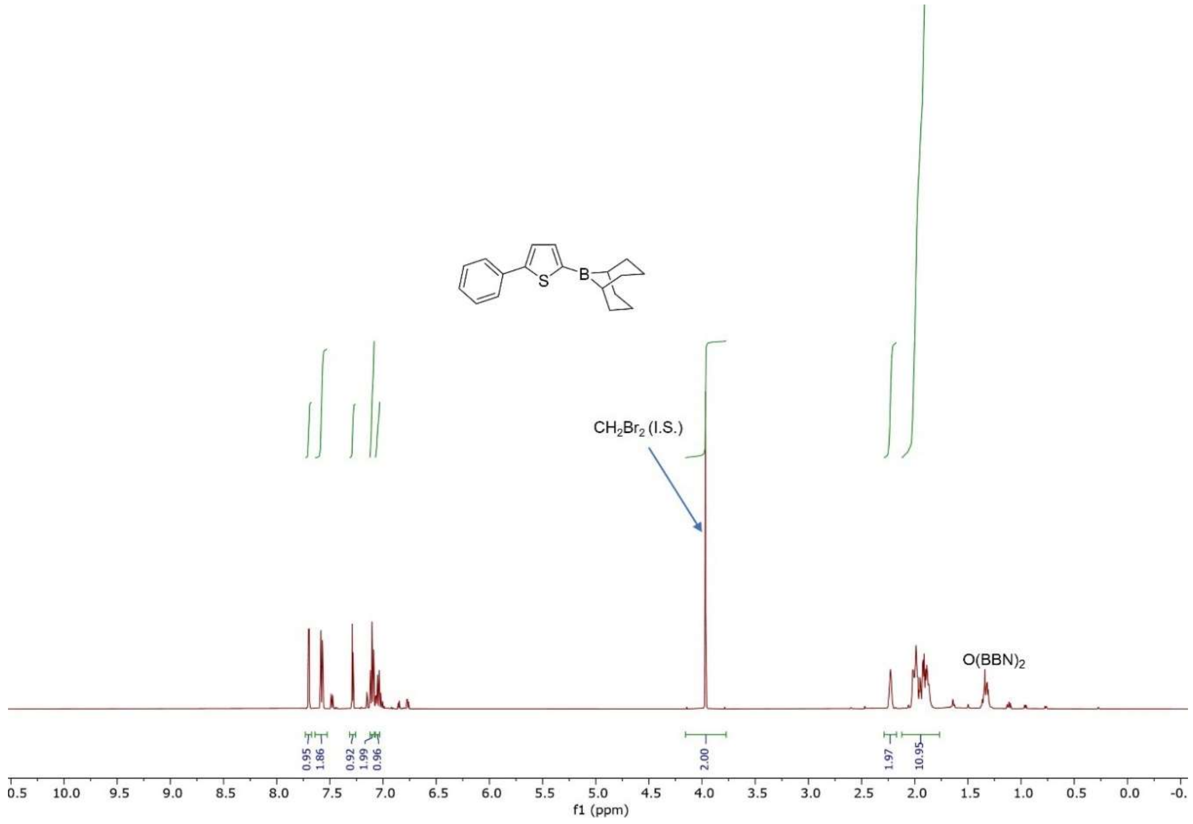

Figure S8: C–H borylation of 2-phenylthiophene in C_6D_6 for determination of NMR yield.

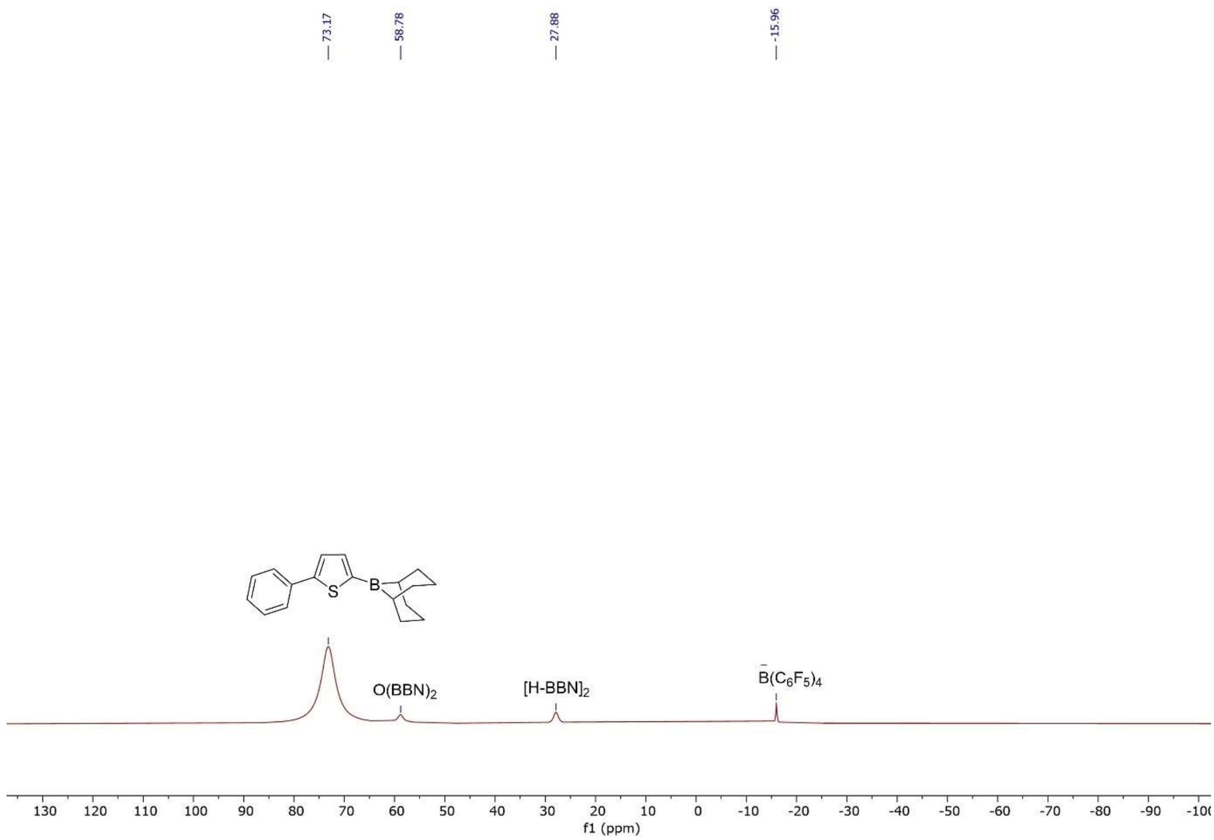

Figure S9: ^{11}B NMR spectroscopy from the crude reaction mixture in C_6D_6.

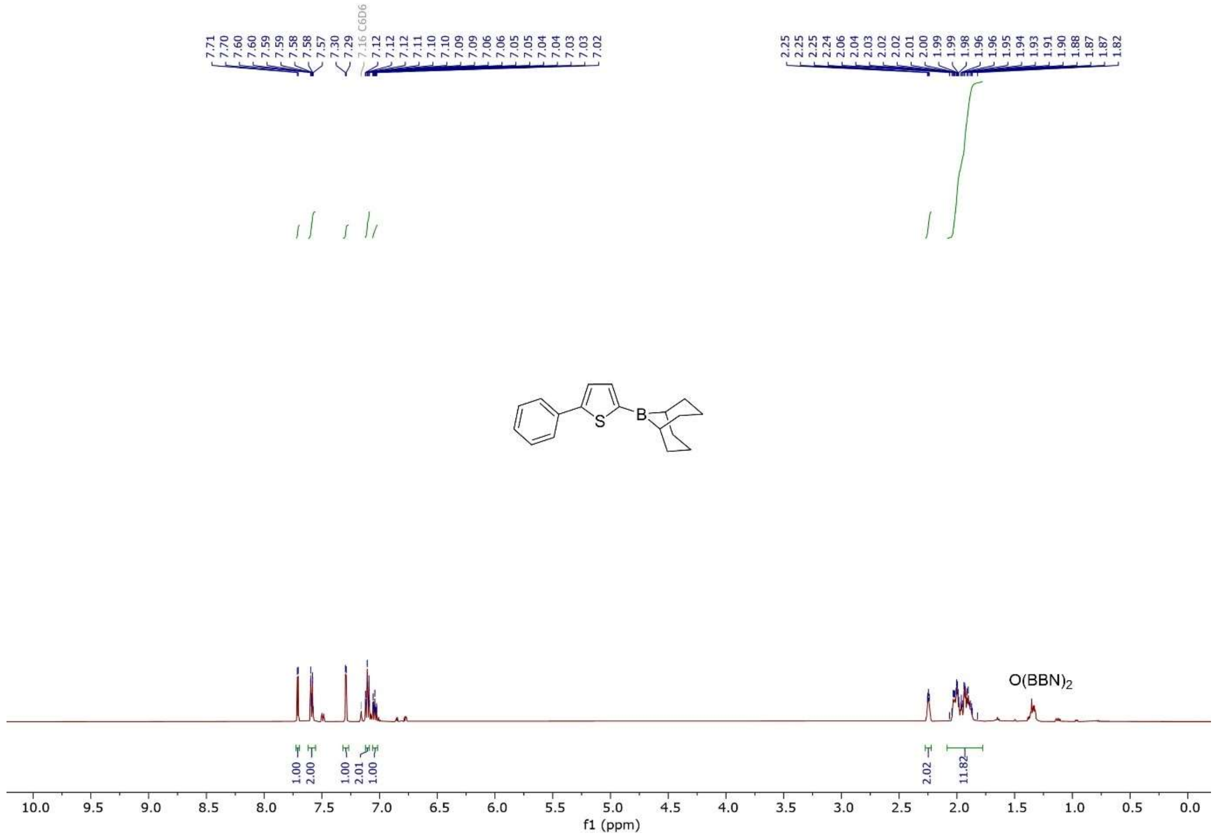

Figure S10: ^{1}H NMR spectrum of compound **3b** in C_6D_6.

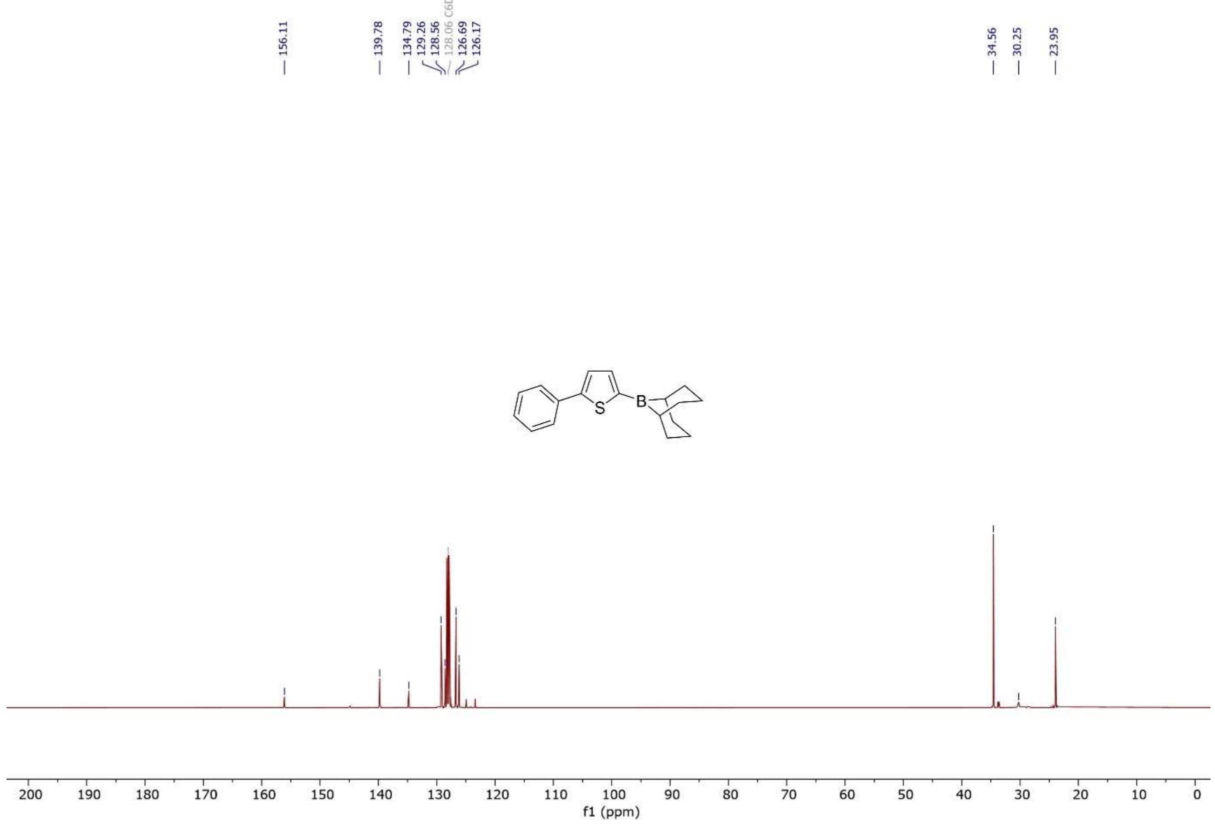

Figure S11: $^{13}C\{^1H\}$ NMR spectrum of compound **3b** in C_6D_6.

S3.2.3. Synthesis of 2-(9-borabicyclo[3.3.1]nonan-9-yl)-5-bromo-thiophene, **3c**

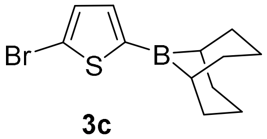

3c

As per general procedure 2, using 2-bromo-thiophene (28.0 µL, 0.287 mmol, 1.15 equiv.) and heating at 80 °C for 24 h. In situ yield by integration of diagnostic ^1H resonances (88% yield by ^1H NMR spectroscopy).

^1H NMR (500 MHz, C_6D_6): δ 7.23 (d, J = 3.8 Hz, 1H, $^{Thienyl}CH$), 6.91 (d, J = 3.6 Hz, 1H, $^{Thienyl}CH$), 2.01-1.98 (m, 2H, BBN), 1.93-1.83 (m, 8H, BBN), 1.78-1.71 (m, 4H, BBN).

$^{13}C\{^1H\}$ NMR (126 MHz, C_6D_6): δ 138.9, 133.1, 125.0, 34.4, 30.0, 23.8.

^{11}B NMR (160 MHz, C_6D_6): δ 73.0.

Mass spectrometry: Calculated for $[C_{12}H_{16}BBrS]^+$: 283.02437, found 283.27931.

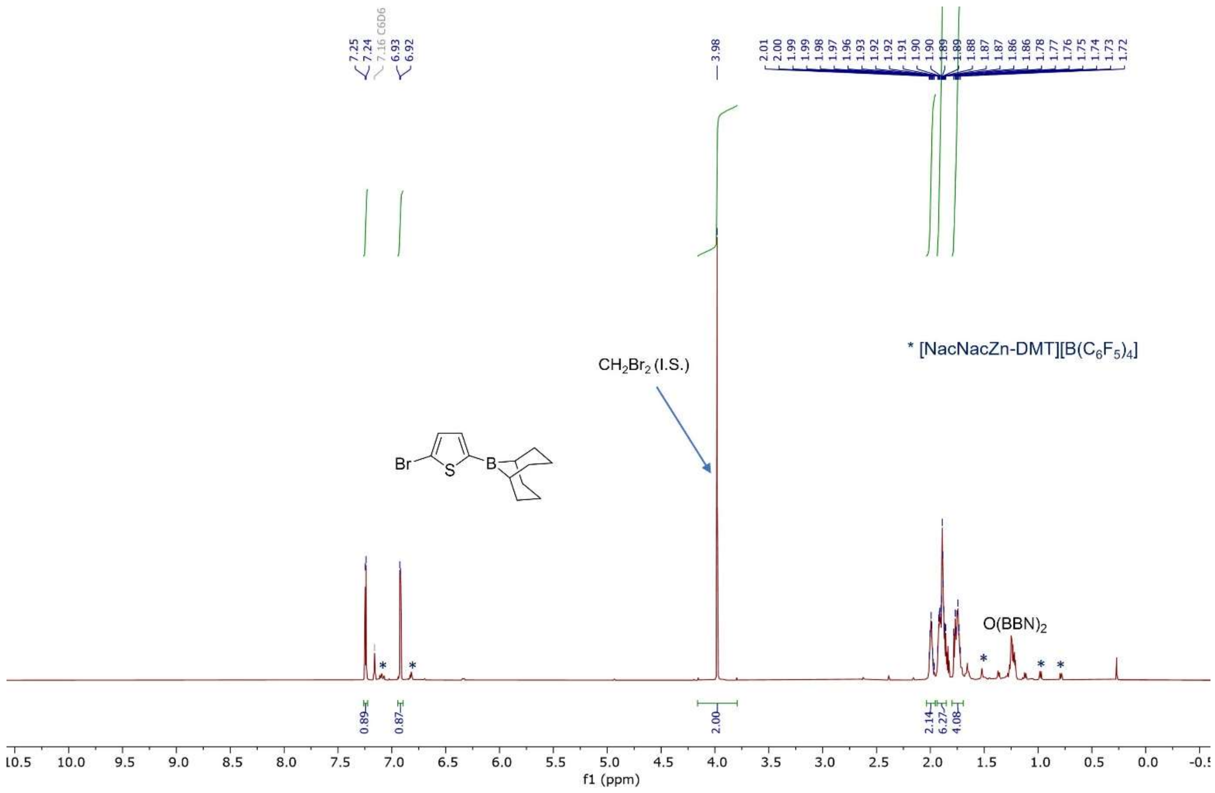

Figure S12: C–H borylation of 2-bromo-thiophene in C_6D_6 for determination of NMR yield.

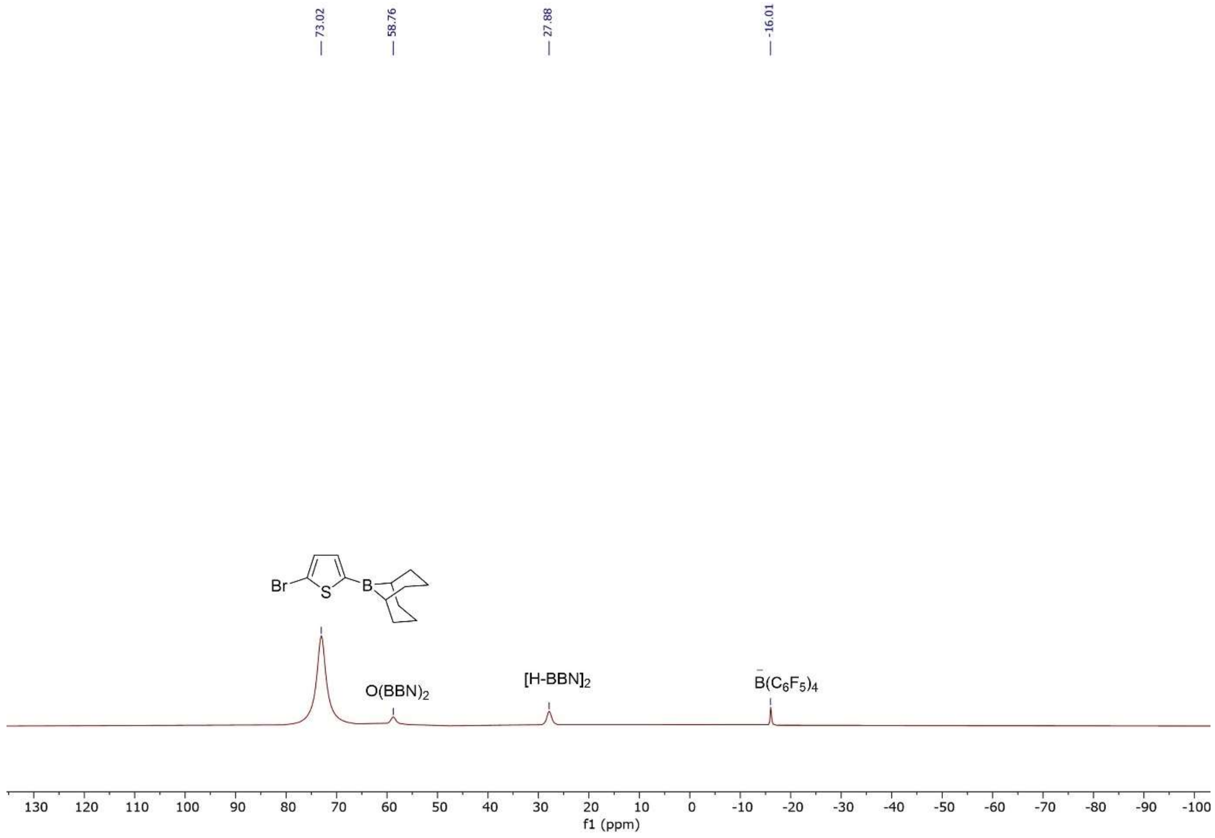

Figure S13: ^{11}B NMR spectroscopy from the crude reaction mixture in C_6D_6.

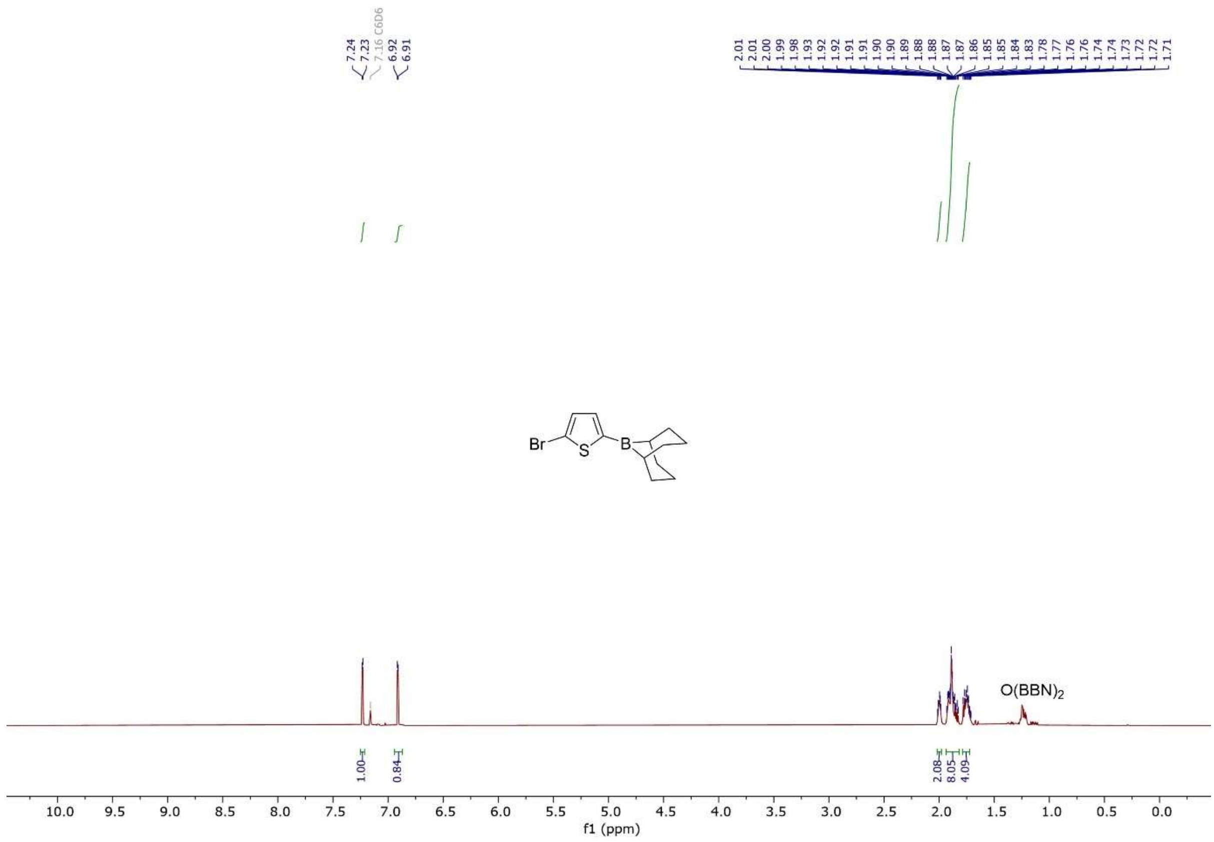

Figure S14: ^{1}H NMR spectrum of compound **3c** in C_6D_6.

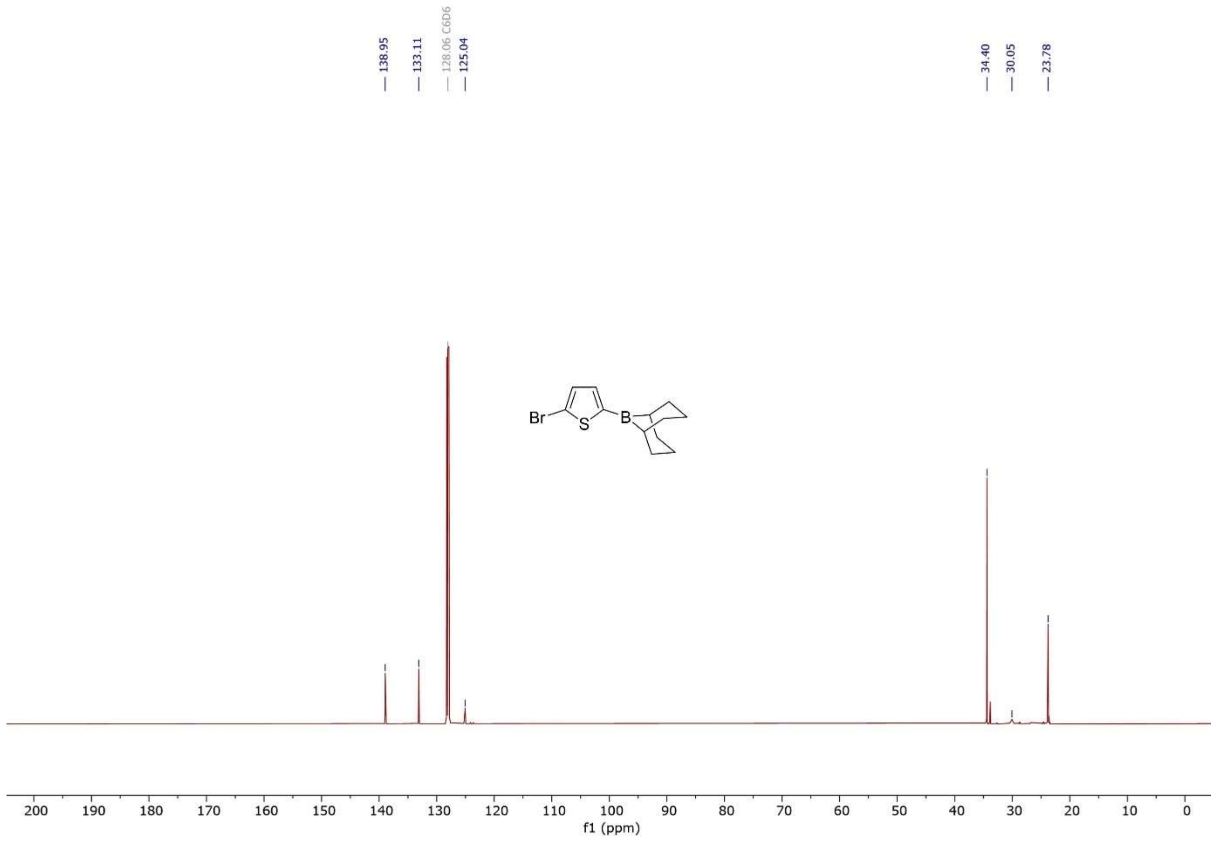

Figure S15: $^{13}C\{^{1}H\}$ NMR spectrum of compound **3c** in C_6D_6.

S3.2.4. Synthesis of 2-(9-borabicyclo[3.3.1]nonan-9-yl)-benzothiophene, **3d**

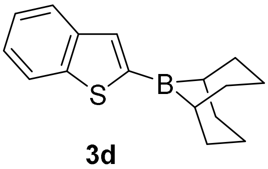

3d

As per general procedure 2, using benzothiophene (38.5 mg, 0.287 mmol, 1.15 equiv.) and heating at 100 °C for 24 h. In situ yield by integration of diagnostic ^{1}H resonances (81% yield by ^{1}H NMR spectroscopy).

^{1}H NMR (500 MHz, C_6D_6): δ 7.91 (s, 1H, ^{Thienyl}C*H*), 7.71-7.65 (m, 2H, *Ar*), 7.16-7.15 (m, 1H, *Ar*), 7.14-7.11 (m, 1H, *Ar*), 2.27 (dt, *J* = 6.6, 3.0 Hz, 2H, BBN), 2.00-1.85 (m, 12H, BBN).

^{13}C{^{1}H} NMR (126 MHz, C_6D_6): δ 146.5, 141.6, 136.0, 129.9, 128.8, 126.5, 125.7, 123.2, 34.6, 31.1, 23.9.

^{11}B NMR (160 MHz, C_6D_6): δ 75.8.

Mass spectrometry: Calculated for $[C_{16}H_{19}BS]^+$: 254.12950, found 254.12962.

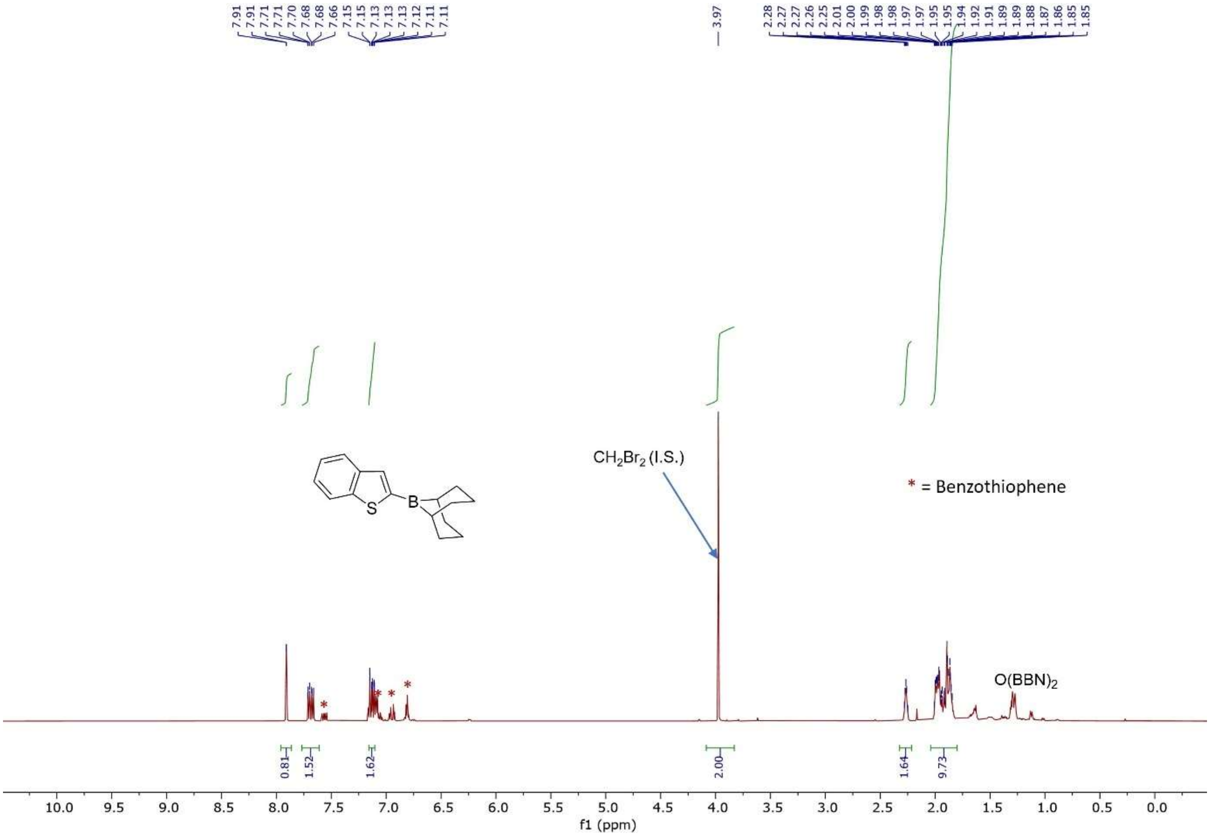

Figure S16: C–H borylation of benzothiophene in C_6D_6 for determination of NMR yield.

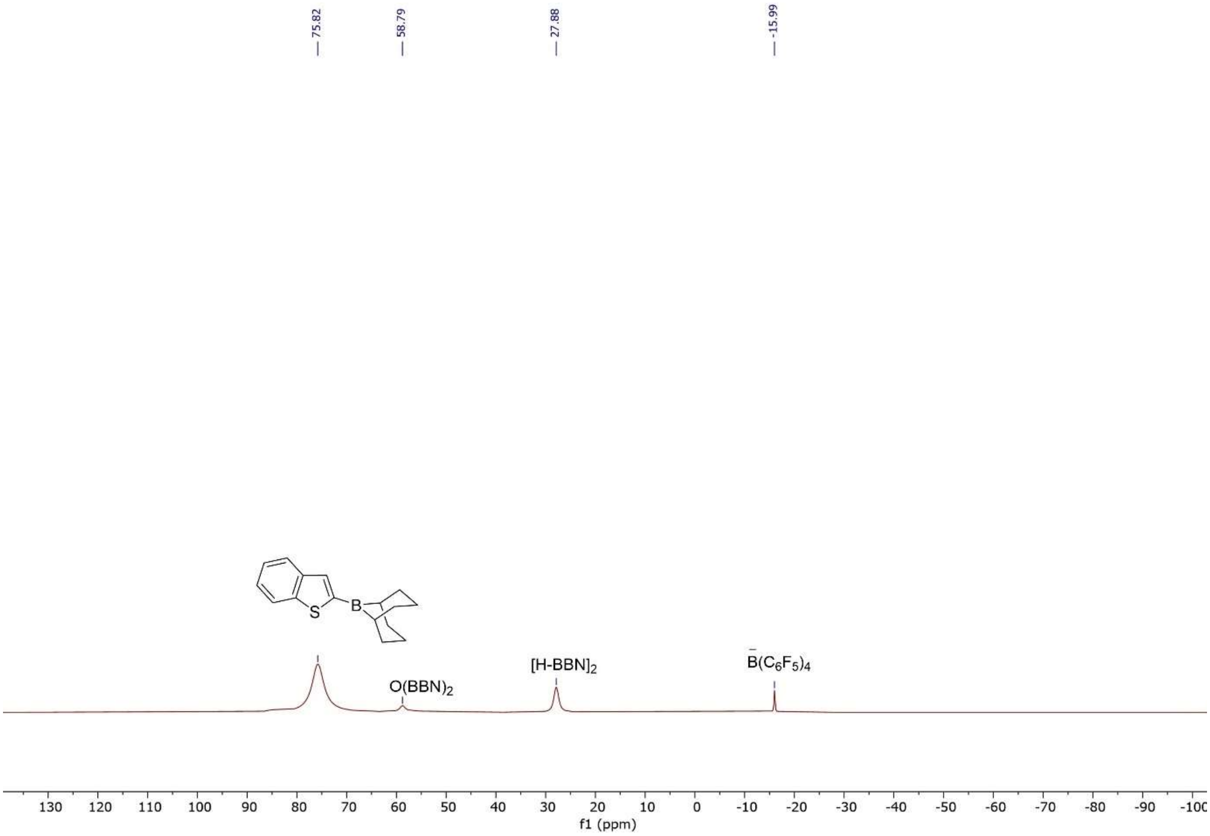

Figure S17: ^{11}B NMR spectroscopy from the crude reaction mixture in C_6D_6.

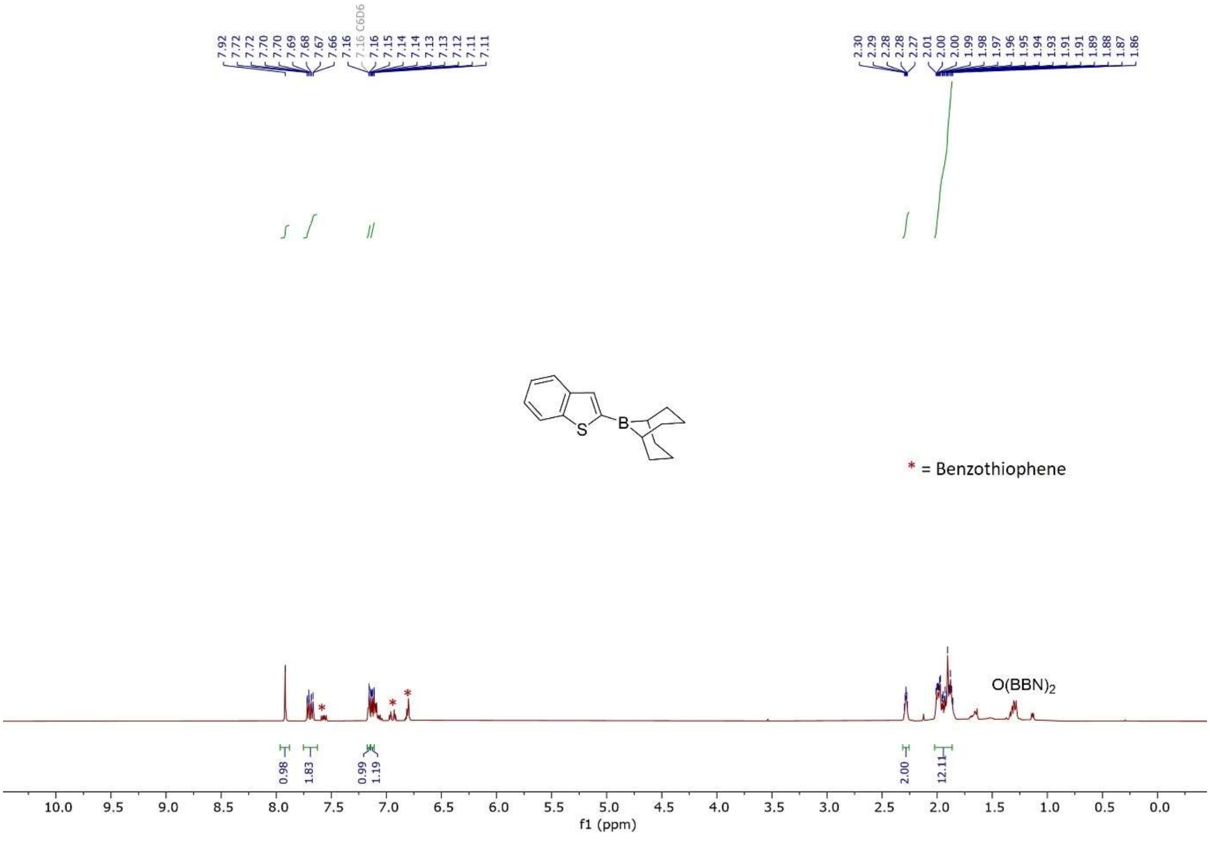

Figure S18: ^{1}H NMR spectrum of compound **3d** in C_6D_6.

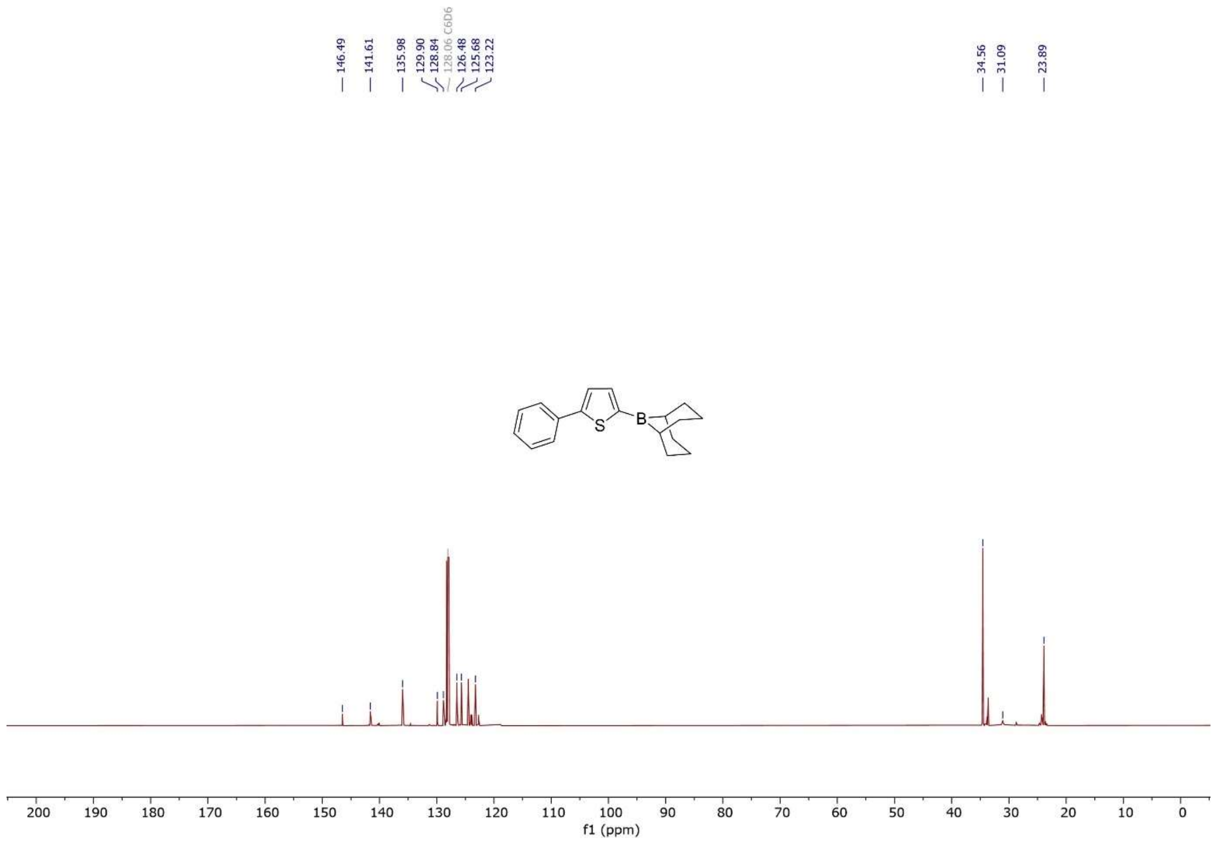

Figure S19: $^{13}C\{^{1}H\}$ NMR spectrum of compound **3d** in C_6D_6.

S3.2.5. Synthesis of 2-(9-borabicyclo[3.3.1]nonan-9-yl)-4-methyl-thiophene, **3e**

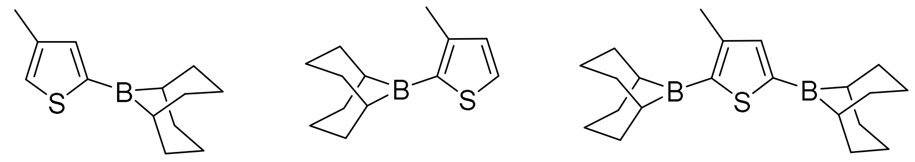

3e (76%) **3e'** (6%) **5a** (5%)

As per general procedure 2, using 3-methylthiophene (27.5 μL, 0.287 mmol, 1.15 equiv.) and heating at 80 °C for 24 h. In situ yield by integration of diagnostic ^{1}H resonances (76% yield of **3e**, 6% yield of **3e'** and 5% of **5a** by ^{1}H NMR spectroscopy).

Compound 3e

^{1}H NMR (500 MHz, C_6D_6): δ 7.55 (d, J = 1.4 Hz, 1H, $^{Thienyl}CH$), 7.10 (m, 1H, $^{Thienyl}CH$), 2.23 (p, J = 3.6 Hz, 2H, BBN), 2.04 (d, J = 1.3 Hz, 3H, $^{Thienyl}CH_3$), 2.04-1.83 (m, 12H, BBN).

$^{13}C\{^{1}H\}$ NMR (126 MHz, C_6D_6): δ 140.7, 140.3, 133.4, 34.5, 30.1, 23.9, 15.1.

^{11}B NMR (160 MHz, C_6D_6): δ 73.2.

Compound 3e'

^{1}H NMR (500 MHz, C_6D_6): *δ* 7.31 (d, *J* = 4.7 Hz, 1H, ^{Thienyl}C*H*), 6.82 (d, *J* = 4.7 Hz, 1H ^{Thienyl}C*H*), 2.36 (s 3H, ^{Thienyl}CH_3), 2.30 (m, 2H, BBN), 2.04-1.83 (m, 12H, BBN).

^{11}B NMR (160 MHz, C_6D_6): *δ* 73.2.

Note, several attempts were made to perform mass spectrometry on these compound, but these all did not show the $[M]^+$ or $[M+H]^+$.

Compound 5a

^{1}H NMR (500 MHz, C_6D_6): *δ* 7.68 (s, 1H, ^{Thienyl}C*H*), 2.43 (s, 3H, ^{Thienyl}CH_3), 2.41-2.35 (br., 4H, BBN), 2.04-1.83 (m, 24H, BBN).

^{11}B NMR (160 MHz, C_6D_6): *δ* 73.4.

Mass spectrometry: Calculated for $[C_{21}H_{32}B_2S]^+$: 338.24053, found 338.24053.

Due to them being minor products, we were unable to obtain $^{13}C\{^{1}H\}$ NMRs for **3e'** and **5a**.

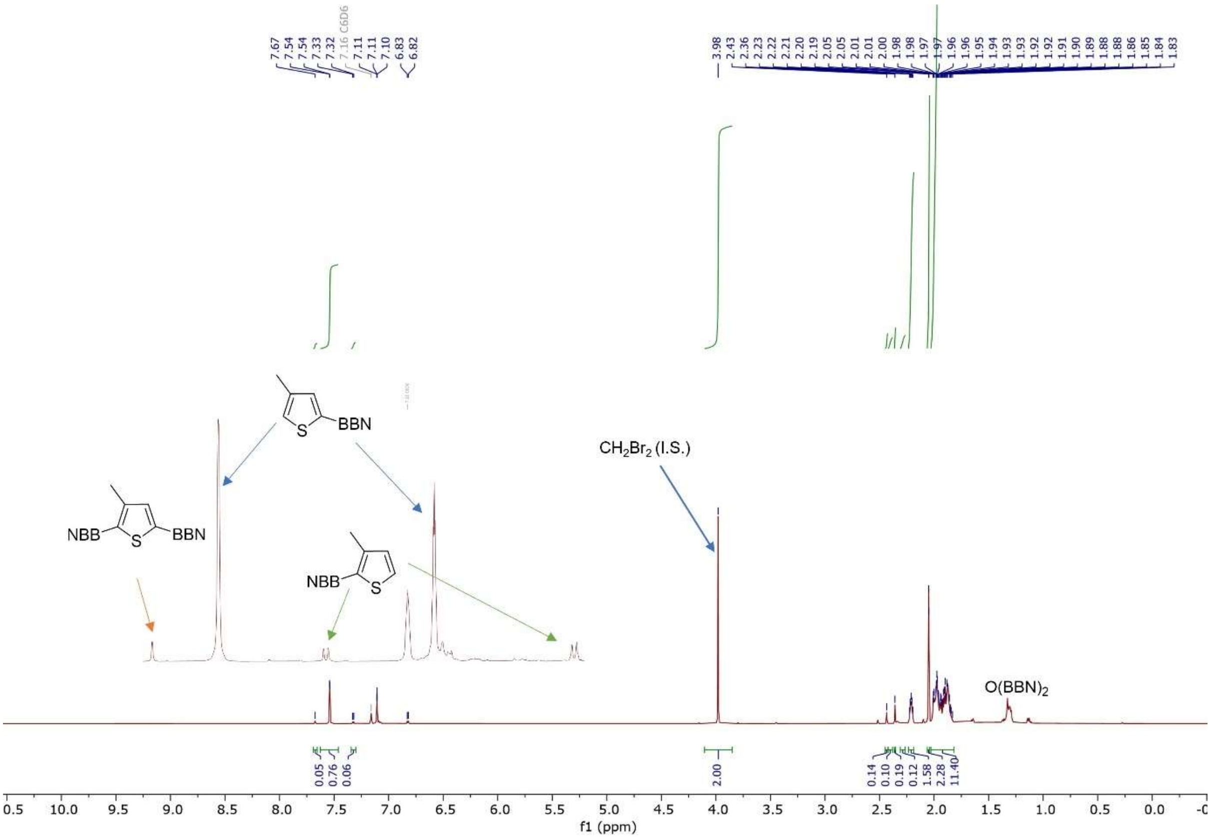

Figure S20: C–H borylation of 3-methylthiophene in C_6D_6 for determination of NMR yield.

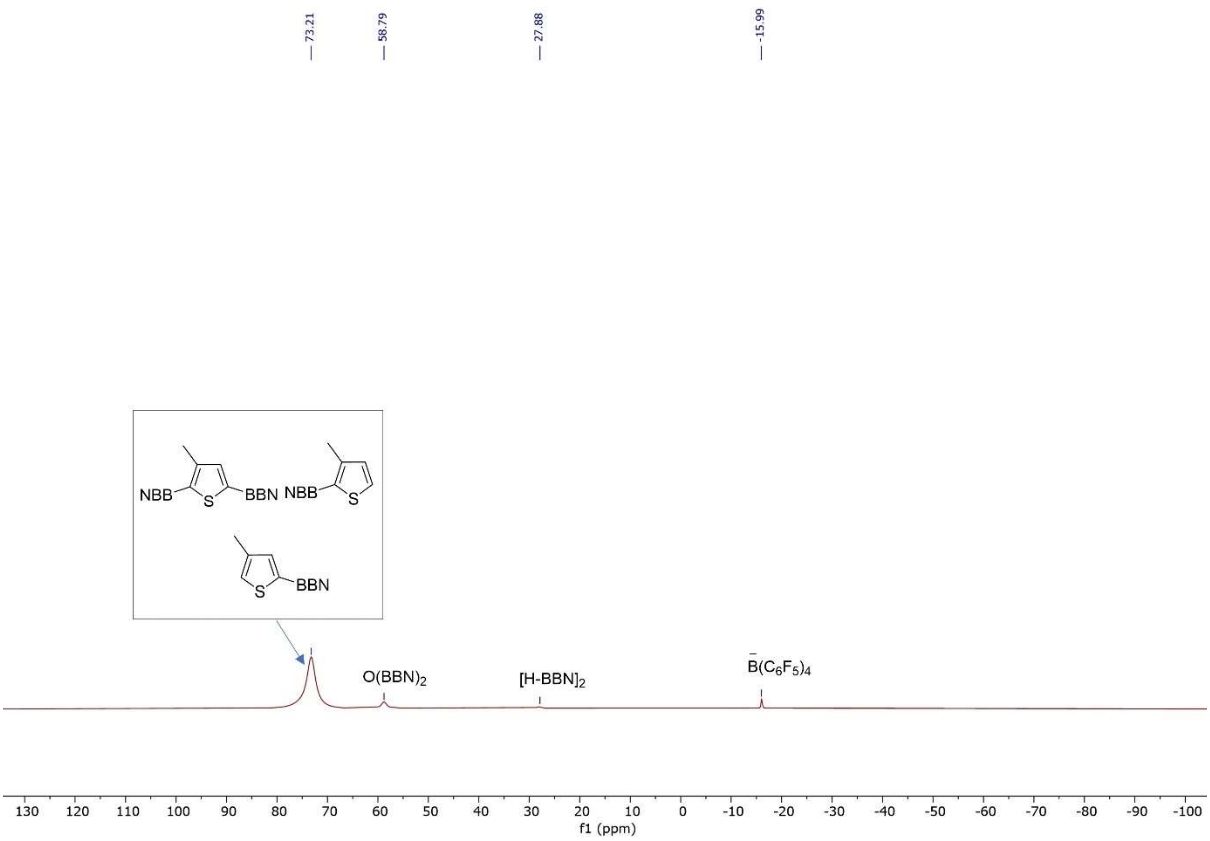

Figure S21: ^{11}B NMR spectroscopy from the crude reaction mixture in C_6D_6.

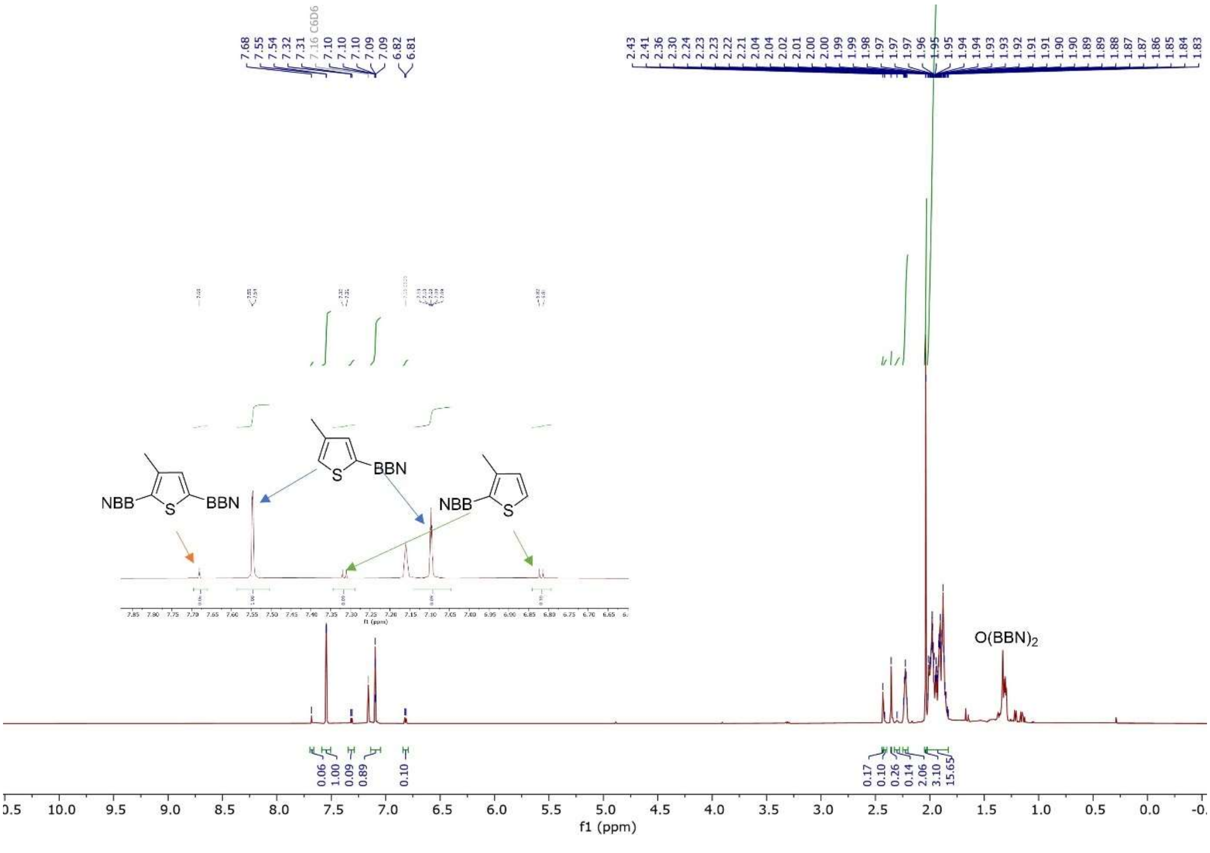

Figure S22: ^{1}H NMR spectrum of compound **3e** (with **3e'** and **5a** side product) in C_6D_6.

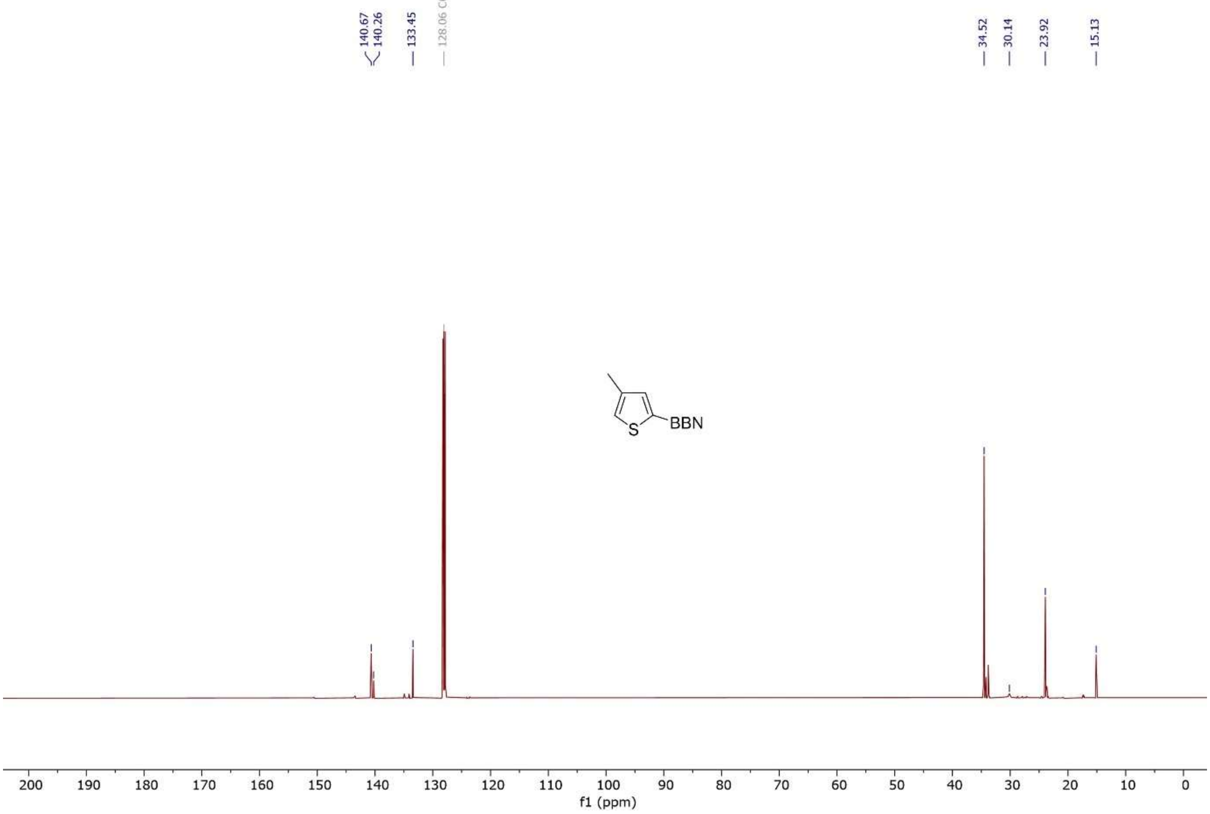

Figure S23: $^{13}C\{^{1}H\}$ NMR spectrum of compound **3e** in C_6D_6.

S3.2.6. Synthesis of 2-(9-borabicyclo[3.3.1]nonan-9-yl)-thiophene, **3f**

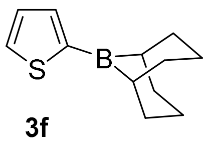

3f

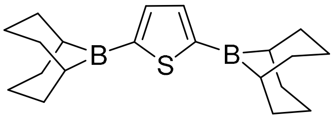

5b

As per general procedure 2, using thiophene (23.0 µL, 0.287 mmol, 1.15 equiv.) and heating at 80 °C for 24 h. In situ yield by integration of diagnostic ^{1}H resonances (68% yield of **3f** and 14% of **5b** by ^{1}H NMR spectroscopy).

Compound 3f

^{1}H NMR (500 MHz, C_6D_6): δ 7.73 (dd, J = 3.5, 1.0 Hz, 1H, $^{Thienyl}CH$), 7.44 (dd, J = 4.7, 1.0 Hz, 1H, $^{Thienyl}CH$), 7.01 (dd, J = 4.7, 3.5 Hz, 1H, $^{Thienyl}CH$), 2.21 (m, 2H, BBN), 2.02-1.80 (m, 12H, BBN).

$^{13}C\{^{1}H\}$ NMR (126 MHz, C_6D_6): δ 138.6, 137.0, 129.7, 34.5, 30.4, 23.9.

^{11}B NMR (160 MHz, C_6D_6): δ 73.4.

Analytical data are consistent with that previously reported.[7]

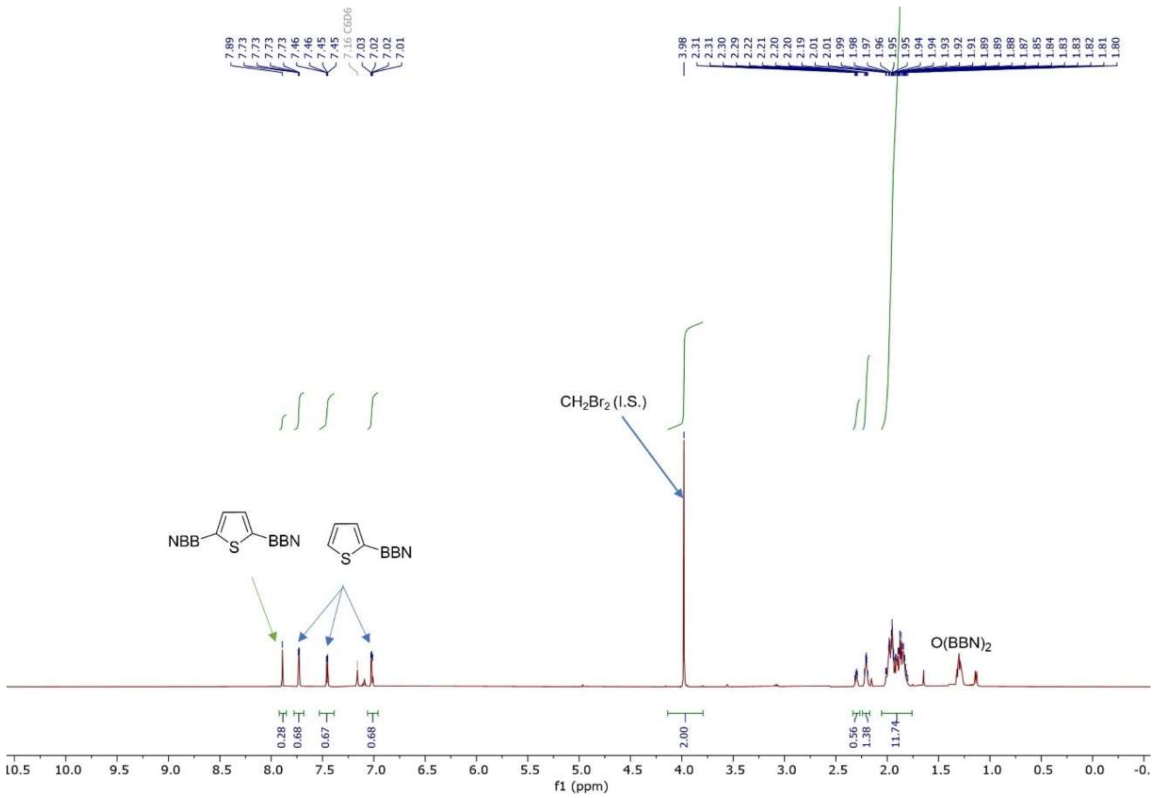

Figure S24: C–H borylation of thiophene in C_6D_6 for determination of NMR yield.

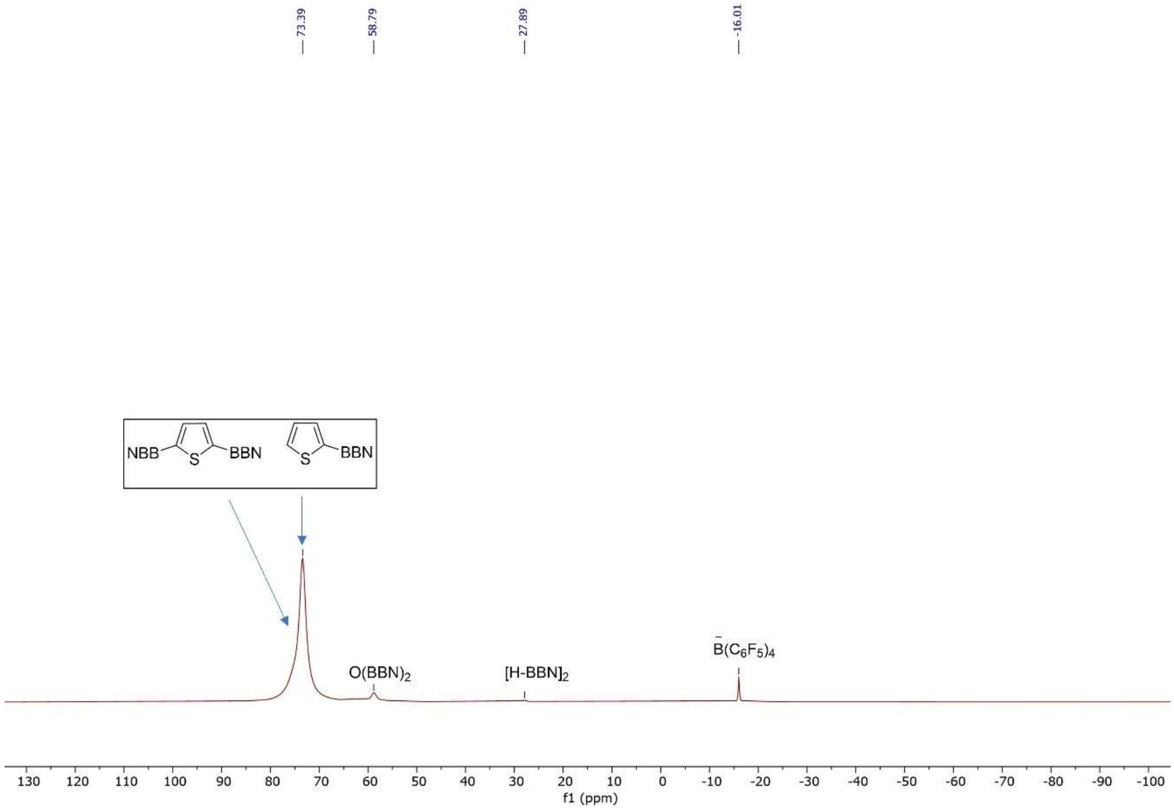

.

Figure S25: ^{11}B NMR spectroscopy from the crude reaction mixture in C_6D_6.

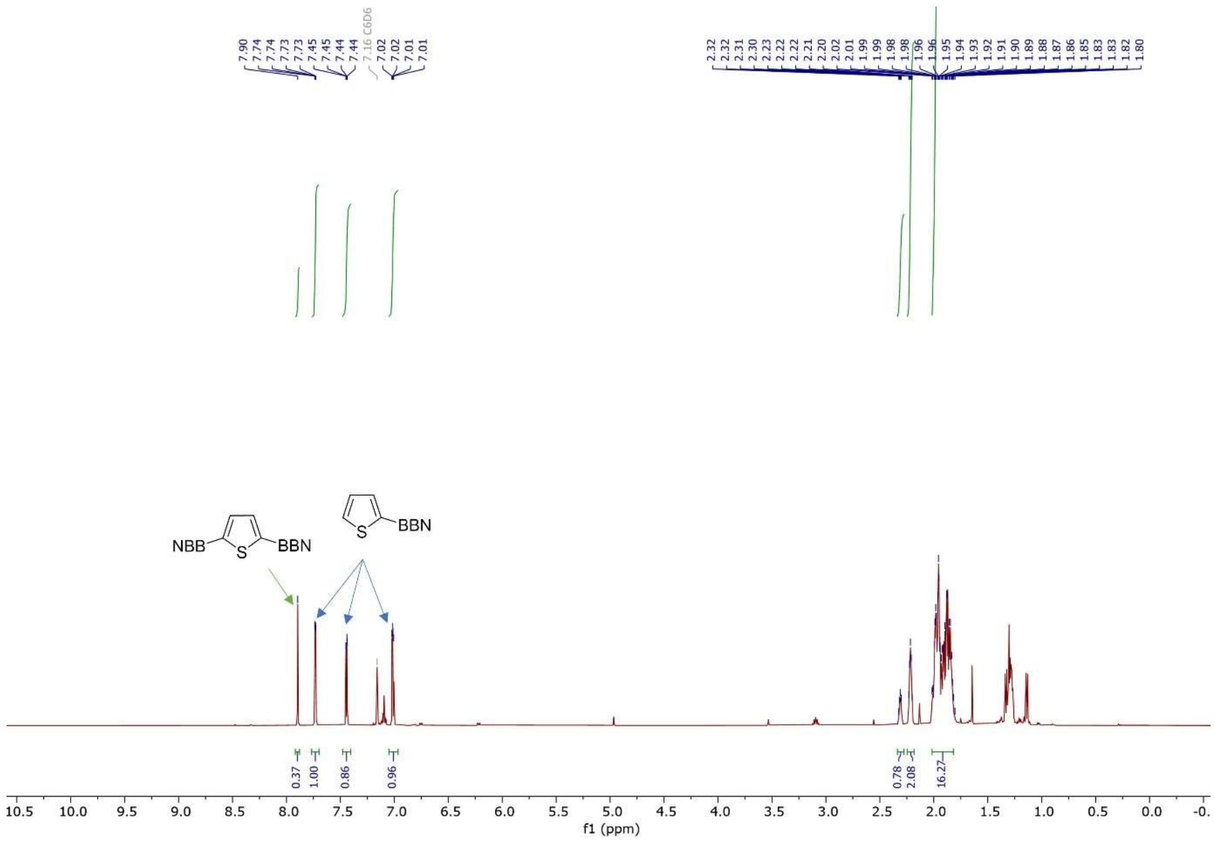

Figure S26: ^{1}H NMR spectrum of compound **3f** (with **5b** as minor product) in C_6D_6.

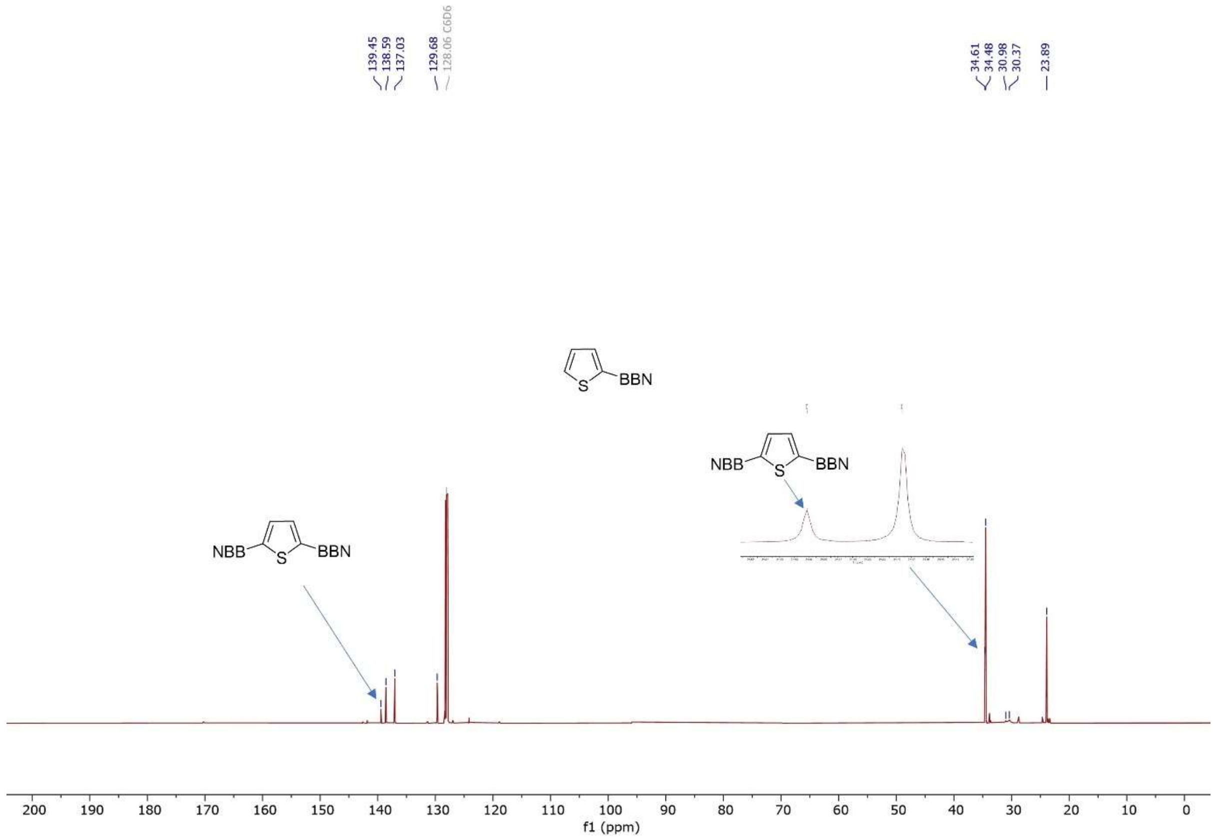

Figure S27: $^{13}C\{^{1}H\}$ NMR spectrum of compound **3f** (with **5b** as minor products) in C_6D_6.

S3.2.7. Synthesis of 2,2'-(9-borabicyclo[3.3.1]nonan-9-yl)-5,5'-bithiophene, **3g**

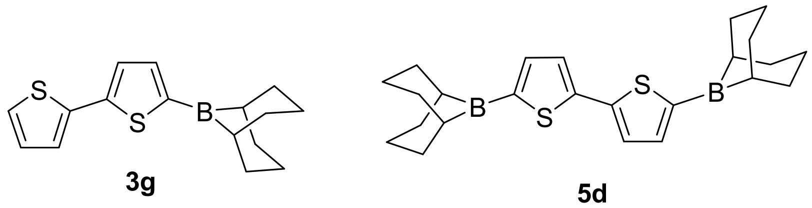

3g **5d**

As per general procedure 2, using 2,2'-bithiophene (47.5 mg, 0.287 mmol, 1.15 equiv.) and heating at 80 °C for 24 h. In situ yield by integration of diagnostic ^{1}H resonances (68% yield of **3g** and 16% of **5d** by ^{1}H NMR spectroscopy).

Compound 3g

^{1}H NMR (500 MHz, $CDCl_3$): *δ* 7.77 (d, *J* = 3.8 Hz, 1H, ^{Thienyl}C*H*), 7.41 (d, *J* = 3.8 Hz, 1H, ^{Thienyl}C*H*), 7.35 (dd, *J* = 3.6, 1.1 Hz, 1H, ^{Thienyl}C*H*), 7.30 (dd, *J* = 5.1, 1.2 Hz, 1H, ^{Thienyl}C*H*), 7.07 (dd, *J* = 5.0, 3.6 Hz, 1H, ^{Thienyl}C*H*), 2.15-2.13 (m, 2H, BBN), 2.04-2.00 (m, 6H, BBN). 1.92-1.85 (m, 6H, BBN).

^{13}C{^{1}H} NMR (126 MHz, $CDCl_3$): *δ* 148.3, 139.1, 137.6, 128.2, 126.2, 125.7, 125.0, 34.3, 30.0, 23.6.

^{11}B NMR (160 MHz, $CDCl_3$): *δ* 73.4.

Note, several attempts were made to perform mass spectrometry on this compound, but these all did not show the $[M]^+$ or $[M+H]^+$.

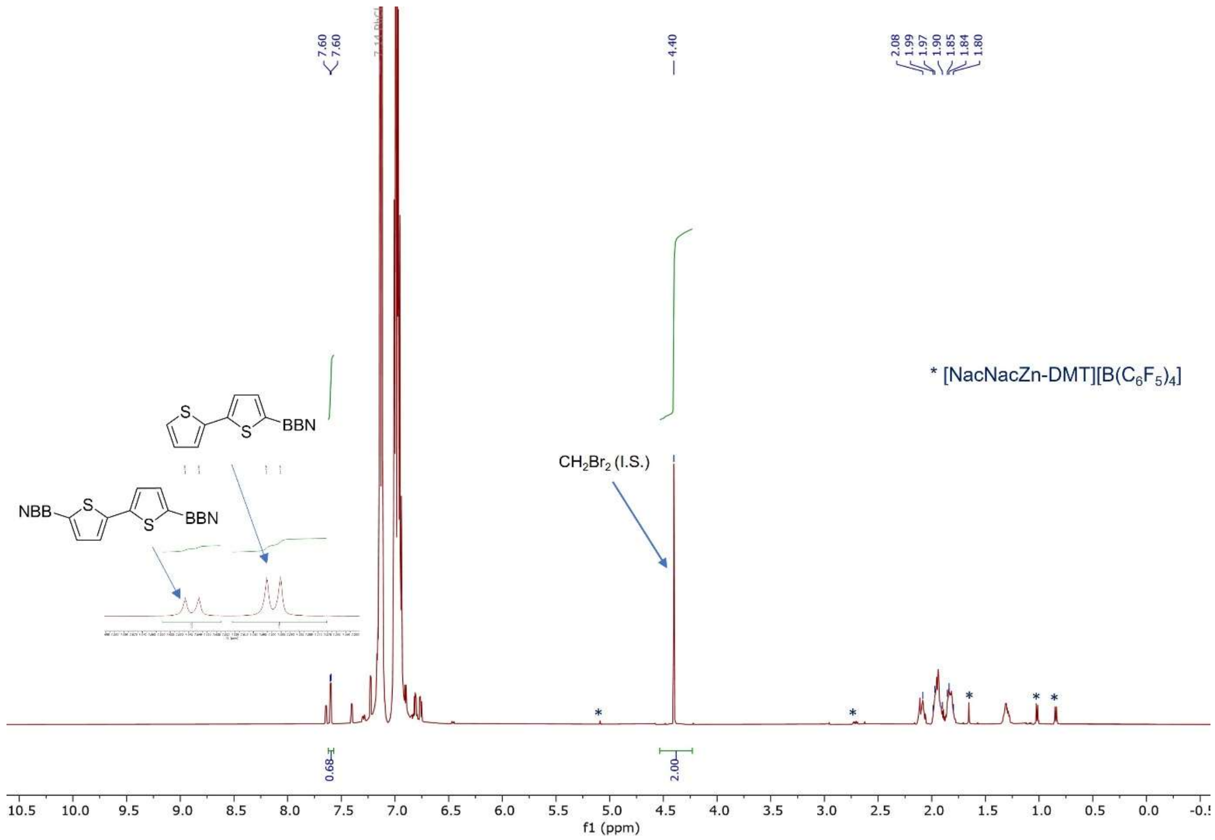

Figure S28: C–H borylation of 2,2'-thiophene in PhCl for determination of NMR yield.

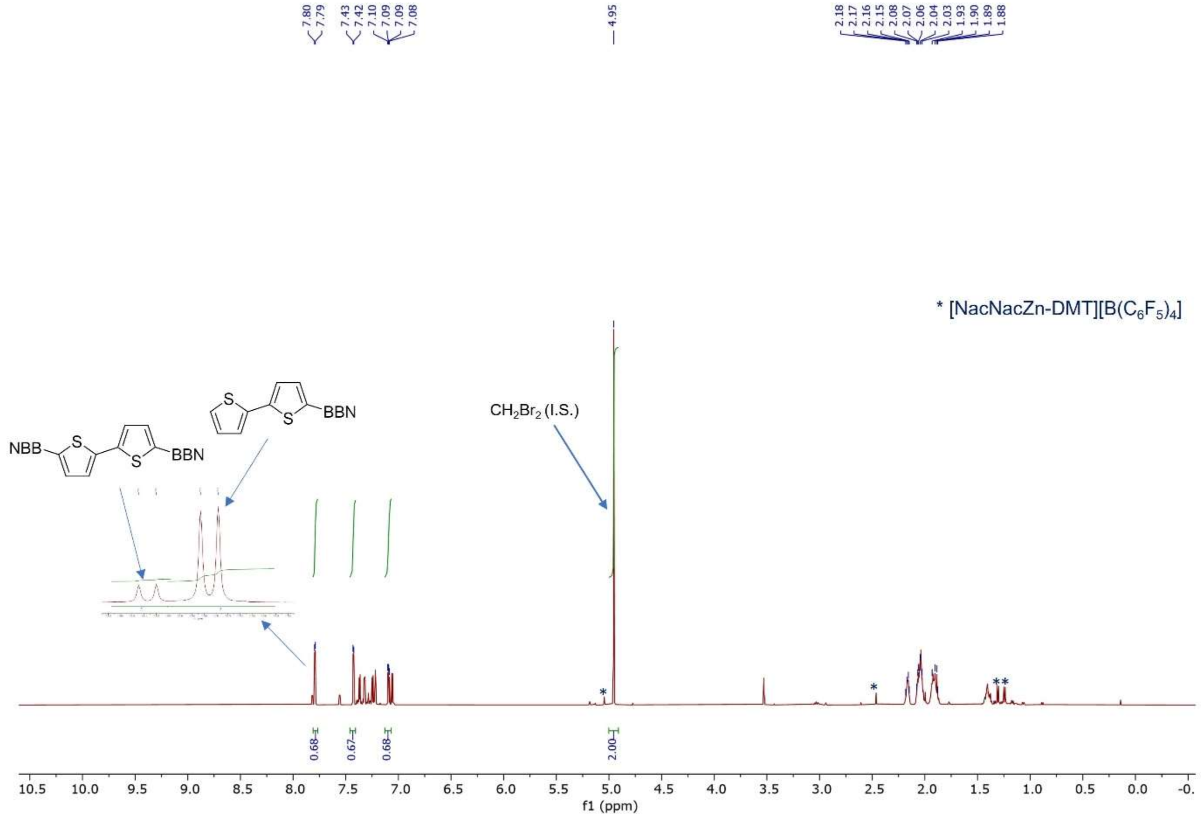

Figure S29: C–H borylation of 2,2'-thiophene in $CDCl_3$ by in situ ^1H NMR spectroscopy.

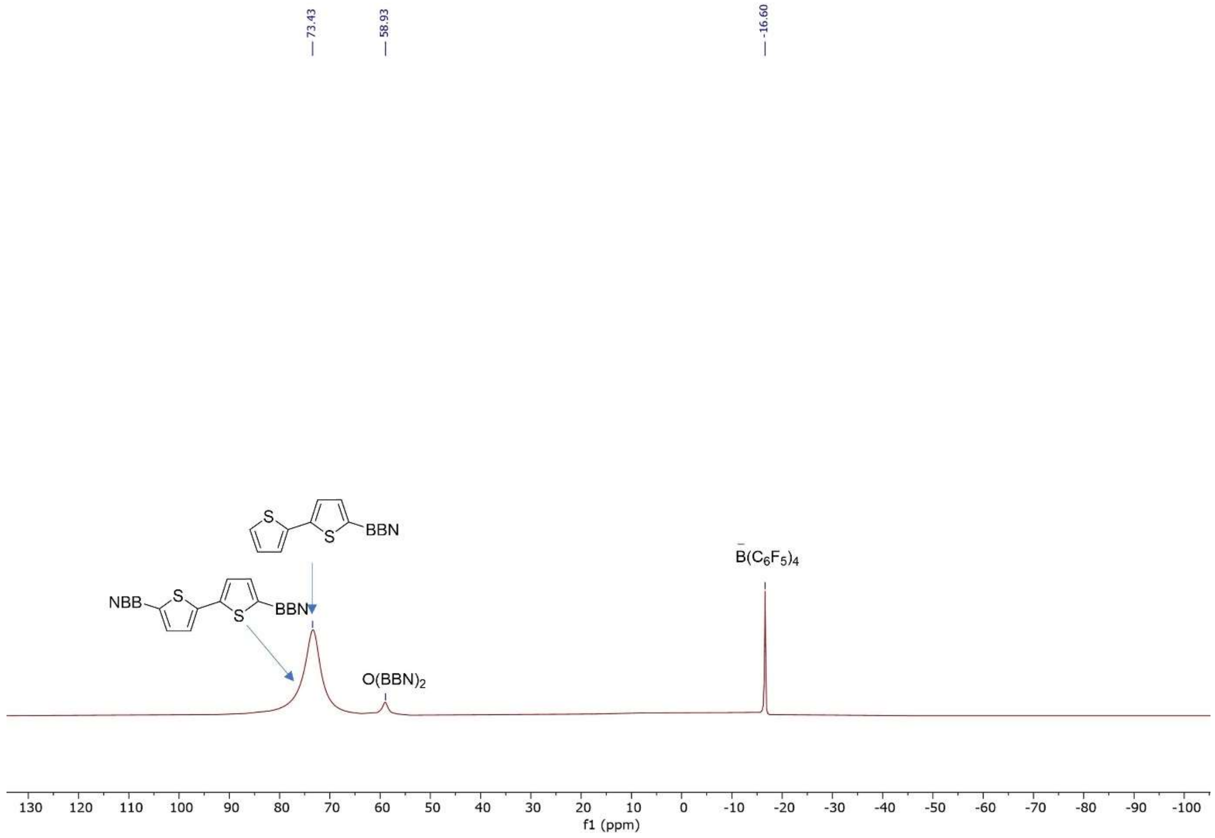

Figure S30: ^{11}B NMR spectroscopy from the crude reaction mixture in $CDCl_3$.

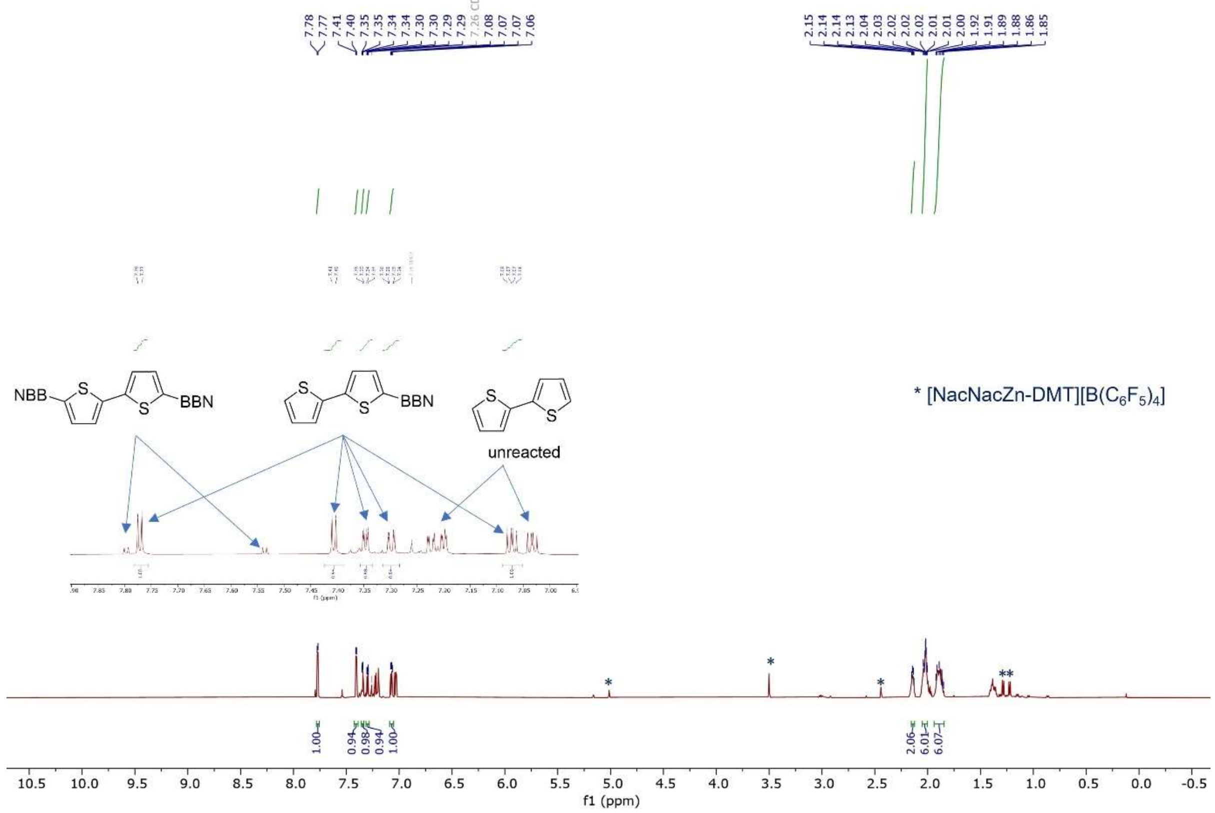

Figure S31: ^{1}H NMR spectrum of compound **3g** (with **5d** minor product) in $CDCl_3$.

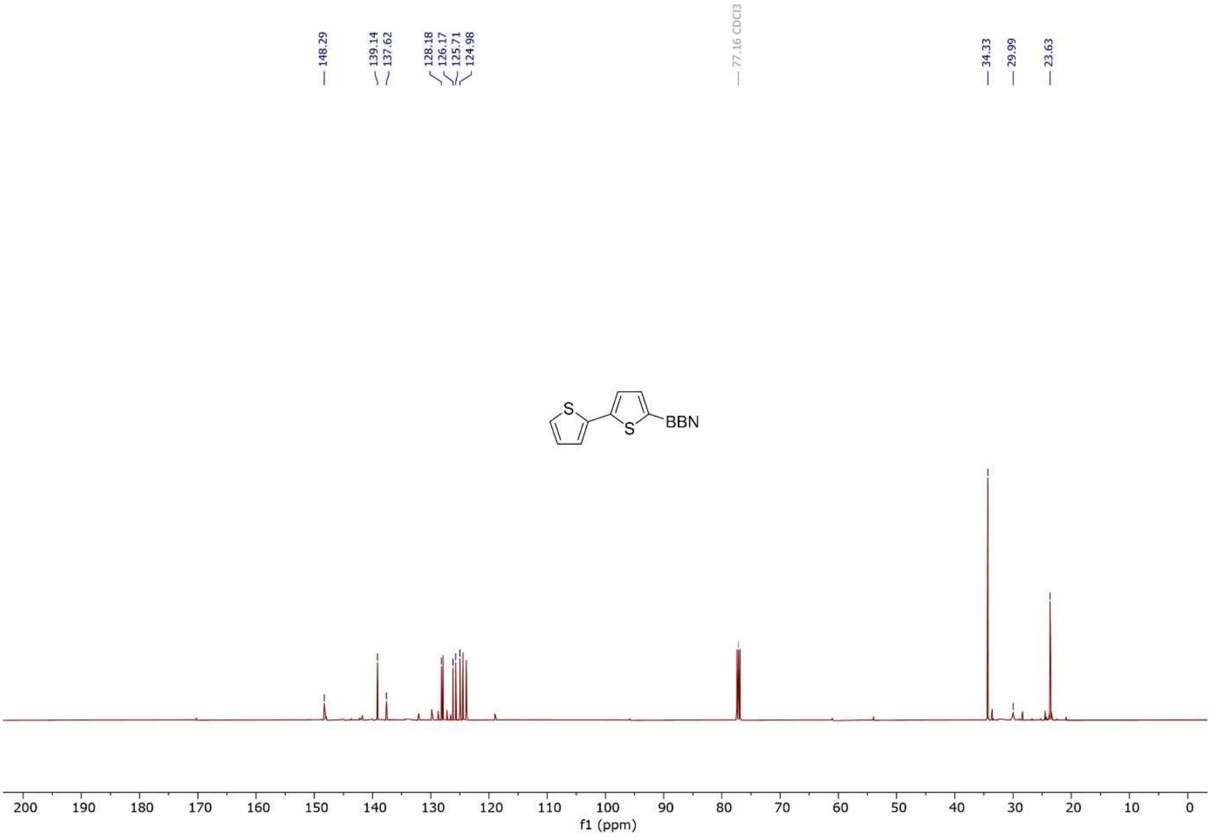

Figure S32: $^{13}C\{^{1}H\}$ NMR spectrum of compound **3g** (with **5d** minor product) in $CDCl_3$.

S3.2.8. Synthesis of 2-(9-borabicyclo[3.3.1]nonan-9-yl)-furan, **3h**

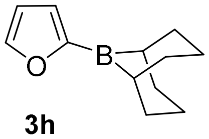

3h

As per general procedure 2, using furan (21.0 µL, 0.25 mmol, 1.0 equiv.), $[H{-}BBN]_2$ (30.5 mg, 0.125 mmol of dimer, 0.5 equiv.) and heating at 80 °C for 24 h. In situ yield by integration of diagnostic ^{1}H resonances (61% yield by ^{1}H NMR spectroscopy).

^{1}H NMR (500 MHz, C_6D_6): δ 7.42 (d, J = 1.6 Hz, 1H, $^{Furanyl}CH$), 7.20 (d, J = 3.3 Hz, 1H, $^{Furanyl}CH$), 6.19 (dd, J = 3.5, 1.6 Hz, 1H, $^{Furanyl}CH$), 2.30 (br., 2H, BBN), 1.99-1.94 (m, 6H, BBN), 1.89-1.83 (m, 6H, BBN).

$^{13}C\{^{1}H\}$ NMR (126 MHz, C_6D_6): δ 150.3, 127.4, 111.8, 34.3, 28.2, 23.8.

^{11}B NMR (160 MHz, C_6D_6): δ 71.1.

Note, several attempts were made to perform mass spectrometry on this compound, but these all did not show the $[M]^+$ or $[M+H]^+$.

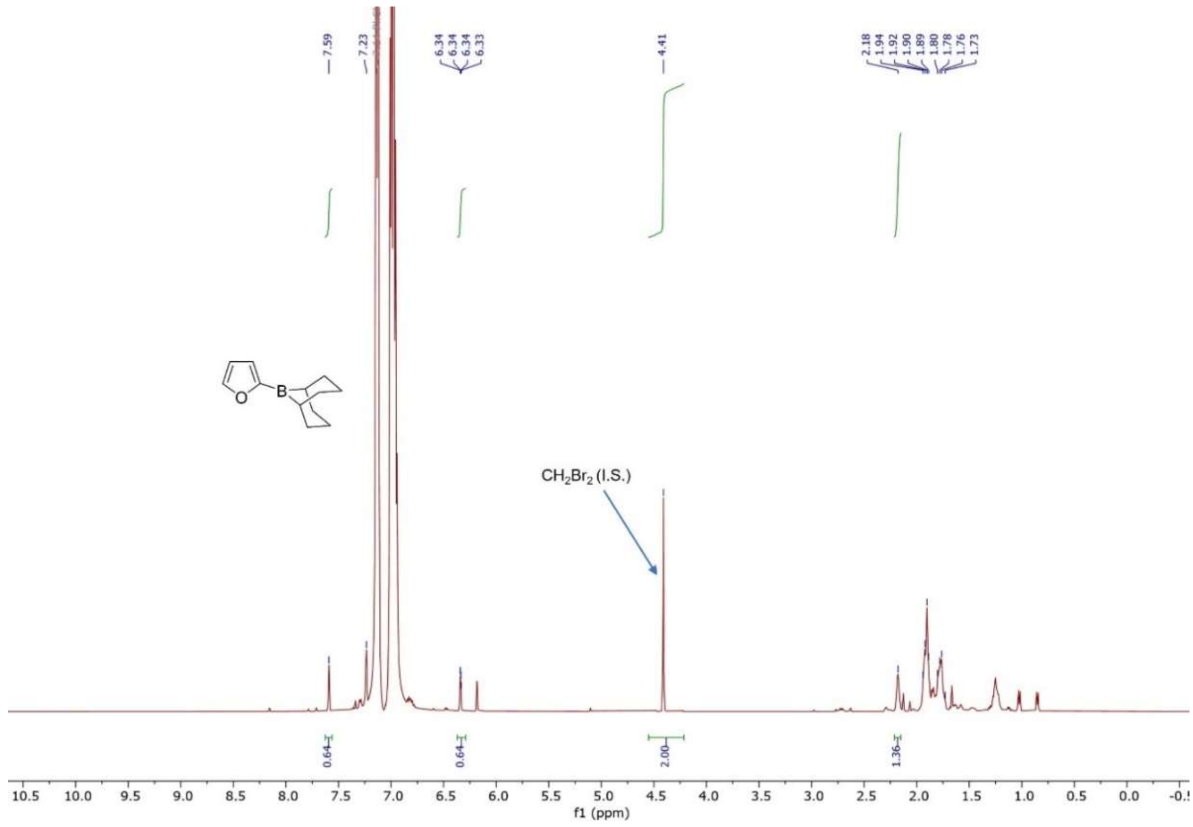

Figure S33: C–H borylation of furan in PhCl for determination of NMR yield.

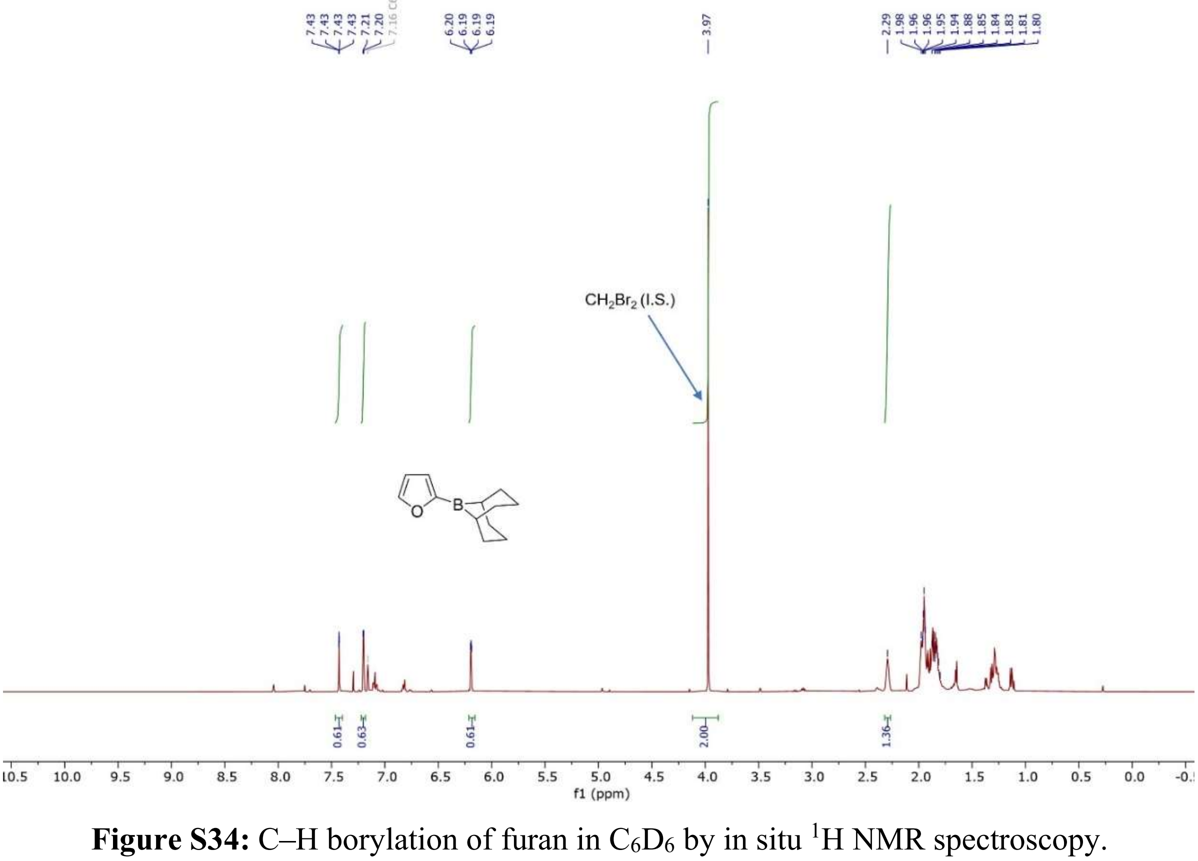

Figure S34: C–H borylation of furan in C_6D_6 by in situ ^1H NMR spectroscopy.

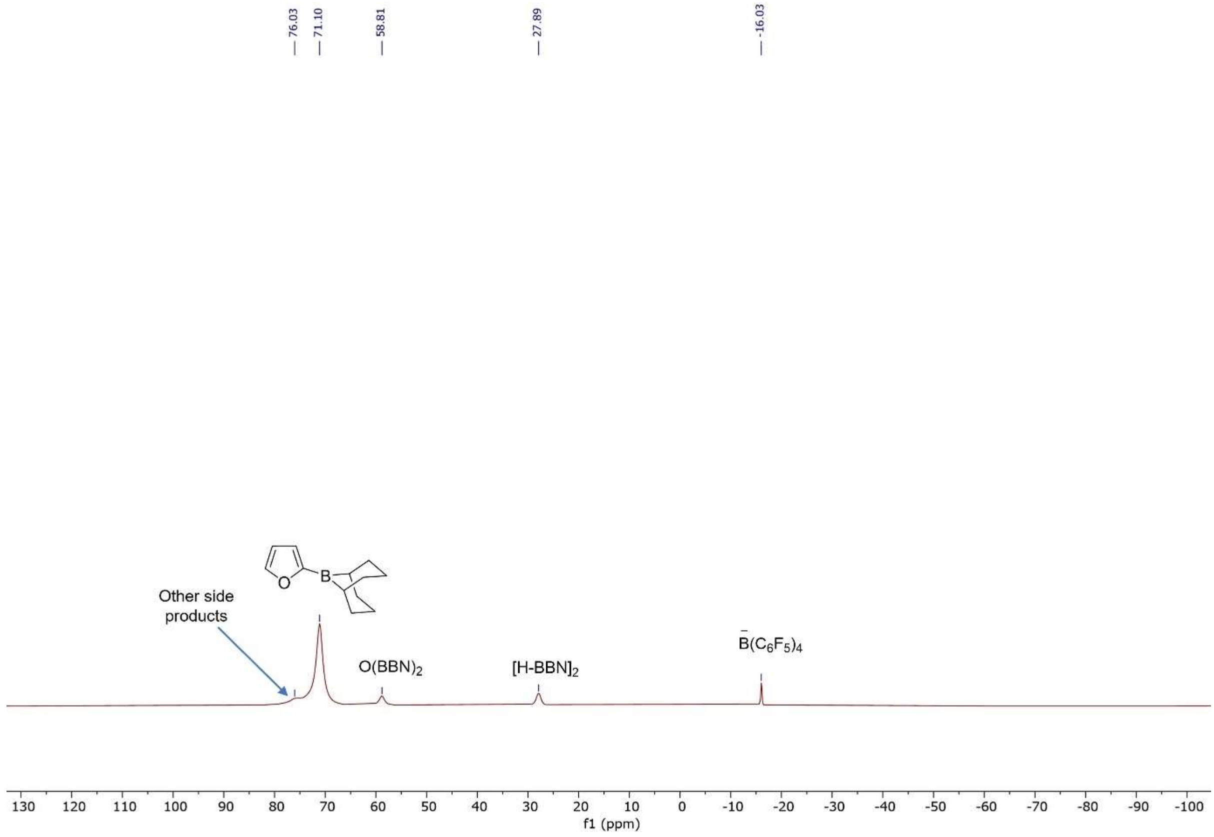

Figure S35: ^{11}B NMR spectroscopy from the crude reaction mixture in C_6D_6.

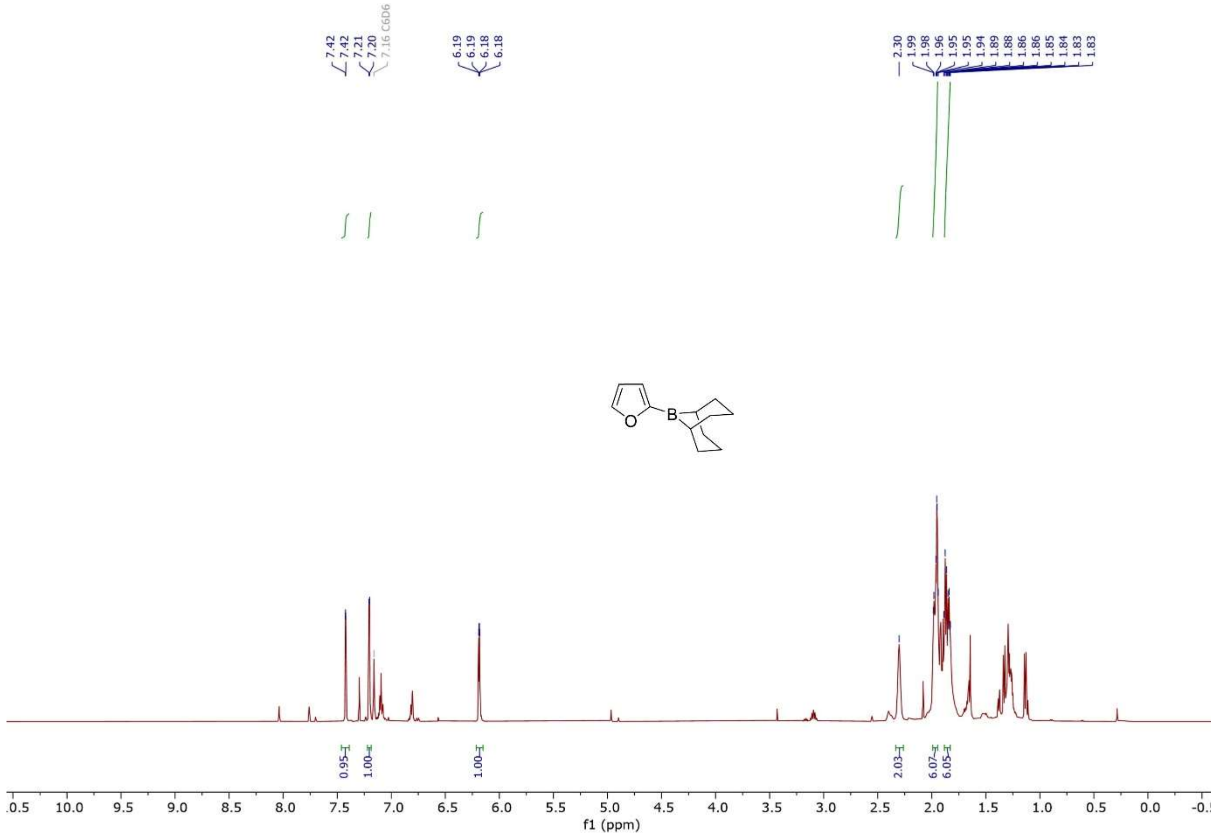

Figure S36: ^{1}H NMR spectrum of compound **3h** in C_6D_6.

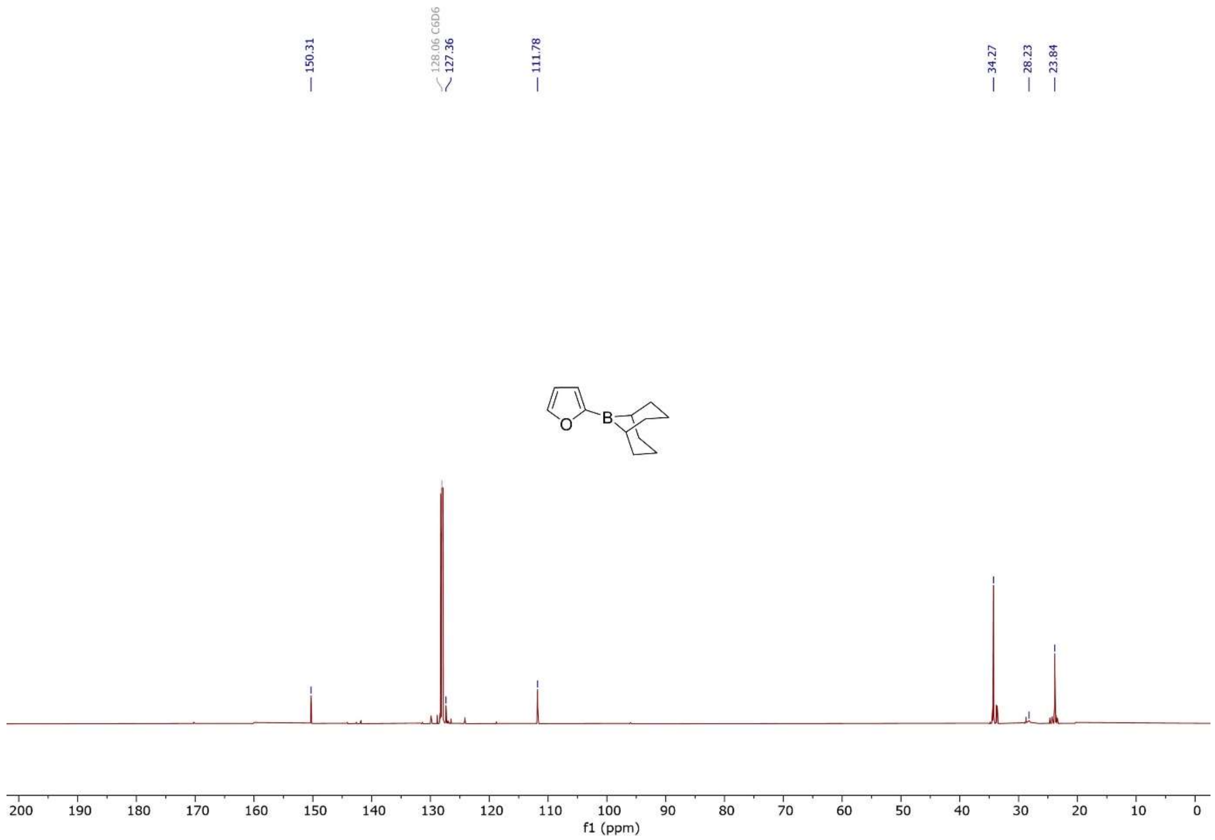

Figure S37: $^{13}C\{^1H\}$ NMR spectrum of compound **3h** in C_6D_6.

S3.2.9. Synthesis of 3-(9-borabicyclo[3.3.1]nonan-9-yl)-1-methyl-indole, **3i**

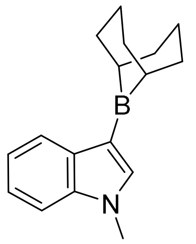

3i

As per general procedure 2, using 1-methylindole (36.0 µL, 0.287 mmol, 1.15 equiv.) and heating at 80 °C for 24 h. In situ yield by integration of diagnostic ^{1}H resonances (50% yield by ^{1}H-NMR spectroscopy).

^{1}H NMR (500 MHz, CD_2Cl_2): δ 8.10 (d, J = 7.9 Hz, 1H, ^{Indole}CH), 7.78 (s, 1H, ^{Indole}CH), 7.41 (d, J = 7.9 Hz, 1H, ^{Indole}CH), 7.31-7.28 (m, 1H, ^{Indole}CH), 7.24-7.20 (m, 1H, ^{Indole}CH), 3.85 (s, 3H, N-*Me*), 2.40 (br., 2H, BBN), 2.09-2.02 (m, 6H, BBN), 1.97-1.92 (m, 4H, BBN), 1.40-1.32 ppm (m, 2H, BBN).

$^{13}C\{^{1}H\}$ NMR (126 MHz, CD_2Cl_2): δ 141.8, 139.9, 133.6, 123.0, 122.3, 121.3, 110.1, 34.4, 33.7, 29.5 (br.), 24.1.

^{11}B NMR (160 MHz, CD_2Cl_2): δ 72.6.

Analytical data are consistent with that previously reported.[8]

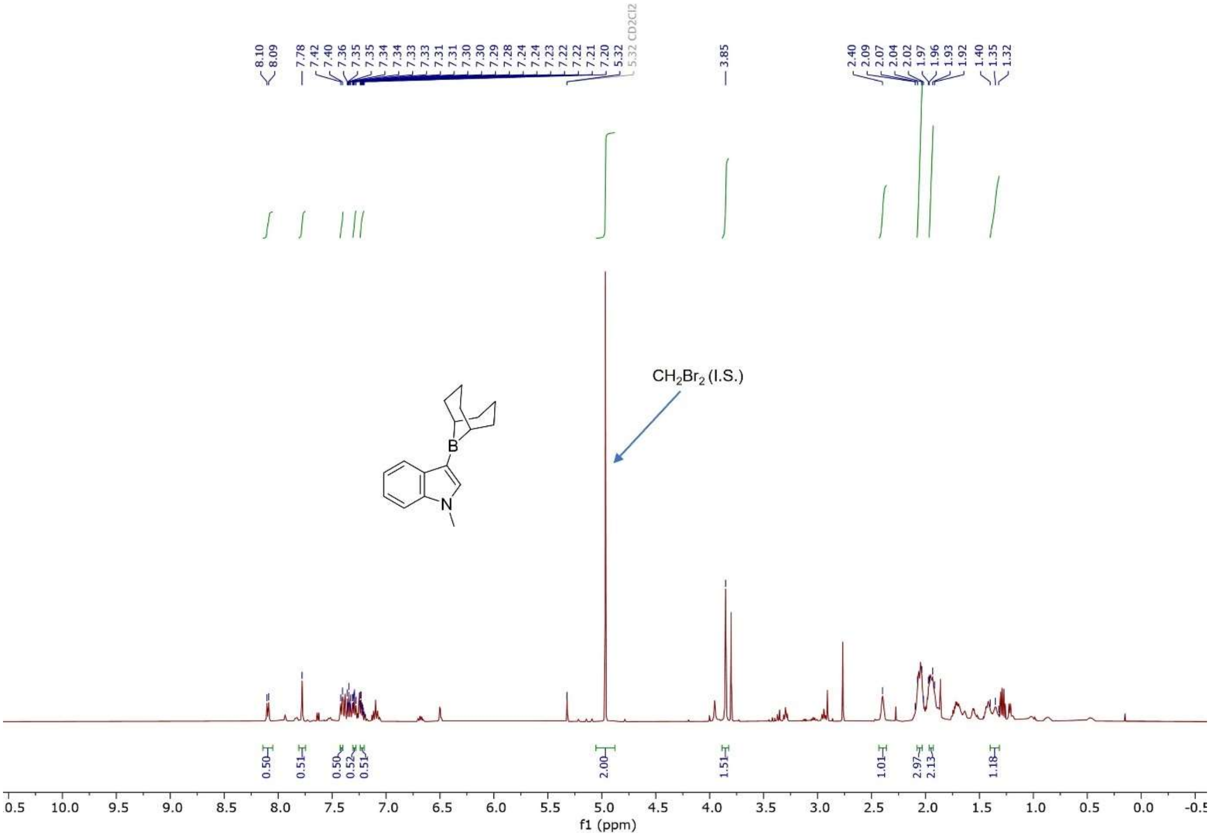

Figure S38: C–H borylation of 1-methylindole in CD_2Cl_2 for determination of NMR yield.

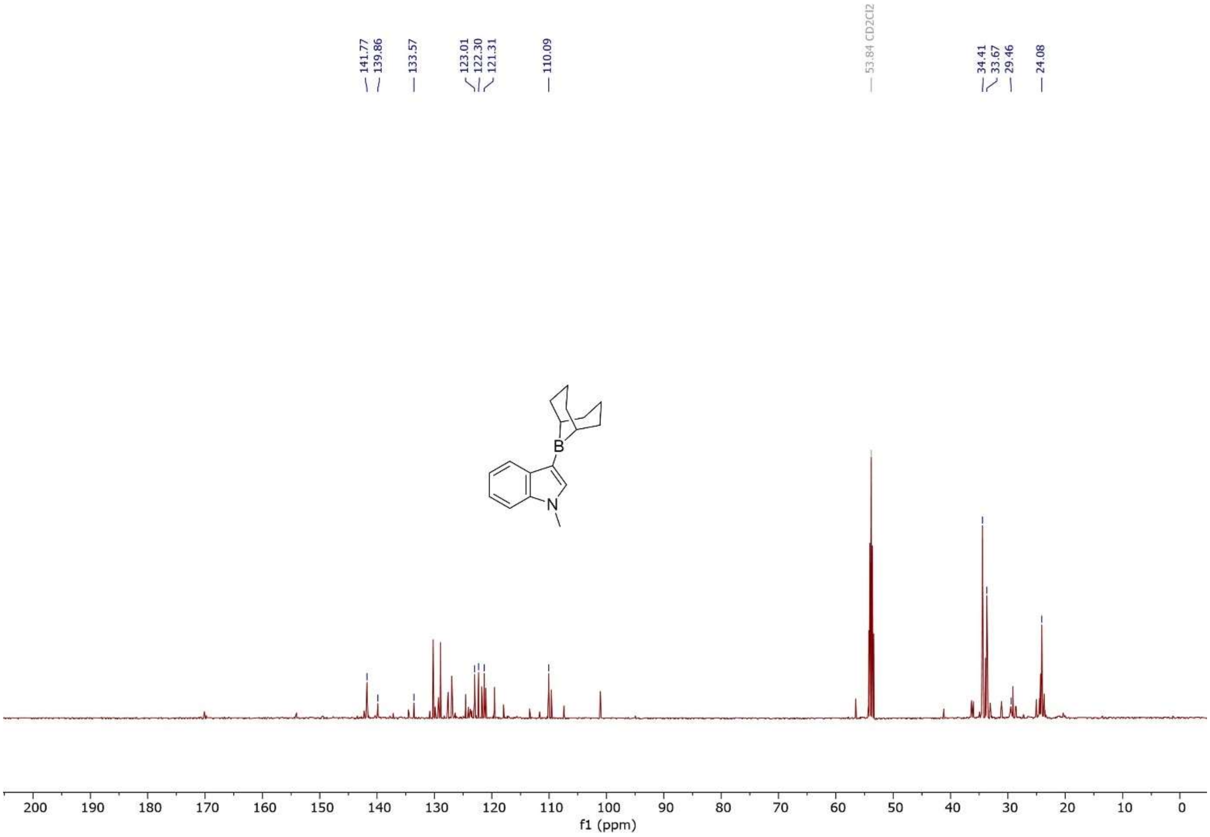

Figure S39: $^{13}C\{^{1}H\}$ NMR spectroscopy from the crude reaction mixture in CD_2Cl_2.

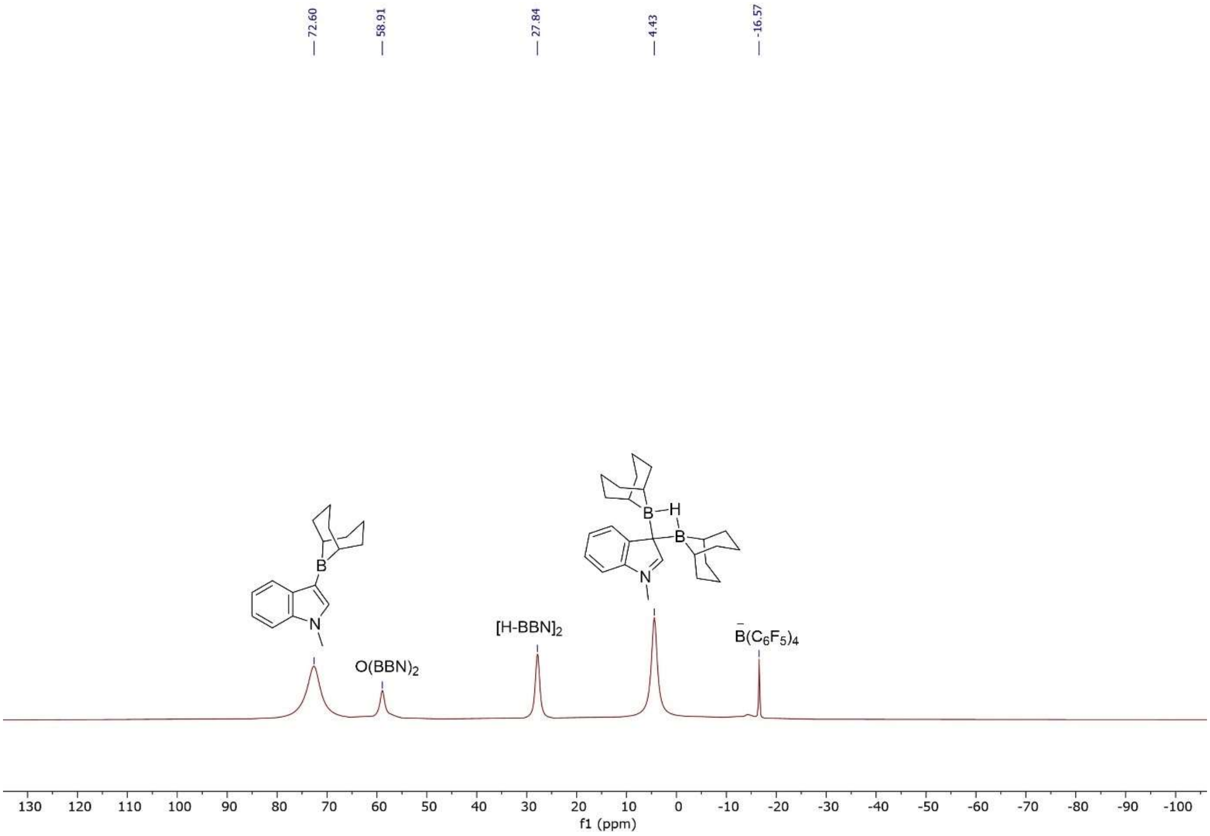

Figure S40: ^{11}B NMR spectroscopy from the crude reaction mixture in CD_2Cl_2.

S3.3. General procedure 3: Zinc catalysed C–H di-borylation of (hetero)arenes

In a glovebox, $[H{-}BBN]_2$ (64.0 mg, 0.262 mmol of dimer, 1.05 equiv.), ^{Dipp}NacNacZnH (6.0 mg, 0.0125 mmol) and $[(DMT)H][B(C_6F_5)_4]$ (10.5 mg, 0.0125 mmol) charged in a J. Young's NMR tube or in Schlenk ampule were dissolved in PhCl (0.6 mL). Subsequently, the corresponding heteroarene (0.250 mmol, 1.0 equiv.) was added to the reaction mixture and heated at 80-100 °C for a specified time. Upon completion, dibromomethane (17.5 µL, 0.250 mmol) was added to the reaction mixture as an internal standard to determine in situ yield by the integration of diagnostic ^{1}H (thienyl-BBN) resonances. In cases where the diagnostic peak in ^{1}H NMR spectrum is obscured by chlorobenzene solvent, the reaction mixture was dried and redissolved in C_6D_6 or $CDCl_3$ to determine in situ yield upon addition of dibromomethane (17.5 µL, 0.250 mmol) as an internal standard.

Di-borylated products **5c-5f** precipitated out from the reaction mixture, these were separated from the solution and washed with small amount (ca 1 ml) of chlorobenzene to isolate them as powders, sufficiently clean for unambiguous characterisation.

Please note, formation of the $O(BBN)_2$ was observed in minor amounts due to the moisture sensitivity of $[H{-}BBN]_2$ and borylated heteroarenes.

S3.3.1. Synthesis of 2,5-bis(9-borabicyclo[3.3.1]nonan-9-yl)-3-methyl-thiophene, **5a**

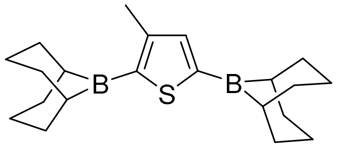

5a

As per general procedure 3, using 3-methylthiophene (24.5 µL, 0.25 mmol, 1.0 equiv.) and heating at 100 °C for 48 h. In situ yield by integration of diagnostic ^{1}H resonances (34% yield by ^{1}H NMR spectroscopy).

Analytical data for compound **5a** are mentioned earlier in the Section S3.2.5, page 18.

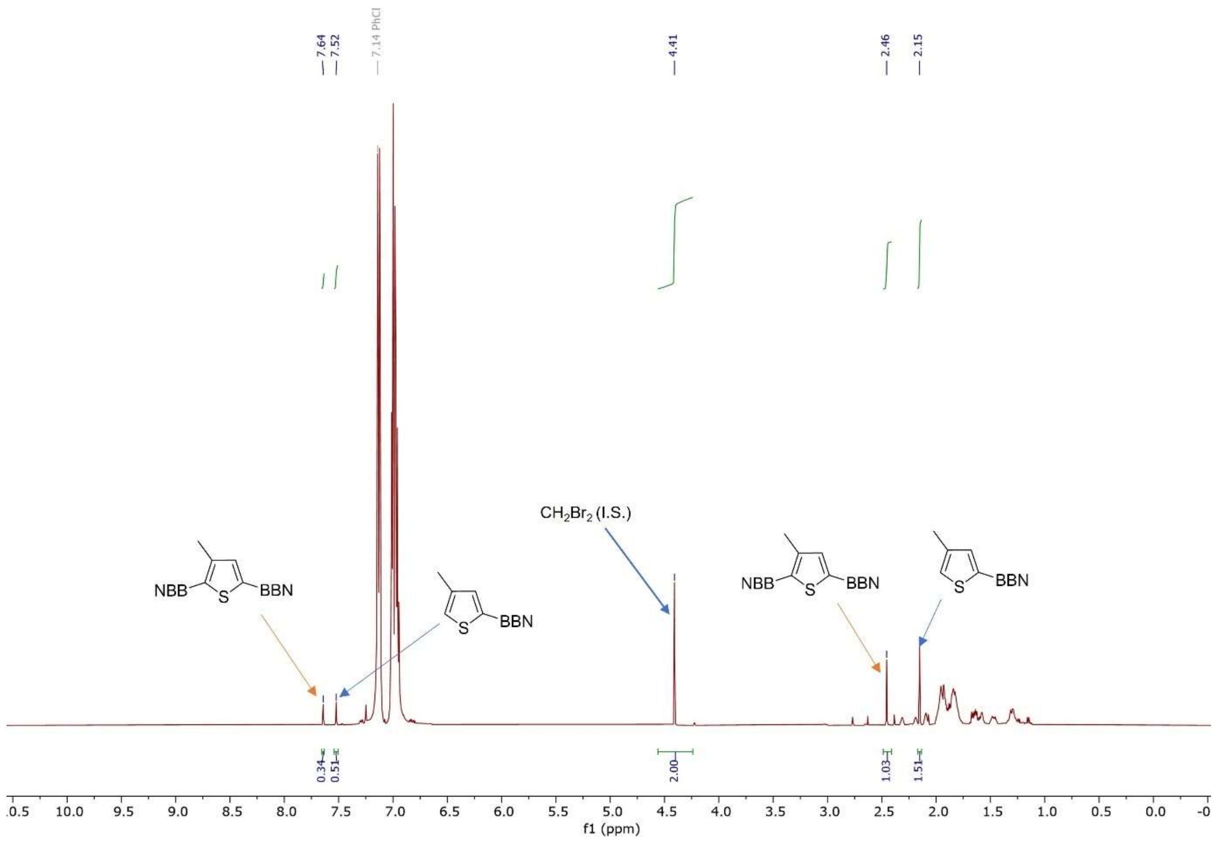

Figure S41: C–H di-borylation of 3-methylthiophene in PhCl for determination of NMR yield.

S3.3.2. Synthesis of 2,5-bis(9-borabicyclo[3.3.1]nonan-9-yl)-thiophene, **5b**

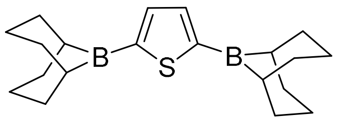

5b

As per general procedure 3, thiophene (20.0 μL, 0.25 mmol, 1.15 equiv.) and heating at 100 °C for 54 h. In situ yield by integration of diagnostic ^{1}H resonances (65% yield by ^{1}H NMR spectroscopy).

^{1}H NMR (500 MHz, C_6D_6): *δ* 7.90 (s, 2H, ^{Thienyl}C*H*), 2.31 (m, 4H, BBN), 2.02-1.96 (m, 8H, BBN), 1.95-1.85 (m, 12H, BBN), 1.32-1.27 (m, 4H, BBN).

$^{13}C\{^1H\}$ NMR (126 MHz, C_6D_6): *δ* 139.5, 34.6, 30.9, 23.9.

^{11}B NMR (160 MHz, C_6D_6): *δ* 75.4.

Note, several attempts were made to perform mass spectrometry on these compound, but these all did not show the $[M]^+$ or $[M+H]^+$.

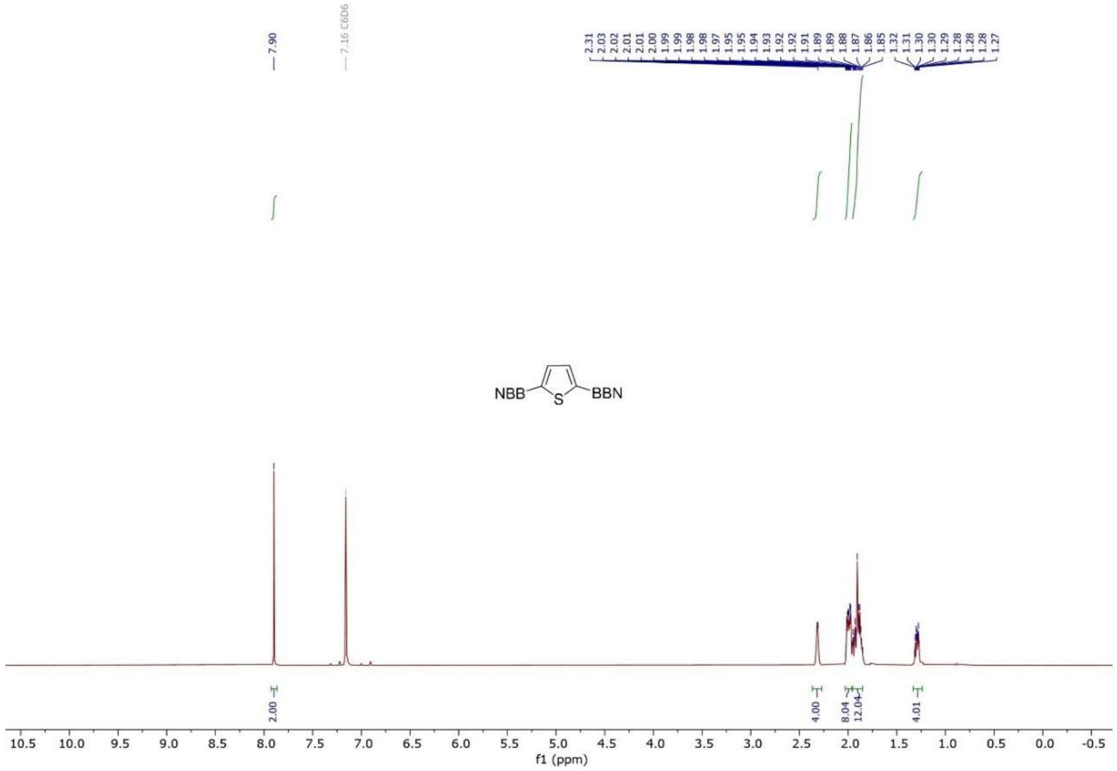

Figure S42: ^{1}H NMR spectrum of compound **5b** in C_6D_6.

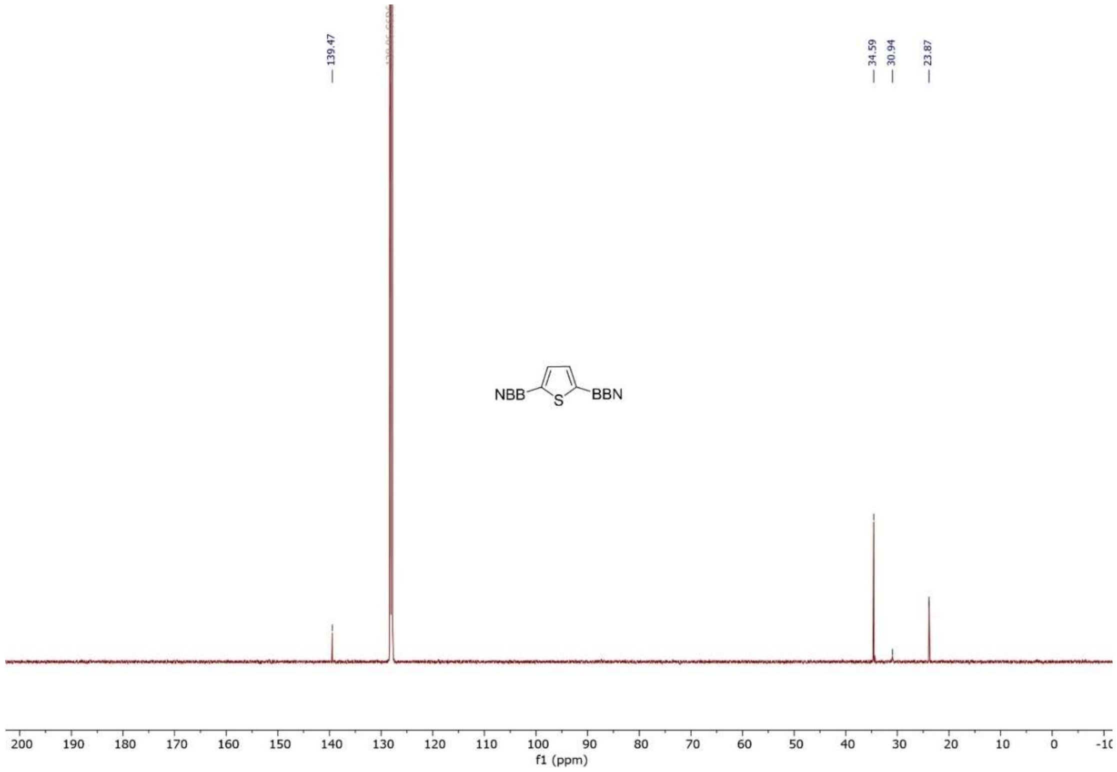

Figure S43: $^{13}C\{^{1}H\}$ NMR spectrum of compound **5b** in C_6D_6.

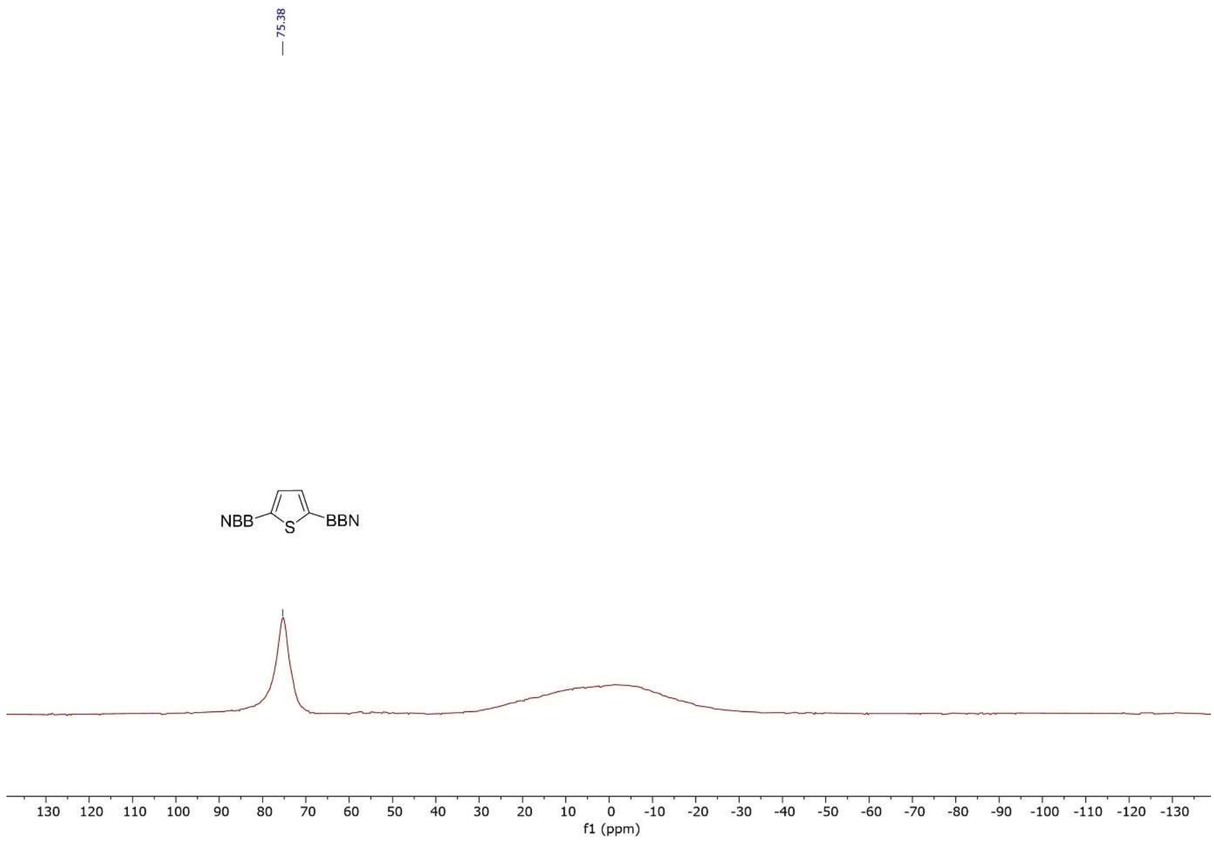

Figure S44: ^{11}B NMR spectrum of compound **5b** in C_6D_6.

S3.3.3. Synthesis of 2,5-bis(9-borabicyclo[3.3.1]nonan-9-yl)thieno-[3,2-b]thiophene, **5c**

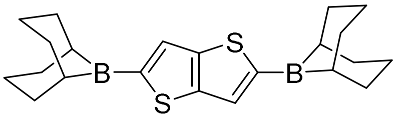

5c

As per general procedure 3, using thieno[3,2-b]thiophene (35.1 mg, 0.25 mmol, 1.0 equiv.) and heating at 80 °C for 48 h. Isolated yield: 61% (58 mg).

^{1}H NMR (500 MHz, $CDCl_3$): δ 8.04 (s, 2H, $^{\mathrm{Thienyl}}C\mathit{H}$), 2.18-2.17 (br., 4H, BBN), 2.06-1.84 (m, 20H, BBN), 1.41-1.33 (m, 4H, BBN).

^{13}C{^{1}H} NMR (126 MHz, $CDCl_3$): δ 150.4, 129.9, 34.4, 23.6.

^{11}B NMR (160 MHz, $CDCl_3$): δ 75.5.

Mass spectrometry: Calculated for $[C_{22}H_{30}B_2S_2]^+$: 380.19976, found 380.19703.

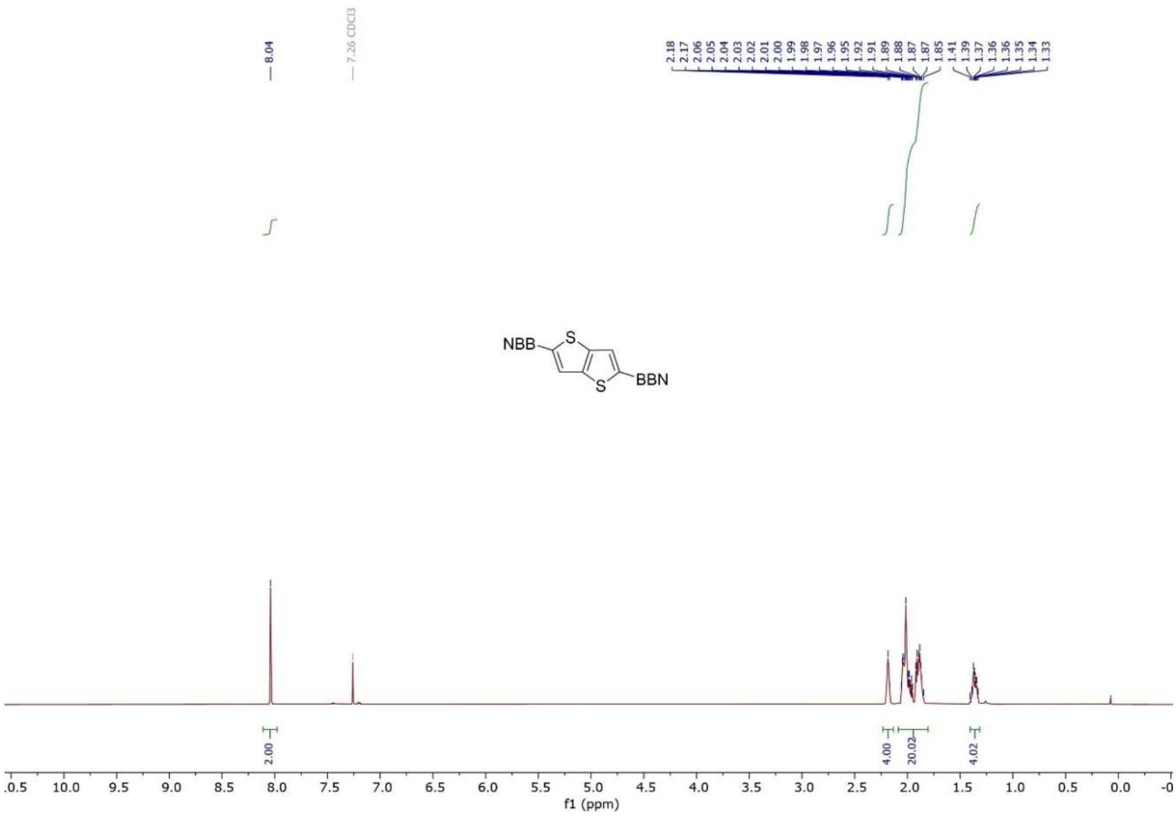

Figure S45: ^{1}H NMR spectrum of compound **5c** in $CDCl_3$.

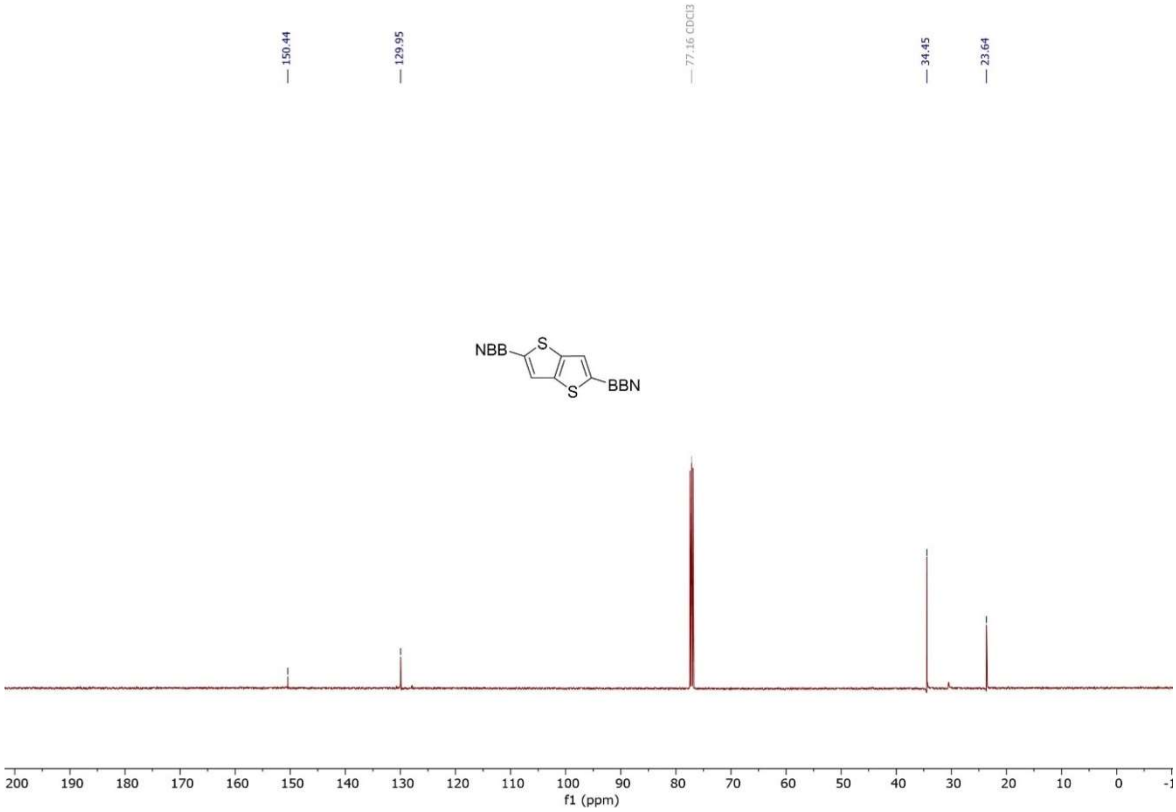

Figure S46: $^{13}C\{^{1}H\}$ NMR spectrum of compound **5c** in $CDCl_3$.

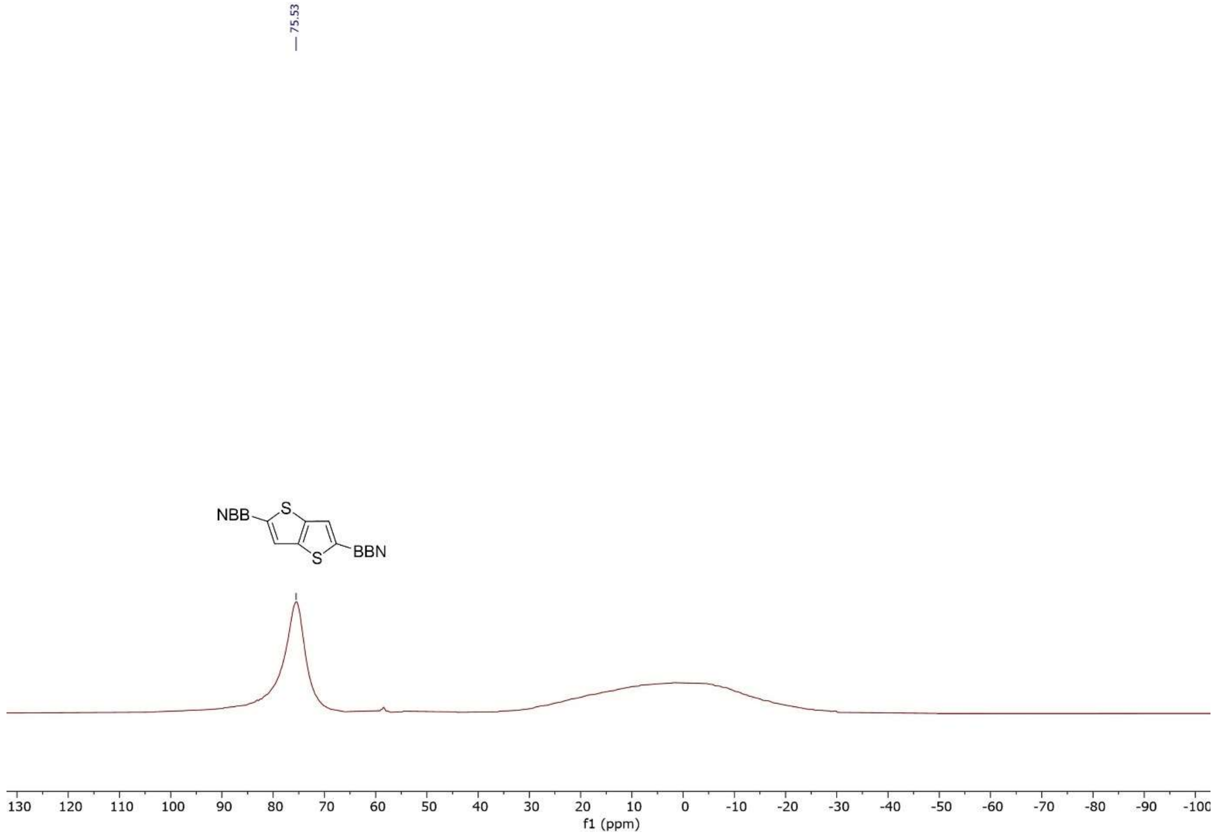

Figure S47: ^{11}B NMR spectrum of compound **5c** in $CDCl_3$.

S3.3.4. Synthesis of 5,5′-bis(9-borabicyclo[3.3.1]nonan-9-yl)-2,2′-bithiophene, **5d**

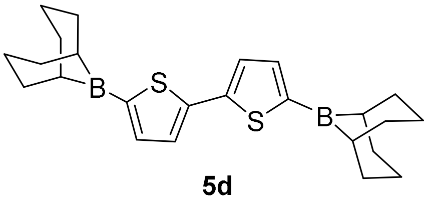

5d

As per general procedure 3, using 2,2′-bihiophene (41.6 mg, 0.25 mmol, 1.0 equiv.) and heating at 80 °C for 48 h. Isolated yield: 79% (80 mg).

^{1}H NMR (500 MHz, $CDCl_3$): δ 7.77 (d, J = 3.6 Hz, 2H, ^{Thienyl}CH), 7.51 (d, J = 3.6 Hz, 2H, ^{Thienyl}CH), 2.13-2.11 (br., 4H, BBN), 2.03-1.94 (m, 12H, BBN), 1.89-1.83 (m, 8H, BBN), 1.38-1.32 (m, 4H, BBN).

^{13}C{^{1}H} NMR (126 MHz, $CDCl_3$): δ 148.0, 139.1, 127.2, 34.4, 23.6.

^{11}B NMR (160 MHz, $CDCl_3$): δ 73.9.

Mass spectrometry: Calculated for $[C_{24}H_{32}B_2S_2]^+$: 406.21344, found 406.21259.

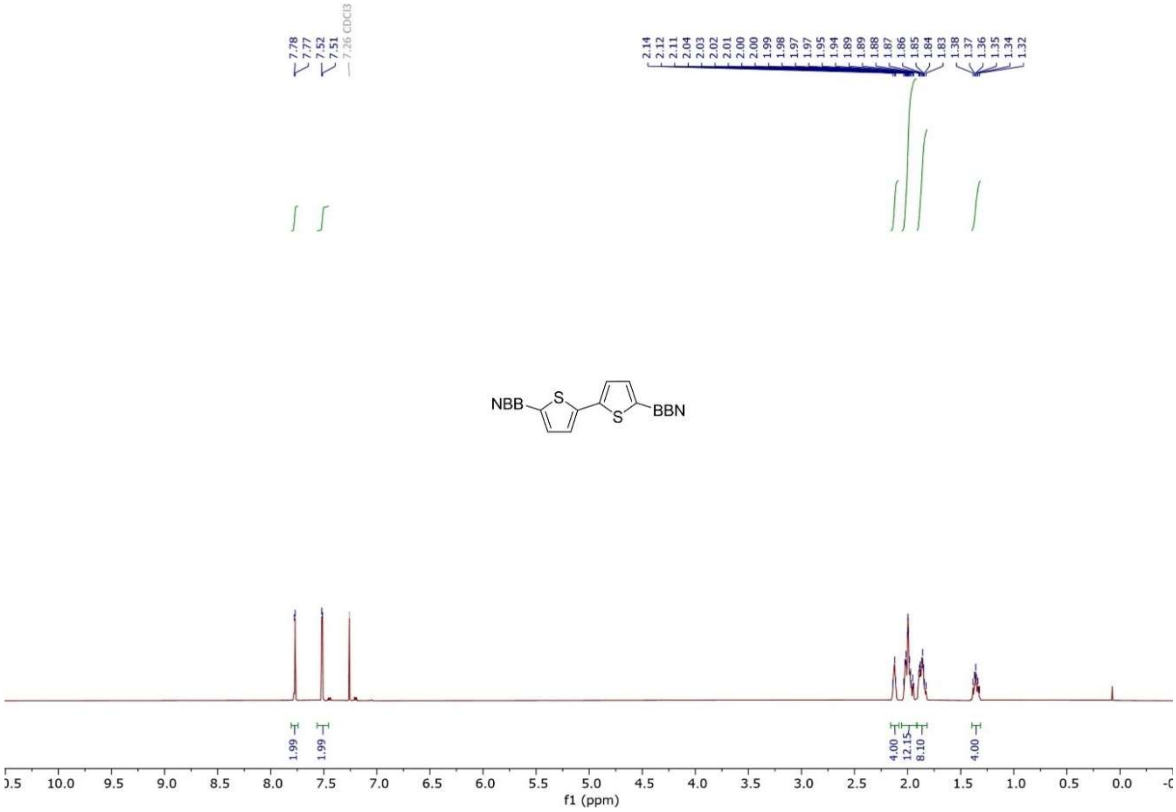

Figure S48: ^{1}H NMR spectrum of compound **5d** in $CDCl_3$.

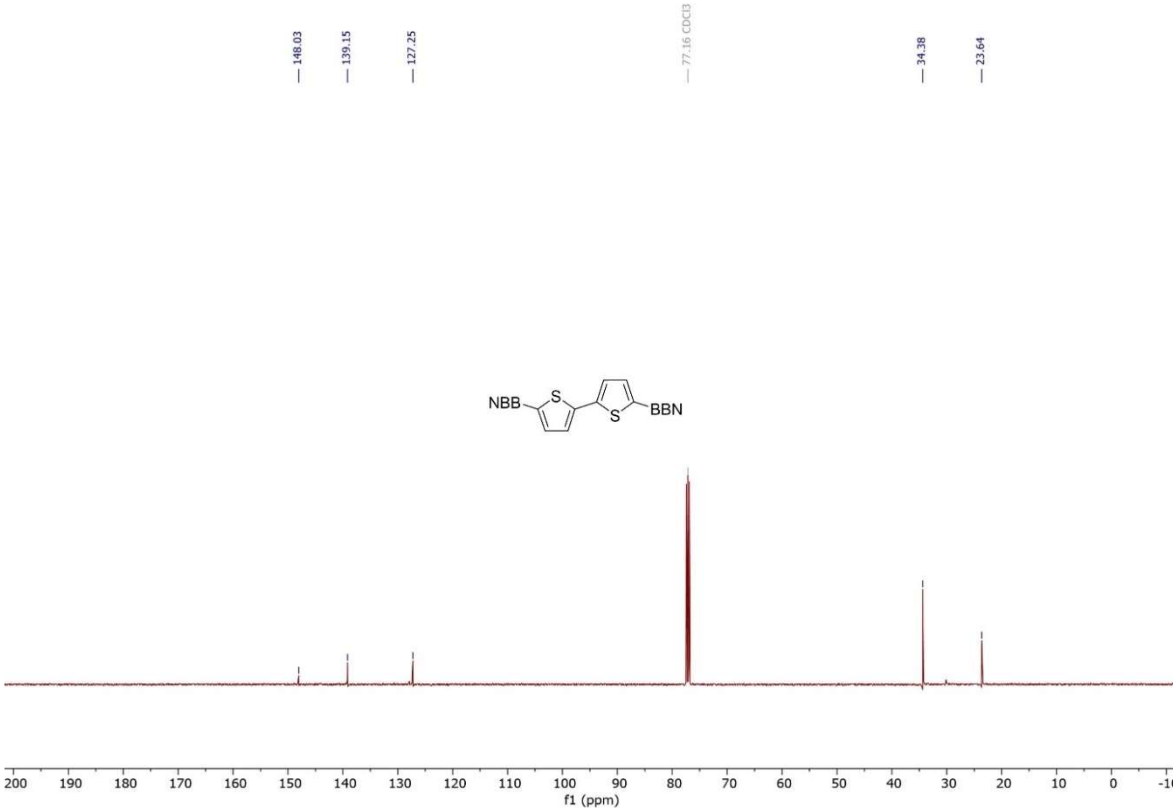

Figure S49: $^{13}C\{^{1}H\}$ NMR spectrum of compound **5d** in $CDCl_3$.

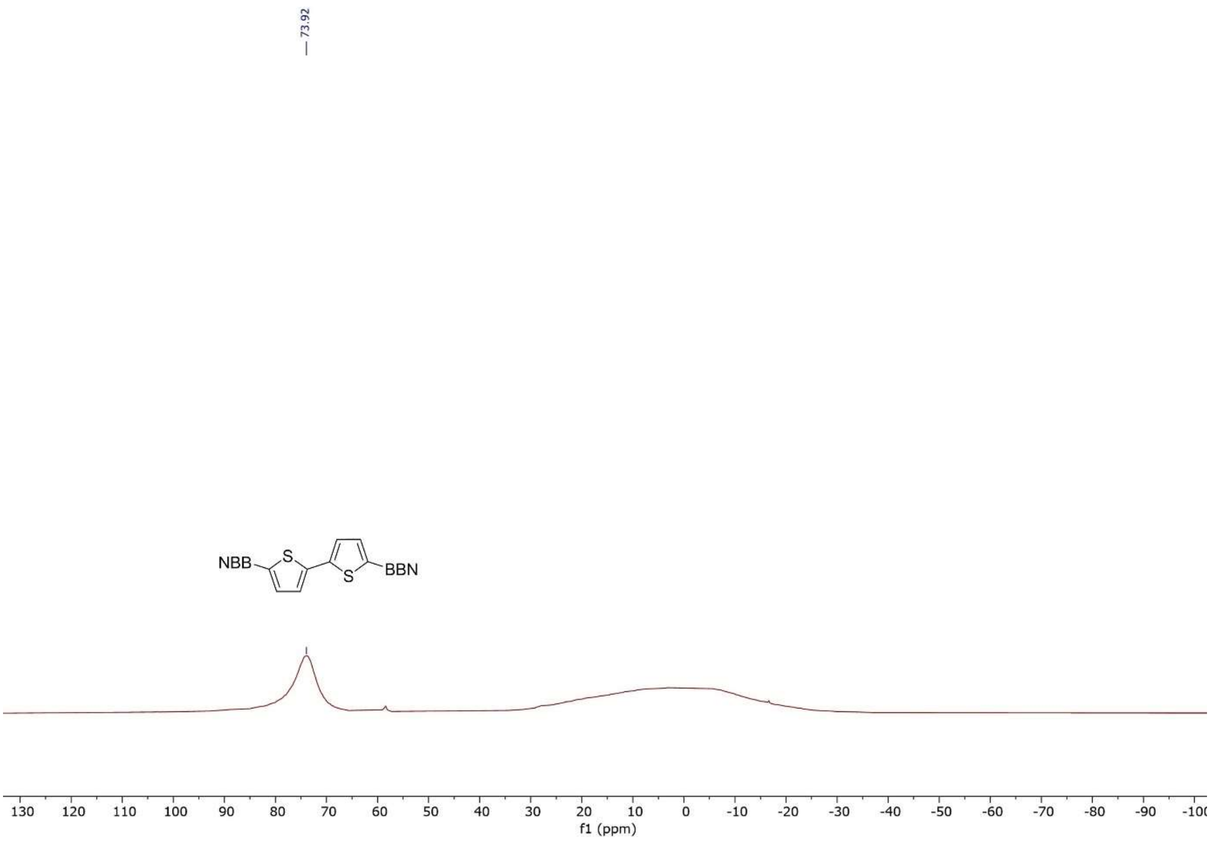

Figure S50: ^{11}B NMR spectrum of compound **5d** in $CDCl_3$.

S3.3.5. Synthesis of 5,5″-bis(9-borabicyclo[3.3.1]nonan-9-yl)-2,2′:5′,2″-terthiophene, **5e**

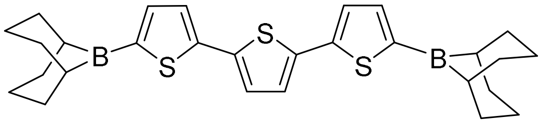

5e

As per general procedure 3, using 2,2′:5′,2″-Terthiophene (62.1 mg, 0.25 mmol, 1.0 equiv.) and heating at 80 °C for 48 h. Isolated yield: 62% (76 mg).

^{1}H NMR (500 MHz, $CDCl_3$): *δ* 7.78 (d, *J* = 3.6 Hz, 2H, ^{Thienyl}C*H*), 7.43 (d, *J* = 3.6 Hz, 2H, ^{Thienyl}C*H*), 7.29 (s, 2H, ^{Thienyl}C*H*), 2.15-2.13 (br., 4H, BBN), 2.06-1.97 (m, 12H, BBN), 1.92-1.85 (m, 8H, BBN), 1.41-1.36 (m, 4H, BBN).

^{13}C{^{1}H} NMR (126 MHz, $CDCl_3$): *δ* 147.7, 139.2, 137.6, 126.3, 125.9, 34.4, 23.6.

^{11}B NMR (160 MHz, $CDCl_3$): *δ* 73.7.

Mass spectrometry: Calculated for $[C_{28}H_{34}B_2S_2]^+$: 488.20133, found 488.20060.

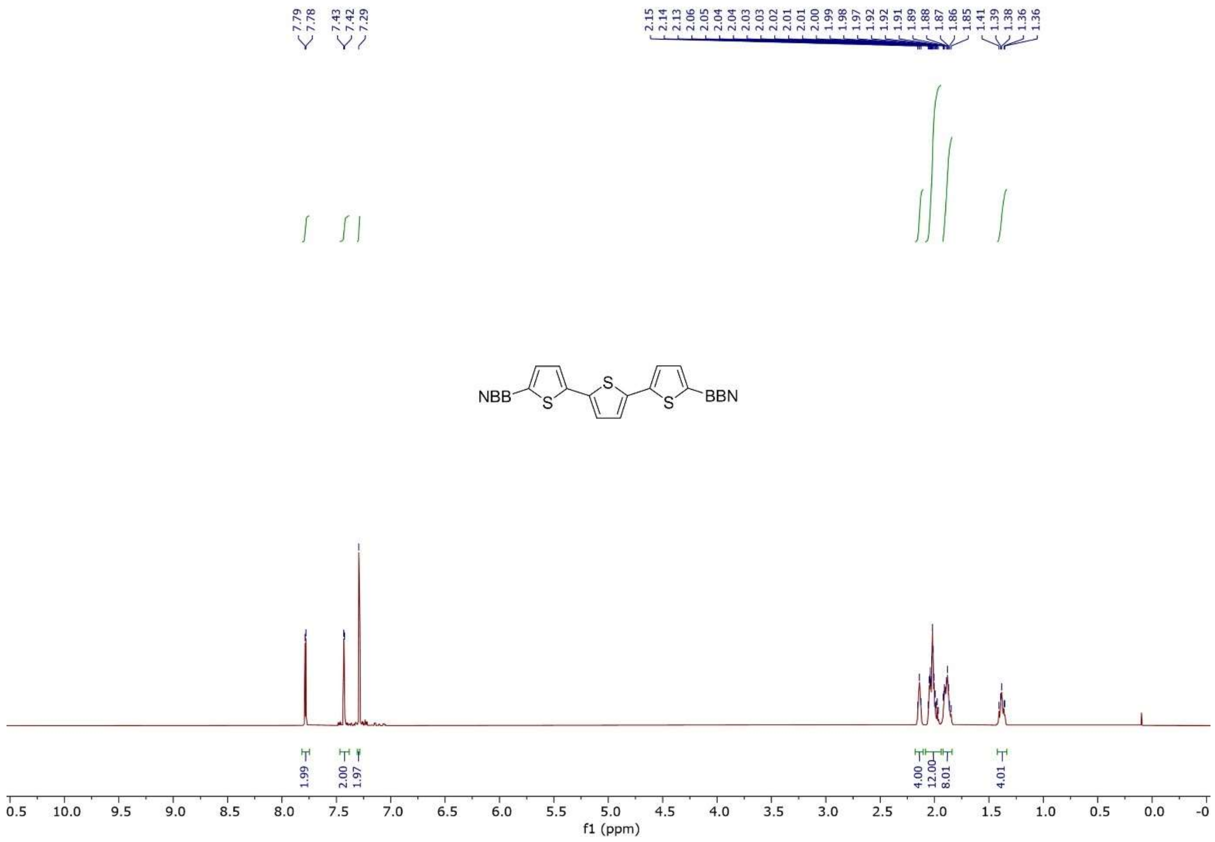

Figure S51: ^{1}H NMR spectrum of compound **5e** in $CDCl_3$.

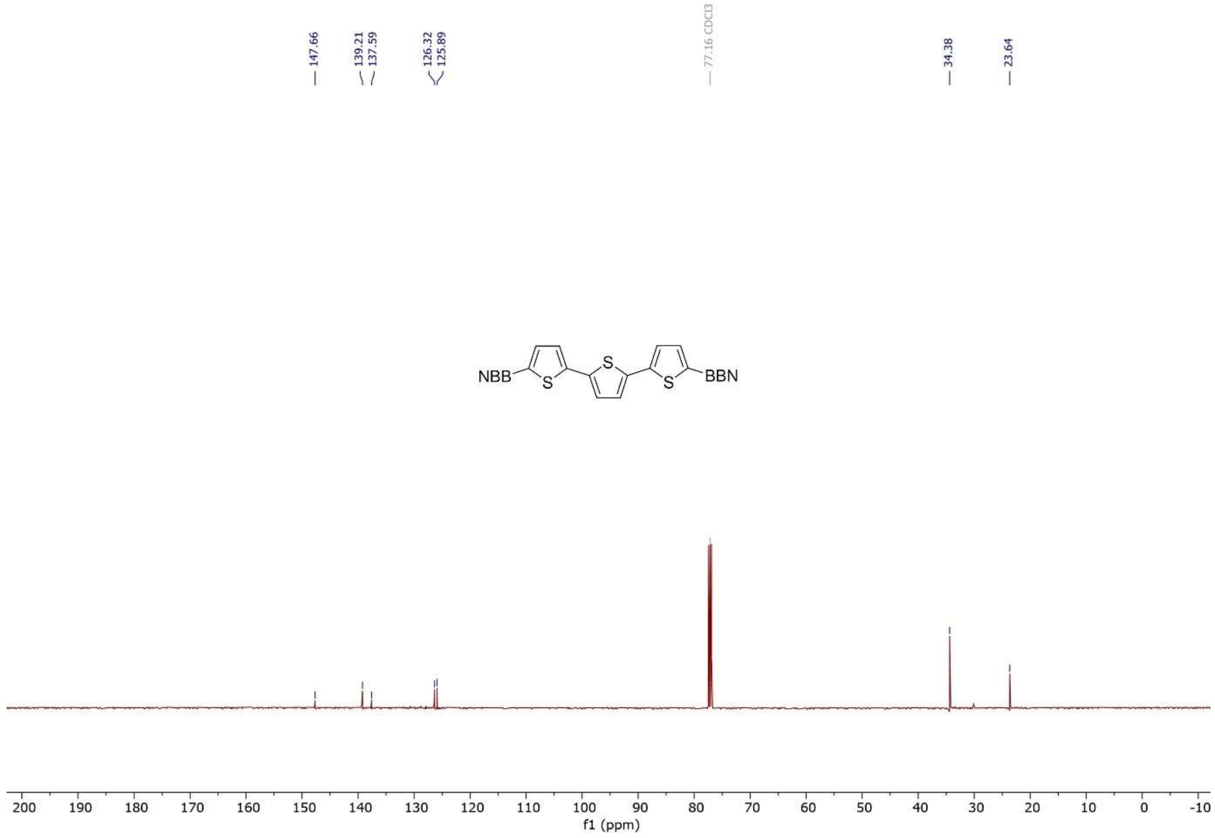

Figure S52: $^{13}C\{^1H\}$ NMR spectrum of compound **5e** in $CDCl_3$.

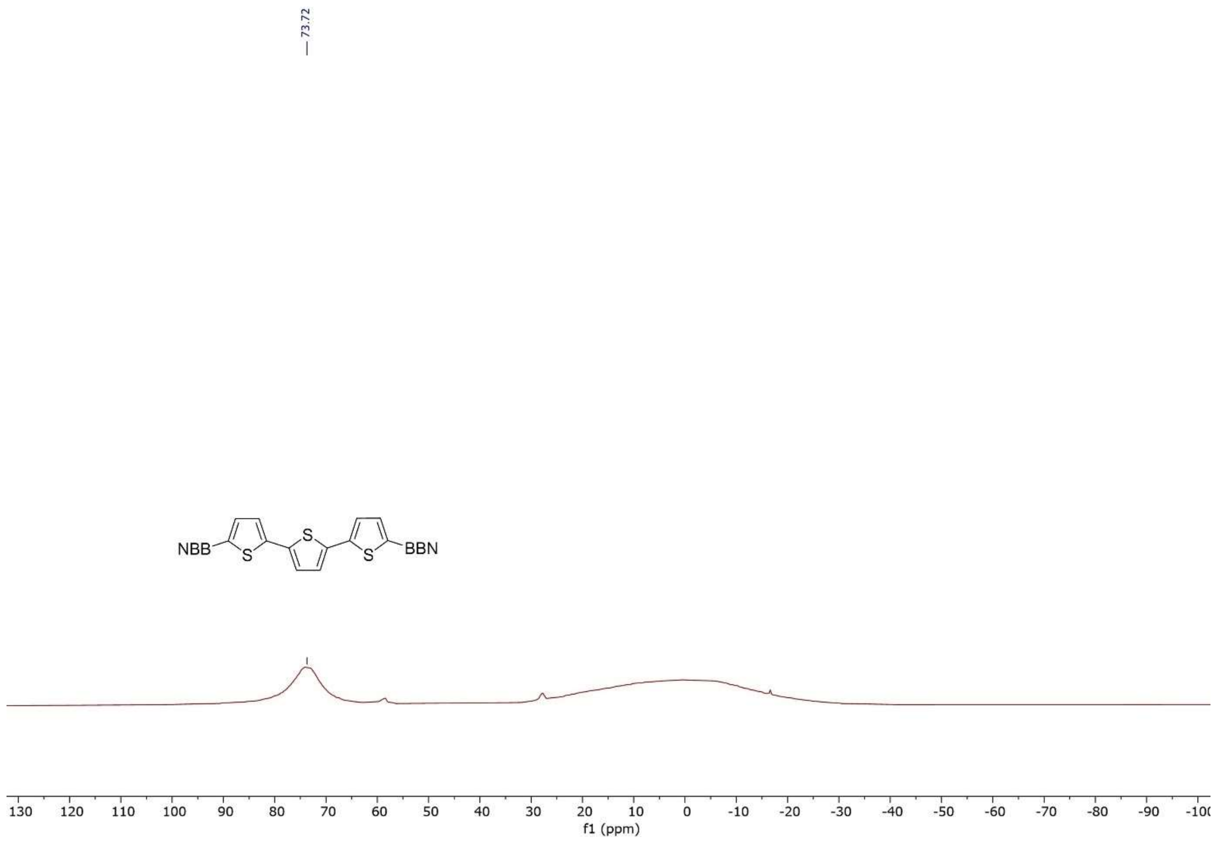

Figure S53: ^{11}B NMR spectrum of compound **5e** in $CDCl_3$.

S3.3.6. Synthesis of 2,2'-bis(9-borabicyclo[3.3.1]nonan-9-yl)-4H-Cyclopenta[1,2-b:5,4-b']dithiophene, **5f**

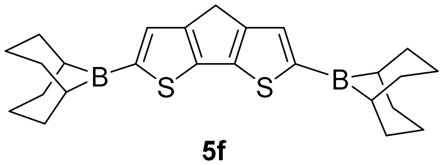

5f

As per general procedure 3, using 4H-Cyclopenta[1,2-b:5,4-b']dithiophene (44.6 mg, 0.25 mmol, 1.0 equiv.) and heating at 80 °C for 48 h. Isolated yield: 55% (57.5 mg).

^{1}H NMR (500 MHz, $CDCl_3$): δ 7.82 (s, 2H, $^{Thienyl}CH$), 3.68 (s, 2H, CH_2), 2.15-2.13 (br., 4H, BBN), 2.04-1.84 (m, 20H, BBN), 1.40-1.34 (m, 4H, BBN).

$^{13}C\{^1H\}$ NMR (126 MHz, $CDCl_3$): δ 154.7, 150.3, 133.4, 34.4, 31.5, 23.7.

^{11}B NMR (160 MHz, $CDCl_3$): δ 73.4.

Note, several attempts were made to perform mass spectrometry on these compound, but these all did not show the $[M]^+$ or $[M+H]^+$.

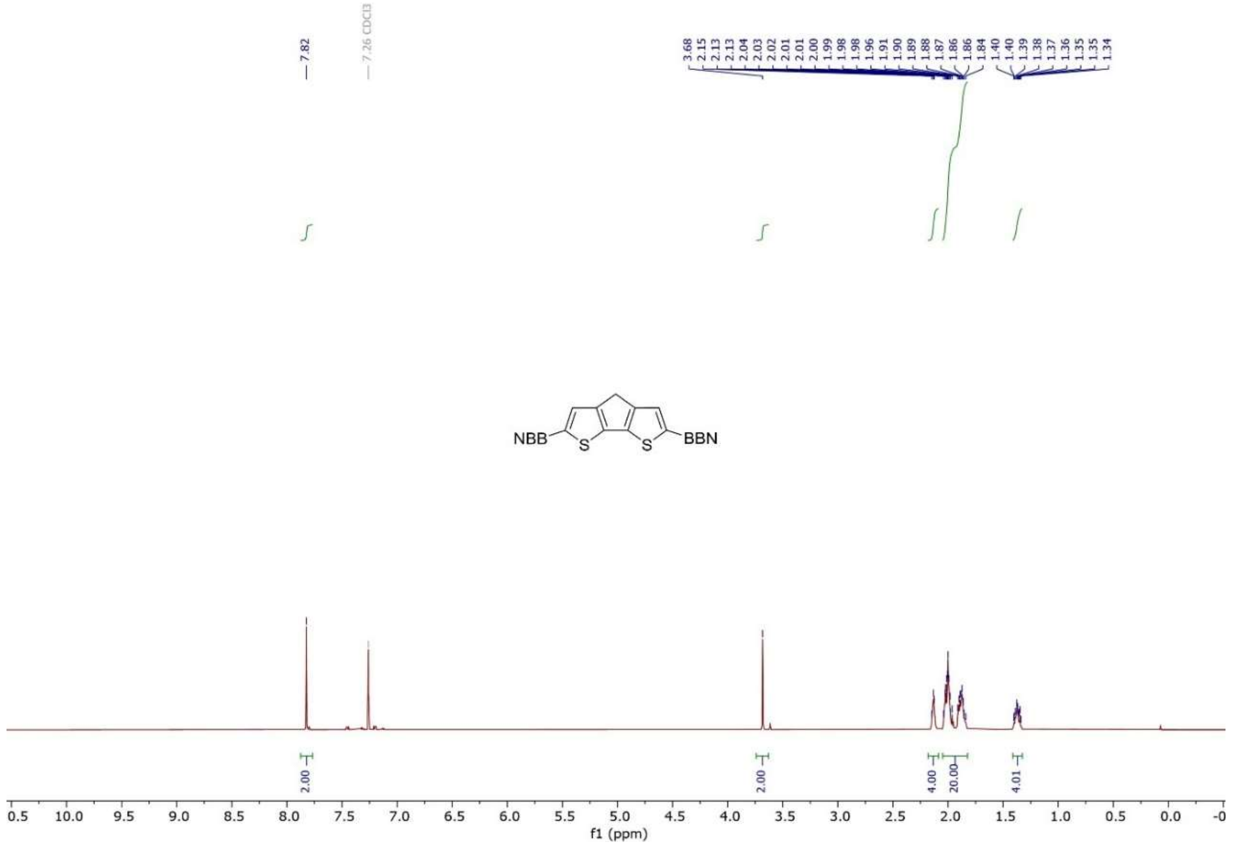

Figure S54: ^{1}H NMR spectrum of compound **5f** in $CDCl_3$.

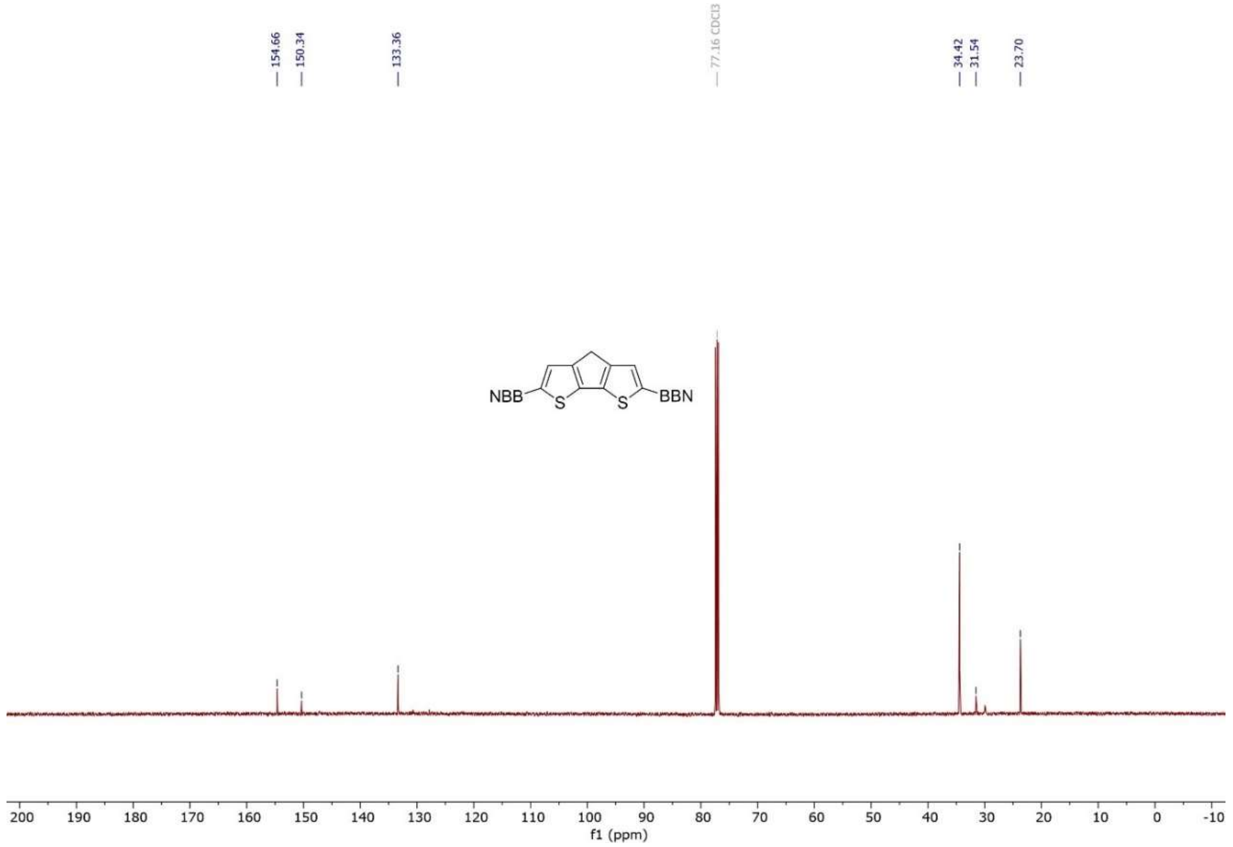

Figure S55: $^{13}C\{^{1}H\}$ NMR spectrum of compound **5f** in $CDCl_3$.

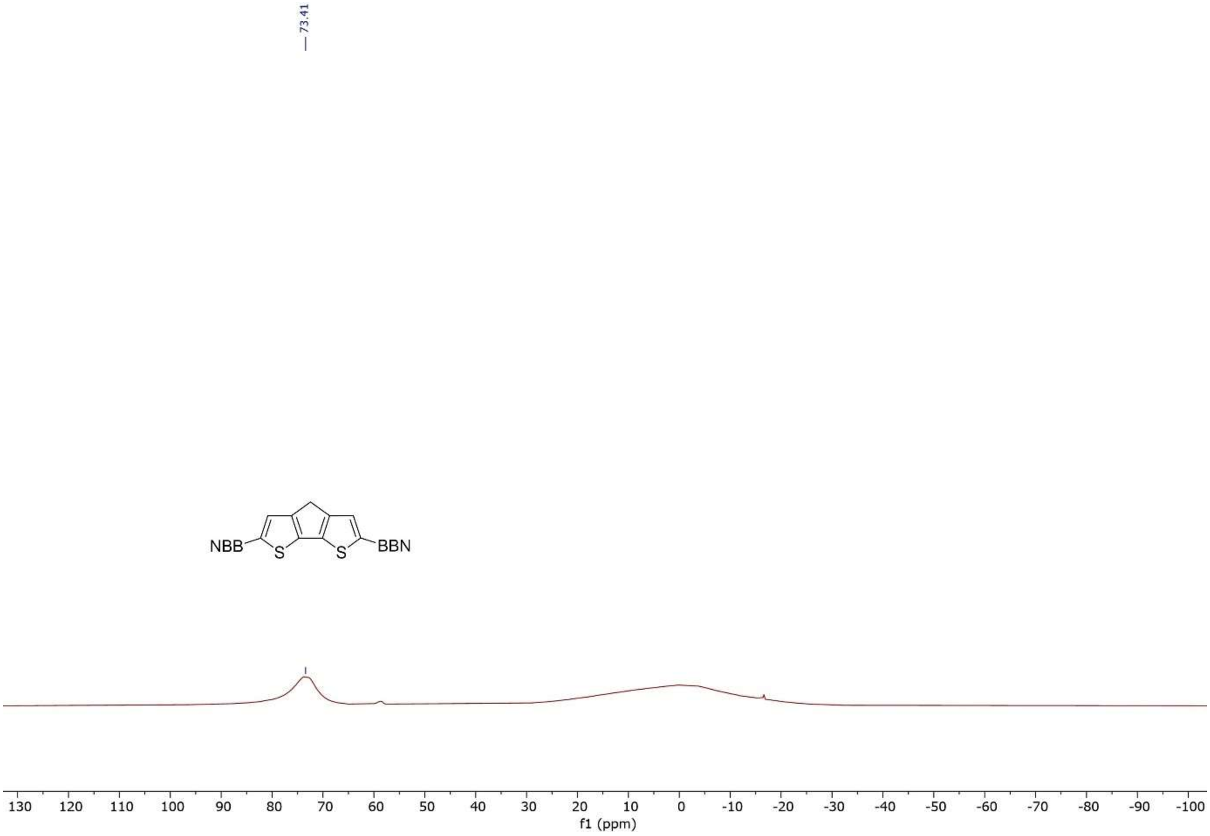

Figure S56: ^{11}B NMR spectrum of compound **5f** in $CDCl_3$.

S3.4. Unsuccessful substrates for zinc catalysed C−H borylation

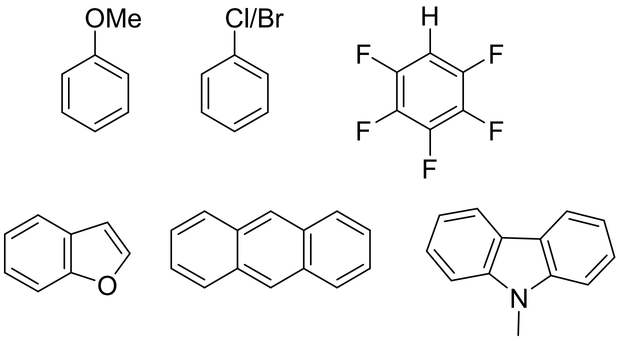

S3.5. General procedure 4: Optimisation for the aluminium catalysed C−H borylation of 2-methyl-thiophene

In a glovebox, $[H{-}BBN]_2$ (30.5 mg, 0.125 mmol of dimer), $^{Dipp}NacNacAlMe_2$ (0.0120-0.0250 mmol) and Brønsted acid (0.0125-0.0250 mmol) charged in a J. Young's NMR tube were dissolved in PhCl (0.5 mL). Subsequently, 2-methyl-thiophene (24.0 µL, 0.250 mmol, 1.0 equiv.) was added to the reaction mixture and heated at a specified temperature for a specified time. Upon completion, the in-situ conversion was determined by the integration of diagnostic ^1H (*Me*-thienyl-BBN in the product versus unreacted *Me*-thienyl in the starting material) resonances.

Table S2: Aluminium catalysed C−H borylation of 2-methyl-thiophene.[a]

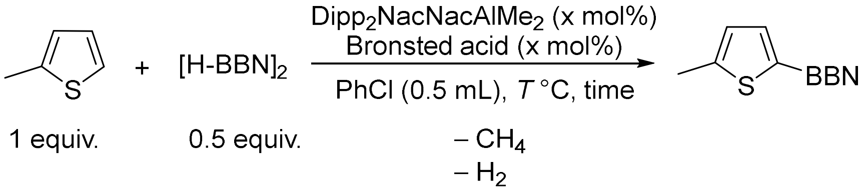

**Entry**	**[Al] (mol%)**	**Brønsted Acid**	**Acid (mol%)**	**T (°C)**	**Time (h)**	**Con. (%)[b]**
**1**	10	$[(DMT)H][B(C_6F_5)_4]$	10	rt	5	0.0
**2**	10	$[(DMT)H][B(C_6F_5)_4]$	10	80	20	65
**3**	10	$[(Et_3N)H][B(C_6F_5)_4]$	10	80	20	52
**4**	10	$[(DMT)H][B(C_6F_5)_4]$	10	100	20	94.0
**5**	5	$[(DMT)H][B(C_6F_5)_4]$	5	100	24	84.0
**6**	10	$[(DMT)H][B(C_6F_5)_4]$	10	100	8	91.0
**7[c]**	10	$[(DMT)H][B(C_6F_5)_4]$	10	100	8	89.0
**8[d]**	10	$[(DMT)H][B(C_6F_5)_4]$	10	100	8	90.0
**9**	10	$[(2,4\text{-}Br_2C_6H_3\text{-}NMe_2)H][B(C_6F_5)_4]$	10	100	8	71.0
**10**	10	$[(DET)H][B(C_6F_5)_4]$	10	100	8	94.0
**11**	-	$[(DMT)H][B(C_6F_5)_4]$	10	100	8	0.0
**12**	10	-	-	100	8	0.0

[a] 2-methyl-thiophene (1.0 equiv.), $[H{-}BBN]_2$ (0.5 equiv.), $^{Dipp}NacNacAlMe_2$ (0.05-0.1 equiv), and Brønsted acid (0.05-0.1 equiv) in PhCl (0.5 mL). [b] Conversion by ^1H NMR spectroscopy CH_3- of the product versus CH_3- of the substrate. [c] reaction carried out in $C_6D_5B_r$. [d] reaction carried out in 1,2-difluorobenzene.

S3.6. General procedure 5: Aluminium catalysed C–H mono-borylation of (hetero)arenes

In a glovebox, $[H-BBN]_2$ (30.5 mg, 0.125 mmol of dimer, 0.5 equiv.), $^{Dipp}NacNacAlMe_2$ (12 mg, 0.0250 mmol) and Brønsted acid (20.5 mg, 0.0250 mmol) charged in a J. Young's NMR tube were dissolved in PhCl (0.5 mL). Subsequently, the corresponding heteroarene (0.250 mmol, 1.0 equiv.) was added to the reaction mixture and heated at 100 °C for 8 h. Upon completion, dibromomethane (17.5 µL, 0.250 mmol) or trimethoxybenzene (12.5 µL, 0.025 mmol) was added to the reaction mixture as an internal standard to determine in situ yield by the integration of diagnostic ^1H resonances. In cases where the diagnostic peak in ^1H NMR spectrum are obscured by chlorobenzene solvent, the reaction mixture was dried and redissolved in CH_2Cl_2 to determine the in situ yield.

Please note, formation of the MeBBN was observed in minor amounts due to the metathesis reaction between $^{Dipp}NacNacAlMe_2$ and $[H-BBN]_2$.

S3.6.1. Synthesis of 2-(9-borabicyclo[3.3.1]nonan-9-yl)-5-methyl-thiophene, **3a**

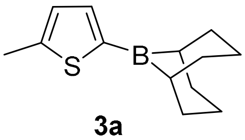

3a

As per general procedure 5, using 2-methyl-thiophene (24.0 µL, 0.250 mmol, 1.0 equiv.). In situ yield by integration of diagnostic ^1H resonances versus dibromomethane in chlorobenzene solvent (91% yield by ^1H NMR spectroscopy).

Analytical data for compound **3a** are mentioned earlier in the Section S3.2.1, page 7.

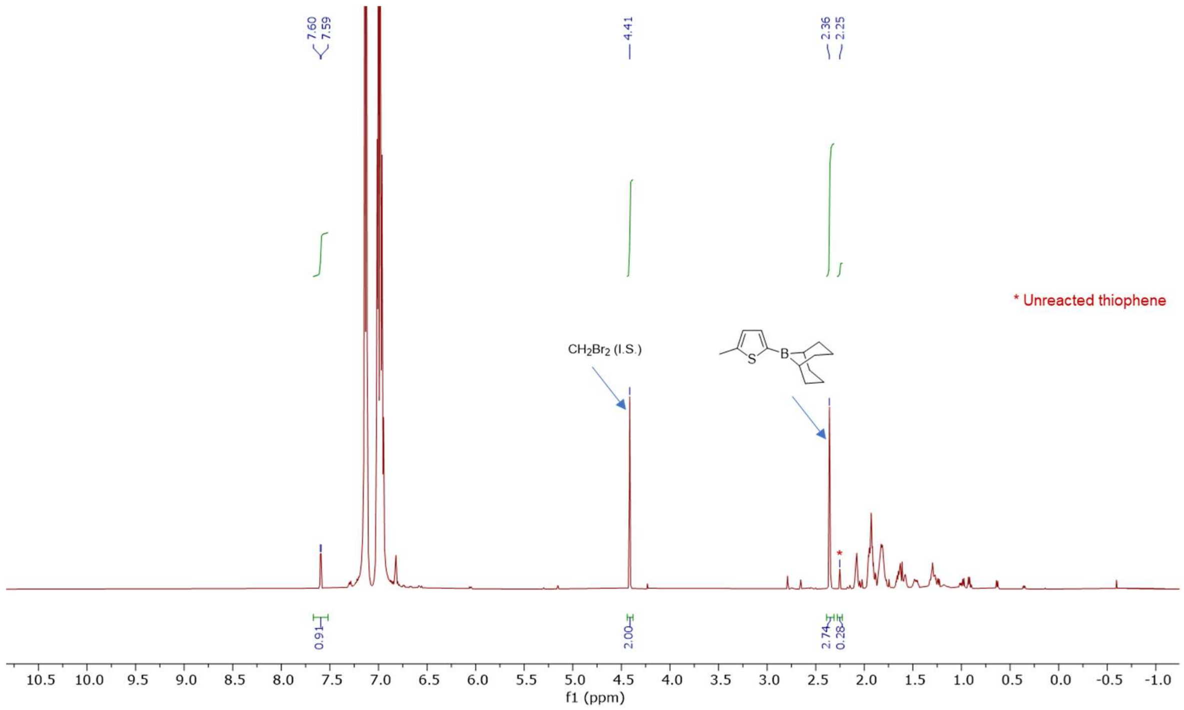

Figure S57: C–H borylation of 2-methylthiophene in PhCl for determination of NMR yield.

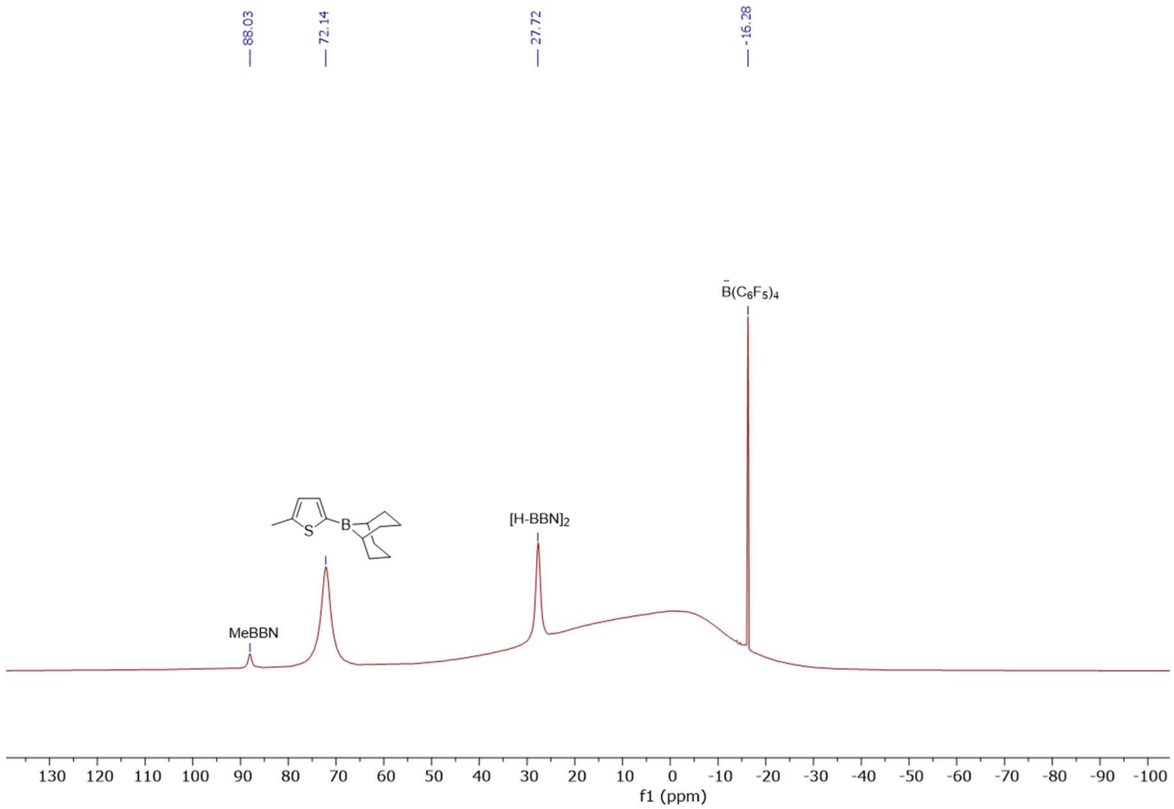

Figure S58: ^{11}B NMR spectrum of the crude reaction mixture in PhCl.

S3.6.2. Synthesis of 3-(9-borabicyclo[3.3.1]nonan-9-yl)-1-methyl-indole, **3i**

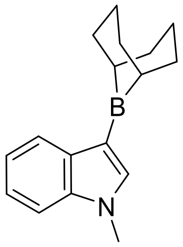

3i

As per general procedure 5, using 1-methylindole (31.0 µL, 0.250 mmol, 1.0 equiv.). In situ yield by integration of diagnostic ^{1}H resonances versus trimethoxybenzene in CH_2Cl_2 (62% yield by ^{1}H NMR spectroscopy). Please note formation of 1-methylindoline was also observed albeit in low yield (14% yield by ^{1}H NMR spectroscopy).[9]

Analytical data for compound **3i** are mentioned earlier in the Section S3.2.9, page 29-30.[8]

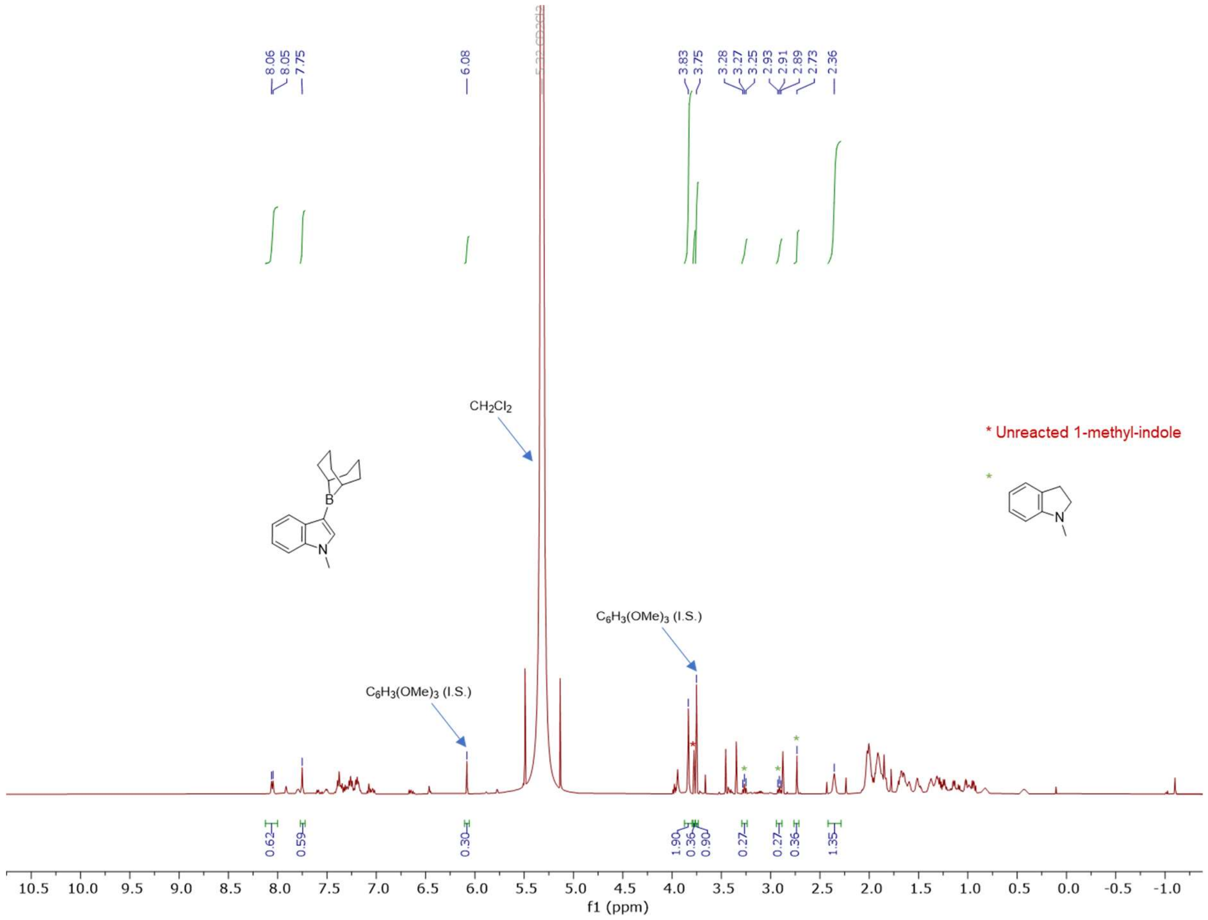

Figure S59: C–H borylation of 1-methylindole in CH_2Cl_2 by in situ ^{1}H NMR spectroscopy.

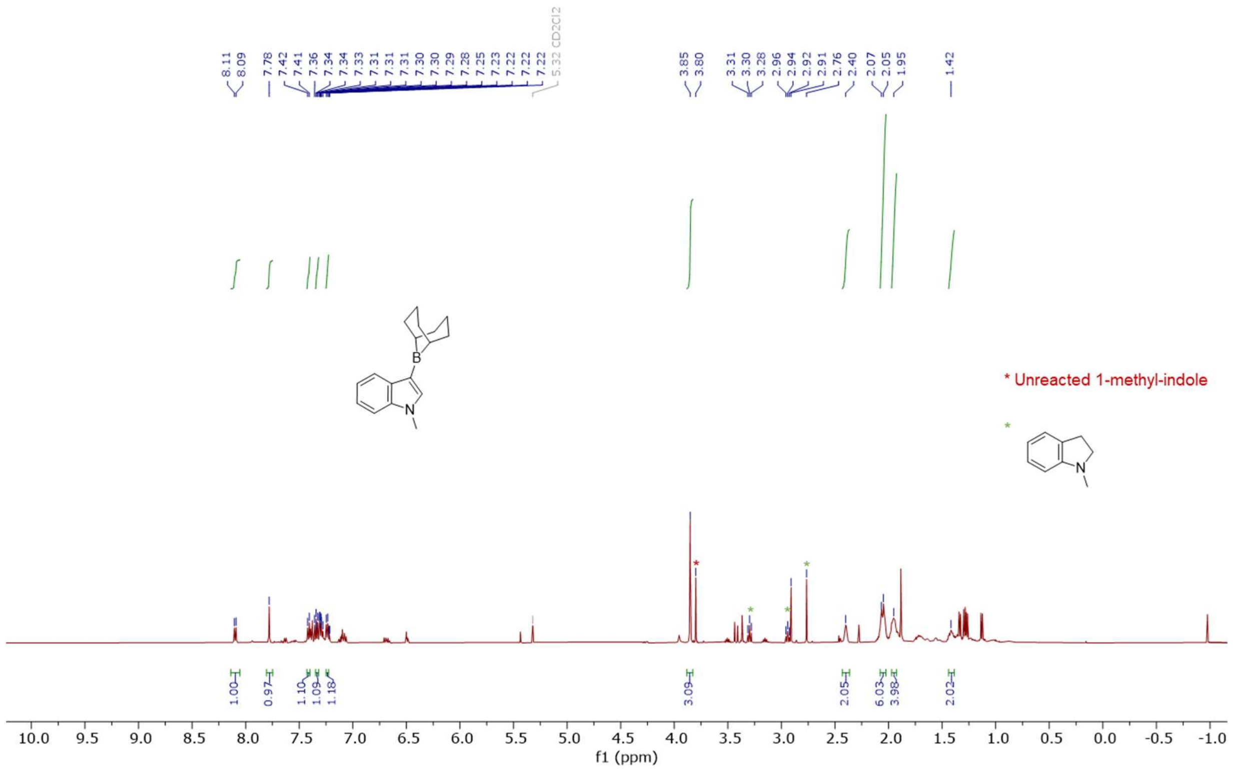

Figure S60: ^{1}H NMR spectroscopy from the crude reaction mixture in CD_2Cl_2.

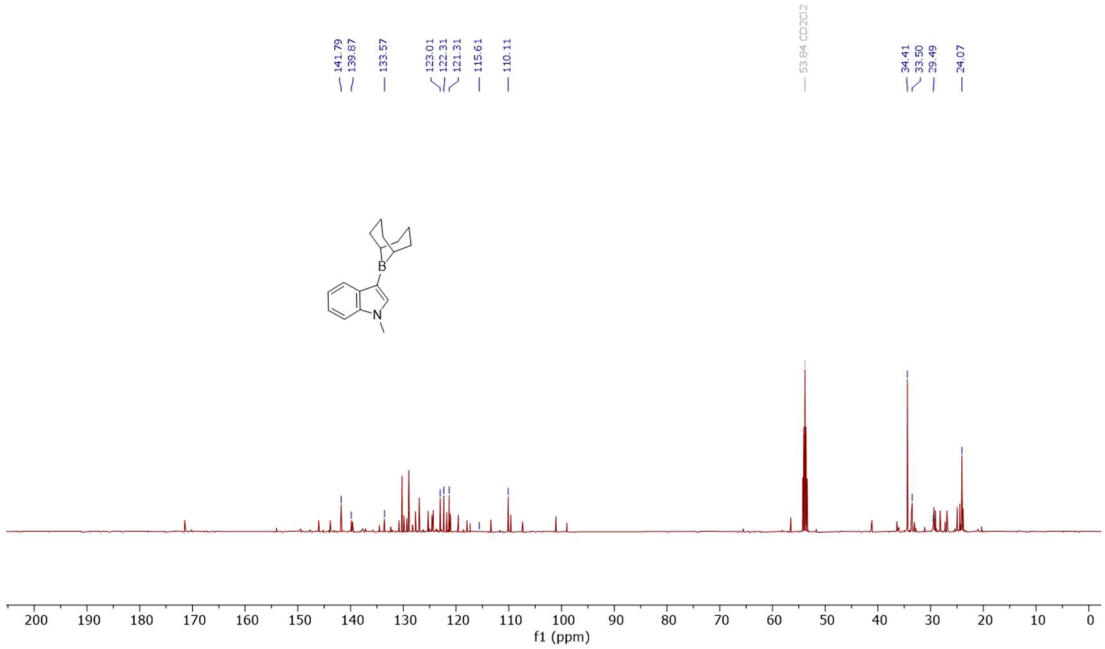

Figure S61: $^{13}C\{^{1}H\}$ NMR spectroscopy from the crude reaction mixture in CD_2Cl_2.

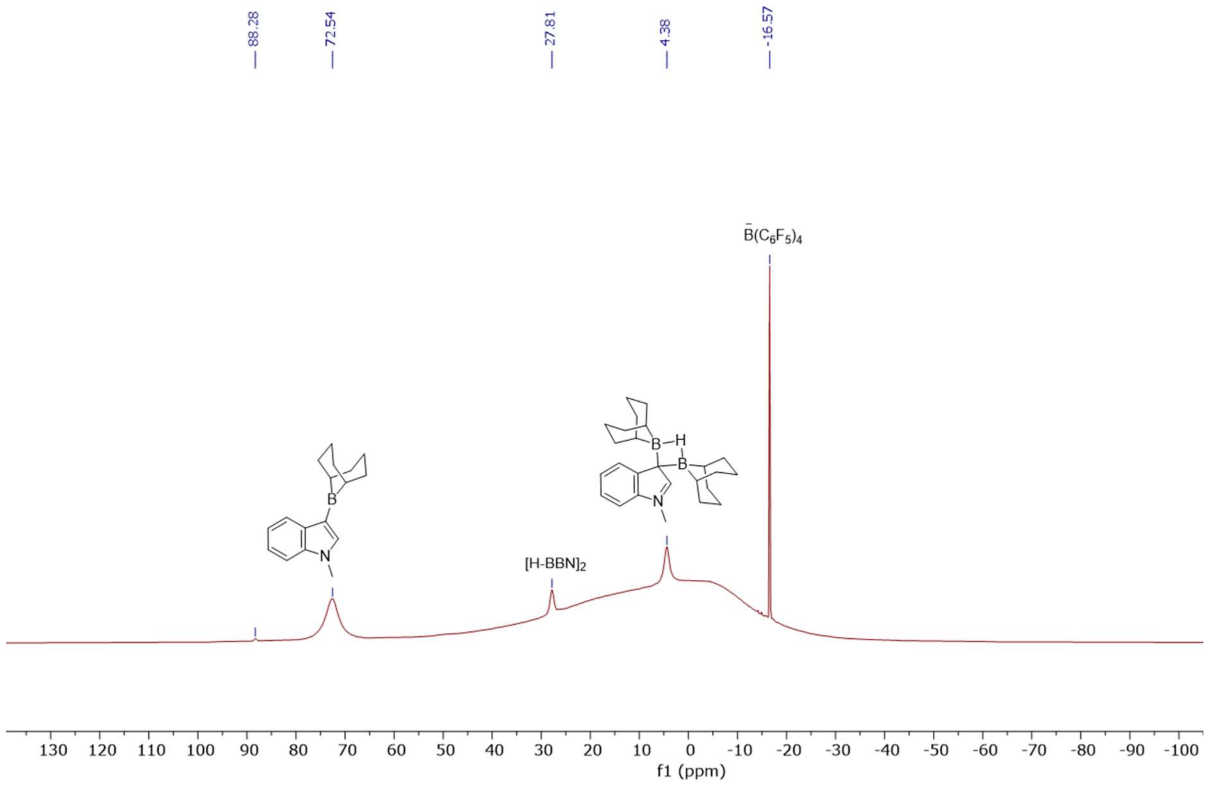

Figure S62: ^{11}B NMR spectroscopy from the crude reaction mixture in CD_2Cl_2.

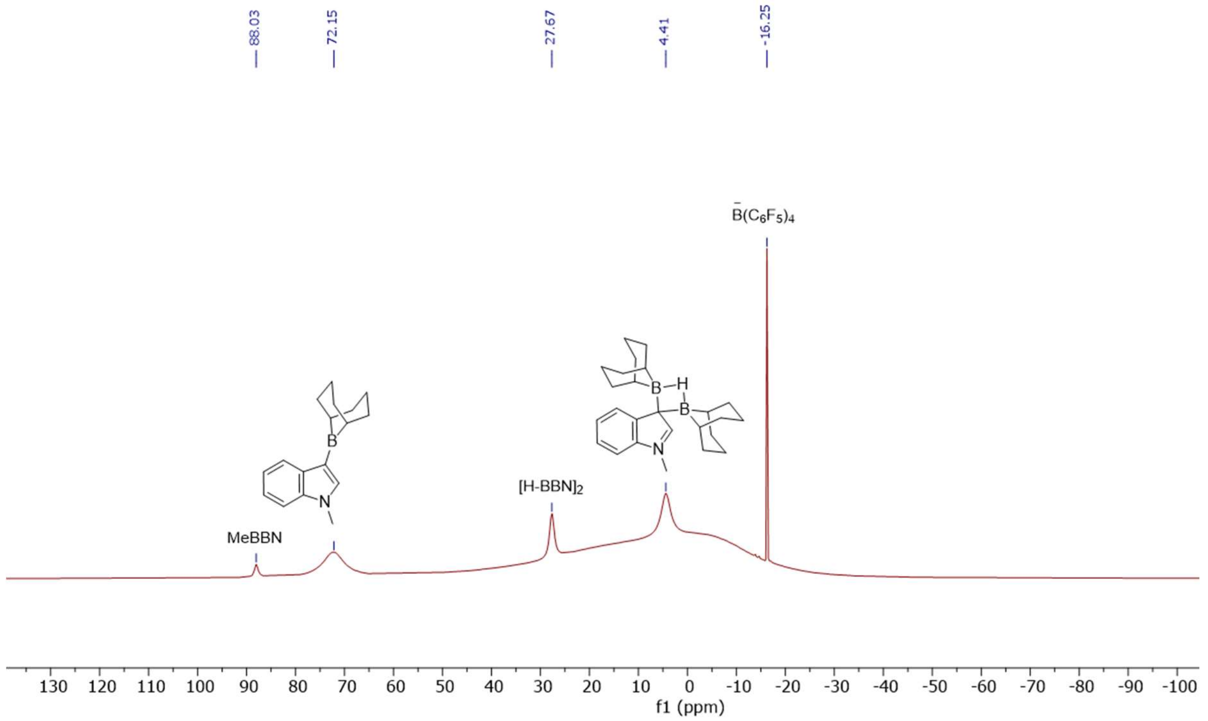

Figure S63: ^{11}B NMR spectroscopy from the crude reaction mixture in PhCl.

S3.7. General procedure 6: Aluminium catalysed C–H di-borylation of (hetero)arenes

In a glovebox, $[H{-}BBN]_2$ (91.5 mg, 0.375 mmol of dimer, 1.5 equiv.), $^{Dipp}NacNacAlMe_2$ (12 mg, 0.0250 mmol) and Brønsted acid (20.5 mg, 0.0250 mmol) charged in a J. Young's NMR tube were dissolved in PhCl (0.5 mL). Subsequently, the corresponding heteroarene (0.250 mmol, 1.0 equiv.) was added to the reaction mixture and heated at 120 °C for 48 h. Upon completion, trimethoxybenzene (12.5 μL, 0.025 mmol) was added to the reaction mixture as an internal standard to determine in situ yield by the integration of diagnostic ^1H resonances. In cases where the diagnostic peak in ^1H NMR spectrum are obscured by chlorobenzene solvent, the reaction mixture was dried and redissolved in CD_2Cl_2 to determine the in situ yield.

Please note, formation of the MeBBN was observed in minor amounts due to the metathesis reaction between $^{Dipp}NacNacAlMe_2$ and $[H{-}BBN]_2$.

S3.7.1. Synthesis of 2,4-Bis(9-borabicyclo[3.3.1]nonan-9-yl)-1-methyl-pyrrole, **3j**

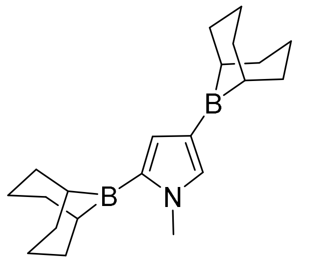

3j

As per general procedure 6, using 1-methylpyrrole (22.0 μL, 0.250 mmol, 1.0 equiv.). In situ yield by integration of diagnostic ^1H resonances versus trimethoxybenzene in CD_2Cl_2 (39% yield by ^1H NMR spectroscopy).

^1H NMR (500 MHz, CD_2Cl_2): δ 7.66 (s, 1H, $^{Pyrrole}CH$), 7.59 (s, 1H, $^{Pyrrole}CH$), 3.96 (s, 3H, N-*Me*), 2.25 (br., 2H, BBN).

$^{13}C\{^1H\}$ NMR (126 MHz, CD_2Cl_2): δ 142.9, 140.6 (br.), 134.6, 124.2, 38.4, 34.6, 34.5, 29.4 (br.), 29.0 (br.), 24.0, 23.9.

^{11}B NMR (160 MHz, CD_2Cl_2): δ 72.7, 69.5.

Analytical data are consistent with that previously reported.[8]

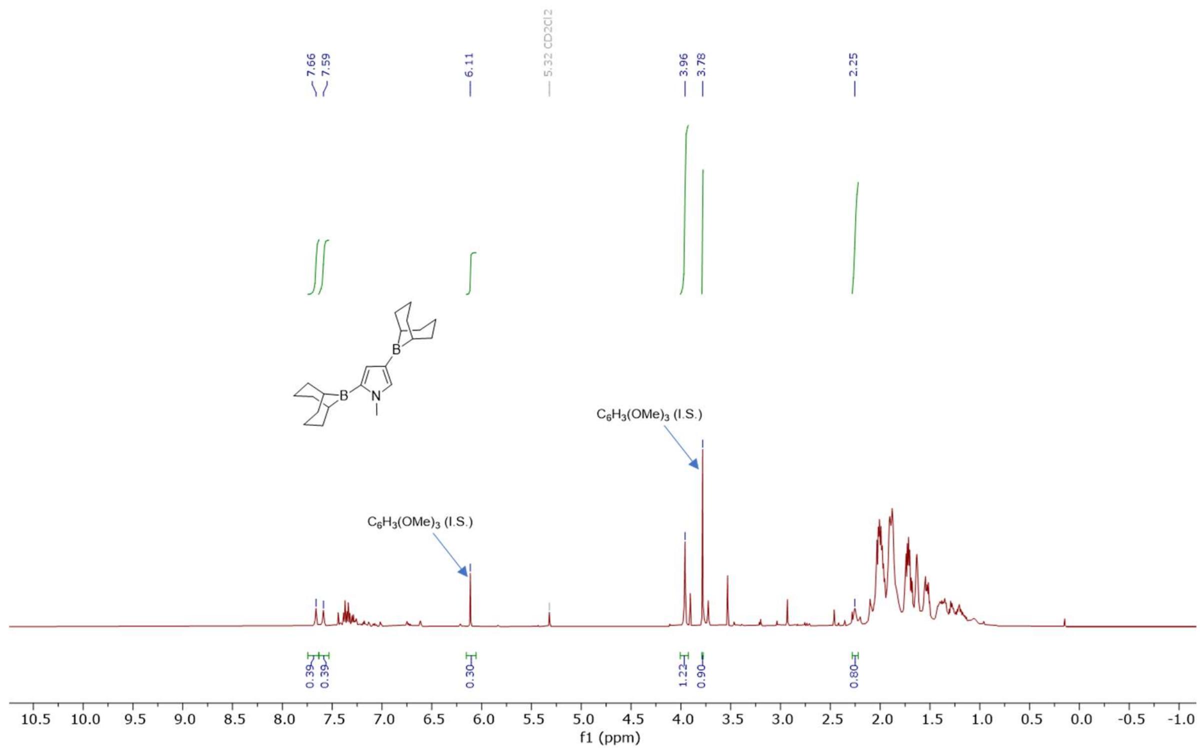

Figure S64: C–H borylation of 1-methylpyrrole in CD_2Cl_2 for determination of NMR yield.

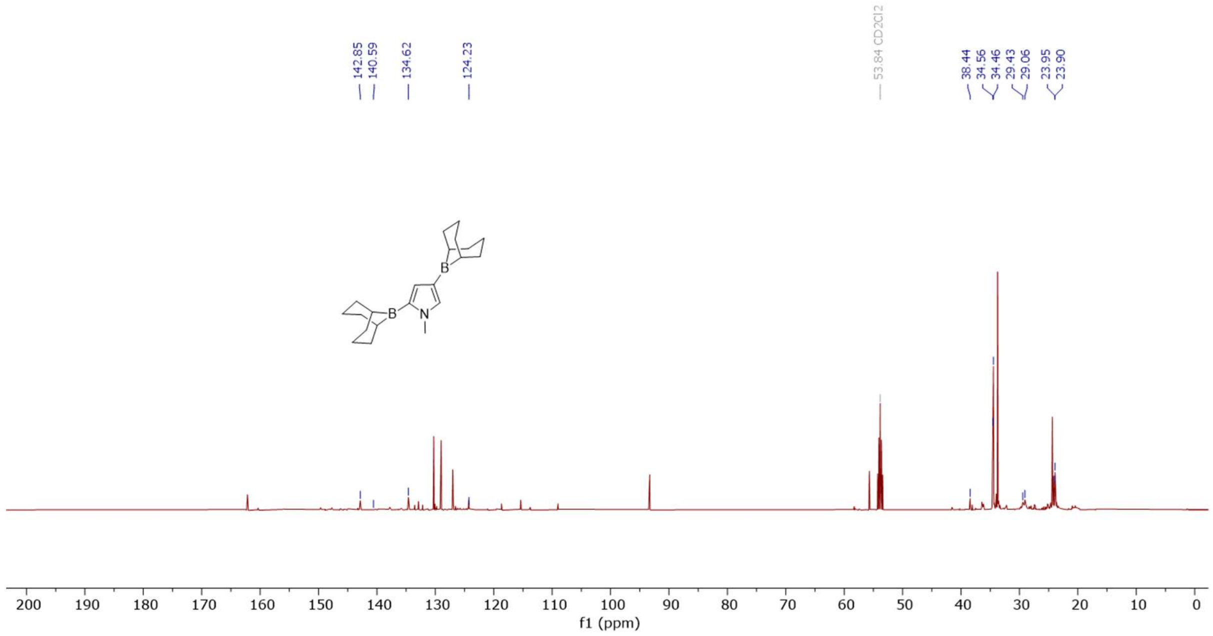

Figure S65: $^{13}C\{^{1}H\}$ NMR spectroscopy from the crude reaction mixture in CD_2Cl_2.

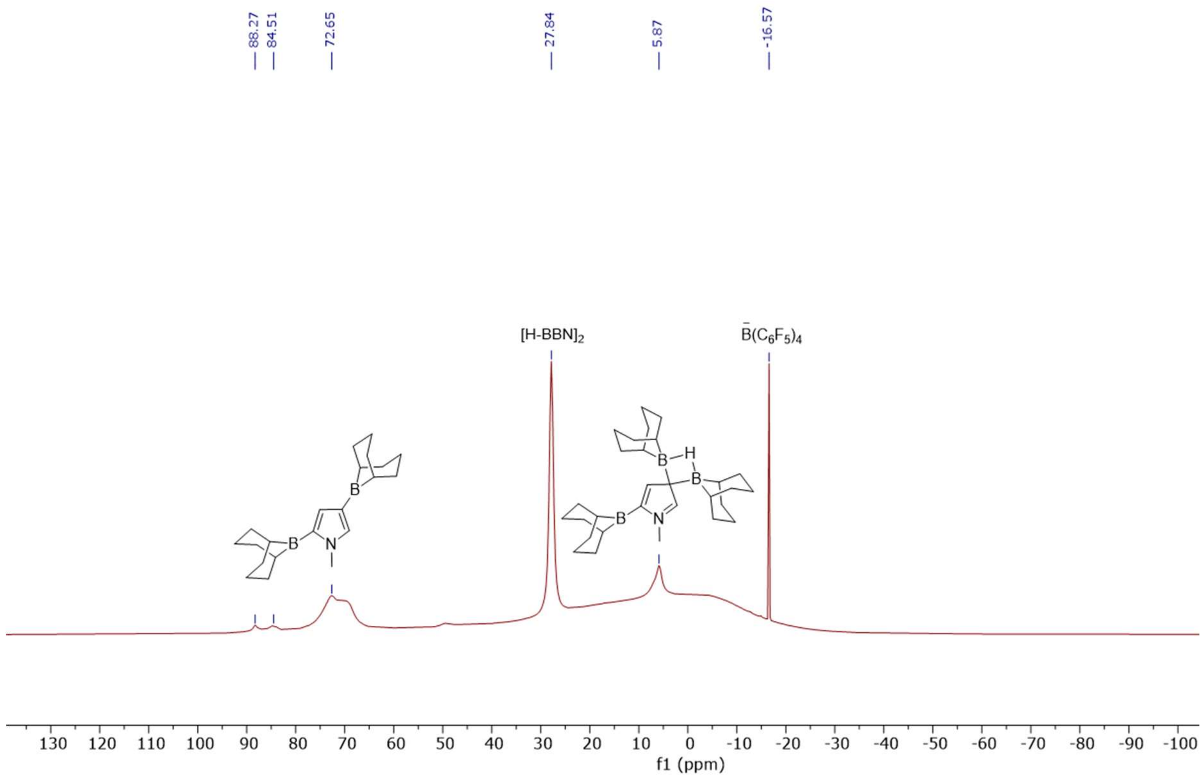

Figure S66: ^{11}B NMR spectroscopy from the crude reaction mixture in CD_2Cl_2.

S3.8. Unsuccessful substrates for aluminium catalysed C−H borylation

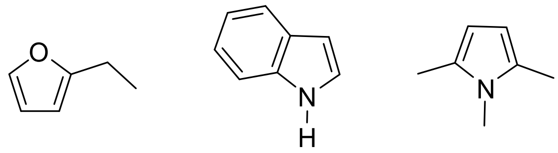

S3.9. Equilibrium studies of H−BBN adducts of aryl-BBN compounds

S3.9.1. Analysis of **3i[H−BBN]**

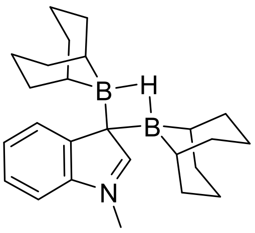

3i-[H-BBN]

From a reaction producing **3i** a small quantity of crystals suitable for X-ray diffraction analysis were produced by slow evaporation of d_2-dichloromethane. These were not **3i** but the H–BBN adduct of **3i**, termed **3i-[H–BBN]**.

Due to the equilibrium between **3i** and **3i-[H–BBN]** favouring the former, we were unable to obtain full characterisation data for **3i-[H–BBN]**. The variable temperature (VT) analysis at 60 ºC of the crystals of **3i-[H–BBN]** dissolved in PhCl showed regeneration of **3i** and $[H{-}BBN]_2$ in solution, suggesting **3i** is the thermodynamically favoured product (see Figure S67). Based on ^{11}B NMR spectroscopy, the ΔG° was calculated as +0.21 kcal mol^{-1} (see Figure S68).

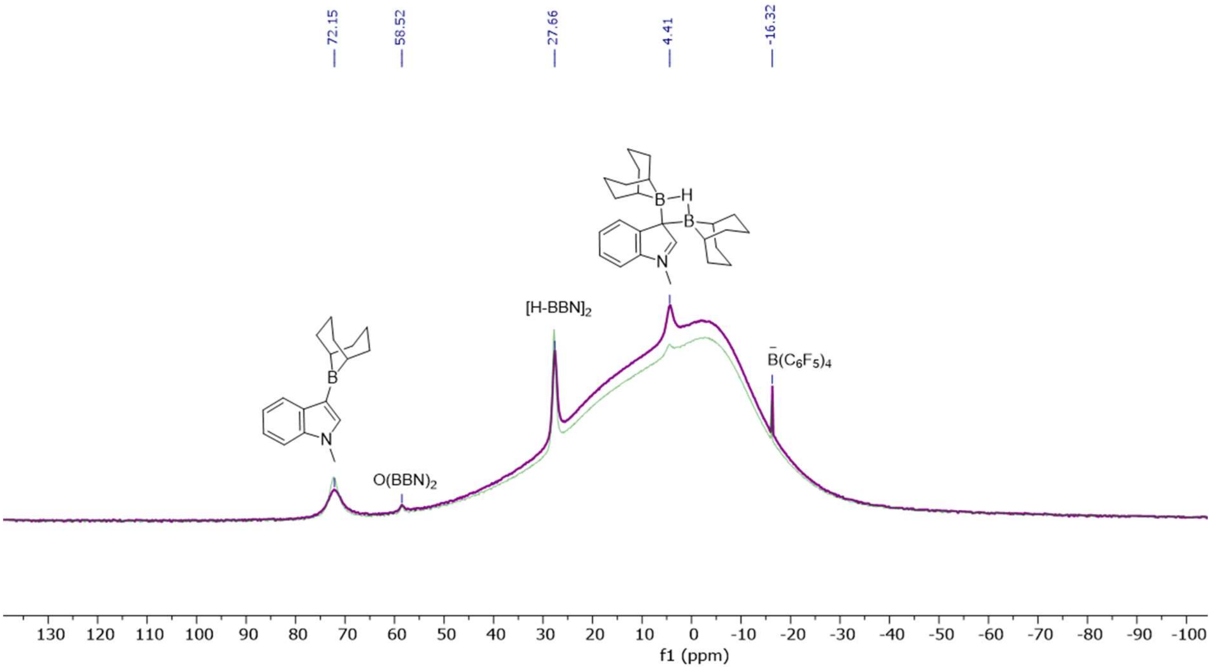

Figure S67: ^{11}B NMR spectroscopy for the VT analysis of crystals of **3i-[H–BBN]** dissolved in PhCl at room temperature (purple) and at 60 ºC (green).

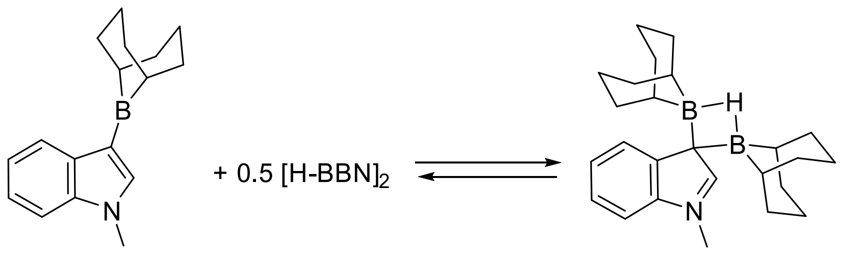

3i **3i-[H-BBN]**

$$\Delta G^\circ = -RT\ln K = -RT\ln\left(\frac{[\mathbf{3i{-}[H{-}BBN]}]}{[\mathbf{3i}][[H{-}BBN]_2]^{0.5}}\right) =$$

$$= -\,8.314\ \text{J mol}^{-1}\ \text{K}^{-1} * 300.1\ \text{K} * \ln\left(\frac{0.36}{0.63(0.67)^{0.5}}\right) =$$

$$= 896.7\ \text{J mol}^{-1} = 0.21\ \text{kcal mol}^{-1}$$

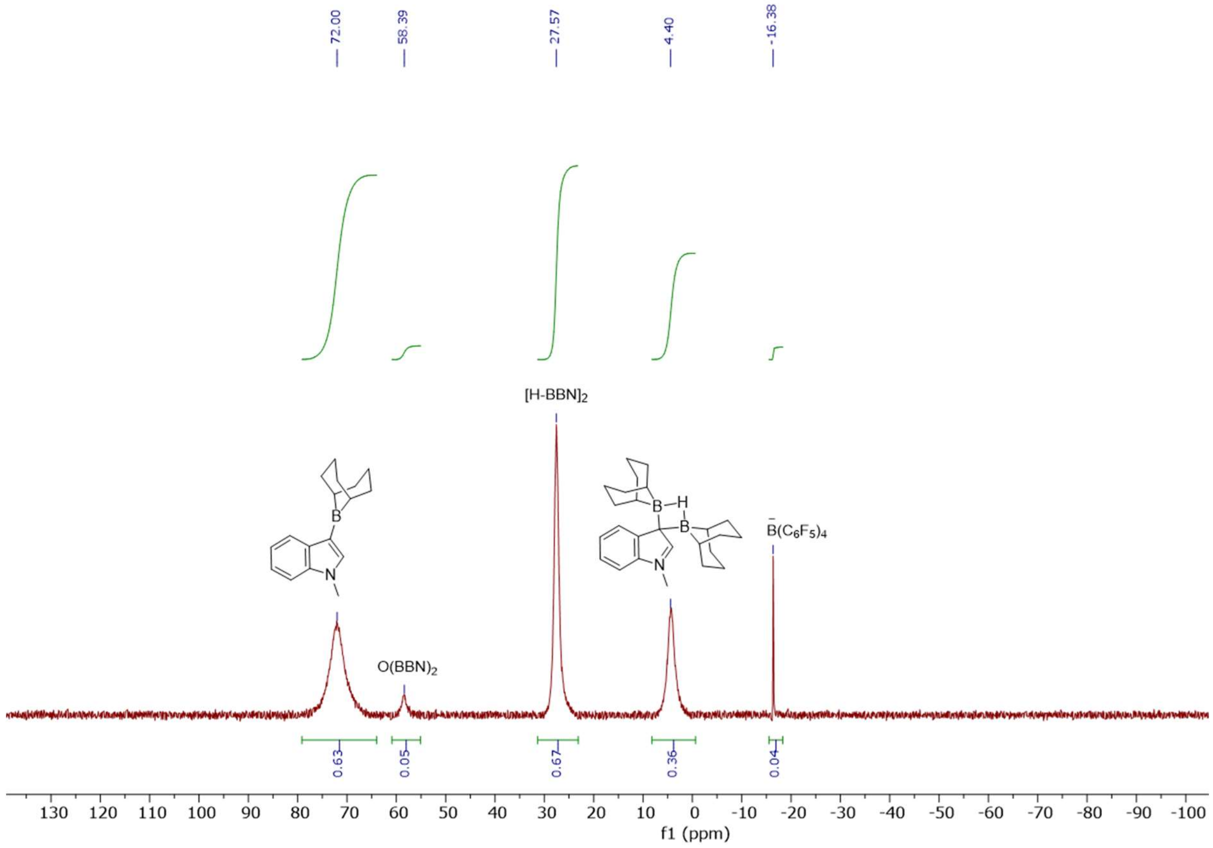

Figure S68: Reaction equation and ΔG° calculations (top) and ^{11}B NMR spectrum of crystals of **3i-[H–BBN]** dissolved in PhCl (bottom).

S3.9.1. Analysis of **3j[H−BBN]**

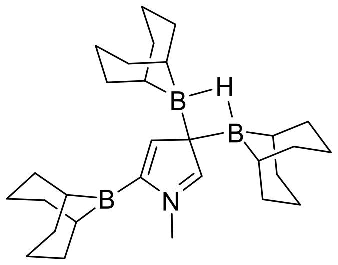

3j-[H-BBN]

From a reaction producing **3j** a small quantity of colourless crystals suitable for X-ray diffraction analysis were produced by slow evaporation of dichloromethane. These were not **3j** but the H–BBN adduct of **3j**, termed **3j-[H–BBN]**. However, upon dissolution in CD_2Cl_2, presence of only **3j** and $[H{-}BBN]_2$ could be identified. Due to the equilibrium between **3j** and **3j-[H–BBN]** favouring the former we were unable to obtain full characterisation data for **3j-[H–BBN]**.

Crystals of **3j-[H–BBN]** (3 mg, 0.007 mmol) were redissolved in PhCl. Addition of extra $[H{-}BBN]_2$ (28.0 mg, 0.115 mmol of dimer) resulted in only minor reformation of the peak at δ 5.87 ppm in the ^{11}B NMR spectrum (see Figure S71).

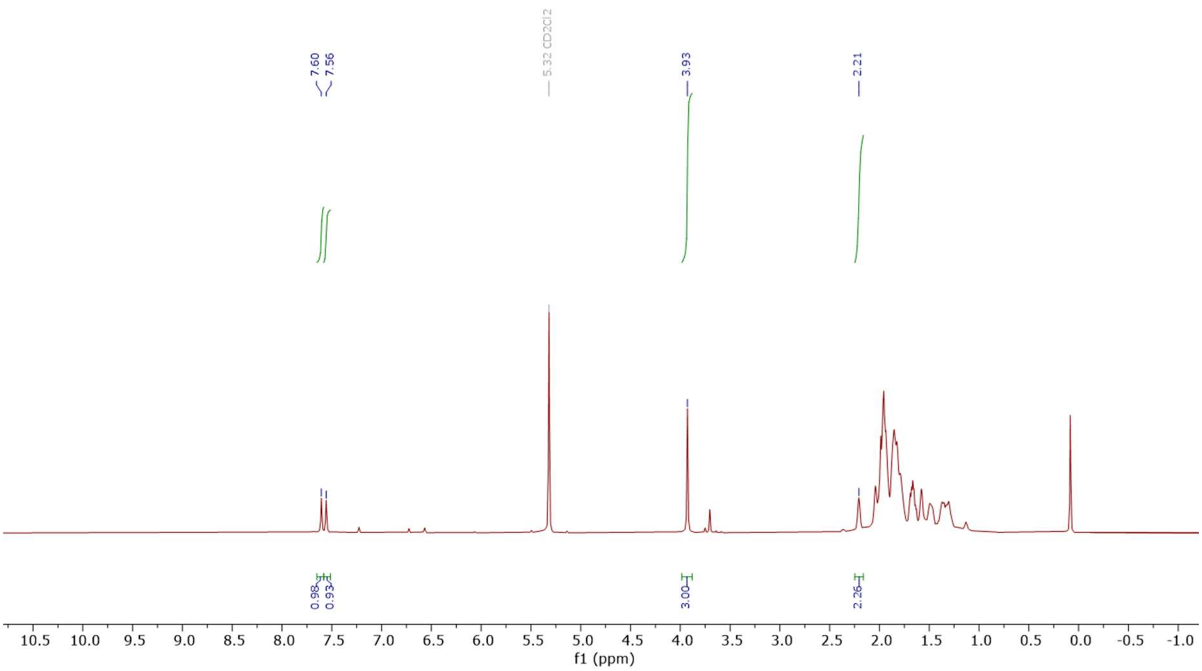

Figure S69: ^1H NMR spectrum of crystals of **3j-[H–BBN]** dissolved in CD_2Cl_2.

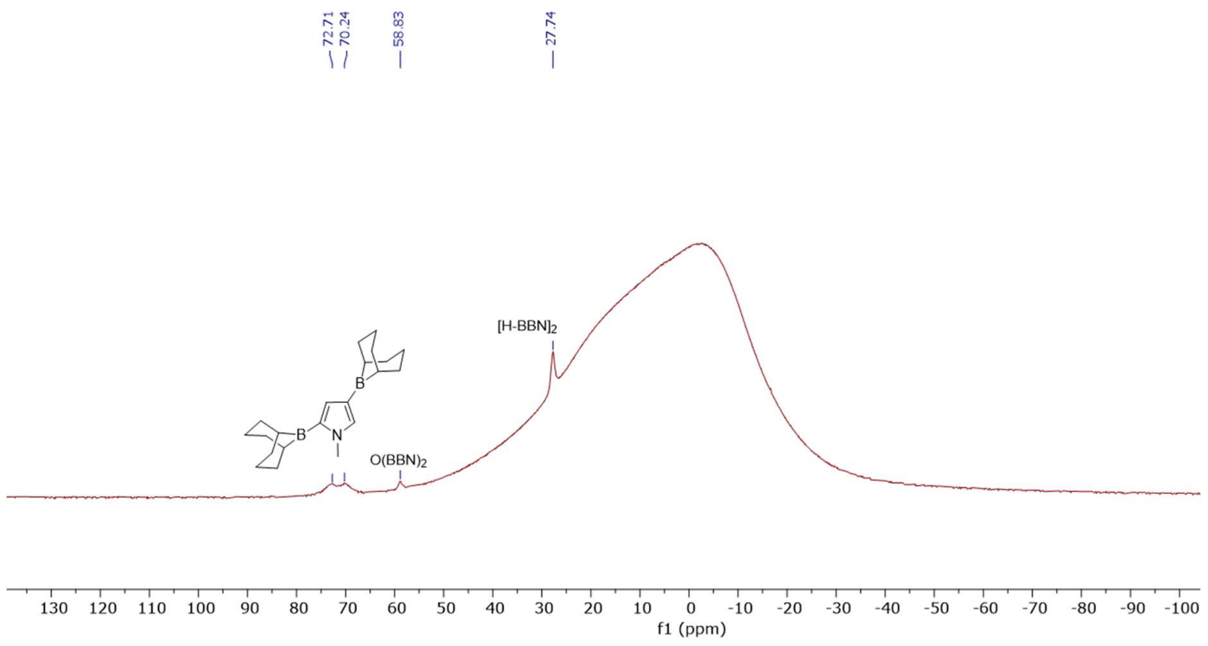

Figure S70: ^{11}B NMR spectrum of crystals of **3j-[H–BBN]** dissolved in CD_2Cl_2.

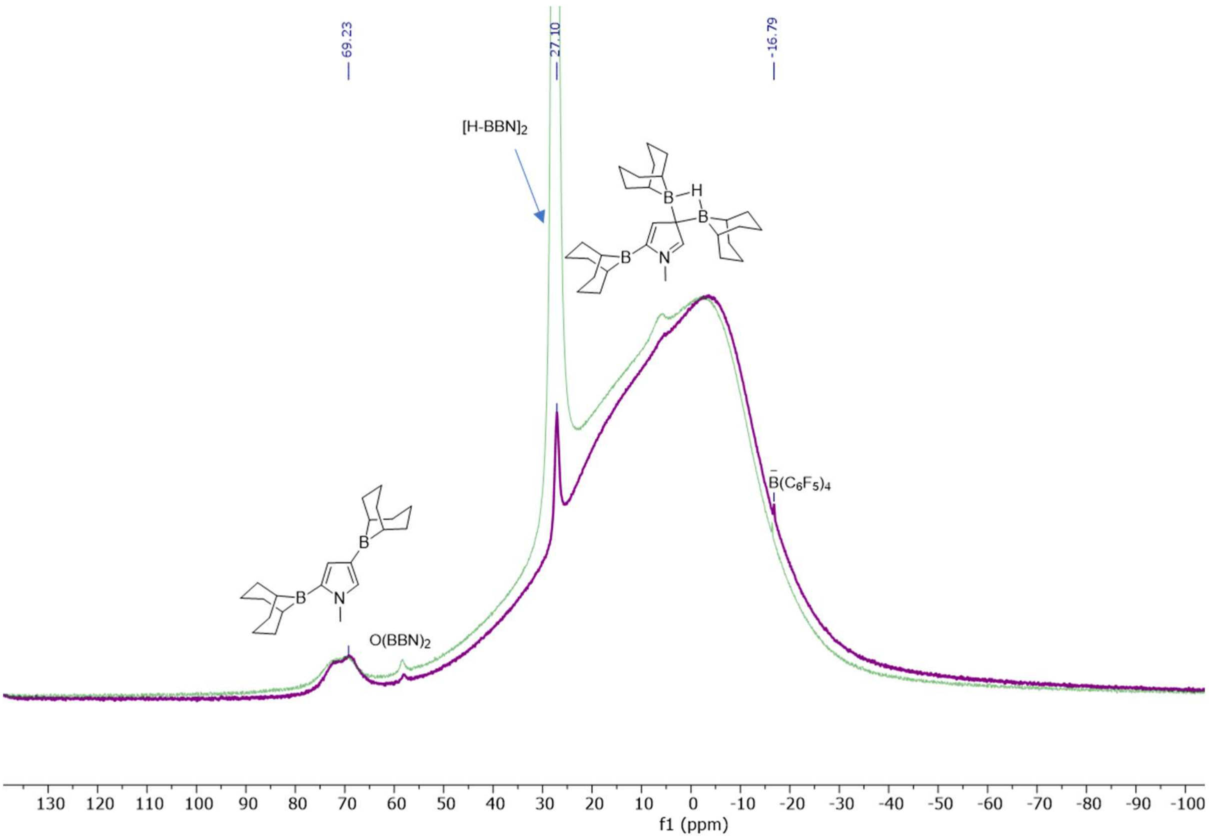

Figure S71: ^{11}B NMR spectroscopy of crystals of **3j-[H–BBN]** dissolved in PhCl (purple) versus upon the addition of extra $[H{-}BBN]_2$ (green).

S4. Synthetic Utility of aryl-BBN compounds

S4.1. Grignard type reaction: Reaction of in situ generated 2-(9-borabicyclo[3.3.1]nonan-9-yl)-5-bromo-thiophene (**3c**) with benzaldehyde

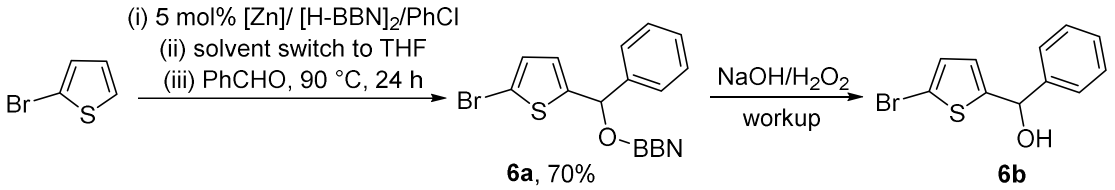

6a, 70% **6b**

Compound **3c** was prepared in situ in a J. Young's NMR tube as per general procedure 2, using 2-bromo-thiophene (28.0 μL, 0.287 mmol) and heating at 80 °C for 24 h. Upon completion, chlorobenzene solvent was removed under vacuum and the compound was redissolved in 0.6 mL THF and then PhCHO (25.5 μL, 1.0 equiv., 0.25 mmol) was added at room temperature. The NMR tube was sealed under argon and the reaction mixture was heated at 90 °C for 24 h. Upon completion, dibromomethane (17.5 μL, 0.250 mmol) was added to the reaction mixture as an internal standard (to determine in situ yield) which revealed a 70% yield by the integration of diagnostic ^{1}H [R_1R_2CH(OBBN)] resonance.

Compound **6a** can be hydrolysed to the corresponding alcohol **6b** upon treatment with NaOH/H_2O_2 following a previously reported protocol[10] and followed by silica gel column chromatography using pet. ether and ethyl acetate (95:5 vol%) as an eluent.

Compound 6a

^{1}H NMR (500 MHz, C_6D_6): δ 7.33-7.31 (m, 2H, Ph), 7.12-7.09 (m, 2H, Ph), 7.06-7.03 (m, 1H, Ph), 6.57 (d, J = 3.8 Hz, 1H, $^{Thienyl}CH$), 6.29 (dd, J = 3.8, 1.3 Hz, 1H, $^{Thienyl}CH$), 6.23 (s, 1H, CH), 1.89-1.87 (m, 2H, BBN), 1.81-1.71 (m, 8H, BBN), 1.64-1.57 (m, 2H, BBN), 1.47-1.43 (m, 2H, BBN).

^{13}C{^{1}H} NMR (126 MHz, C_6D_6): δ 149.9, 142.7, 129.7, 128.9, 128.3, 126.8, 125.2, 112.4, 75.9, 34.4, 33.8, 33.5, 33.4, 23.5.

^{11}B NMR (160 MHz, C_6D_6): δ 57.3.

Note, several attempts were made to perform mass spectrometry on this compound, but these all did not show the $[M]^+$ or $[M+H]^+$.

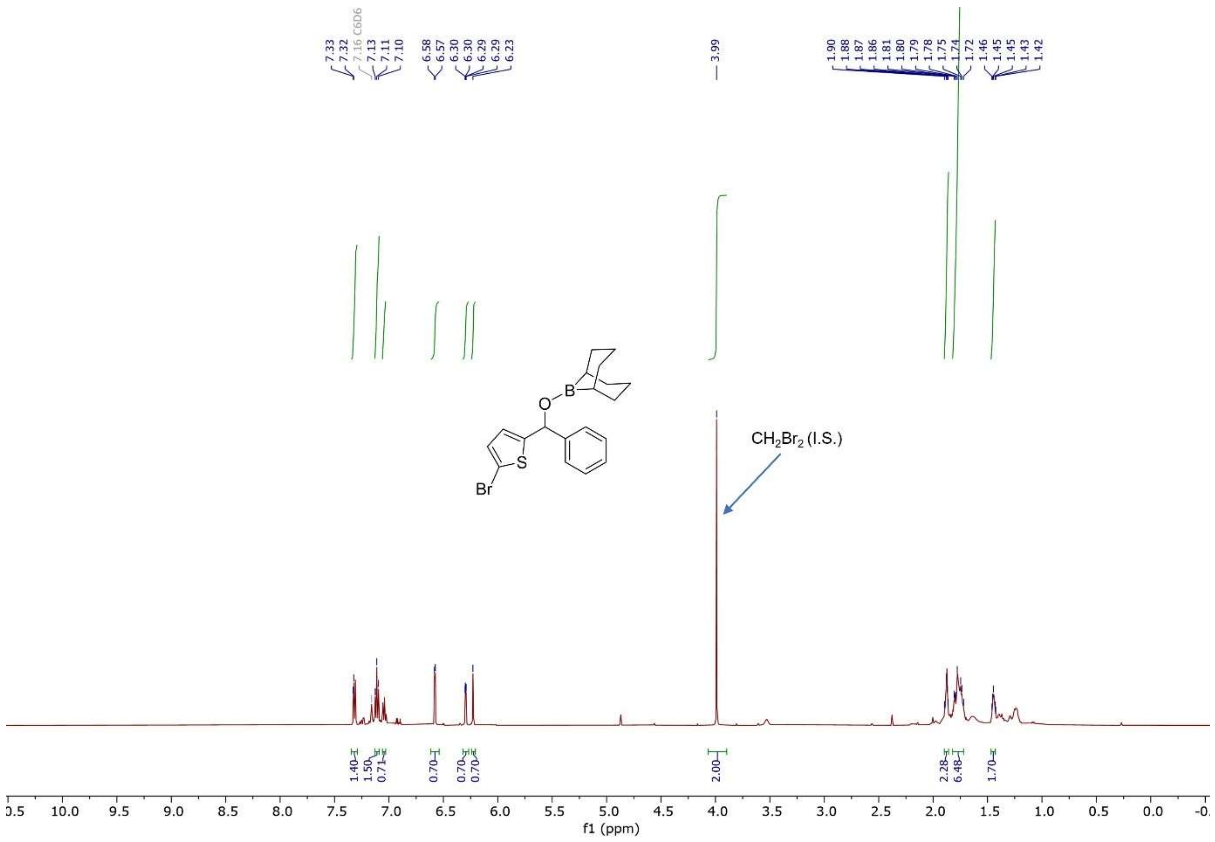

Figure S72: ^{1}H NMR spectrum of compound **6a** in C_6D_6 with internal standard.

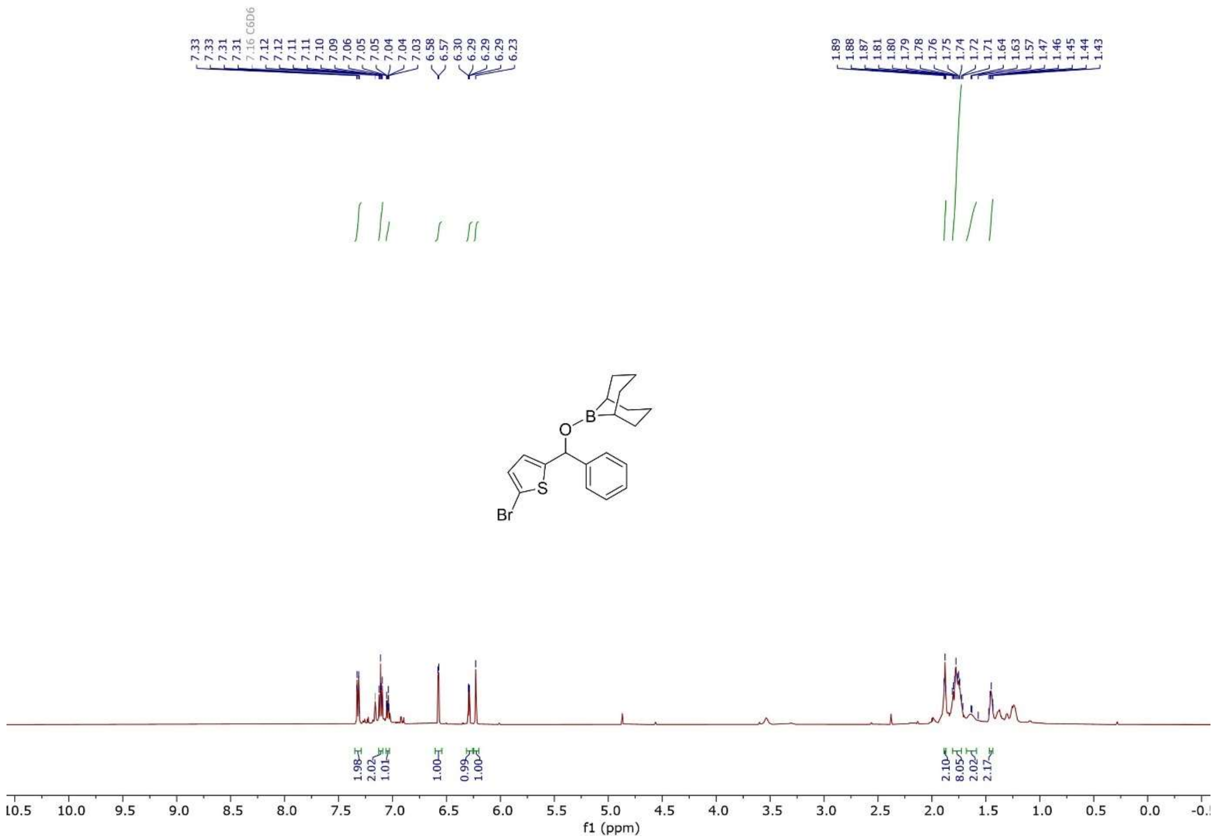

Figure S73: ^{1}H NMR spectrum of compound **6a** in C_6D_6.

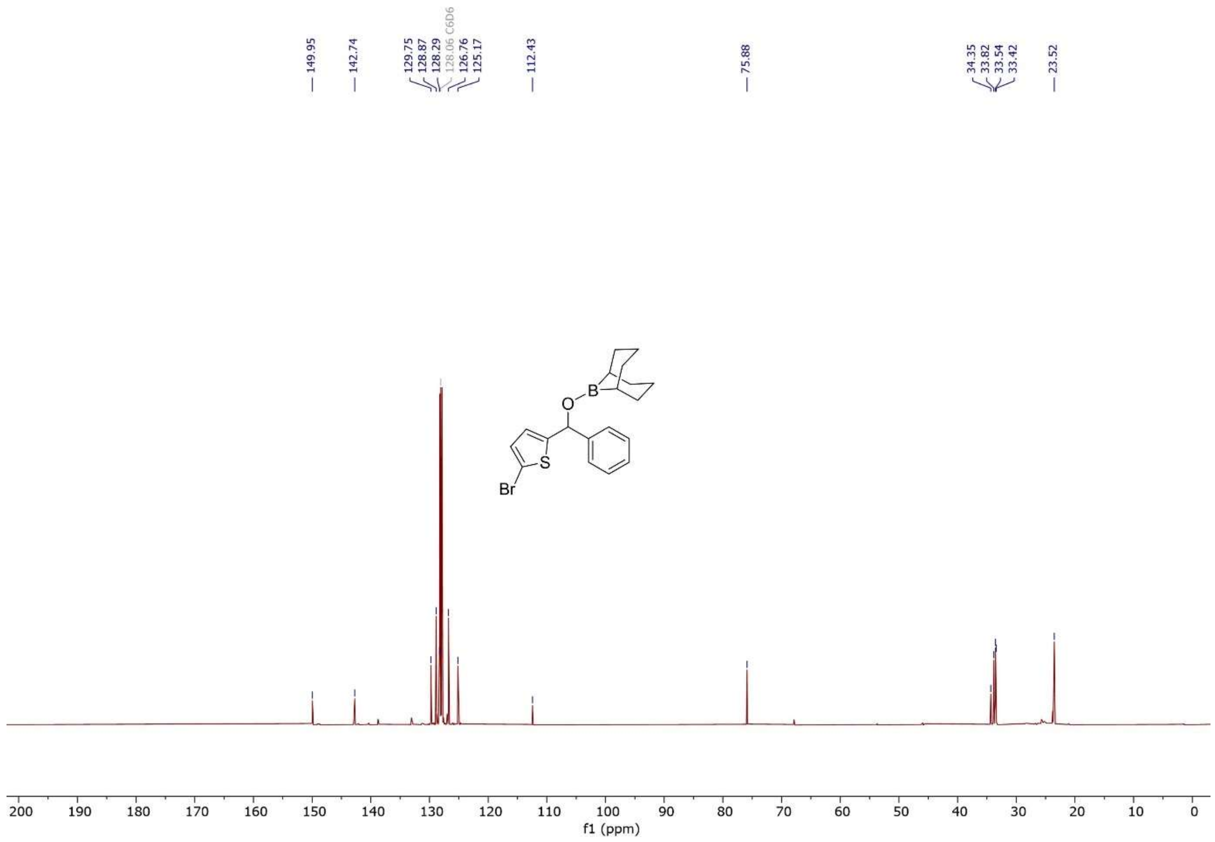

Figure S74: $^{13}C\{^{1}H\}$ NMR spectrum of compound **6a** in C_6D_6.

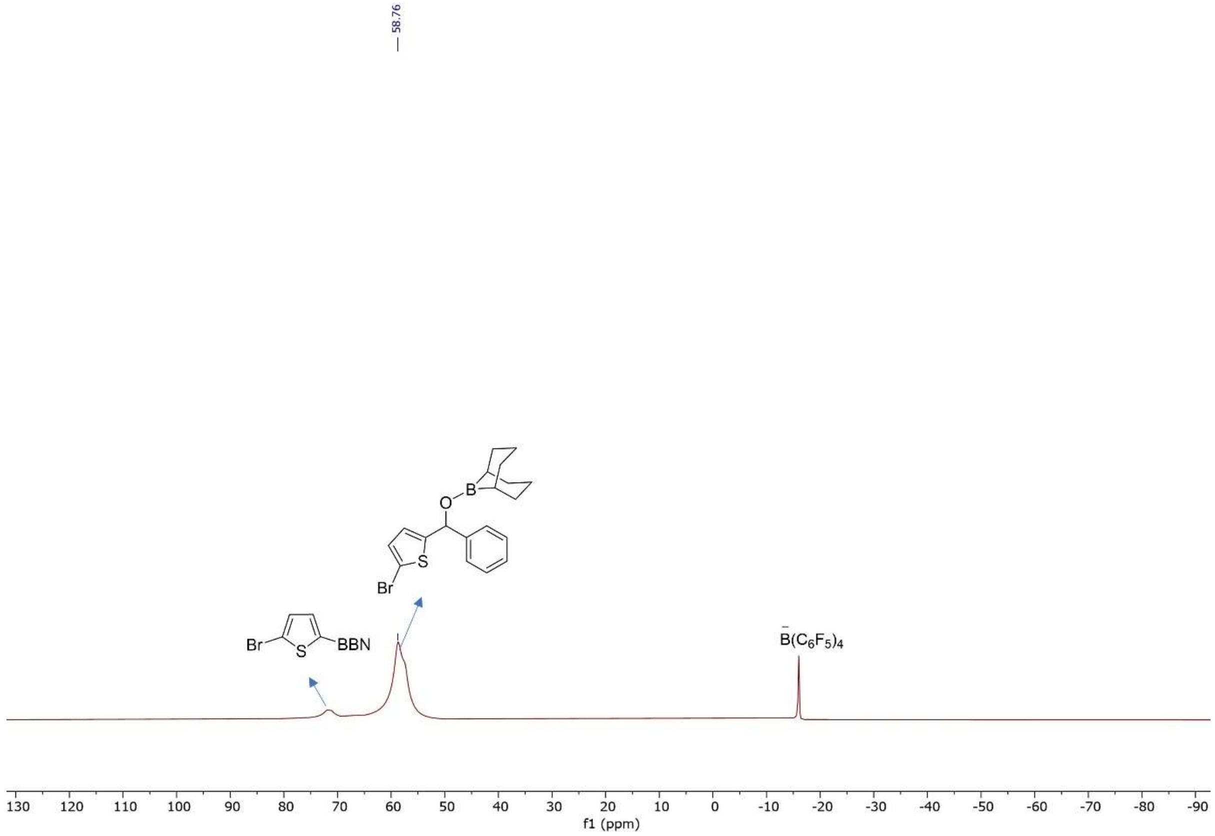

Figure S75: ^{11}B NMR spectrum of compound **6a** (from crude) in C_6D_6.

Compound 6b

^{1}H NMR (500 MHz, CDCl$_3$): δ 7.43-7.31 (m, 5H, Ph), 6.88 (d, J = 3.7 Hz, 1H, ^{Thienyl}C*H*), 6.61 (dd, J = 3.8, 1.0 Hz, 1H, ^{Thienyl}C*H*), 5.93 (s, 1H, CH), 2.55 (s, 1H, O*H*).

^{13}C{^{1}H} NMR (126 MHz, CDCl$_3$): δ 149.9, 142.7, 129.7, 128.9, 126.8, 125.2, 112.4, 75.9, 34.4, 33.8, 33.5, 33.4, 23.5.

Analytical data are consistent with that previously reported.[11]

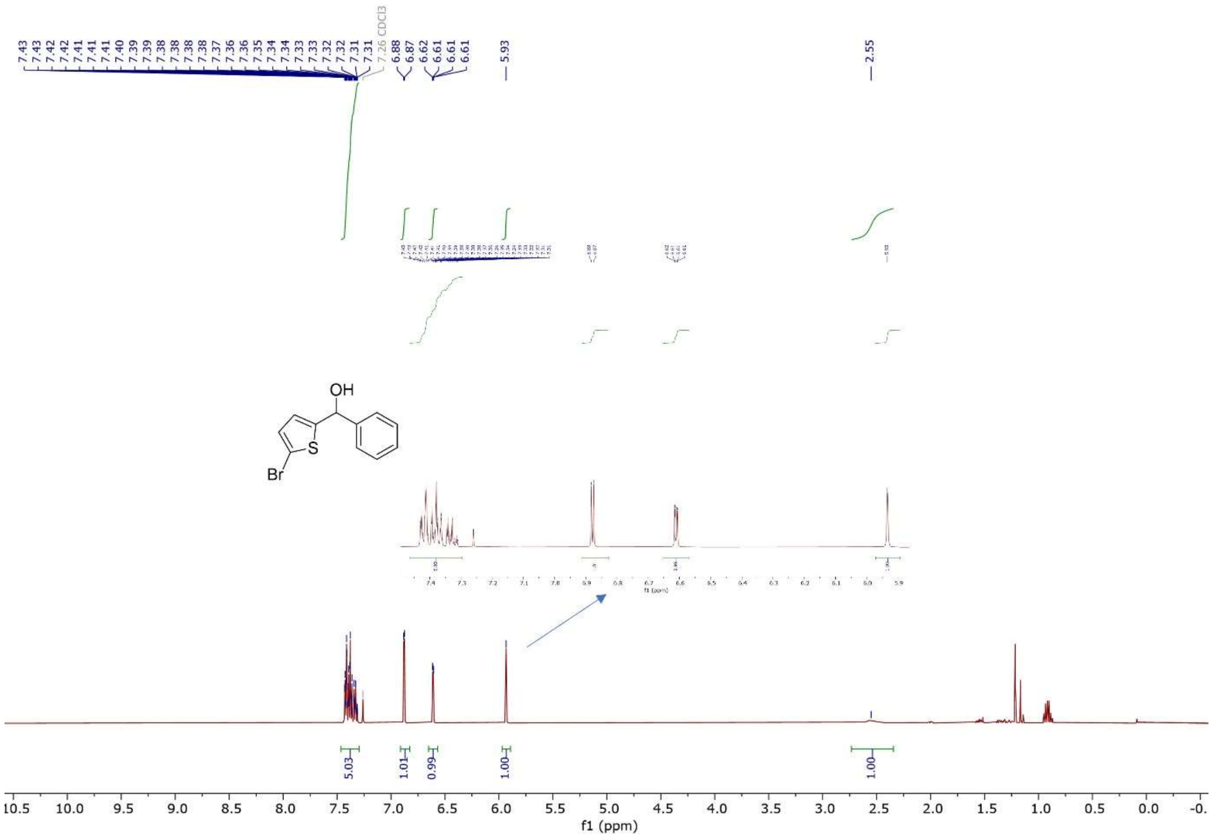

Figure S76: ^{1}H NMR spectrum of compound **6b** in CDCl$_3$.

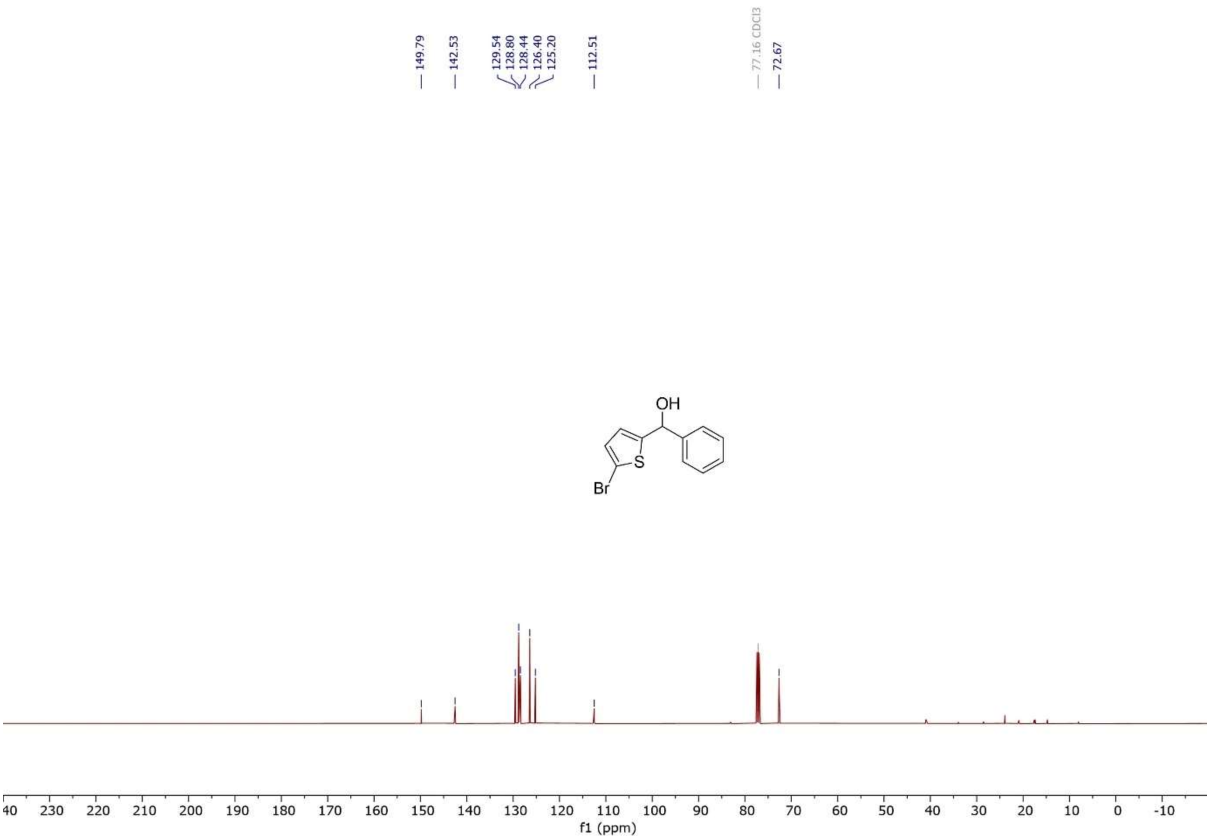

Figure S77: $^{13}C\{^{1}H\}$ NMR spectrum of compound **6b** in $CDCl_3$.

S4.2. Cross coupling reaction of in situ generated **3a** with 4-iodotoluene

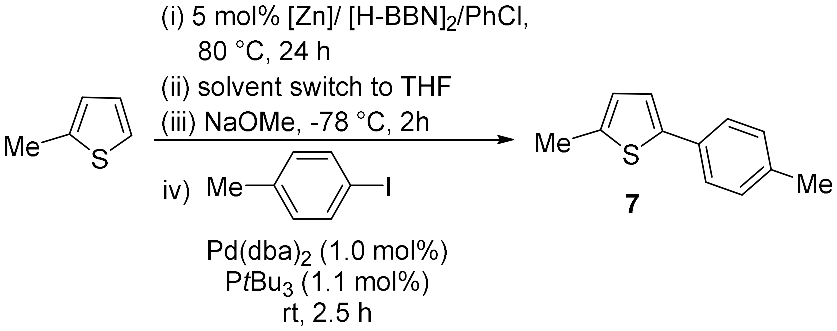

Compound **3c** was prepared in situ in a J. Young's NMR tube as per general procedure 2, using 2-methyl-thiophene (27.5 µL, 0.287 mmol) and heating at 80 °C for 24 h. Upon completion, PhCl solvent was removed under vacuum and the compound was redissolved in 1.0 mL THF. In a separate J. Young's ampoule, 57.0 µL NaOMe solution (25 wt% in methanol) was added and dried under vacuum to afford a white powder, which was then dissolved in 1.0 mL of THF. This NaOMe solution was cooled to -78 °C and a THF solution of **3c** was added slowly to the reaction mixture which was stirred for 2 h at -78 °C. Upon completion, 4-iodotoluene (54.5 mg, 0.25 mmol) was added followed by [Pd(dba)$_2$] (1.0 mol%, 1.4 mg) and P*t*Bu$_3$ (1.1 mol%,

2.5 µL 1.0 M toluene solution). The reaction mixture was slowly warmed to room temperature and stirred for 2.5 h. Upon completion, the reaction mixture was adsorbed into silica gel and purified by silica gel column chromatography using pet ether and ethyl acetate (98:2 vol%) as an eluent to afford off white solid **7**. Yield: 85.0% (38 mg).

Compound 7

^{1}H NMR (500 MHz, CDCl$_3$): δ 7.44 (d, J = 8.1 Hz, 2H, ^{Ph}CH), 7.16 (d, J = 7.8 Hz, 2H, ^{Ph}CH), 7.05 (d, J = 3.5 Hz, 1H, ^{Thienyl}CH), 6.71 (dq, J = 3.5, 1.2 Hz, 1H, ^{Thienyl}CH), 2.50 (d, J = 1.3 Hz, 3H, ^{Thienyl}CH_3), 2.35 (s, 3H, ^{Ph}CH_3).

^{13}C{^{1}H} NMR (126 MHz, CDCl$_3$): δ 142.3, 139.1, 136.9, 132.1, 129.6, 126.2, 125.58, 122.5, 21.3, 15.6.

Analytical data are consistent with that previously reported.[12]

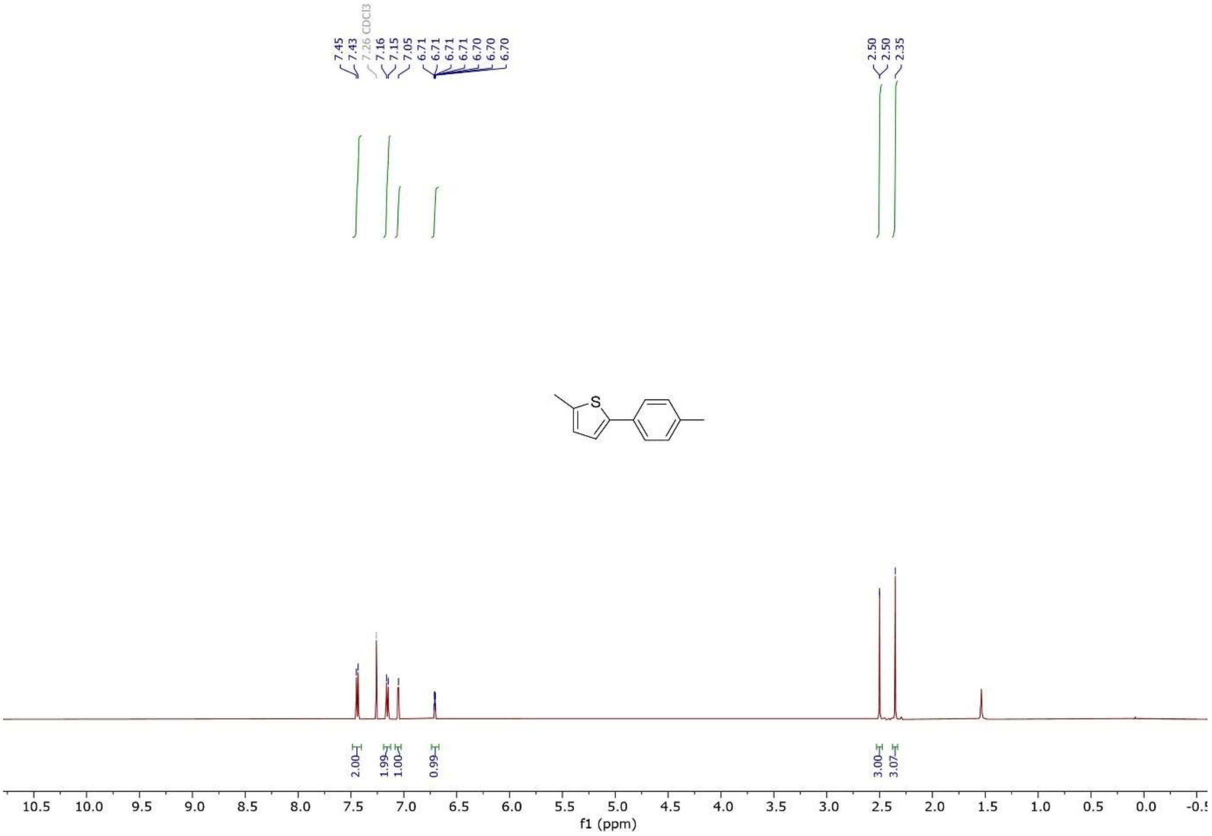

Figure S78: ^{1}H NMR spectrum of compound **7** in CDCl$_3$.

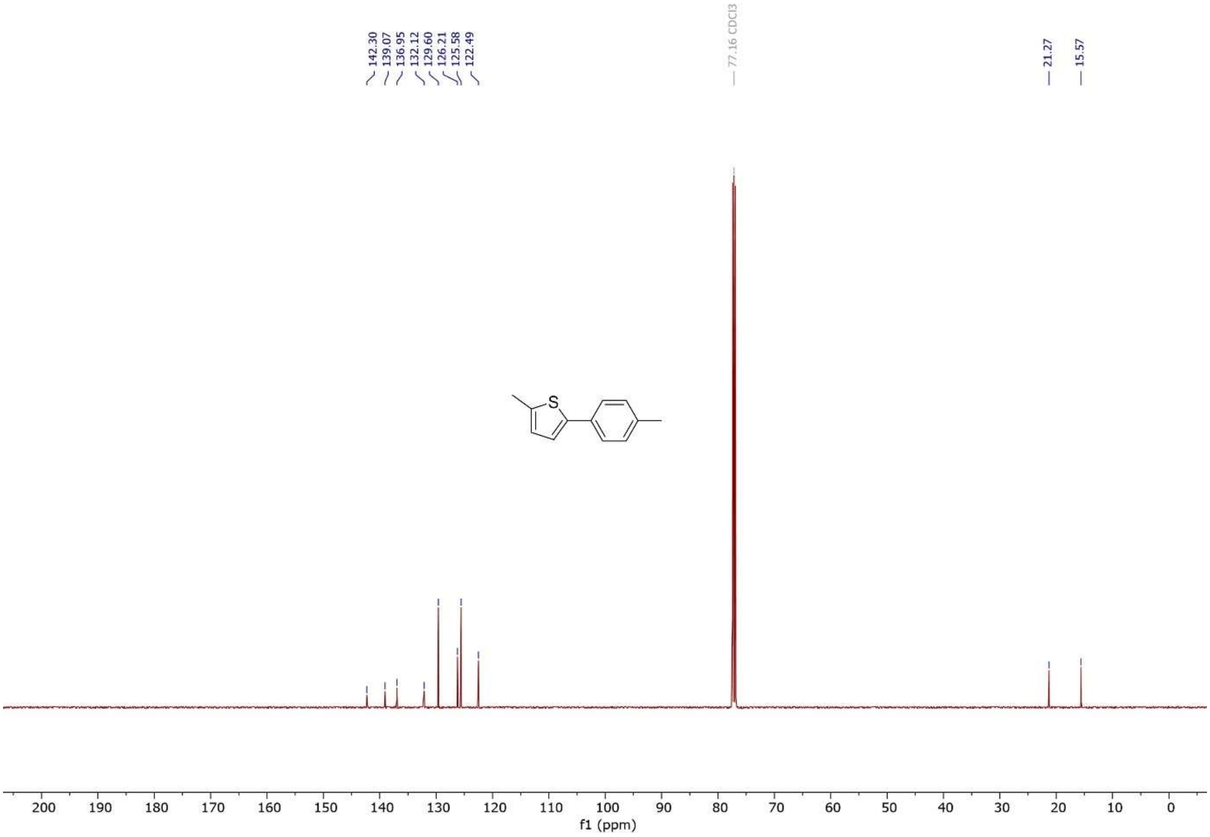

Figure S79: $^{13}C\{^1H\}$ NMR spectrum of compound **7** in $CDCl_3$.

S4.3. Trans-borylation of **5e** with pinacolborane

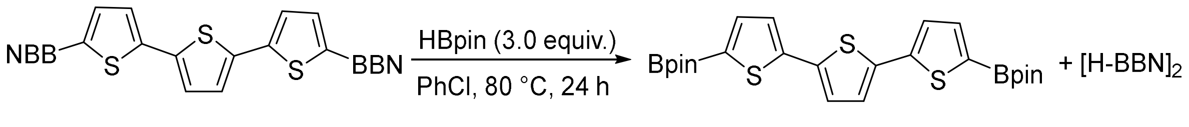

A J. Young's ampoule charged with **5e** (20 mg, 0.040 mmol, 1.0 equiv.) was dissolved in 0.6 mL of PhCl solvent. Following this, pinacolborane (18.0 µL, 0.124 mmol, 3.0 equiv.) was added to the reaction mixture and heated at 80 ºC for 24 hours. NMR analysis showed approximately 92% conversion. Upon completion, PhCl solvent and excess pinacolborane were removed under vacuum. Next, 1 mL of n-pentane was added to the reaction mixture, leading to the precipitation of compound **8** as a clean product. Isolated yield 76% (15.5 mg).

Compound 8

^{1}H NMR (500 MHz, $CDCl_3$): δ 7.53 (d, J = 3.6 Hz, 2H, $^{Thienyl}CH$), 7.24 (d, J = 3.5 Hz, 2H, $^{Thienyl}CH$), 7.14 (s, 2H, $^{Thienyl}CH$), 1.35 (s, 24H, CH_3).

$^{13}C\{^{1}H\}$ NMR (126 MHz, CDCl$_3$): δ 143.8, 138.1, 136.8, 125.3, 125.1, 84.4, 24.9.

^{11}B NMR (160 MHz, CDCl$_3$): δ 29.3.

Mass spectrometry: Calculated for $[C_{16}H_{19}BS]^+$: 254.12950, found 254.12962.

Analytical data are consistent with that previously reported.[13]

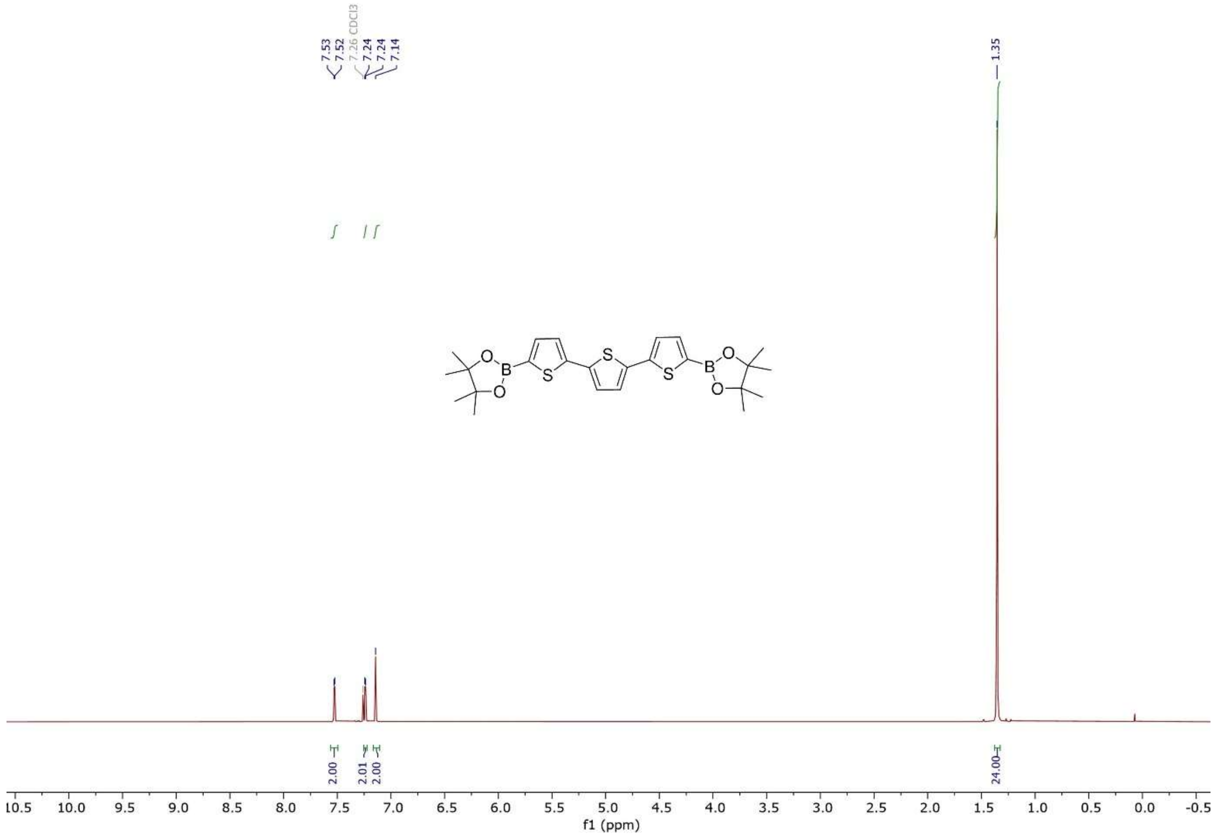

Figure S80: ^{1}H NMR spectrum of compound **8** in CDCl$_3$.

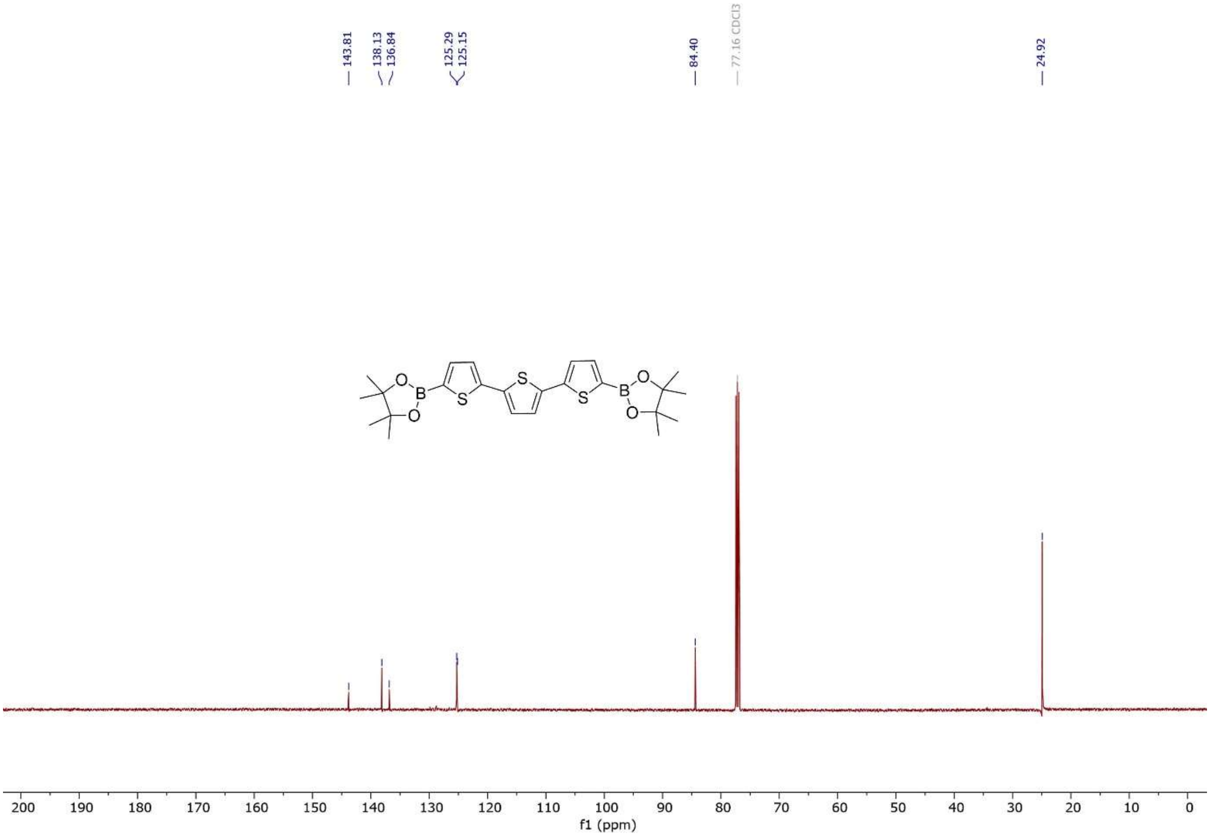

Figure S81: $^{13}C\{^{1}H\}$ NMR spectrum of compound **8** in $CDCl_3$.

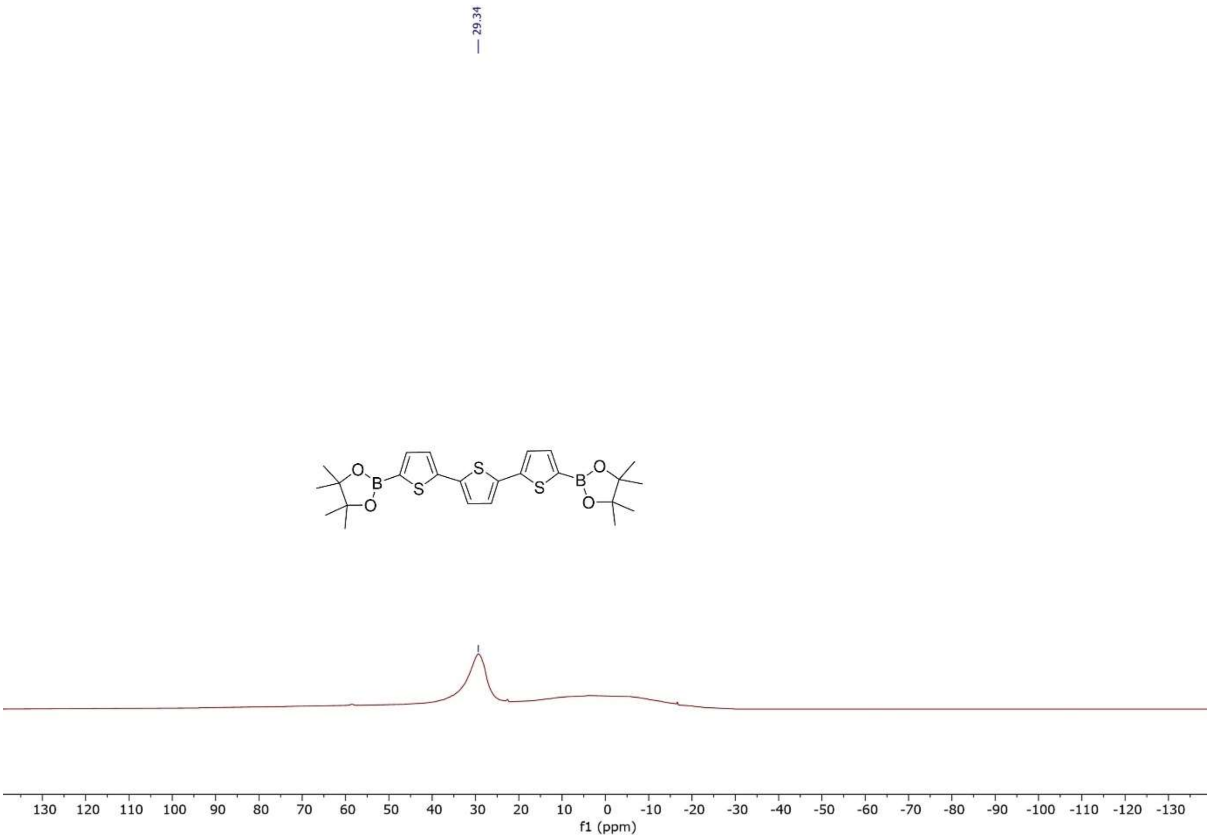

Figure S82: ^{11}B NMR spectrum of compound **8** in $CDCl_3$.

S5. Mechanistic Studies

S5.1. Attempted Catalytic C−H borylation without ^{Dipp}NacNacZnH or $[(DMT)H][B(C_6F_5)_4]$

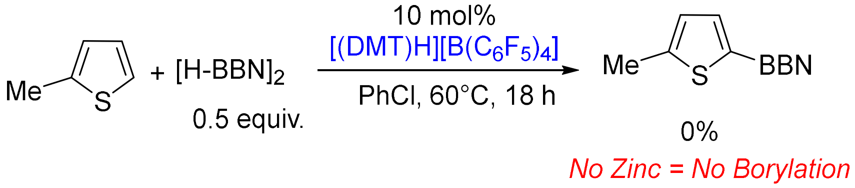

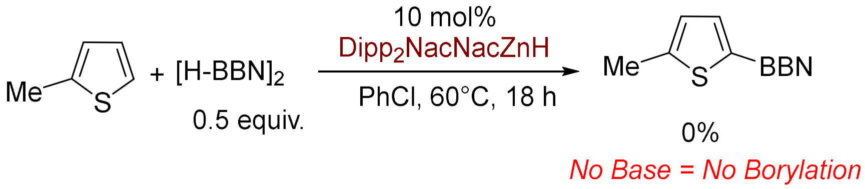

For the top experiment, $[H{-}BBN]_2$ (30.5 mg, 0.125 mmol of dimer, 0.5 equiv.), and $[(DMT)H][B(C_6F_5)_4]$ (21.0 mg, 0.025 mmol) charged in a J. Young's NMR tube were dissolved in PhCl (0.6 mL). Subsequently, 2-methyl-thiopehene (0.250 mmol, 1.0 equiv.) was added to the reaction mixture and it was heated at 60 °C for 18 h.

For the bottom experiment, $[H{-}BBN]_2$ (30.5 mg, 0.125 mmol of dimer, 0.5 equiv.) and ^{Dipp}NacNacZnH (12.0 mg, 0.025 mmol) charged in a J. Young's NMR tube were dissolved in PhCl (0.6 mL). Subsequently, the corresponding 2-methyl-thiopehene (0.250 mmol, 1.0 equiv.) was added to the reaction mixture and heated at 60 °C for 18 h.

Upon completion, dibromomethane (17.5 µL, 0.250 mmol) was added to the reaction mixture as an internal standard to determine in situ yield by the integration of diagnostic ^{1}H (thienyl-BBN) resonances. However, no borylation product was observed in absence of either ^{Dipp}NacNacZnH or $[(DMT)H][B(C_6F_5)_4]$.

S5.2. No reaction between [(DMT)H][B(C_6F_5)$_4$] and [H−BBN]$_2$

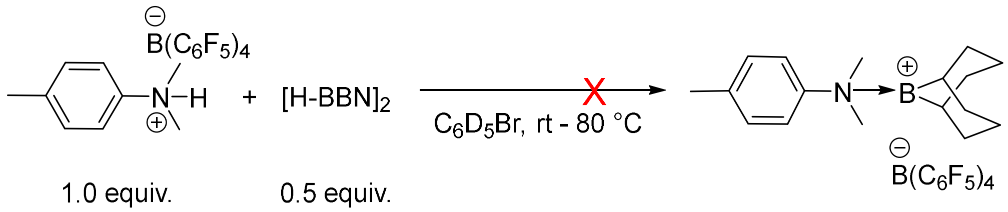

In a glovebox, [H−BBN]$_2$ (3.0 mg, 0.012 mmol, 0.5 equiv.) and [(DMT)H][B(C_6F_5)$_4$] (20.0 mg, 0.024 mmol) charged in a J. Young's NMR tube were dissolved in C_6D_5Br (0.6 mL) at room temperature, which was sealed and mixed well by rotation (ca. 30 rpm) for 20 h. The reaction mixture was further heated at 80 ºC for 20 h, however, no reaction was observed when monitored by ^1H, $^{13}C\{^1H\}$, ^{11}B and ^{19}F NMR spectroscopy. Note, formation of O(BBN)$_2$ was attributed due to the presence of trace moisture in solvent.

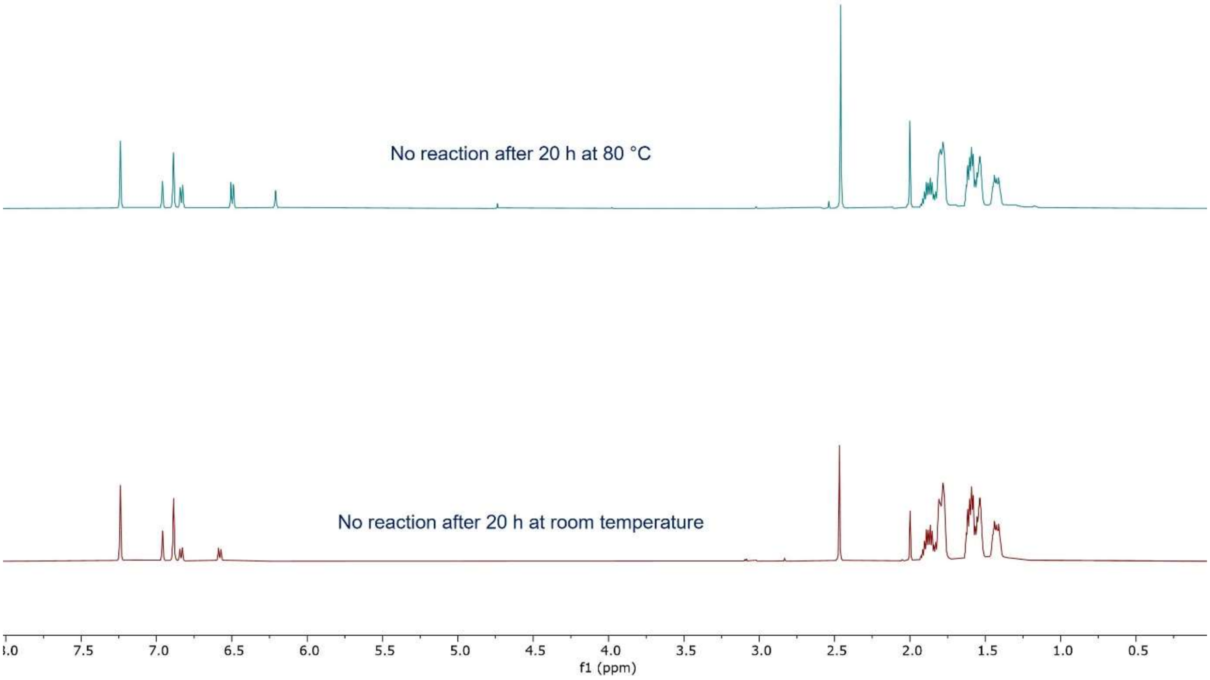

Figure S83: Reaction of [H−BBN]$_2$ and [(DMT)H][B(C_6F_5)$_4$] at room temperature (bottom) and at 80 °C (top) in C_6D_5Br solvent as observed by ^1H NMR spectroscopy.

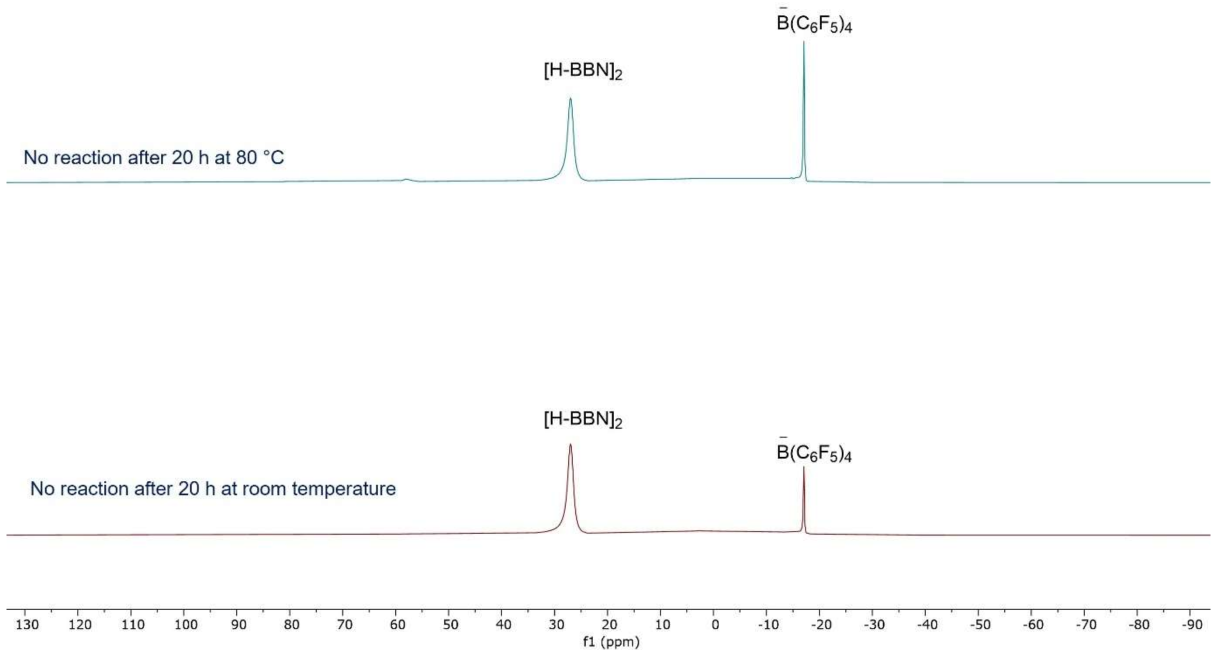

Figure S84: Reaction of $[H{-}BBN]_2$ and $[(DMT)H][B(C_6F_5)_4]$ at room temperature (bottom) and at 80 °C (top) in C_6D_5Br solvent as observed by ^{11}B NMR spectroscopy.

S5.3. No observable reaction between DMT and $[H{-}BBN]_2$

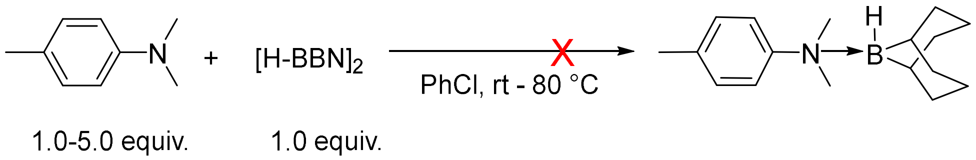

In a glovebox, $[H{-}BBN]_2$ (43.0 mg, 0.17 mmol, 1.0 equiv.) and DMT (25 µL, 0.17 mmol, 1.0 equiv.) charged in a J. Young's NMR tube were dissolved in PhCl (0.6 mL) at room temperature, which was sealed and mixed well by rotation (ca. 30 rpm). Further DMT (100 µL, 0.69 mmol, 4.0 equiv.) was added. Reaction mixture was heated at 80 ºC for 1.5 h, however, no reaction was observed as monitored by ^{1}H, $^{13}C\{^{1}H\}$, ^{11}B and ^{19}F NMR spectroscopy. Note, formation of $O(BBN)_2$ was attributed due to the presence of trace moisture in DMT.

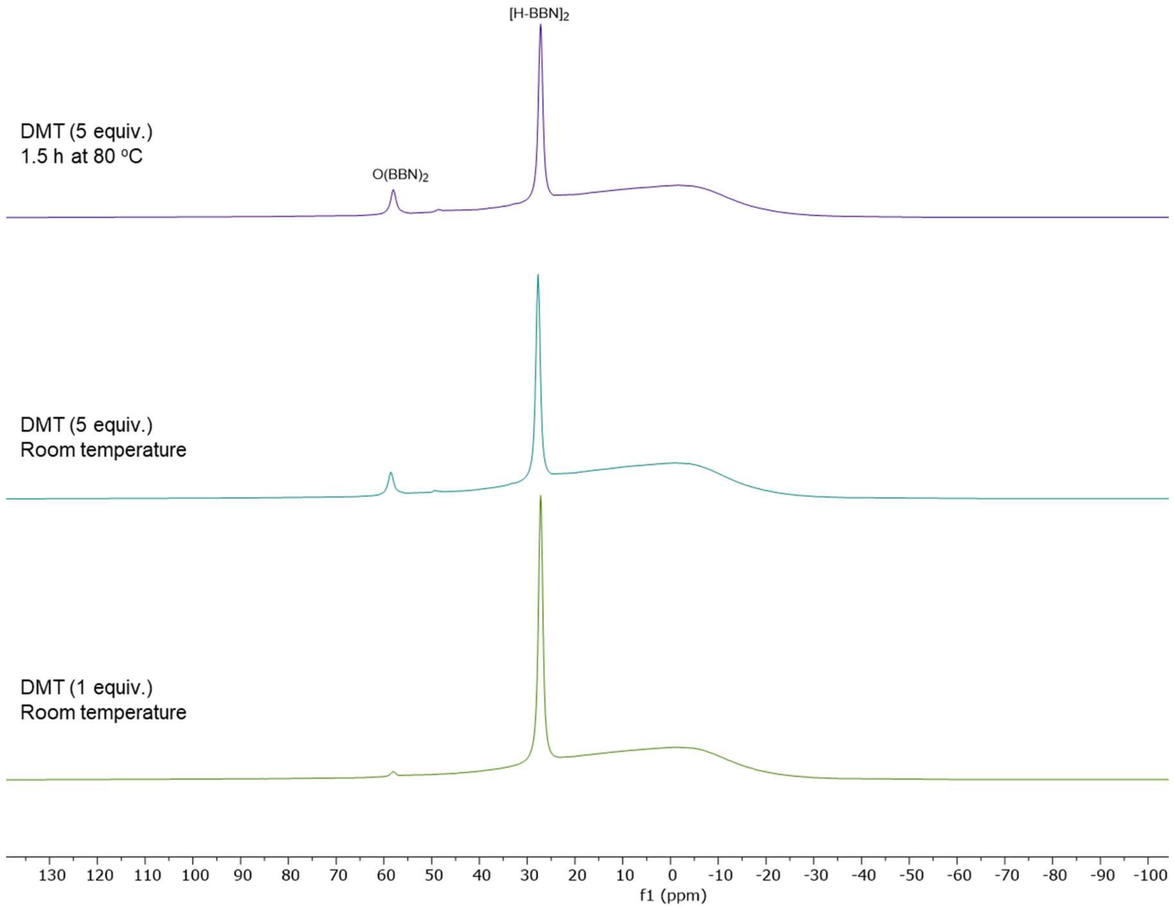

Figure S85: Reaction of [H−BBN]$_2$ and DMT (1 equiv.) at room temperature (green), reaction of [H−BBN]$_2$ and DMT (5 equiv.) at room temperature (blue) and reaction of [H−BBN]$_2$ and DMT (5 equiv.) at 80 °C (purple) in PhCl solvent as observed by ^{11}B NMR spectroscopy.

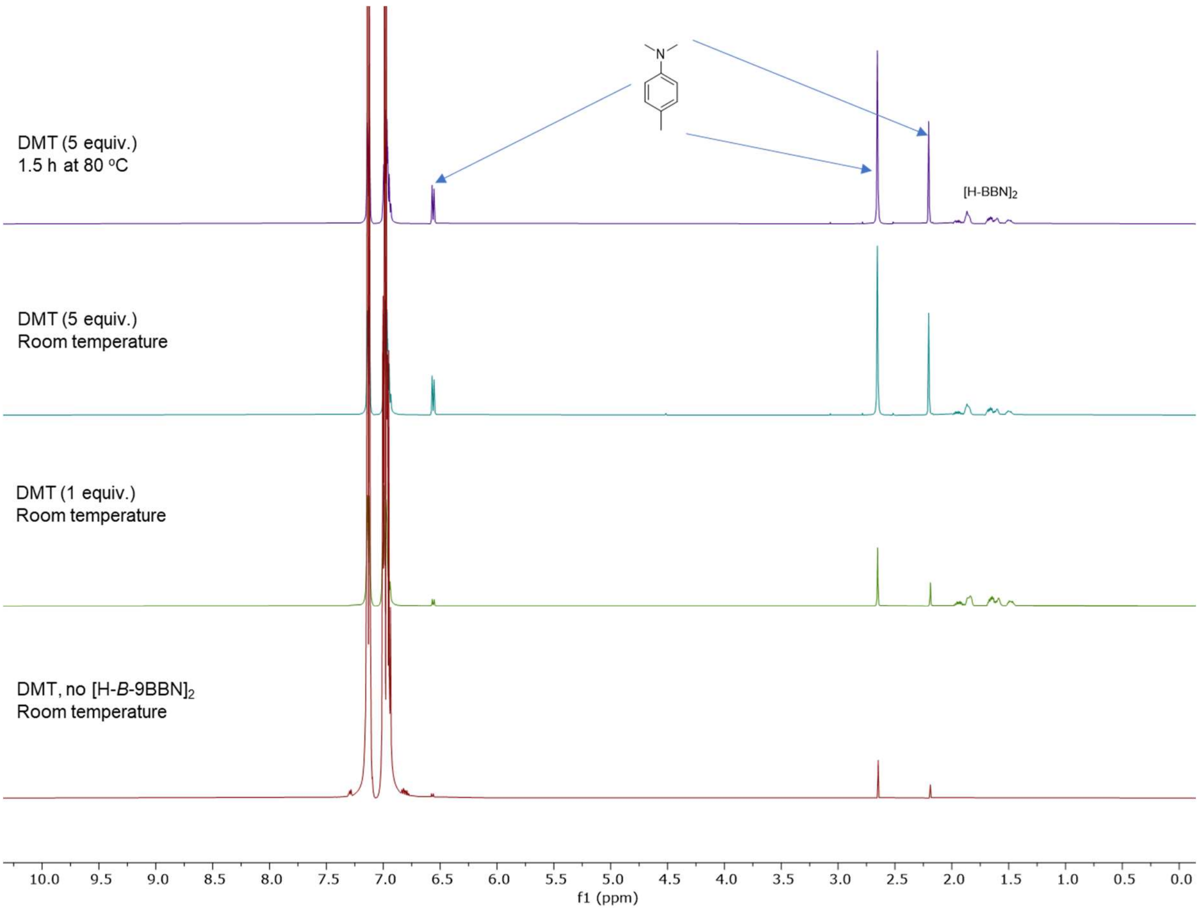

Figure S86: Free DMT (no [H−BBN]$_2$) (red), reaction of [H−BBN]$_2$ and DMT (1 equiv.) at room temperature (green), reaction of [H−BBN]$_2$ and DMT (5 equiv.) at room temperature (blue) and reaction of [H−BBN]$_2$ and DMT (5 equiv.) at 80 °C (purple) in PhCl solvent as observed by ^{11}B NMR spectroscopy.

S5.4. σ-bond metathesis between ^{Dipp}NacNacZn-thienyl and [H−BBN]$_2$

<u>Reaction with 0.5 equiv. [H−BBN]$_2$</u>

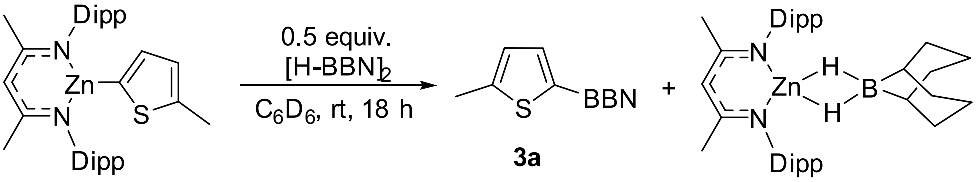

10 **3a** **11**

In a glovebox, ^{Dipp}NacNacZn-thienyl (10.0 mg, 0.017 mmol) and [H−BBN]$_2$ (2.1 mg, 0.017 mmol) charged in a J. Young's NMR tube were dissolved in C_6D_6 (0.6 mL) at room temperature, which was sealed and mixed well by rotation (ca. 30 rpm) for ca. 18 h. Upon completion the reaction mixture was monitored by ^{1}H, ^{13}C{^{1}H}, and ^{11}B NMR spectroscopy,

which suggest ca. 47% formation of **11**/**3a** along with 45% unreacted NacNacZn-thienyl (**10**) starting compound and 8% NacNacH in the reaction mixture.

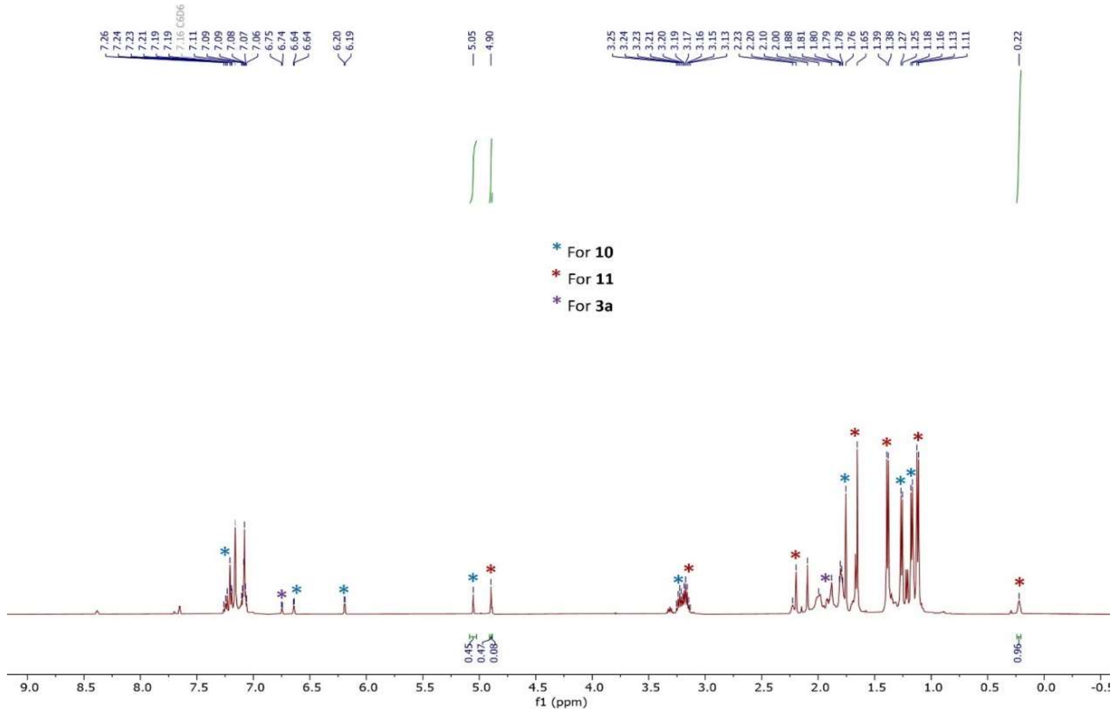

Figure S87: Reaction of ^{Dipp}NacNacZn-thienyl and $[H{-}BBN]_2$ (0.5 equiv.) at room temperature for 18 h in C_6D_6 solvent as observed by ^{1}H NMR spectroscopy.

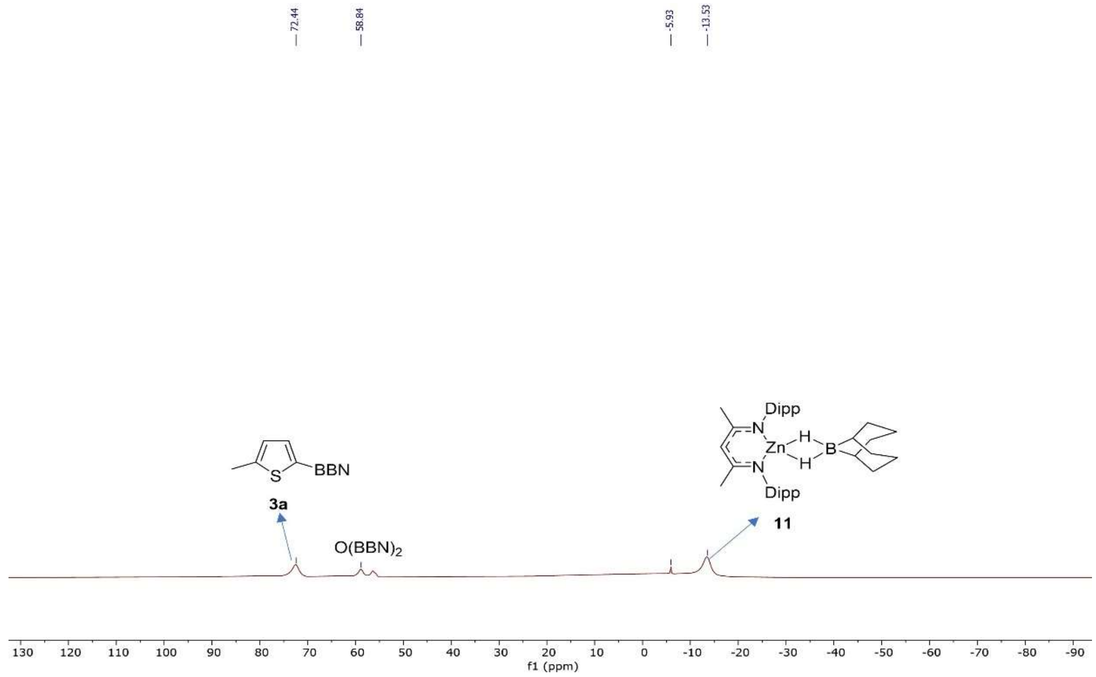

Figure S88: Reaction of ^{Dipp}NacNacZn-thienyl and $[H{-}BBN]_2$ (0.5 equiv.) at room temperature for 18 h in C_6D_6 solvent as observed by ^{11}B NMR spectroscopy.

Reaction with 1.0 equiv. [H−BBN]$_2$

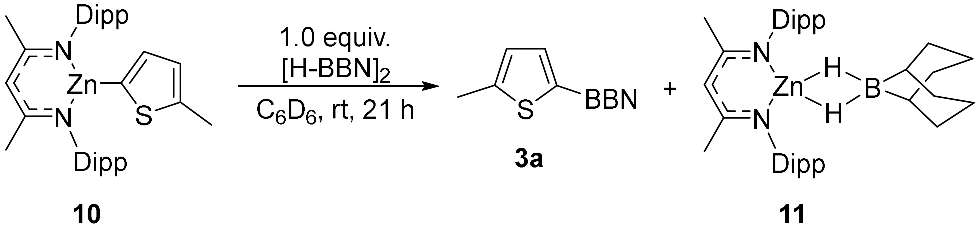

In a glovebox, ^{Dipp}NacNacZn-thienyl (11.9 mg, 0.020 mmol) and [H−BBN]$_2$ (5.0 mg, 0.020 mmol) charged in a J. Young's NMR tube were dissolved in C_6D_6 (0.6 mL) at room temperature, which was sealed and mixed well by rotation (ca. 30 rpm) for ca. 21 h. Upon completion the reaction mixture was monitored by ^{1}H, ^{13}C{^{1}H}, and ^{11}B NMR spectroscopy, which suggest complete consumption of ^{Dipp}NacNacZn-thienyl and almost quantitative formation of **3a** and **11**.

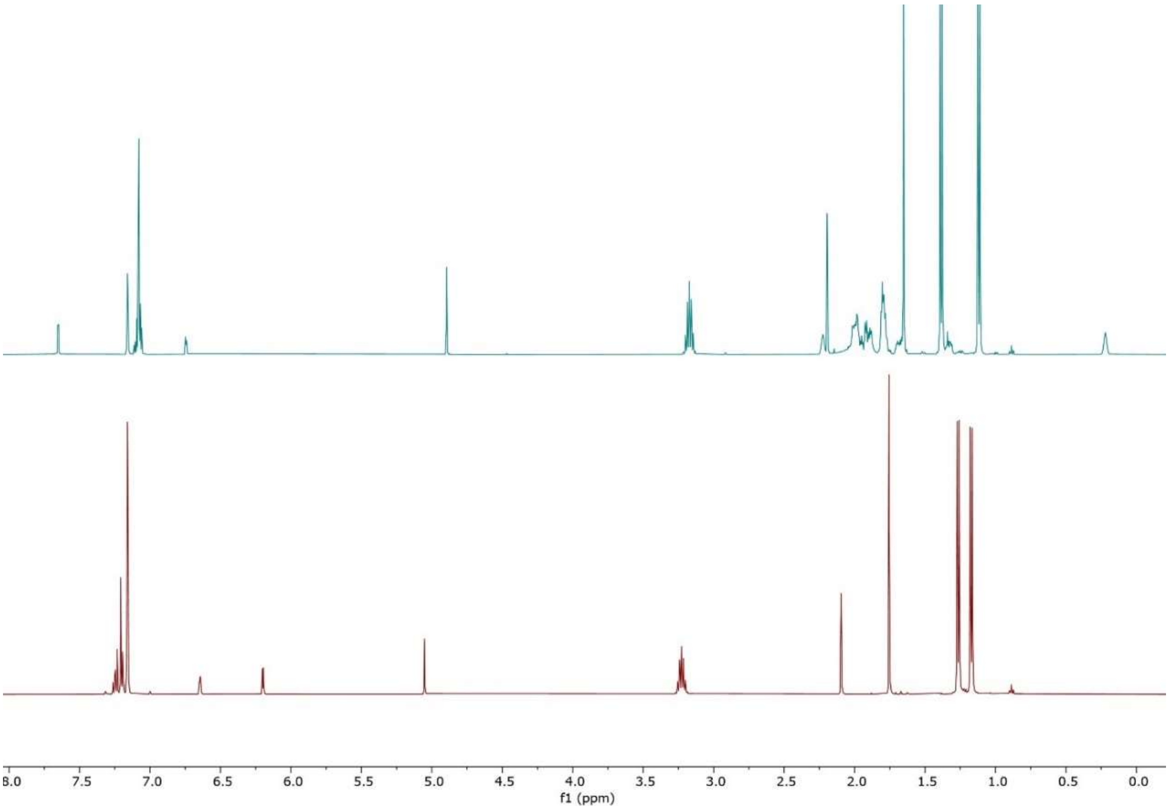

Figure S89: (Bottom NMR): ^{Dipp}NacNacZn-thienyl in C_6D_6 solvent as observed by ^{1}H NMR spectroscopy. (Top NMR): After addition of [H−BBN]$_2$ to the reaction mixture in C_6D_6 solvent as observed by ^{1}H NMR spectroscopy after 21 h.

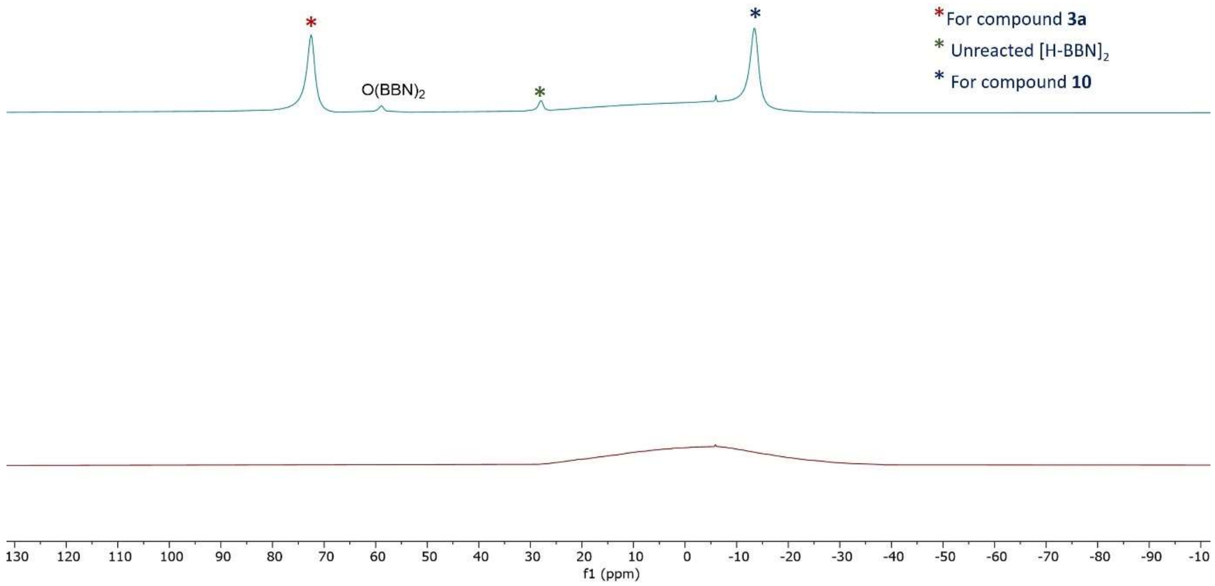

Figure S90: (Bottom NMR): DippNacNacZn-thienyl in C_6D_6 solvent as observed by ^{11}B NMR spectroscopy. (Top NMR): After addition of $[H{-}BBN]_2$ to the reaction mixture in C_6D_6 solvent as observed by ^{11}B NMR spectroscopy after 21 h.

S5.5. Reaction of DippNacNacZn–H and $[H{-}BBN]_2$

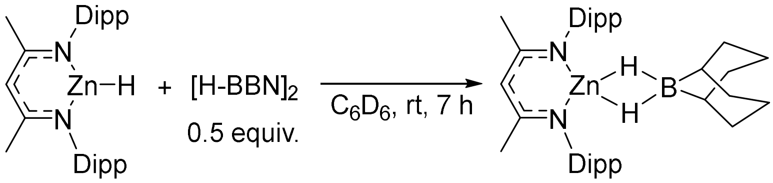

11

In a glovebox, DippNacNacZnH (20.0 mg, 0.041 mmol) and $[H{-}BBN]_2$ (5.0 mg, 0.041 mmol) charged in a J. Young's NMR tube were dissolved in C_6D_6 (0.6 mL) at room temperature, which was sealed and mixed well by rotation (ca. 30 rpm) for ca. 7 h. The reaction mixture was monitored by ^{1}H, $^{13}C\{^{1}H\}$, and ^{11}B NMR spectroscopy and ca. 92% conversion to the product was determined. Single colourless block-shaped crystals of **11** was obtained after slow evaporation of C_6D_6.

Compound 11

^{1}H NMR (500 MHz, C_6D_6): *δ* 7.11-7.06 (m, 6H, *Ar*), 4.90 (s, 1H, γ-C*H*), 3.17 (sept, *J* = 6.9 Hz, 4H, C*H*Me_2), 2.06-1.89 (m, 4H, BBN), 1.81-1.73 (m, 8H, BBN), 1.71-1.65 (m, 2H, BBN), 1.65 (s, 6H, ^{BDI}CH_3), 1.38 (d, *J* = 6.9 Hz, 12H, CH*Me*$_2$), 1.12 (d, *J* = 6.9 Hz, 12H, CH*Me*$_2$), 0.22 (s, 2H, 3c-2e *H*).

^{13}C{^{1}H} NMR (126 MHz, C_6D_6): *δ* 169.6 (s, *C*CH*C*), 143.2 (*Ar*), 141.8 (*Ar*), 126.8 (*Ar*), 124.2 (*Ar*), 94.9 (C*C*HC), 34.8 (BBN), 28.6 (*C*HMe$_2$), 25.1 (BBN), 24.3 (CH*Me*$_2$), 24.2 (CH*Me*$_2$), 23.2 (^{Me}BDI), 19.7 (BBN).

^{11}B NMR (160 MHz, C_6D_6): *δ* -13.5 (s).

Note, several attempts were made to perform mass spectrometry on these compound, but these all did not show the $[M]^+$ or $[M+H]^+$.

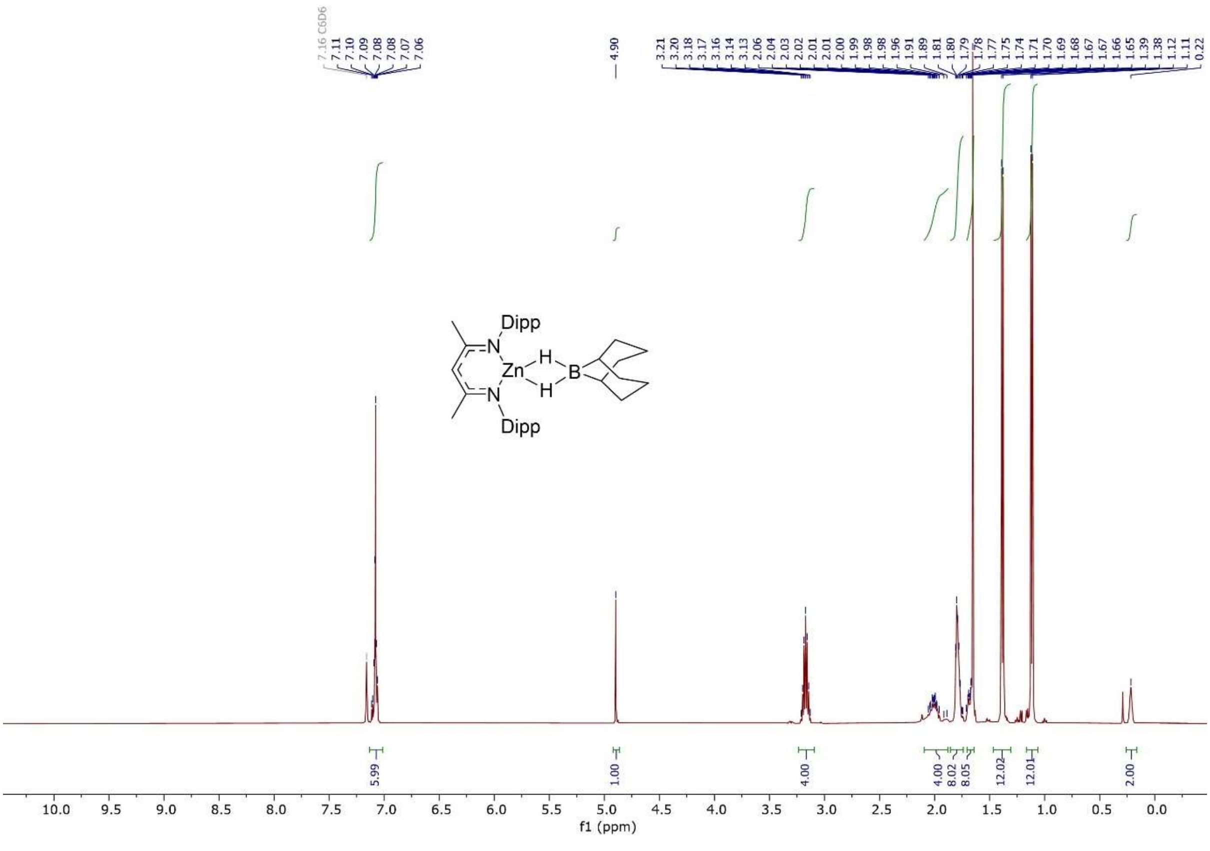

Figure S91: ^{1}H NMR spectrum of compound **11** in C_6D_6.

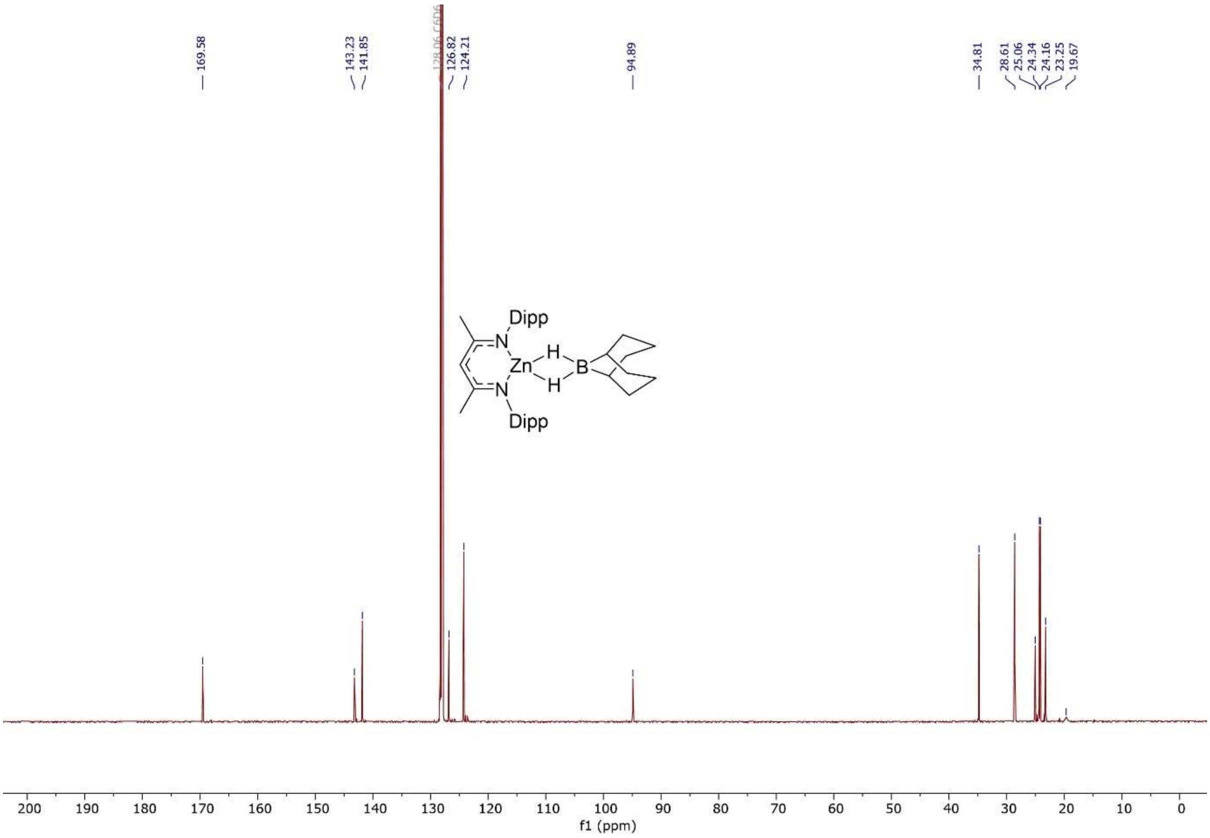

Figure S92: $^{13}C\{^{1}H\}$ NMR spectrum of compound **11** in C_6D_6.

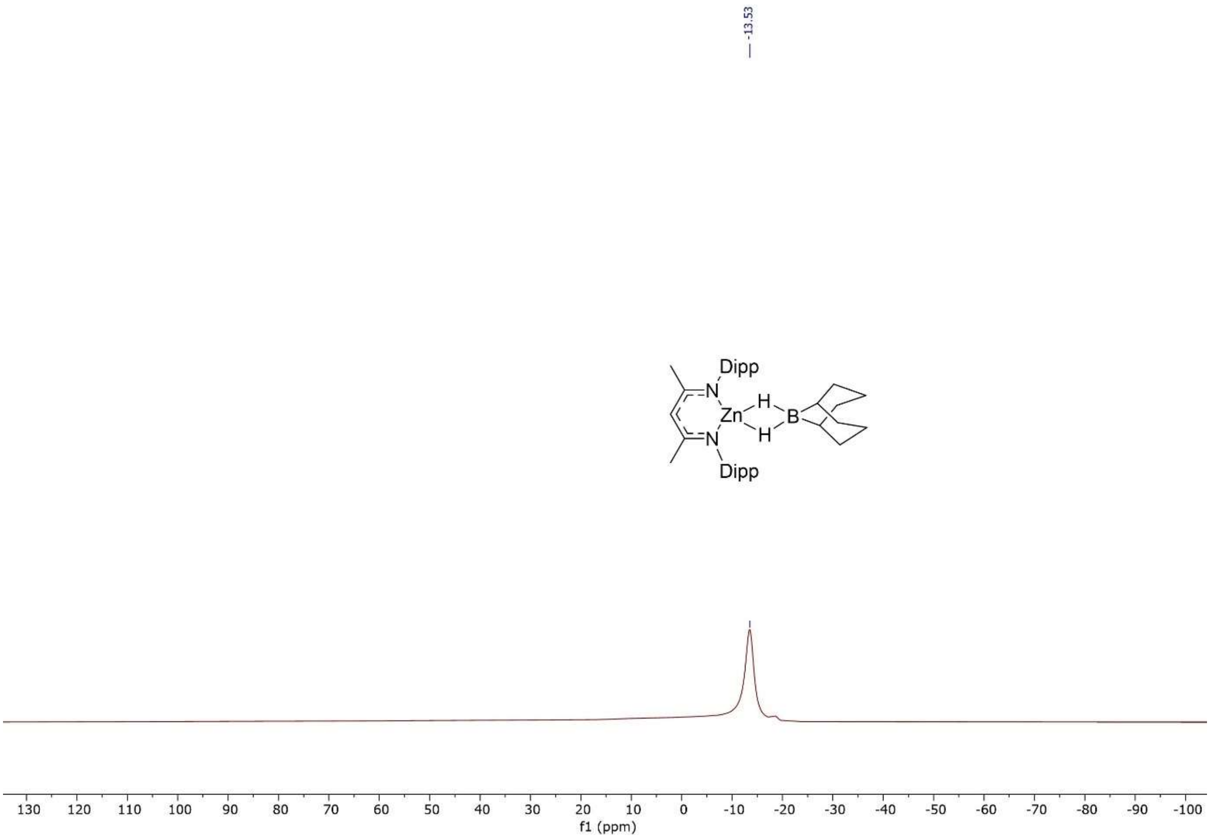

Figure S93: ^{11}B NMR spectrum of compound **11** in C_6D_6.

S5.6. Reaction of compound **11** with [(DMT)H][B(C_6F_5)$_4$]

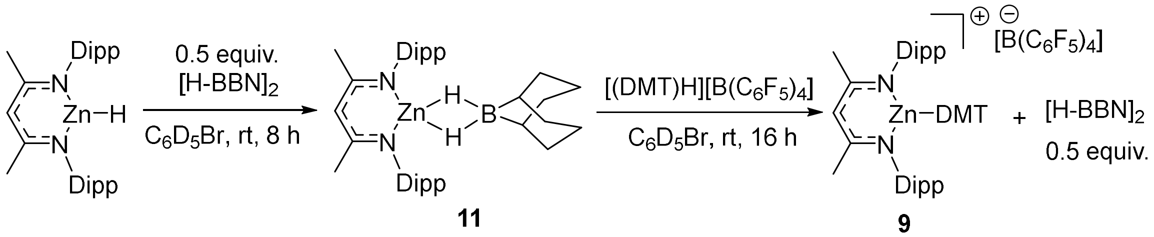

In a glovebox, ^{Dipp}NacNacZnH (12.0 mg, 0.024 mmol) and [H−BBN]$_2$ (3.0 mg, 0.012 mmol) charged in a J. Young's NMR tube were dissolved in C_6D_5Br (0.6 mL) at room temperature, which was sealed and mixed well by rotation (ca. 30 rpm) for ca. 8 h. The reaction mixture was monitored by ^{1}H, ^{13}C{^{1}H}, and ^{11}B NMR spectroscopy, which indicated formation of **11** (>95%). [(DMT)H][B(C_6F_5)$_4$] (20.0 mg, 0.024 mmol) was then added to the reaction mixture and mixed well by rotation (ca. 30 rpm) for ca. 16 h. After that the reaction mixture was monitored by ^{1}H, ^{13}C{^{1}H}, ^{19}F and ^{11}B NMR spectroscopy, which indicated the formation of [^{Dipp}NacNacZn-DMT][B(C_6F_5)$_4$] (**9**) and [H−BBN]$_2$ and consumption of compound **11**.

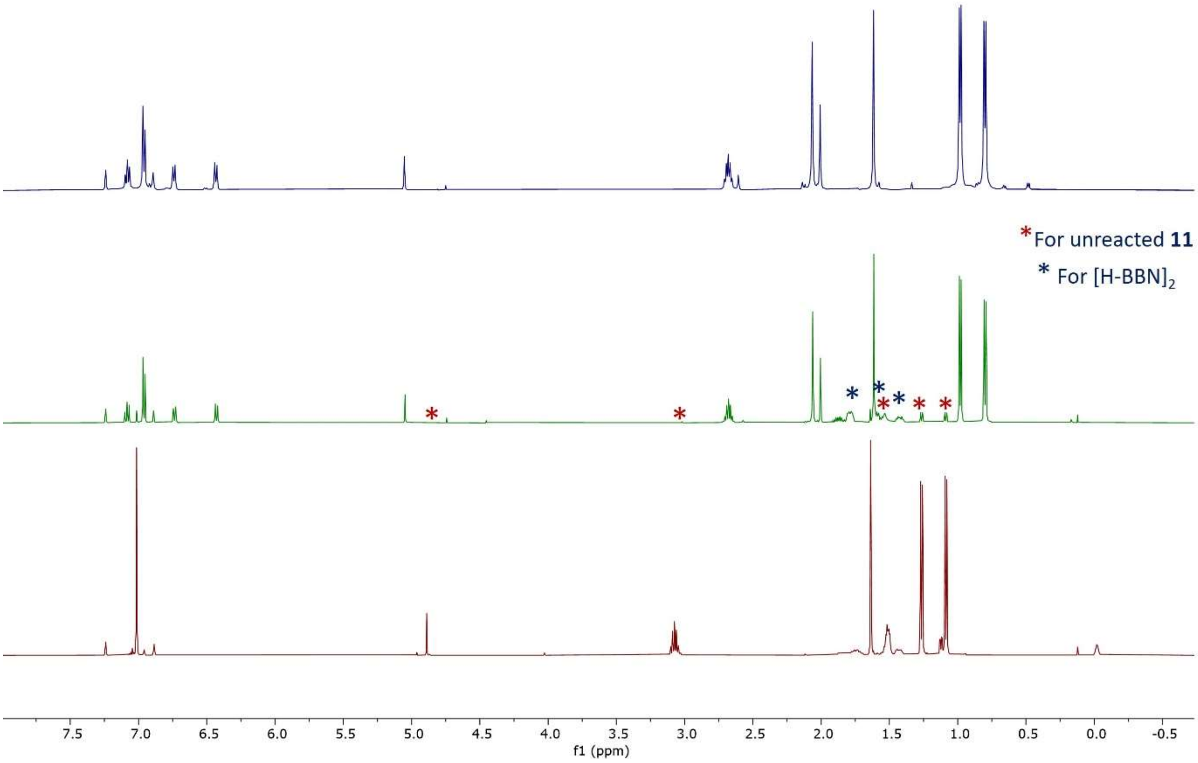

Figure S94: (Bottom NMR): Reaction of [H−BBN]$_2$ and ^{Dipp}NacNacZnH at room temperature for 8 h in C_6D_5Br solvent as observed by ^{1}H NMR spectroscopy. (Middle NMR): After addition of [(DMT)H][B(C_6F_5)$_4$] to the reaction mixture in C_6D_5Br solvent as observed by ^{1}H NMR

spectroscopy after 16 h. (Top NMR): ^{1}H NMR spectroscopy of the [^{Dipp}NacNacZn-DMT][$B(C_6F_5)_4$] (prepared separately following literature[5]) in C_6D_5Br solvent for comparison.

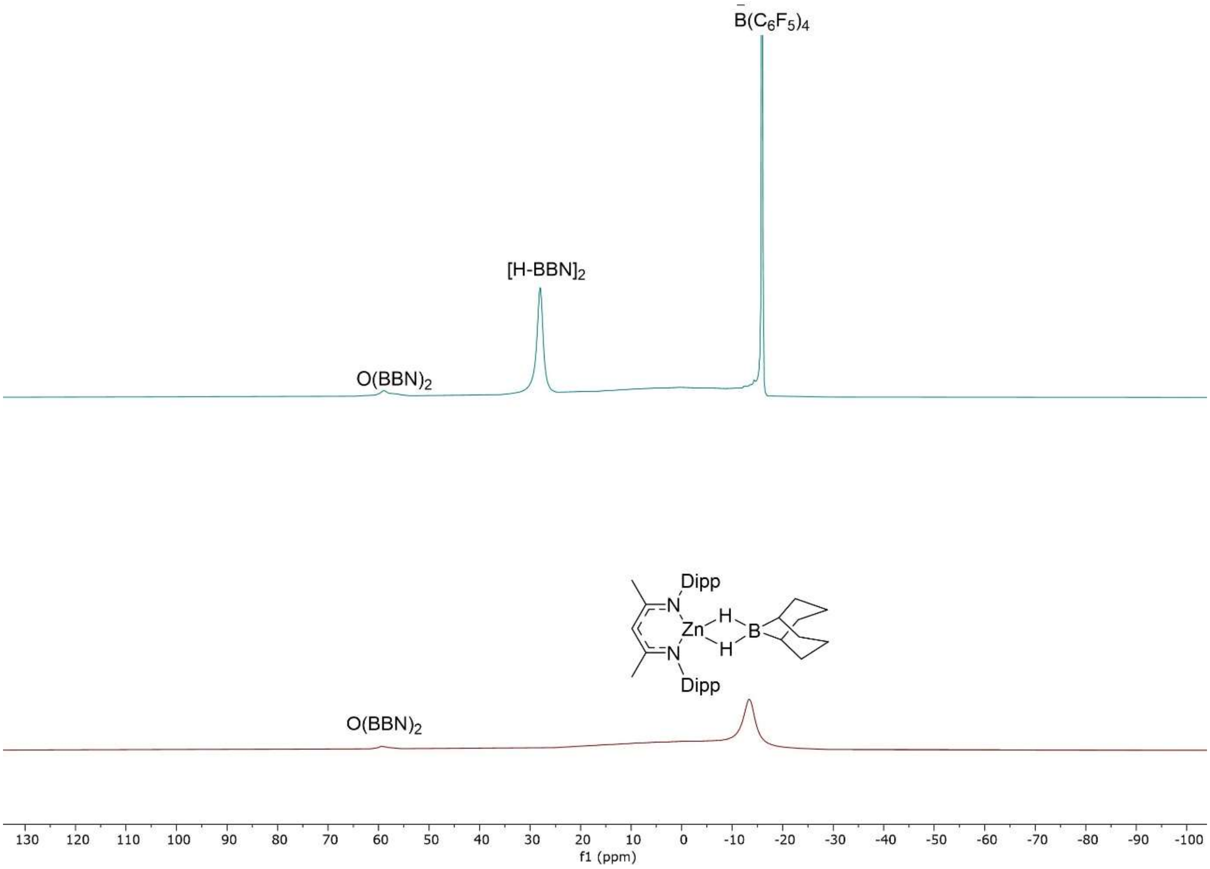

Figure S95: (Bottom NMR): Reaction of [H−BBN]$_2$ and ^{Dipp}NacNacZnH at room temperature for 8 h in C_6D_5Br solvent as observed by ^{11}B NMR spectroscopy. (Top NMR): After addition of [(DMT)H][$B(C_6F_5)_4$] to the reaction mixture in C_6D_5Br solvent as observed by ^{11}B NMR spectroscopy after 16 h.

S5.7. Reaction of compound **11** with [$(Et_3N)H$][B{$C_6H_3(CF_3)_2$}$_4$]

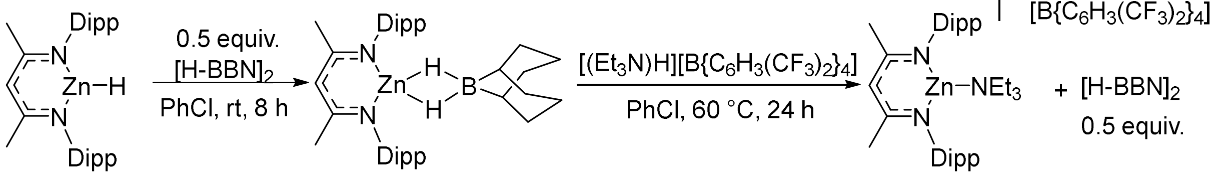

11

In a glovebox, ^{Dipp}NacNacZnH (5.0 mg, 0.010 mmol) and [H−BBN]$_2$ (1.3 mg, 0.005 mmol) charged in a J. Young's NMR tube were dissolved in PhCl (0.6 mL) at room temperature, which was sealed and mixed well by rotation (ca. 30 rpm) for ca. 8 h. The reaction mixture was monitored by ^{1}H, $^{13}C\{^{1}H\}$, and ^{11}B NMR spectroscopy, which indicated formation of **11**

(>95%). $[(Et_3N)H][B\{C_6H_3(CF_3)_2\}_4]$ (10.0 mg, 0.010 mmol) was then added to the reaction mixture and mixed well by rotation (ca. 30 rpm) for ca. 3 h at room temperature and then heated at 60 ºC for 24 h. The reaction mixture was monitored by ^{1}H, $^{13}C\{^{1}H\}$, ^{19}F and ^{11}B NMR spectroscopy, which indicated no reaction at room temperature after 3 h, but 59% conversion to $[^{Dipp}NacNacZn\text{-}NEt_3][B(C_6F_5)_4]$ and $[H{-}BBN]_2$ after heating for 24 h at 60 °C.

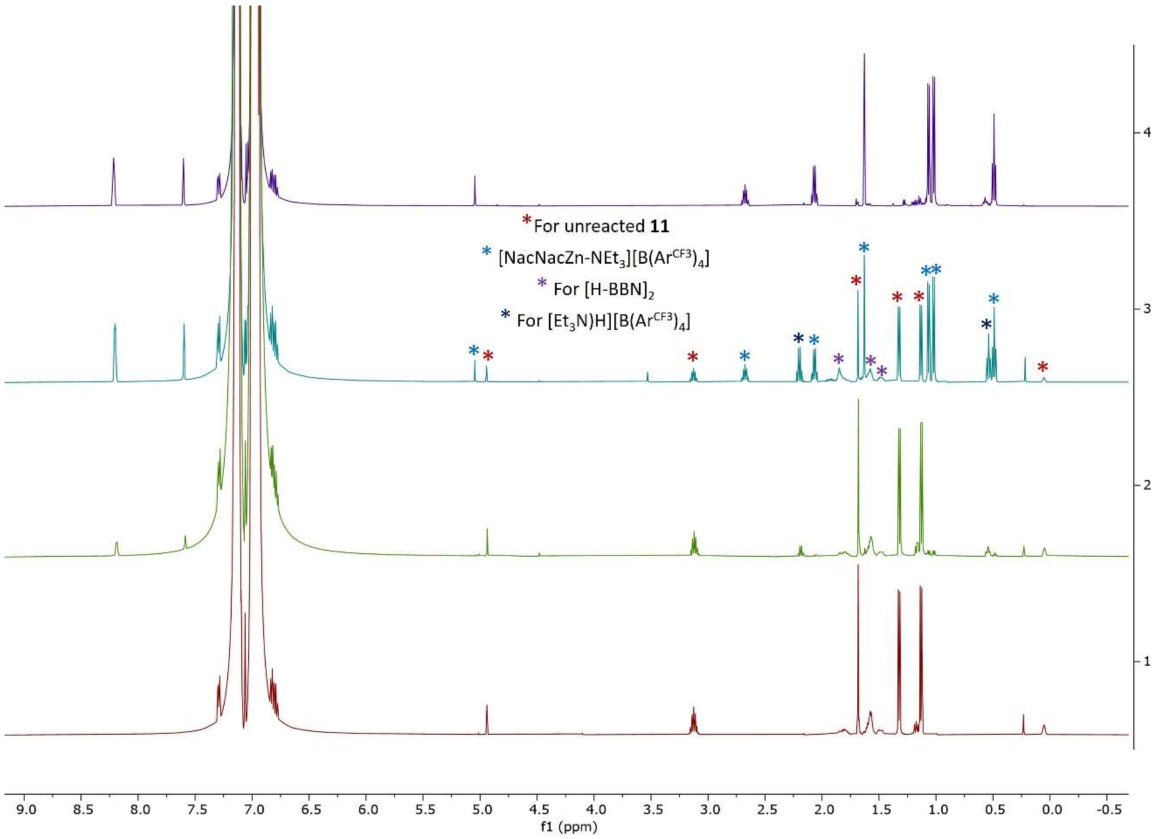

Figure S96: (NMR 1): Reaction of $[H{-}BBN]_2$ and $^{Dipp}NacNacZnH$ at room temperature for 8 h in PhCl solvent as observed by ^{1}H NMR spectroscopy. (NMR 2): After addition of $[(Et_3N)H][B\{C_6H_3(CF_3)_2\}_4]$ to the reaction mixture in PhCl solvent as observed by ^{1}H NMR spectroscopy after 3 h reaction at room temperature. (NMR 3): The reaction mixture in PhCl solvent as observed by ^{1}H NMR spectroscopy after 24 h heating at 60 ºC. (NMR 4): ^{1}H NMR spectroscopy of the $[^{Dipp}NacNacZn\text{-}NEt_3][B\{C_6H_3(CF_3)_2\}_4]$ (prepared separately following literature[5]) in PhCl solvent for comparison.

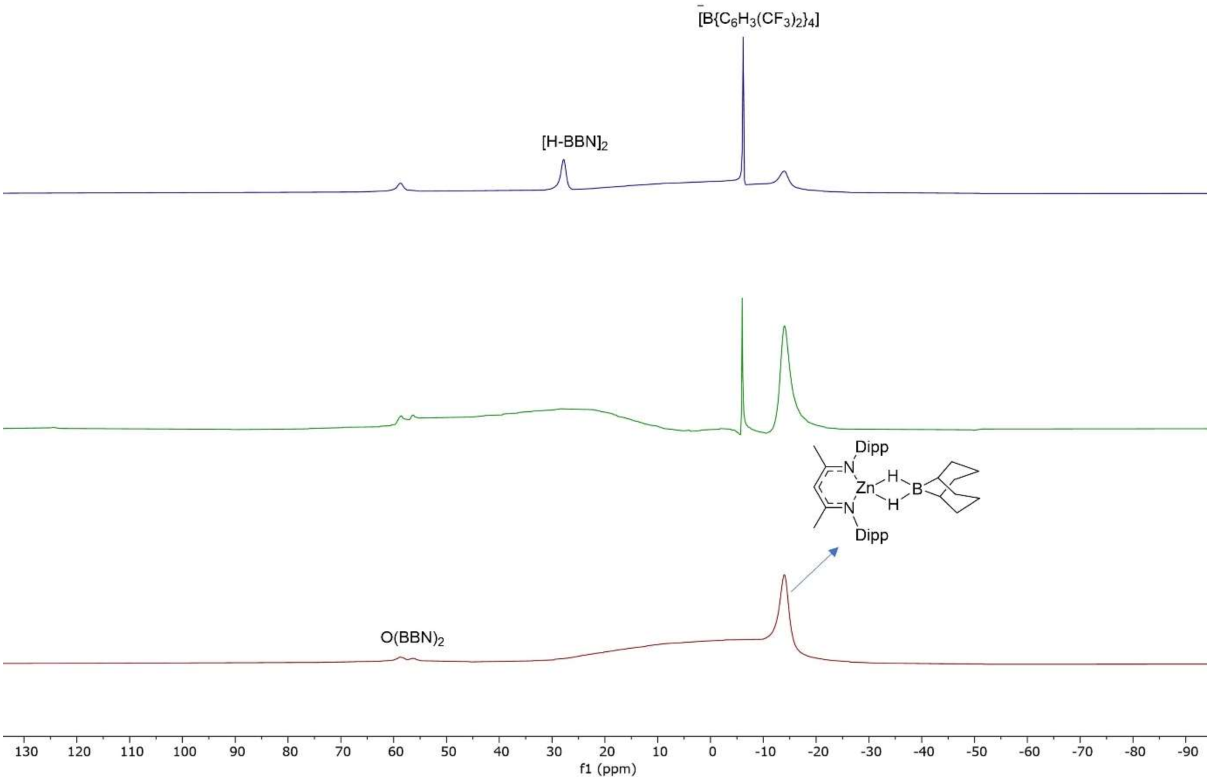

Figure S97: (Bottom NMR): Reaction of [H−BBN]$_2$ and ^{Dipp}NacNacZnH at room temperature for 8 h in PhCl solvent as observed by ^{11}B NMR spectroscopy. (Middle NMR): After addition of [(Et$_3$N)H][B{C$_6$H$_3$(CF$_3$)$_2$}$_4$] to the reaction mixture in PhCl solvent as observed by ^{11}B NMR spectroscopy after 3 h reaction at room temperature. (Top NMR): The reaction mixture in PhCl solvent as observed by ^{11}B NMR spectroscopy after 24 h reaction at 60 ºC.

S5.8. Reaction of compound **11** with [(Et$_3$N)H][B(C$_6$F$_5$)$_4$]

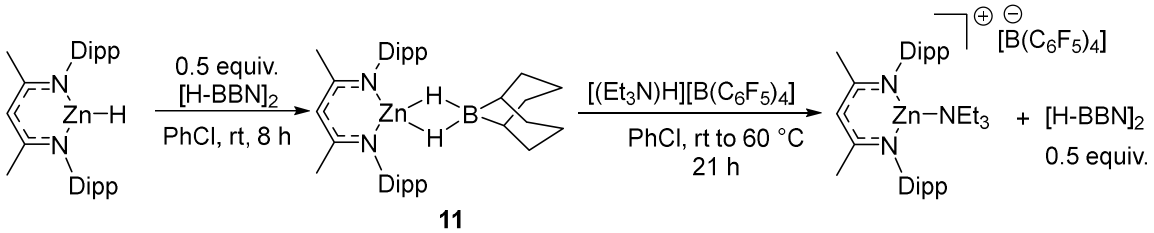

In a glovebox, ^{Dipp}NacNacZnH (10.0 mg, 0.020 mmol) and [H−BBN]$_2$ (2.6 mg, 0.010 mmol) charged in a J. Young's NMR tube were dissolved in PhCl (0.6 mL) at room temperature, which was sealed and mixed well by rotation (ca. 30 rpm) for ca. 8 h. The reaction mixture was monitored by ^{1}H, ^{13}C{^{1}H}, and ^{11}B NMR spectroscopy, which indicated formation of **11** (>95%). [(Et$_3$N)H][B(C$_6$F$_5$)$_4$] (16.2 mg, 0.020 mmol) was then added to the reaction mixture and mixed well by rotation (ca. 30 rpm) for ca. 21 h at room temperature and then heated at 60

ºC for 24 h. The reaction mixture was monitored by ^{1}H, $^{13}C\{^{1}H\}$, ^{19}F and ^{11}B NMR spectroscopy, which indicated ca. 10% (after 21 h, at room temperature) and total 43% (after another 18 h at 60 °C) conversion to [^{Dipp}NacNacZn-NEt$_3$][B(C$_6$F$_5$)$_4$] and [H−BBN]$_2$.

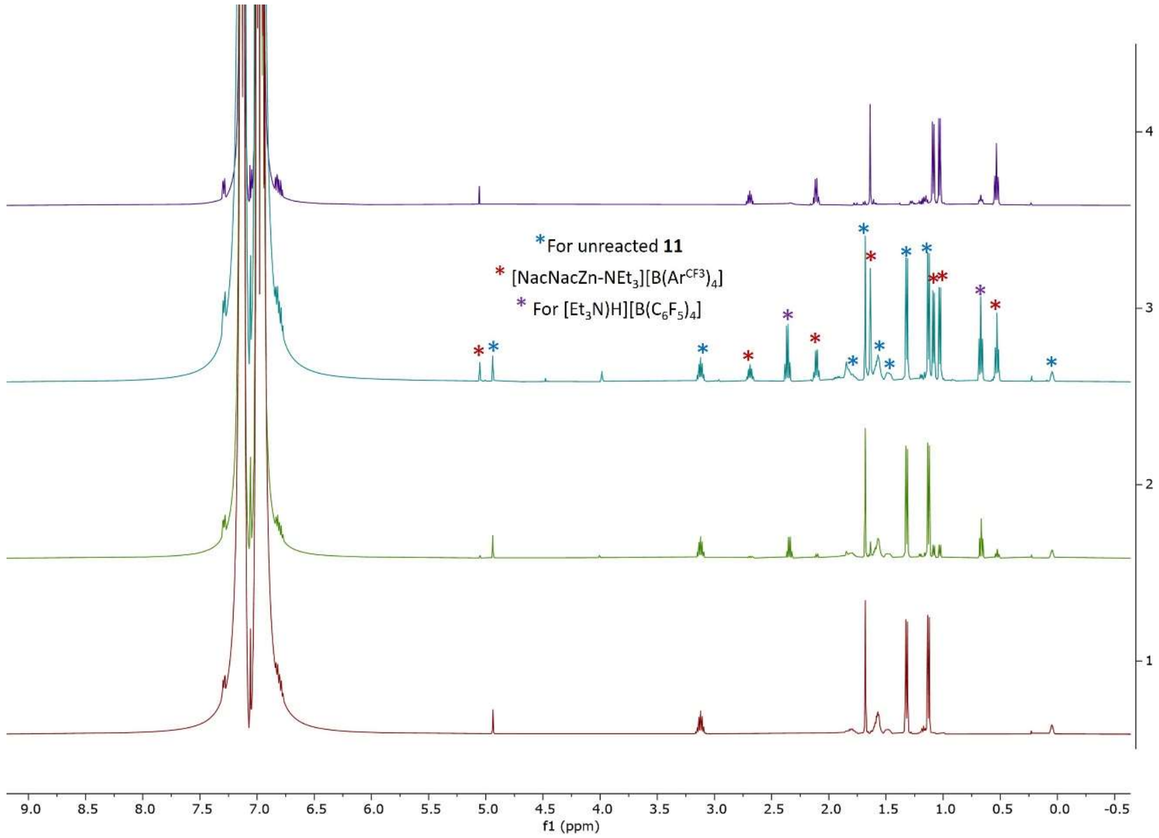

Figure S98: (NMR 1): Reaction of [H−BBN]$_2$ and ^{Dipp}NacNacZnH at room temperature for 8 h in PhCl solvent as observed by ^{1}H NMR spectroscopy. (NMR 2): After addition of [(Et$_3$N)H][B(C$_6$F$_5$)$_4$] to the reaction mixture in PhCl solvent as observed by ^{1}H NMR spectroscopy after 21 h reaction at room temperature. (NMR 3): The reaction mixture in PhCl solvent as observed by ^{1}H NMR spectroscopy after 18 h heating at 60 ºC. (NMR 4): ^{1}H NMR spectroscopy of the [^{Dipp}NacNacZn-NEt$_3$][B(C$_6$F$_5$)$_4$] (prepared separately following literature[5]) in PhCl solvent for comparison.

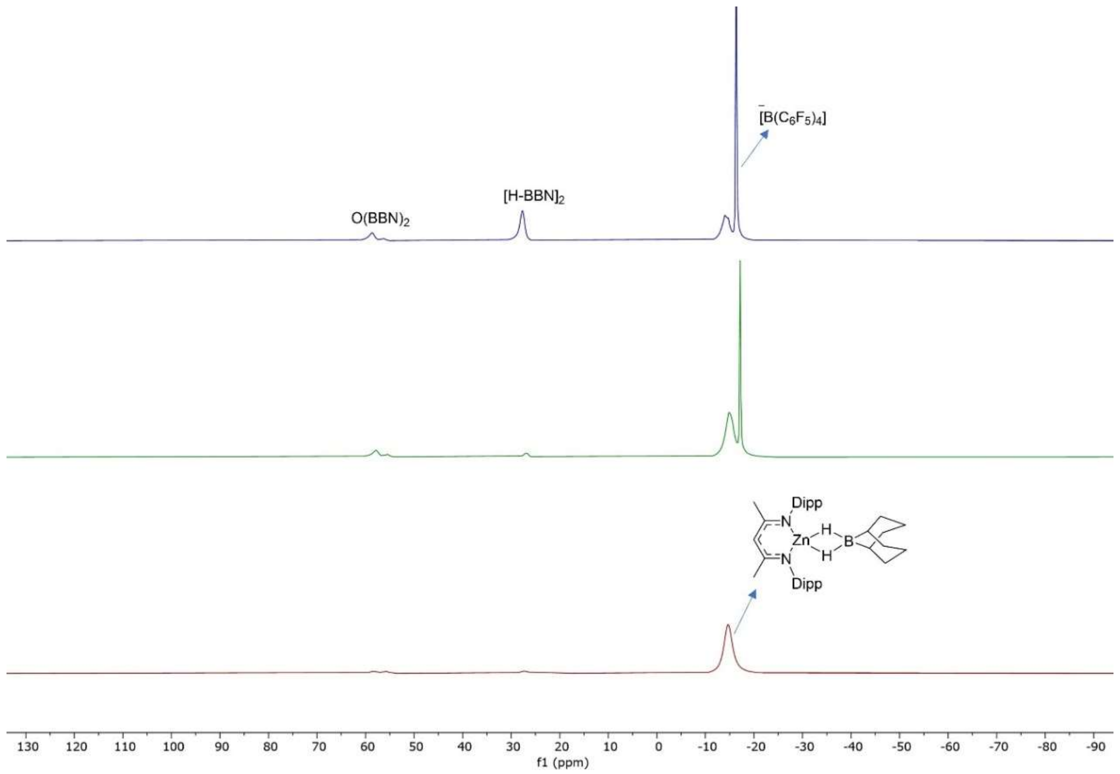

Figure S99: (Bottom NMR): Reaction of [H−BBN]$_2$ and ^{Dipp}NacNacZnH at room temperature for 8 h in PhCl solvent as observed by ^{11}B NMR spectroscopy. (Middle NMR): After addition of [(Et$_3$N)H][B(C$_6$F$_5$)$_4$] to the reaction mixture in PhCl solvent as observed by ^{11}B NMR spectroscopy after 21 h reaction at room temperature. (Top NMR): The reaction mixture in PhCl solvent as observed by ^{11}B NMR spectroscopy after 18 h reaction at 60 ºC.

S5.9. Variable time normalization analysis (VTNA)

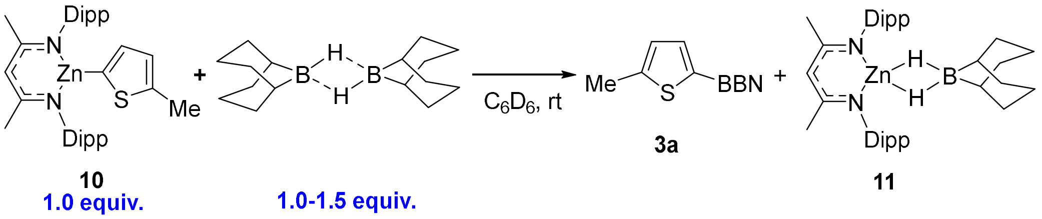

<u>Standard reaction:</u> **10** (12.0 mg, 0.020 mmol, 1.0 equiv.) and [H−BBN]$_2$ (5.0 mg, 0.020 mmol, 1.0 equiv.) charged in a quartz NMR tube were dissolved in C$_6$D$_6$ (0.6 mL) at room temperature, which was sealed and mixed well and immediately introduced in to the Pro500 NMR spectrometer.

<u>Reaction with different [H−BBN]$_2$ concentration:</u> **10** (12.0 mg, 0.020 mmol, 1.0 equiv.) and [H−BBN]$_2$ (7.5 mg, 0.030 mmol, 1.5 equiv.) charged in a quartz NMR tube were dissolved in

C_6D_6 (0.6 mL) at room temperature, which was sealed and mixed well and immediately introduced in to the Pro500 NMR spectrometer.

Following the method described by Burés,[14] the concentration of $[H\text{-}BBN]_2$ was varied while concentration of **10** remained the same. Note, analysis of kinetic profile using ^1H NMR spectroscopy was found to be challenging due to the overlaps of aliphatic proton resonances from unreacted $(H\text{-}BBN)_2$ and products (**3a** and **11**). Hence, ^{11}B NMR spectra were acquired using NS = 512, D1 = 1 s at the interval of 10 min at room temperature. In independent experiments this D1 value was found to be sufficiently long to give accurate integration values and no significant changes were observed in changing D1 from 0.1 to 1.0 s.

The spectra obtained were adequately processed using MestreNova (with subtraction of the probe glass peak, two random ^{11}B spectrum from 1:1 and 1.5 reaction are shown in Figure S102-103 for the clarification) and the kinetic profiles were then plotted on a time-normalised axis. Best overlay of two reaction progress profiles (considering first half of the plot) with orders = 0.9 in $[H{-}BBN]_2$ was observed (Figure S100). Although, the plot with 1.0 order in $[H{-}BBN]_2$ is very close, 0.5 order in $[H{-}BBN]_2$ shows no overlay between two reaction profile. Slight discrepancies from 1.0 order in $[H{-}BBN]_2$ could be due to the experimental and calculation error or mechanistic complexity.

To further support VTNA result and to confirm that $[H\text{-}BBN]_2$ is not off-cycle during the reaction, we calculated initial rates and plotted against the conc. of $[H\text{-}BBN]_2$. Initial rate vs $[H\text{-}BBN]_2$ plot showed a linear corelation passing through the origin (0,0 coordinate) (Figure S101).

1:1 DippNacNacZn-thienyl and $[H\text{-}BBN]_2$ reaction			
Entry	Time (min)	[Product] (mM)	$[H\text{-}BBN]_2$ (mM)
1	0	0	0.0205
2	10	0.002032	0.018468
3	20	0.004484	0.016016
4	30	0.005426	0.015074
5	40	0.007767	0.012733
6	50	0.008581	0.011919
7	60	0.010041	0.010459
8	70	0.011958	0.008542
9	80	0.012429	0.008071
10	90	0.013152	0.007348
11	100	0.014053	0.006447

1:1.5 DippNacNacZn-thienyl and $[H\text{-}BBN]_2$ reaction			
Entry	Time (min)	[Product] (mM)	$[H\text{-}BBN]_2$ (mM)
1	0	0	0.03075
2	10	0.002795	0.027955
3	20	0.00575	0.025
4	30	0.008467	0.022283
5	40	0.010911	0.019839
6	50	0.012872	0.017878
7	60	0.016107	0.014643
8	70	0.016448	0.014302
9	80	0.01825	0.0125
10	90	0.018691	0.012059
11	100	0.018738	0.012012

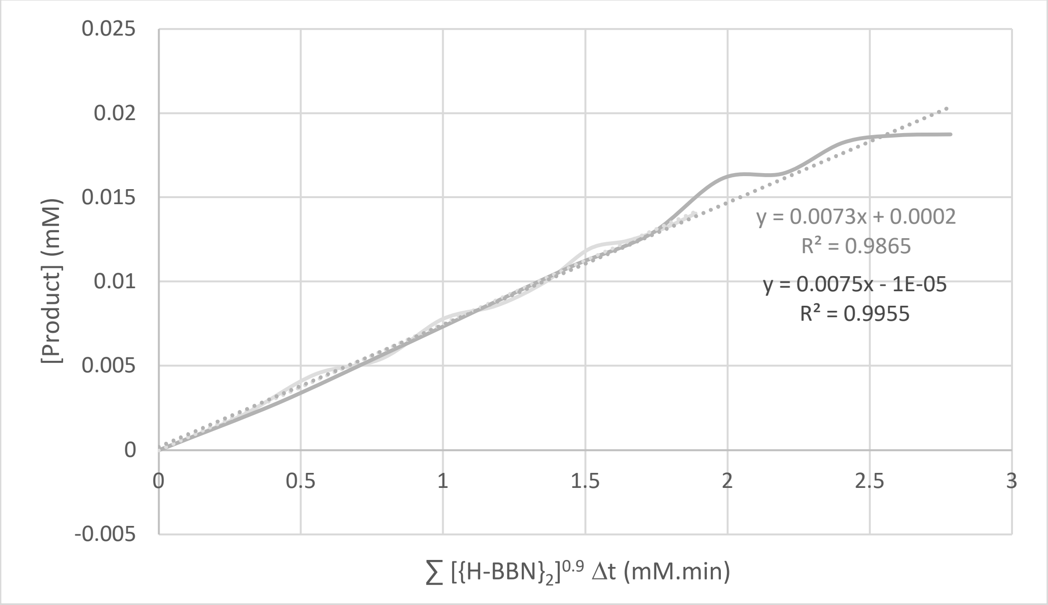

Figure S100: Variable time normalization analysis enables the determination of the order (0.9) in substrate $[H{-}BBN]_2$.

$[Zn] : [H\text{-}BBN]_2$	[Pdt] (mM)	t (min)	Init. rate ($mM.min^{-1}$)	$[H\text{-}BBN]_2$ (mM)
1:0	0	0	0	0
1:1	0.007767	40	0.000194	0.020
1:1.5	0.008467	30	0.000282	0.030

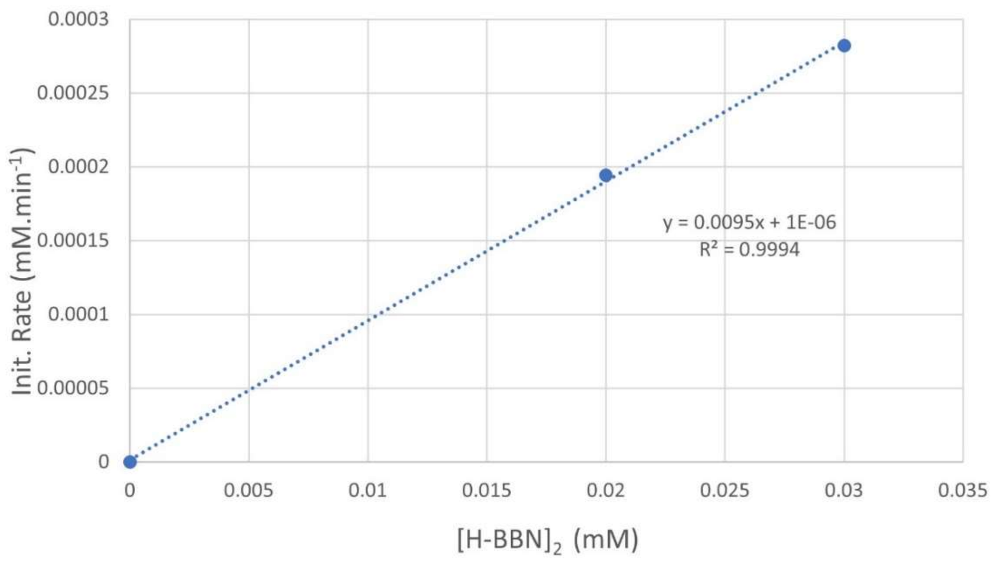

Figure S101: Initial rate vs $[H\text{-}BBN]_2$ plot.

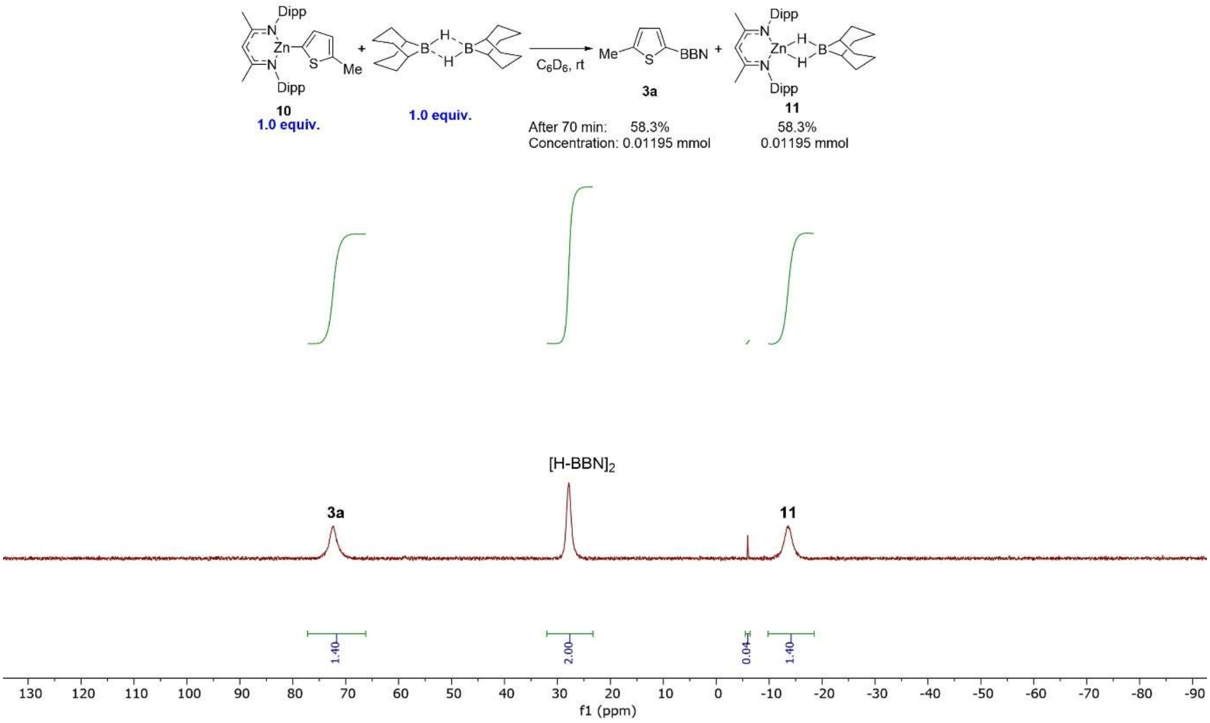

Figure S102: ^{11}B NMR spectrum of 1:1 reaction of **10** and [H-BBN]$_2$ after 70 min in C_6D_6.

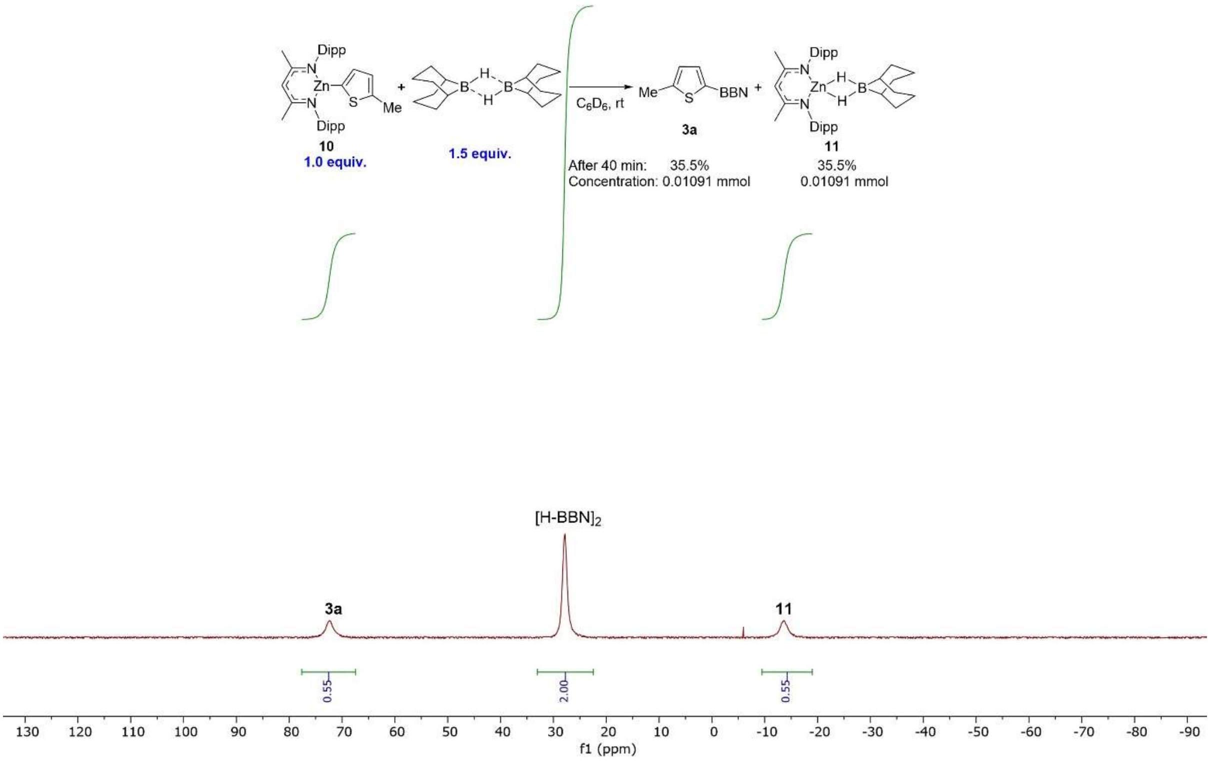

Figure S103: ^{11}B NMR spectrum of 1:1.5 reaction of **10** and [H-BBN]$_2$ after 40 min in C_6D_6.

S5.10. σ-Bond metathesis between DippNacNacZn-thienyl and [H−BBN]$_2$ without or with additives

To disfavour the possibility of hidden catalysis of the sigma bond metathesis step, four separate NMR tube reactions were performed; one reaction without any external additive and the other reactions either with 5 mol% [(DMT)H][B(C_6F_5)$_4$] or DMT or BH_3.SMe_2.

In a glovebox, DippNacNacZn-thienyl (11.9 mg, 0.020 mmol) and [H−BBN]$_2$ (5.0 mg, 0.020 mmol) with (or without) 5 mol% additive charged in a J. Young's NMR tube were dissolved in C_6D_6 (0.6 mL) at room temperature, which was sealed and mixed well. The reaction mixture was monitored by ^{1}H NMR spectroscopy at intervals of 30 min. The spectra obtained were processed using MestreNova and product (%) vs time (min.) plot was obtained which suggest no significant change in the rate of σ-bond metathesis in presence of the three additives used (Figure S104).

Table S3: Product and reactant ratio vs time.

	Without additive		5 mol% [(DMT)H][B(C_6F_5)$_4$]		5 mol% DMT		5 mol% BH_3.SMe_2	
Time (min)	Product (%)	Reactant (%)	Product (%)	Reactant (%)	Product (%)	Reactant (%)	Product (%)	Reactant (%)
0	0	100	0	100	0	100	0	100
30	19	80	22	75	20	76	21	73
60	41	58	39	59	45	62	37	58
90	48	51	53	45	47	49	50	45
120	59	41	66	32	57	39	60	35
150	68	31	75	23	65	31	68	26

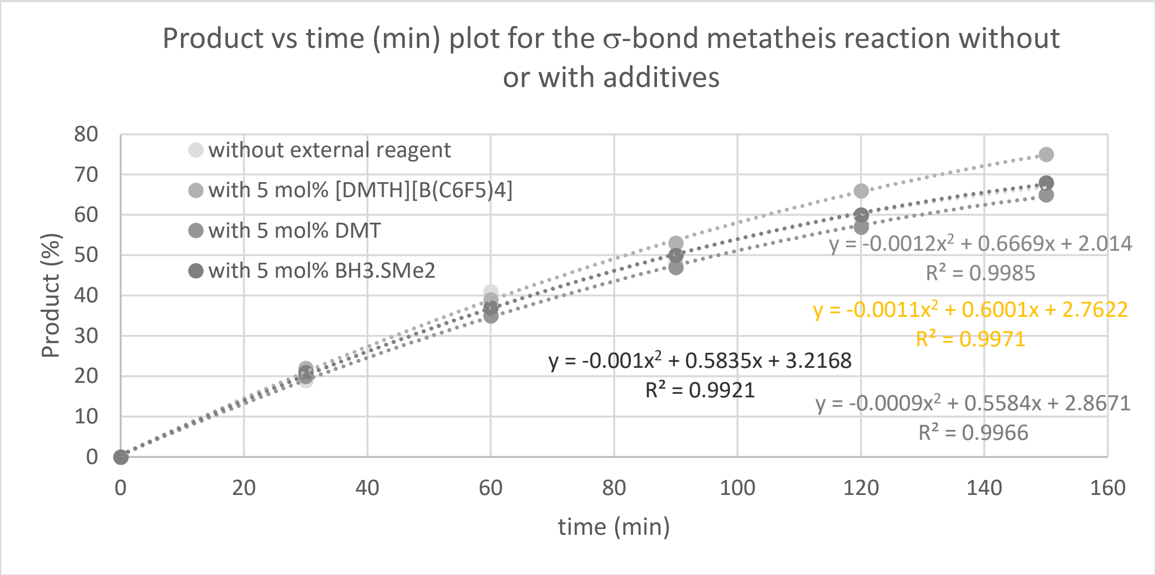

Figure S104: Product vs time plot for the σ-bond metathesis reaction without or with additives.

S5.11. Variable Temperature (VT) experiments

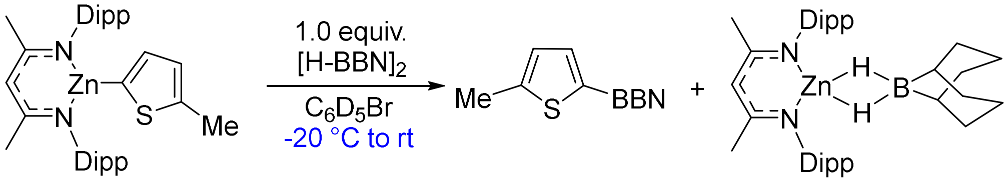

In a glovebox, ^{Dipp}NacNacZn-thienyl (11.9 mg, 0.020 mmol) and [H−BBN]$_2$ (5.0 mg, 0.020 mmol) charged in a J. Young's NMR tube were dissolved in C_6D_5Br (0.6 mL) at room temperature, which was sealed and immediately frozen using liquid nitrogen. The NMR tube was inserted into pre-cooled probe of Ava 400 MHz NMR spectrometer and the reaction mixture was monitored by ^{11}B NMR spectroscopy at different temperature from -20 ºC to 28 ºC. However, no other species (such as ^{Dipp}NacNacZnH) were detected in the selected temperature range in VT NMR.

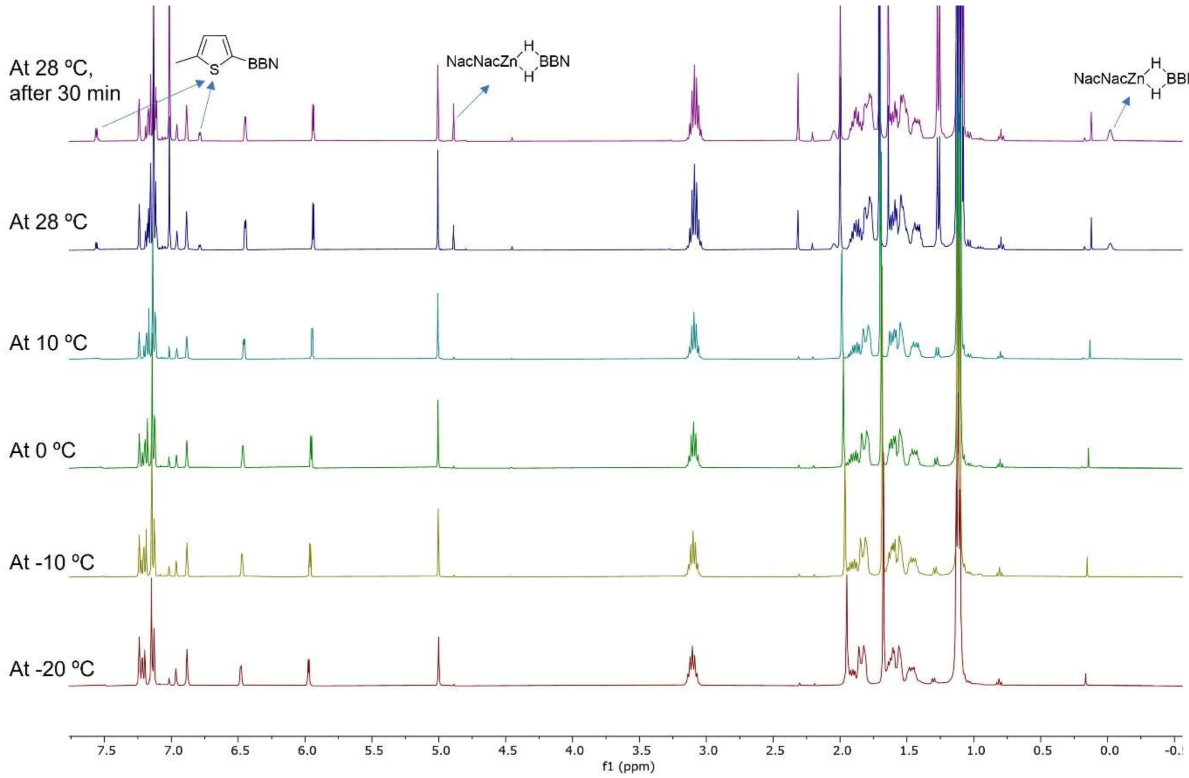

Figure S105: VT (-20 ºC to 28 ºC) dependent ^{1}H NMR spectrum in C_6D_5Br.

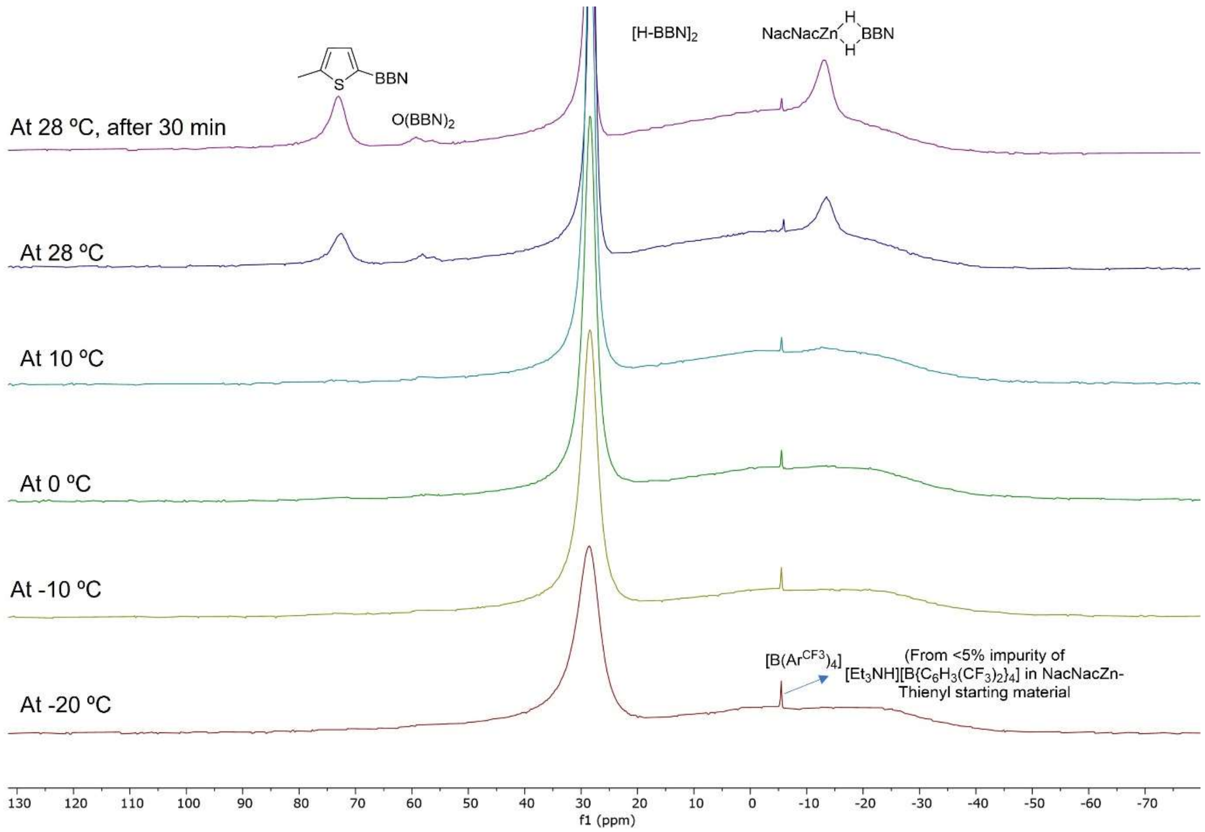

Figure S106: VT (-20 ºC to 28 ºC) dependent ^{11}B NMR spectrum in C_6D_5Br.

S5.12. Rate of C−H borylation w.r.t. catalyst loading

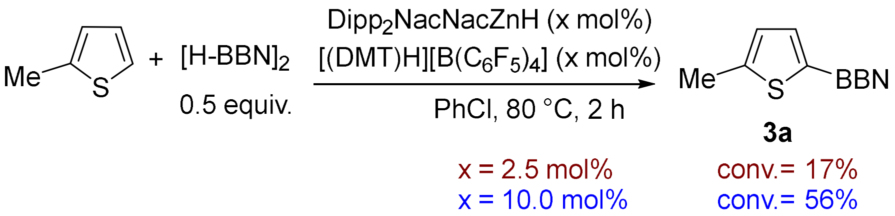

As per general procedure 2, using 2-methyl-thiophene (27.5 µL, 0.287 mmol, 1.15 equiv.) two different reactions were performed, one with 2.5 mol% and another with 10.0 mol% catalyst loading and heating at 80 °C for 2 h. Reaction progress was monitored by ^{1}H NMR spectroscopy and conversion to the C−H borylation product (**3a**) was determined from the relative ratio with unreacted 2-methylthiophne. From the outcome of the two different reactions, it was concluded that the rate of the reaction is dependent on the concentration of the zinc compound.

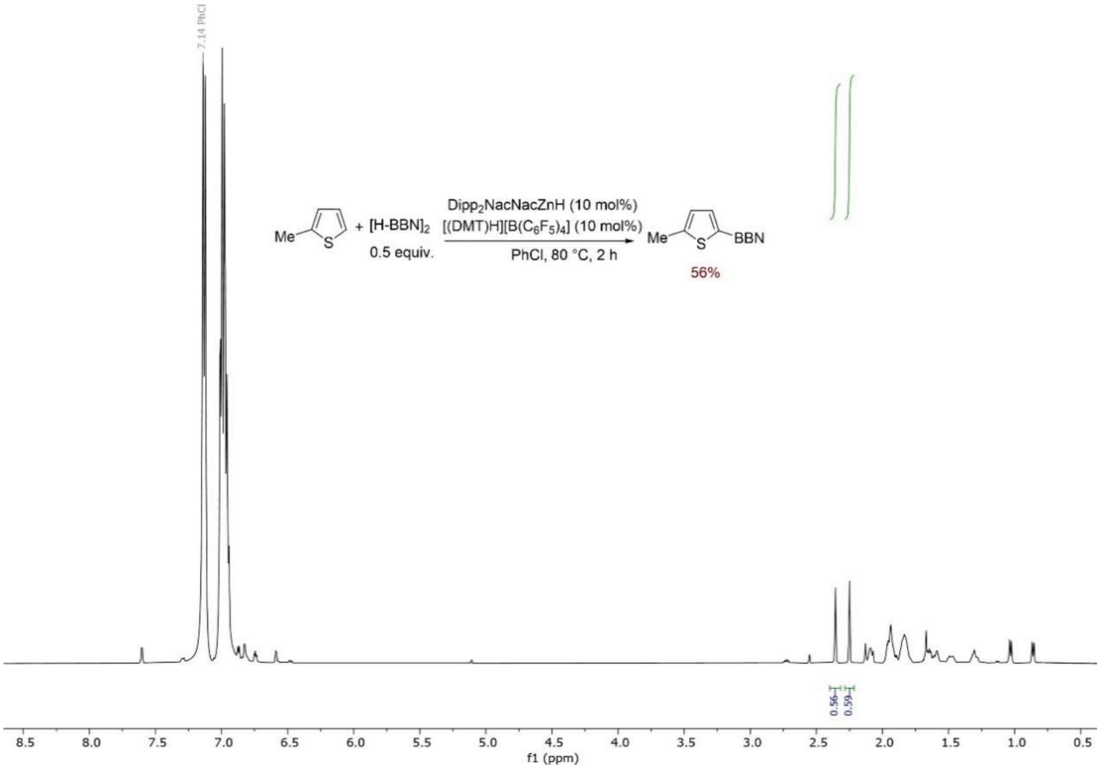

Figure S107: C–H borylation of 2-methylthiophene with 2.5 mol% catalyst loading in PhCl by in situ ^{1}H NMR spectroscopy.

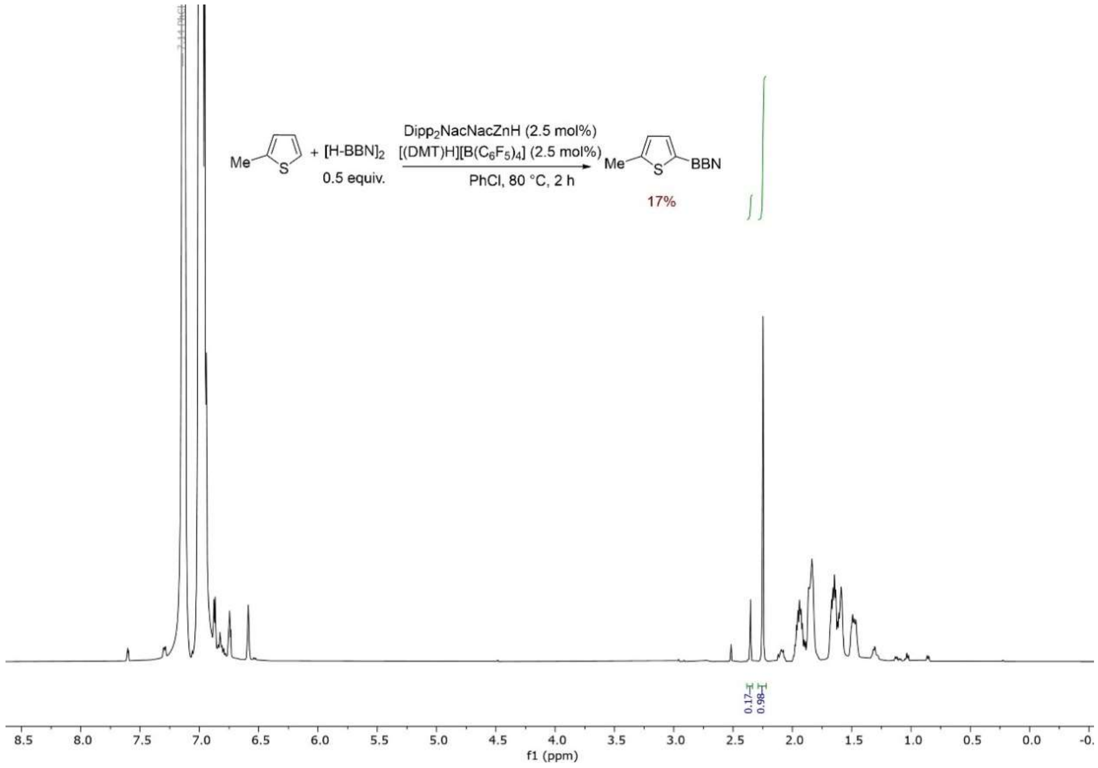

Figure S108: C–H borylation of 2-methylthiophene with 10 mol% catalyst loading in PhCl by in situ ^{1}H NMR spectroscopy.

S6. Crystallographic data

S6.1. Crystal structure of **3i-[H–BBN]**

CCDC Deposition Number: **2417816**

Experimental. Single colourless block-shaped crystals of **3i-[H–BBN]** recrystallised from dichloromethane by slow evaporation. A suitable crystal with dimensions 0.33 × 0.25 × 0.16 mm^3 was selected and mounted on a MITIGEN holder in paratone oil on a Bruker D8 Venture diffractometer. The crystal was kept at a steady T = 100.00 K during data collection. The structure was solved with the ShelXS (Sheldrick, 2008) solution program using direct methods and by using Olex2 1.5-beta (Dolomanov et al., 2009) as the graphical interface. The model was refined with olex2.refine 1.5-beta (Bourhis et al., 2015) using full matrix least squares minimisation on $\boldsymbol{F}^2$.

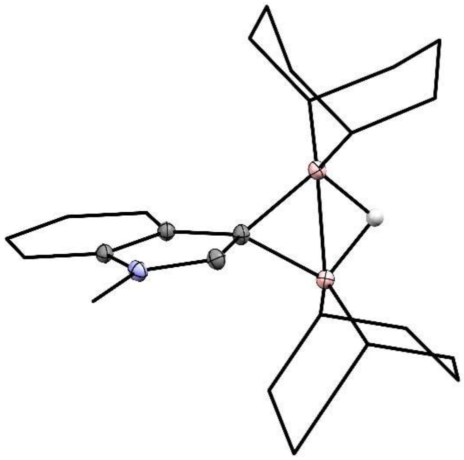

Compound	3i-[H–BBN]
Formula	$C_{25}H_{37}B_2N$
$D_{calc.}$/ g cm^{-3}	1.208
μ/mm^{-1}	0.067
Formula Weight	373.228
Colour	colourless
Shape	block-shaped
Size/mm^3	0.33×0.25×0.16
T/K	100.00
Crystal System	monoclinic
Space Group	$P2_1/n$
a/Å	11.2146(3)
b/Å	14.2724(4)
c/Å	13.2961(4)
α/°	90
β/°	105.2979(12)
γ/°	90
V/Å^3	2052.76(10)
Z	4
Z'	1
Wavelength/Å	0.71073
Radiation type	Mo K_a
Θ_{min}/°	2.12
Θ_{max}/°	37.83
Measured Refl's.	158724
Indep't Refl's	11041
Refl's I≥2σ(I)	8952
R_{int}	0.0477
Parameters	586
Restraints	0
Largest Peak	0.3550
Deepest Hole	-0.2404
GooF	1.0776
wR_2 (all data)	0.0468
wR_2	0.0411
R_1 (all data)	0.0403
R_1	0.0249

S6.2. Crystal structure of **3j-[H–BBN]**

CCDC Deposition Number: **2417817**

Experimental. Single colourless block-shaped crystals of **3j-[H–BBN]** recrystallised from d_2-dichloromethane by slow evaporation. A suitable crystal with dimensions 0.57 × 0.42 × 0.29 mm^3 was selected and mounted on a MITIGEN holder in paratone oil on a Bruker D8 Venture diffractometer. The crystal was kept at a steady T = 100.00 K during data collection. The structure was solved with the ShelXS (Sheldrick, 2008) solution program using direct methods and by using Olex2 1.5-beta (Dolomanov et al., 2009) as the graphical interface. The model was refined with olex2.refine 1.5-beta (Bourhis et al., 2015) using full matrix least squares minimisation on $\boldsymbol{F}^2$.

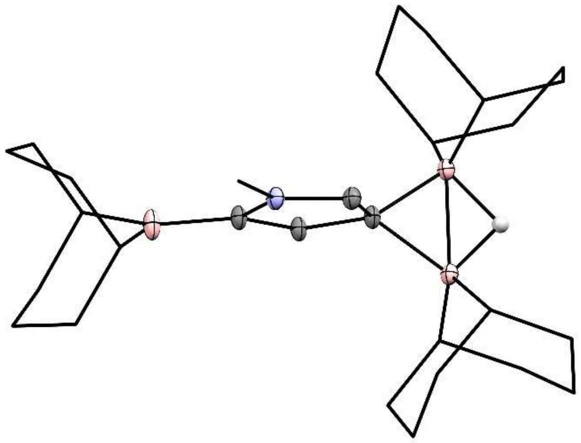

**Compound**	**3j-[H–BBN]**
Formula	$C_{29}H_{48}B_3N$
$D_{calc.}$/ g cm^{-3}	1.142
μ/mm^{-1}	0.063
Formula Weight	443.184
Colour	colourless
Shape	block-shaped
Size/mm^3	0.57×0.42×0.29
T/K	100.00
Crystal System	orthorhombic
Space Group	*Pnma*
a/Å	13.5001(5)
b/Å	15.0759(5)
c/Å	12.6679(5)
α/°	90
β/°	90
γ/°	90
V/Å^3	2578.25(16)
Z	4
Z'	0.5
Wavelength/Å	0.71073
Radiation type	Mo K_a
Θ_{min}/°	2.59
Θ_{max}/°	36.36
Measured Refl's.	232973
Indep't Refl's	6441
Refl's I≥2σ(I)	5450
R_{int}	0.0475
Parameters	388
Restraints	0
Largest Peak	0.3395
Deepest Hole	-0.1990
GooF	1.1684
wR_2 (all data)	0.0388
wR_2	0.0349
R_1 (all data)	0.0301
R_1	0.0199

S6.3. Crystal structure of **5c**

CCDC Deposition Number: **2417818**

Experimental. Single colourless slab-shaped crystals of **5c** recrystallised from chlorobenzene by slow evaporation. A suitable crystal with dimensions 0.34 × 0.15 × 0.07 mm^3 was selected and mounted on a mitegen tip NVH oil on a Bruker D8 VENTURE diffractometer. The crystal was kept at a steady T = 100.00 K during data collection. The structure was solved with the ShelXS (Sheldrick, 2008) solution program using direct methods and by using Olex2 1.5-beta (Dolomanov et al., 2009) as the graphical interface. The model was refined with olex2.refine 1.5-beta (Bourhis et al., 2015) using full matrix least squares minimisation on $\boldsymbol{F^2}$.

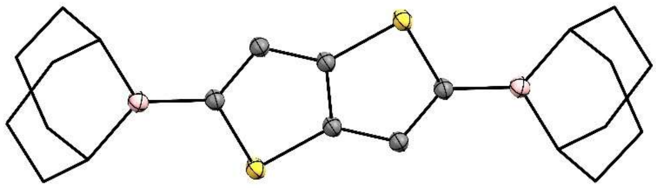

**Compound**	**5c**
Formula	$C_{22}H_{30}B_2S_2$
$D_{calc.}$/ g cm^{-3}	1.298
μ/mm^{-1}	0.277
Formula Weight	380.264
Colour	colourless
Shape	slab-shaped
Size/mm^3	0.34×0.15×0.07
T/K	100.00
Crystal System	triclinic
Space Group	P-1
a/Å	6.4133(7)
b/Å	8.766(1)
c/Å	9.6703(11)
α/°	112.891(3)
β/°	91.006(3)
γ/°	102.222(3)
V/Å^3	486.47(10)
Z	1
Z'	0.5
Wavelength/Å	0.71073
Radiation type	Mo K_α
Θ_{min}/°	2.30
Θ_{max}/°	33.17
Measured Refl's.	28911
Indep't Refl's	3539
Refl's I≥2σ(I)	3117
R_{int}	0.0407
Parameters	253
Restraints	18
Largest Peak	0.5784
Deepest Hole	-0.4715
GooF	1.0978
wR_2 (all data)	0.1183
wR_2	0.1121
R_1 (all data)	0.0494
R_1	0.0423

S6.4. Crystal structure of **5d**

CCDC Deposition Number: **2417819**

Experimental. Single clear colourless block-shaped crystals of **5d** recrystallised from chlorobenzene by slow evaporation. A suitable crystal with dimensions 0.28 × 0.09 × 0.05 mm^3 was selected and mounted on a MITIGEN holder NVH oil on a Rigaku Oxford Diffraction SuperNova diffractometer. The crystal was kept at a steady T = 120.00(10) K during data collection. The structure was solved with the ShelXS (Sheldrick, 2008) solution program using direct methods and by using Olex2 1.5-beta (Dolomanov et al., 2009) as the graphical interface. The model was refined with ShelXL 2018/3 (Sheldrick, 2015) using full matrix least squares minimisation on $\boldsymbol{F^2}$.

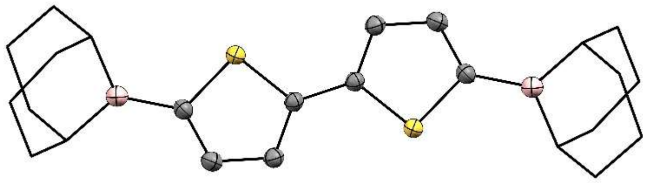

**Compound**	**5d**
Formula	$C_{24}H_{32}B_2S_2$
$D_{calc.}$/ g cm^{-3}	1.249
μ/mm^{-1}	2.259
Formula Weight	406.23
Colour	clear colourless
Shape	block-shaped
Size/mm^3	0.28×0.09×0.05
T/K	120.00(10)
Crystal System	triclinic
Space Group	P-1
a/Å	6.5122(2)
b/Å	9.5179(5)
c/Å	10.1405(7)
α/°	116.147(6)
β/°	98.075(4)
γ/°	99.496(3)
V/Å^3	540.11(6)
Z	1
Z'	0.5
Wavelength/Å	1.54184
Radiation type	Cu K_{α}
Θ_{min}/°	5.006
Θ_{max}/°	76.452
Measured Refl's.	5735
Indep't Refl's	5735
Refl's I≥2σ(I)	5068
R_{int}	0.1516
Parameters	129
Restraints	0
Largest Peak	0.794
Deepest Hole	-0.216
GooF	1.065
wR_2 (all data)	0.1330
wR_2	0.1300
R_1 (all data)	0.0509
R_1	0.0455

S6.5. Crystal structure of **5e**

CCDC Deposition Number: **2417820**

Experimental. Single translucent dark orange blade-shaped crystals of **5e** recrystallised from chlorobenzene by slow evaporation. A suitable crystal with dimensions 0.40 × 0.05 × 0.05 mm^3 was selected and mounted on a MITIGEN holder NVH oil on a Rigaku Oxford Diffraction SuperNova diffractometer. The crystal was kept at a steady T = 120.00(10) K during data collection. The structure was solved with the ShelXS (Sheldrick, 2008) solution program using direct methods and by using Olex2 1.5-beta (Dolomanov et al., 2009) as the graphical interface. The model was refined with ShelXL 2018/3 (Sheldrick, 2015) using full matrix least squares minimisation on $\boldsymbol{F}^2$.

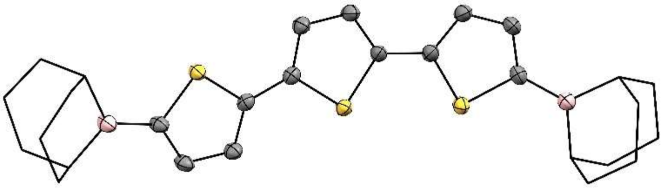

**Compound**	**5e**
Formula	$C_{28}H_{34}B_2S_3$
$D_{calc.}$/ g cm^{-3}	1.289
μ/mm^{-1}	2.786
Formula Weight	488.35
Colour	translucent dark orange
Shape	blade-shaped
Size/mm^3	0.40×0.05×0.05
T/K	120.00(10)
Crystal System	monoclinic
Space Group	0.204(14)
a/Å	-0.001(4)
b/Å	P21
c/Å	6.45827(7)
α/°	21.26402(19)
β/°	18.39205(17)
γ/°	90
V/Å^3	94.8085(9)
Z	90
Z'	2516.87(4)
Wavelength/Å	4
Radiation type	Cu K$_\alpha$
Θ_{min}/°	4.158
Θ_{max}/°	75.967
Measured Refl's.	97425
Indep't Refl's	10393
Refl's I≥2σ(I)	10035
R_{int}	0.0594
Parameters	742
Restraints	316
Largest Peak	0.441
Deepest Hole	-0.317
GooF	1.031
wR_2 (all data)	0.0975
wR_2	0.0959
R_1 (all data)	0.0394
R_1	0.0376

S6.6. Crystal structure of **11**

CCDC Deposition Number: **2417821**

Experimental. Single colourless block-shaped crystals of **11** recrystallised from d_6-benzene by slow evaporation. A suitable crystal with dimensions $0.14 \times 0.11 \times 0.09$ mm^3 was selected and mounted on a MITIGEN holder in Paratone oil on a Bruker D8 VENTURE diffractometer. The crystal was kept at a steady T = 100.00 K during data collection. The structure was solved with the **ShelXT** 2018/2 (Sheldrick, 2018) solution program using iterative methods and by using **Olex2** 1.5-beta (Dolomanov et al., 2009) as the graphical interface. The model was refined with **olex2.refine** 1.5-beta (Bourhis et al., 2015) using full matrix least squares minimisation on $\boldsymbol{F}^2$.

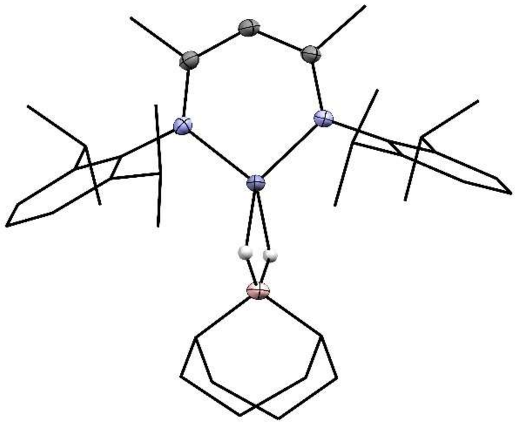

**Compound**	**11**
Formula	$C_{43}H_{57}BD_6N_2Zn$
$D_{calc.}$/ g cm^{-3}	1.149
μ/mm^{-1}	0.646
Formula Weight	690.17
Colour	colourless
Shape	block-shaped
Size/mm^3	0.14×0.11×0.09
T/K	100.00
Crystal System	monoclinic
Space Group	$P2_1/n$
a/Å	12.7925(4)
b/Å	24.0811(7)
c/Å	13.5539(5)
α/°	90
β/°	107.123(2)
γ/°	90
V/Å^3	3990.3(2)
Z	4
Z'	1
Wavelength/Å	0.71073
Radiation type	Mo K_α
Θ_{min}/°	2.10
Θ_{max}/°	33.19
Measured Refl's.	242361
Indep't Refl's	15206
Refl's I≥2σ(I)	12154
R_{int}	0.0479
Parameters	1082
Restraints	93
Largest Peak	0.3983
Deepest Hole	-0.4898
GooF	1.0899
wR_2 (all data)	0.0346
wR_2	0.0307
R_1 (all data)	0.0410
R_1	0.0237

S7. Computational details

DFT calculations were run with Gaussian 16 (Revision A.03).[15] Geometry optimizations and thermodynamic corrections were performed with the B3PW91 functional[16] with Zn and S centres described by Stuttgart RECPs and associated basis sets[17] and 6-31G** basis sets for all other atoms.[18,19] A set of d-orbital polarization functions was added to S ($\zeta^d = 0.503$).[20] All stationary points were fully characterized via analytical frequency calculations as either minima (all positive frequencies) or transition states (one negative frequency) and the latter were characterized via IRC calculations and subsequent geometry optimizations to confirm the adjacent minima. Electronic energies were recomputed with the B3PW91 functional[16] using def2-TZVP basis sets (BS2),[21,22] a correction for dispersion (BJD3)[23] and chlorobenzene solvent (PCM approach).[24] The thermochemical corrections from the B3PW91 frequency calculations were then added to give the free energies quoted in the text.

Additional functional testing was performed with the BP86,[25,26] BLYP,[25,27] B3LYP,[16] PBE,[28] PBE0,[29] B97D3,[30] B97D,[30] M06,[31] wB97x-D[32] and TPSS[33] functionals. Electronic energies were recomputed with each functional using def2-TZVP basis sets (BS2), a correction for dispersion (BJD3), where applicable (no dispersion was added to functionals: B97D, B97D3, M06 and wB97x-D), and chlorobenzene solvent (PCM approach). The thermochemical corrections from the B3PW91 frequency calculations were then added to give the free energies. Details of functional testing and all computed structures are provided below, the latter also as a separate XYZ file.

S7.1 Off-metal reactions of (H-BBN)$_2$ and functional testing

Table S4: Functional testing of the free energy change (ΔG) for the [H–BBN]$_2$ dimer splitting into 2 monomeric units.

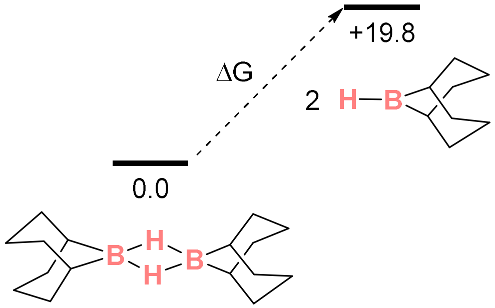

ΔG	BP86	BLYP	B3LYP	PBE	PBE0	B97D3	B97D	M06	WB97xD	TPSS	B3PW91
**H–BBN**	20.0	12.6	13.4	21.0	20.7	13.3	15.4	12.1	16.8	17.3	19.8

Table S5: Functional testing of the free energy change (ΔG) for the formation of H–BBN adducts of **3a**, **3i** and **3j**. Note, while **3i-/3j-(H–BBN)** were observed experimentally, the respective thienyl analogue, **3a-(H–BBN)**, was not observed in any of the reactions.

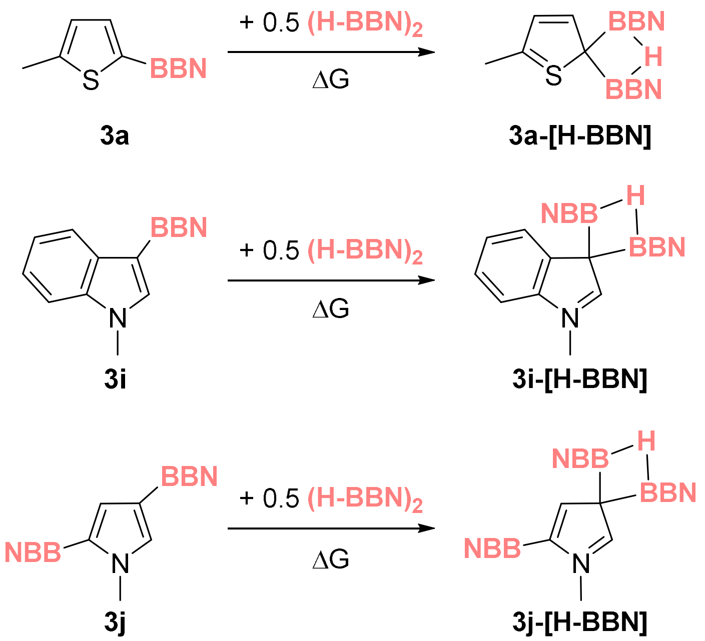

ΔG	BP86	BLYP	B3LYP	PBE	PBE0	B97D3	B97D	M06	wB97x-D	TPSS	B3PW91
**3a-[H-BBN]**	-2.1	4.3	4.9	0.2	0.7	3.9	2.0	2.8	2.6	0.6	-1.0
**3i-[H-BBN]**	-4.8	1.4	1.5	-2.6	-2.8	0.9	-2.9	0.1	-3.1	-2.3	-4.5
**3j-[H-BBN]**	-4.2	1.9	2.0	-2.4	-2.5	1.2	-2.3	0.4	-2.4	-2.0	-3.8

S7.2. Computed energy profiles for processes within the metal-catalysed C–H borylation

S7.2.1. Metalation and dehydrocoupling

Details of both of these processes are presented in Figure S109 and Figure S110, respectively, reproduced here from our earlier work.[5] An alternative dehydrocoupling mechanism involving protonation at C_γ of the NacNac ligand in **11** by $[DMT–H]^+$ followed by loss of H_2 is kinetically inaccessible (see Figure S111).

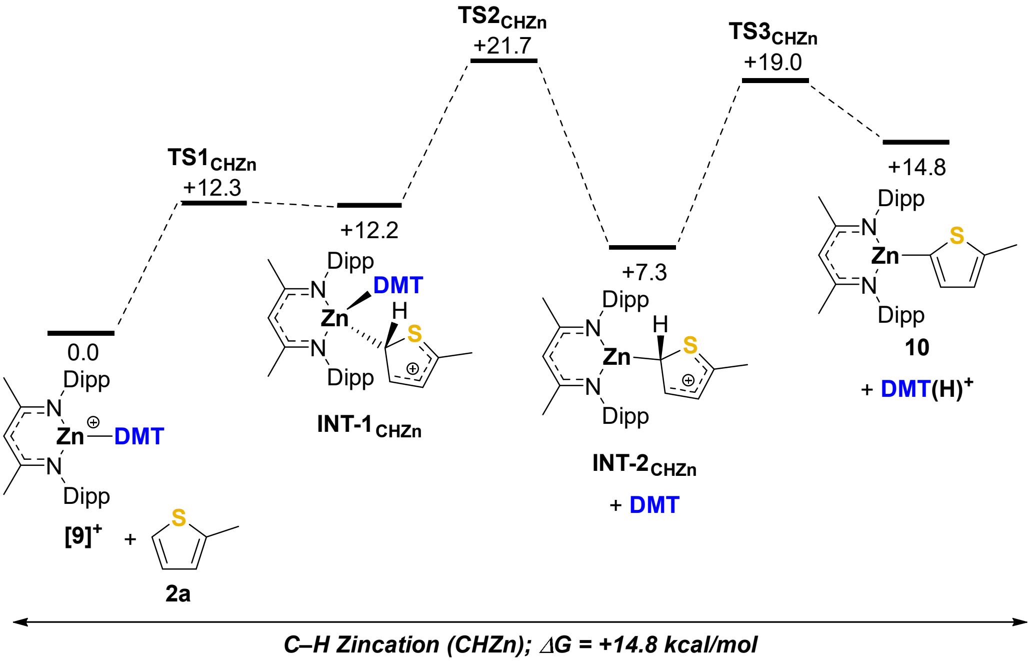

Figure S109: Computed free energy profile (kcal/mol) for the metalation phase of the catalytic C–H borylation of 2-methyl-thiophene [Method: B3PW91(def2-TZVP, BJD3, PhCl)//B3PW91(Zn: SDD; S: SDD(d); other atoms: 6-31G**)].

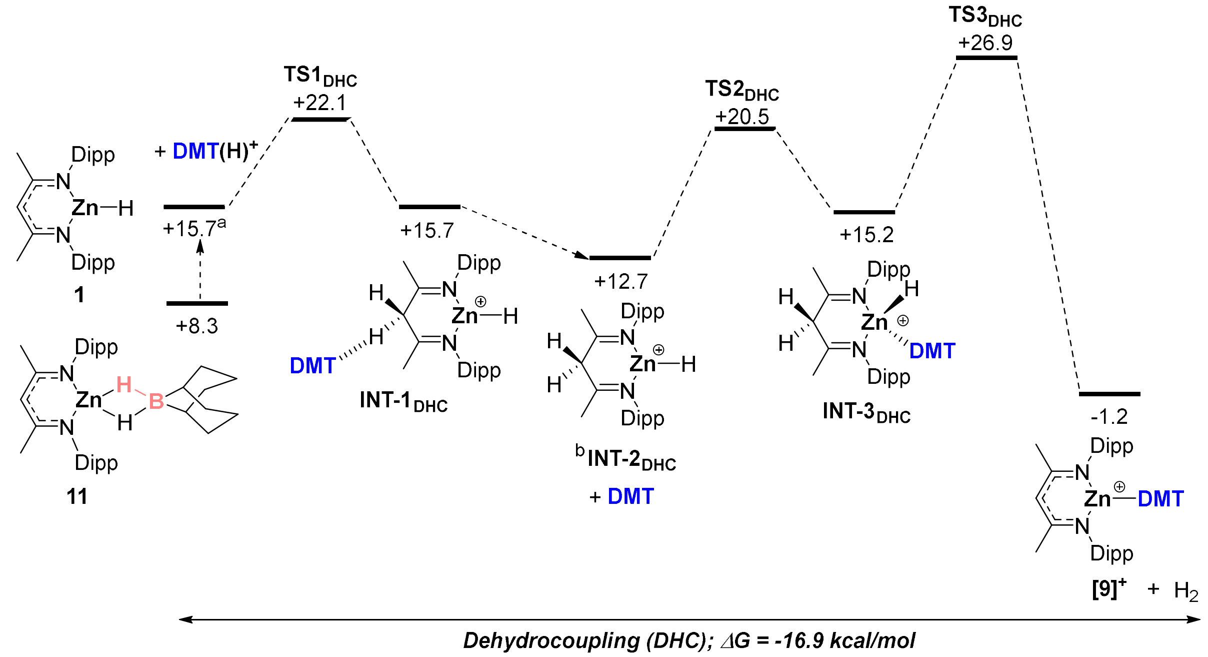

Figure S110: Computed free energy profile (kcal/mol) for the dehydrocoupling phase of the catalytic C–H borylation of 2-methyl-thiophene proceeding via NacNac ligand backbone protonation in **1** [Method: B3PW91(def2-TZVP, BJD3, PhCl)//B3PW91(Zn: SDD; S: SDD(d); other atoms: 6-31G**)]. [a] No transition state could be located for H–BBN dissociation from **11** to form **1**. [b] No transition state was found between intermediates **INT-1DHC** and **INT-2DHC**.

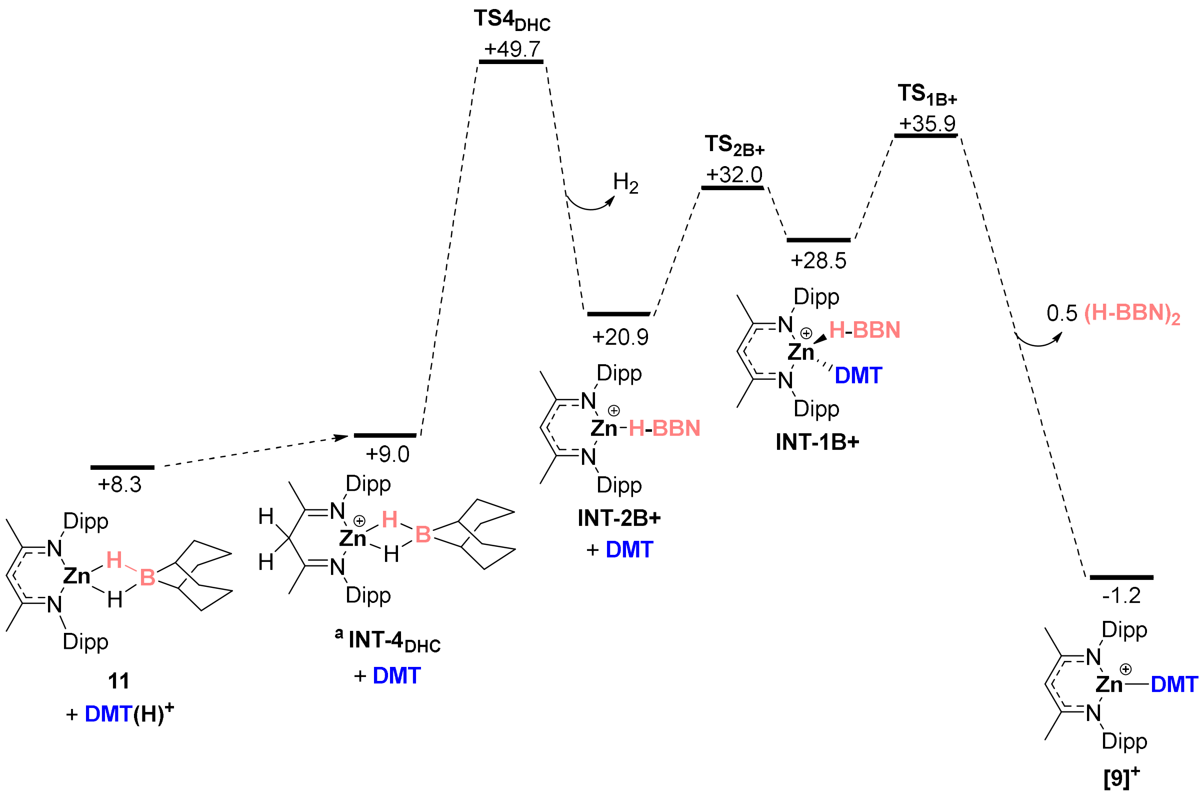

Figure S111: Computed free energy profile (kcal/mol) for the dehydrocoupling phase of the catalytic C–H borylation of 2-methyl-thiophene proceeding via NacNac ligand backbone protonation in **11** [Method: B3PW91(def2-TZVP, BJD3, PhCl)//B3PW91(Zn: SDD; S: SDD(d); other atoms: 6-31G**)]. [a] Location of a transition state between intermediates **11** and **INT-4**$_{\mathbf{DHC}}$ was not attempted.

S7.2.2. σ-Bond metathesis

The proposed pathway for the catalytic C–H borylation of 2-methyl-thiophene shown in Figure 5(a) in the main paper is reproduced here in Figure S112 with the enthalpies computed at 298 K included in parenthesis. This pathway is based on the observation of 1st order kinetics in the $(H–BBN)_2$ dimer that requires either the dimer or both H–BBN monomer units to be directly involved in the rate-determining process. Our proposed pathway involves **INT-1$_{Zn-B}$** as the key intermediate and takes into account both the computed energetics and the experimental observations. Pathways for the formation of **INT-1$_{Zn-B}$** are discussed below.

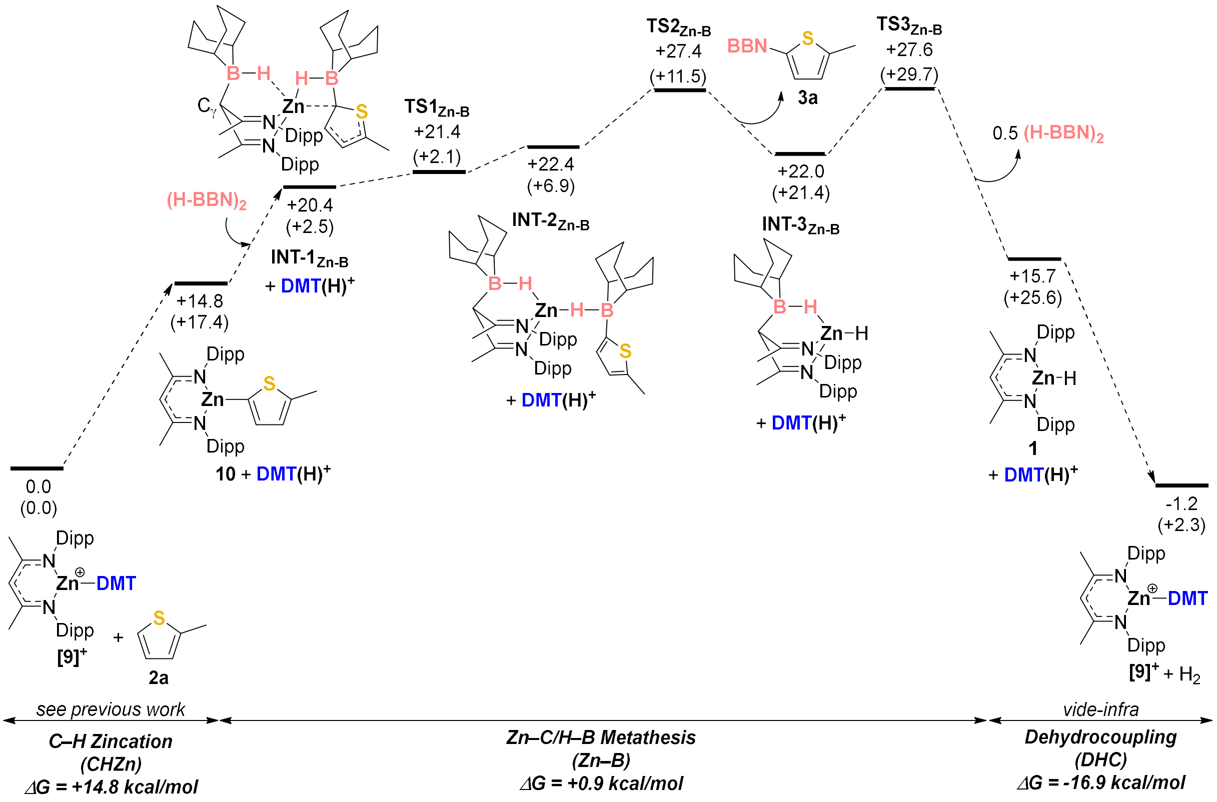

Figure S112: Computed free energy profile (kcal/mol) for the σ-bond metathesis phase of the catalytic C–H borylation of 2-methyl-thiophene [Method: B3PW91(def2-TZVP, BJD3, PhCl)//B3PW91(Zn: SDD; S: SDD(d); other atoms: 6-31G**)]. Computed enthalpies at 298 K are provided in parenthesis.

Computed mechanisms characterised for the addition of $(H–BBN)_2$ to **10** are shown in Figure S113. Pathway 1 involves direct addition of the $(H–BBN)_2$ dimer to **10** to give **INT-6$_{Zn-B}$** (+25.6 kcal/mol) in which the half-opened dimer bridges the Zn-C_{aryl} moiety. This proceeds via **TS7$_{Zn-B}$** at +49.9 kcal/mol. No transition state for the alternative direct addition of the (H–BBN)$_2$ dimer over the Zn···C_γ vector of the Zn(NacNac) moiety could be located, although an indirect route to this species, **Int-0A$_{Zn-B}$**, was characterized via stepwise addition of H–BBN monomers (Pathway 3b, see below). All attempts to locate a transition state for the direct formation of **INT-1$_{Zn-B}$** via dimer addition to **10** were unsuccessful.

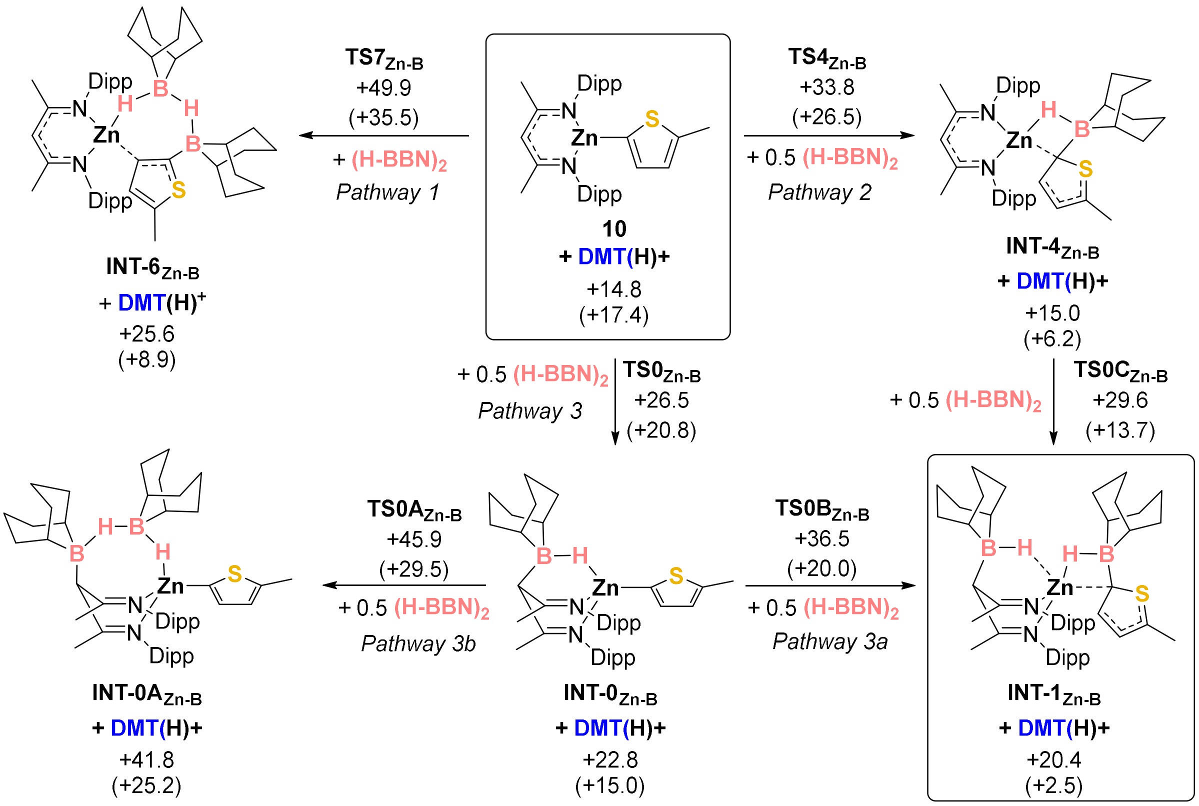

Figure S113: Computed free energy profiles (kcal/mol) surveying possible reactions of **10** with the $(H–BBN)_2$ dimer to form key intermediate **INT-1$_{Zn-B}$**. Details of alternative processes are also provided. [Method: B3PW91(def2-TZVP, BJD3, PhCl)//B3PW91(Zn: SDD; S: SDD(d); other atoms: 6-31G**)]. Computed enthalpies at 298 K are provided in parenthesis.

Off-metal dimer opening would allow the stepwise addition of two H–BBN monomers to **10**. In Pathway 2, H–BBN first adds across the Zn–C_{aryl} bond via **TS4$_{Zn-B}$** at +33.8 kcal/mol to give

INT-4$_{\mathbf{Zn\text{-}B}}$ at +15.0 kcal/mol. A second H–BBN monomer then adds across the Zn···C_γ vector via **TS0C**$_{\mathbf{Zn\text{-}B}}$ at +29.6 kcal/mol to give **INT-1**$_{\mathbf{Zn\text{-}B}}$.

Alternatively, Pathway 3 starts with H–BBN addition across the Zn···C_γ vector in **10** via **TS0**$_{\mathbf{Zn\text{-}B}}$ at +26.5 kcal/mol to give **INT-0**$_{\mathbf{Zn\text{-}B}}$ at +22.8 kcal/mol. Pathway 3a then involves H–BBN addition over the Zn-C_{aryl} bond via **TS0B**$_{\mathbf{Zn\text{-}B}}$ at +36.5 kcal/mol to give **INT-1**$_{\mathbf{Zn\text{-}B}}$. In Pathway 3b, insertion of the second H–BBN monomer into the Zn–H bond of **INT-0**$_{\mathbf{Zn\text{-}B}}$ gives **INT-0A**$_{\mathbf{Zn\text{-}B}}$ at +41.8 kcal/mol via **TS0A**$_{\mathbf{Zn\text{-}B}}$ at +45.9 kcal/mol. Location of transition states involving monomer delivery to **10** by $(H–BBN)_2$ dimer to form **INT-0**$_{\mathbf{Zn\text{-}B}}$ or **INT-4**$_{\mathbf{Zn\text{-}B}}$ was attempted but proved unsuccessful.

Overall, the calculations suggest Pathway 2 is the lowest energy route to **INT-1**$_{\mathbf{Zn\text{-}B}}$ and this proceeds via **TS4**$_{\mathbf{Zn\text{-}B}}$ as the highest-lying transition state with G = +33.8 kcal/mol. However, this initially forms **INT-4**$_{\mathbf{Zn\text{-}B}}$ and this species was shown to undergo facile product release with formation of **1** (see Figure S114). This pathway is therefore inconsistent with the 1st order in $(H–BBN)_2$ dimer seen experimentally.

Alternatively, Pathway 3a forms **INT-1**$_{\mathbf{Zn\text{-}B}}$ via **TS0B**$_{\mathbf{Zn\text{-}B}}$ at 36.5 kcal/mol, 2.7 kcal/mol higher than **TS4**$_{\mathbf{Zn\text{-}B}}$ along Pathway 2. **TS0B**$_{\mathbf{Zn\text{-}B}}$ involves addition of two H–BBN monomers and so is entropically disfavoured over **TS4**$_{\mathbf{Zn\text{-}B}}$ which involves only one monomer. Such entropic contributions calculated in the gas-phase are thought to be over-estimated compared to the solution phase[34,35] and so both the difference between **TS0B**$_{\mathbf{Zn\text{-}B}}$ and **TS4**$_{\mathbf{Zn\text{-}B}}$ and the absolute barriers for Pathways 2 and 3a are likely to exaggerated. One approach to address this issue has been to include only 50% of the gas-phase entropy in the calculation of the solution-phase free energy (denoted G_{50}[36,37]). Applying this approach gives G_{50} values of 29.1 kcal/mol for **TS4**$_{\mathbf{Zn\text{-}B}}$ compared to 23.6 kcal/mol for **TS0**$_{\mathbf{Zn\text{-}B}}$ and 28.3 kcal/mol for **TS0B**$_{\mathbf{Zn\text{-}B}}$. In the absence of entropic effects formation of **INT-1**$_{\mathbf{Zn\text{-}B}}$ via Pathway 3a is clearly favoured enthalpically (**TS0**$_{\mathbf{Zn\text{-}B}}$: H = 20.8 kcal/mol; **TS0B**$_{\mathbf{Zn\text{-}B}}$: H = 20.0 kcal/mol; **TS4**$_{\mathbf{Zn\text{-}B}}$ H = 26.5 kcal/mol).

With these caveats in mind, Pathway 3a represents our current best hypothesis for the formation of **INT-1**$_{\mathbf{Zn\text{-}B}}$.

We also note that the direct reaction of **10** with $(H\text{-}BBN)_2$ dimer via Pathway 3a entails a free energy barrier of 21.7 kcal/mol via **TS0B**$_{\mathbf{Zn\text{-}B}}$ as the rate-limiting transition state. Despite likely being over-estimated for the reasons described above, this barrier would still be consistent with the facile stoichiometric room temperature reaction that forms **3a** and **11**.

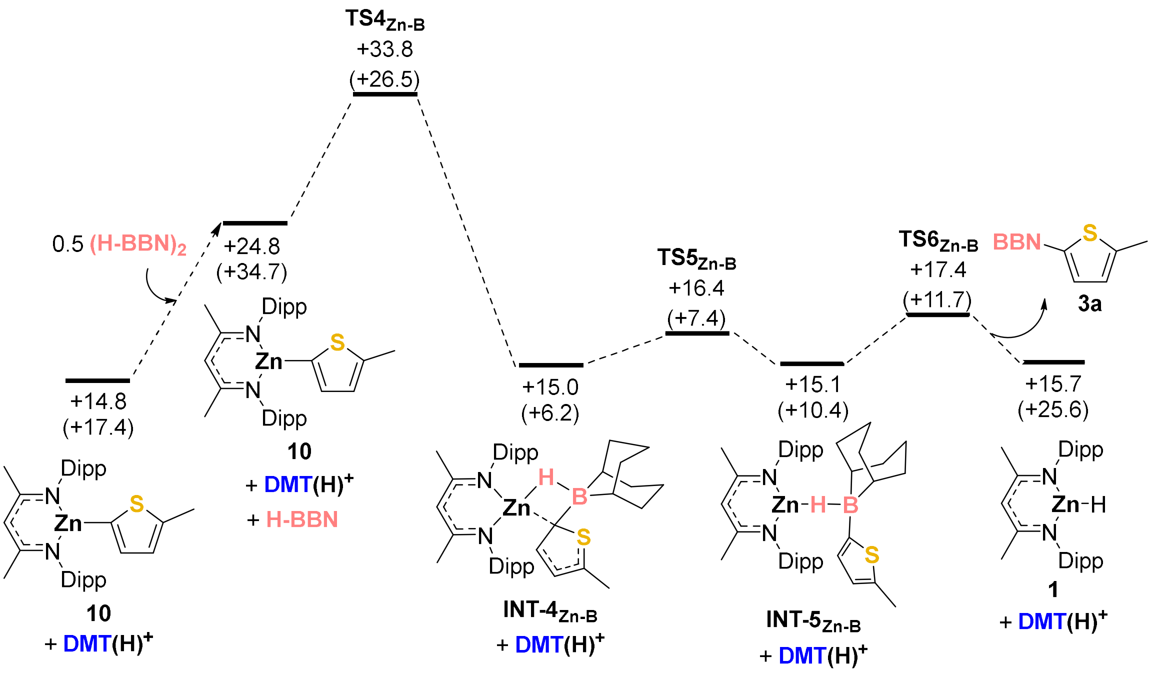

Figure S114: Computed free energy profile (kcal/mol) for the σ-bond metathesis phase of the catalytic C–H borylation of 2-methyl-thiophene proceeding via H–BBN monomer addition across the Zn–C bond [Method: B3PW91(def2-TZVP, BJD3, PhCl)//B3PW91(Zn: SDD; S: SDD(d); other atoms: 6-31G**)]. Computed enthalpies at 298 K are provided in parenthesis.

Details of the borenium-mediated mechanism summarised in Scheme 3 in the main paper are provided in Figure S115. The initial addition of a H–BBN monomer to **[9]**$^{+}$ is strongly disfavoured on both free energy and enthalpy grounds.

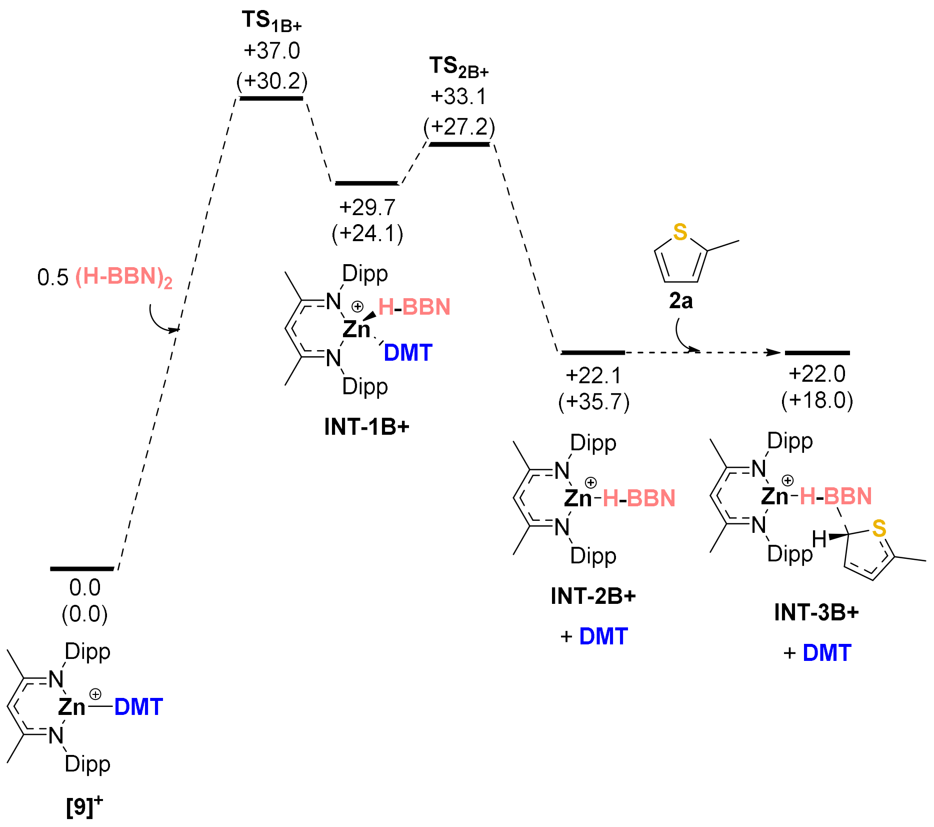

Figure S115: Computed selected points of the free energy profile (kcal/mol) for the borenium-mediated catalytic C–H borylation of 2-methyl-thiophene [Method: B3PW91(def2-TZVP, BJD3, PhCl)//B3PW91(Zn: SDD; S: SDD(d); other atoms: 6-31G**)]. Computed enthalpies at 298 K are provided in parenthesis.

S7.3. Computed cartesian coordinates (Å) and energies (hartrees) for all species

H2
B3PW91
SCF = -1.17751660098
SCF (C6H5Cl) = -1.17761829839
SCF (D3BJ) = -1.17765349976
SCF (BS2) = -1.17863419440
H (0 K) = -1.167369
H (298 K) = -1.164064
G (298 K) = -1.178858
Low freq. = 4454.3412
Second freq. =

2
H2
H 0.00000 0.00000 0.37177
H 0.00000 0.00000 -0.37177

DMT
B3PW91
SCF = -405.392451505
SCF (C6H5Cl) = -405.395908470
SCF (D3BJ) = -405.432669780
SCF (BS2) = -405.523891386
H (0 K) = -405.191017
H (298 K) = -405.179474
G (298 K) = -405.228086
Low freq. = 22.1259
Second freq. = 72.2345

23
DMT
H 2.48607 1.99031 -0.67122
H 3.83398 -1.05959 -0.02431
H 3.83398 1.05959 -0.02431
C 2.76194 -1.23900 0.07792
C 2.76194 1.23900 0.07792
H 2.57986 -1.67232 1.07500
H 2.57986 1.67232 1.07500
N 2.05027 -0.00000 -0.14370
H 2.48607 -1.99031 -0.67122
C -0.07402 1.20208 -0.04848
C 0.66246 0.00000 -0.07606
C -1.46406 1.18988 -0.01764
C -2.19708 0.00000 -0.00071
C -0.07402 -1.20208 -0.04848
C -1.46406 -1.18988 -0.01764
H 0.43592 -2.15865 -0.05524
H -1.99160 -2.14177 -0.00265
C -3.70149 -0.00000 0.06872
H -4.12519 0.88402 -0.41846
H -4.05966 -0.00001 1.10646
H -4.12519 -0.88401 -0.41847
H -1.99160 2.14177 -0.00265
H 0.43592 2.15865 -0.05524

[DMTH]+
B3PW91
SCF = -405.772731399
SCF (C6H5Cl) = -405.836130532
SCF (D3BJ) = -405.815448175
SCF (BS2) = -405.897902913
H (0 K) = -405.556370
H (298 K) = -405.544953
G (298 K) = -405.593015
Low freq. = 30.3062
Second freq. = 51.3724

24
[DMTH]+
H 2.21802 0.23750 2.11422
H 3.76750 0.04538 -1.22047
H 3.76460 0.02373 1.22989
C 2.70266 -0.19144 -1.24403
C 2.69970 -0.21338 1.24689
H 2.56039 -1.27087 -1.27936
H 2.55749 -1.29328 1.26295
N 2.05816 0.33939 0.00548
H 2.22286 0.27461 -2.10436
C -0.20002 1.33265 0.00450
C 0.58133 0.18431 0.00236
C -1.58660 1.20236 -0.00261
C -2.19728 -0.05738 -0.00987
C 0.01369 -1.08576 -0.00752
C -1.37305 -1.19250 -0.01447
H 0.62127 -1.98616 -0.01310
H -1.82353 -2.18080 -0.02537
C -3.69328 -0.19466 0.00920
H -4.18474 0.70560 -0.36656
H -4.04904 -0.36296 1.03242
H -4.02408 -1.04414 -0.59417
H -2.20050 2.09814 -0.00405
H 0.25028 2.32371 0.00835
H 2.22935 1.34857 0.01462

H-BBN
B3PW91
SCF = -338.622980810
SCF (C6H5Cl) = -338.623555833
SCF (D3BJ) = -338.667863097
SCF (BS2) = -338.720434224
H (0 K) = -338.403779
H (298 K) = -338.394463
G (298 K) = -338.436461
Low freq. = 79.2151
Second freq. = 114.8214

24
H-BBN
C 0.00563 1.30360 0.70460
B 0.15687 -0.00002 1.56283
C 0.00343 -1.30350 0.70470
C 1.28967 -1.29055 -0.17905
C 1.51567 -0.00122 -0.98684
C 1.29277 1.28789 -0.17791
C -1.32479 -1.28973 -0.08676
C -1.61553 0.00122 -0.86910
C -1.32183 1.29235 -0.08808
H 0.03871 2.22423 1.30036
H 0.50679 -0.00017 2.71268
H 0.03519 -2.22410 1.30058
H 1.27406 -2.15517 -0.85761
H 2.15530 -1.44714 0.48078
H 2.54232 -0.00229 -1.37508
H 0.87233 0.00004 -1.87056
H 2.15829 1.44137 0.48282
H 1.28022 2.15324 -0.85558
H -2.13889 -1.45316 0.63379
H -1.36326 -2.14690 -0.77424
H -1.05105 -0.00001 -1.80590
H -2.67096 0.00229 -1.17056
H -2.13607 1.45878 0.63162
H -1.35749 2.14881 -0.77661

[H-BBN]2
B3PW91
SCF = -677.293580347
SCF (C6H5Cl) = -677.294116018
SCF (D3BJ) = -677.398999131
SCF (BS2) = -677.486072707
H (0 K) = -676.848762
H (298 K) = -676.831386
G (298 K) = -676.891919
Low freq. = 41.5827
Second freq. = 54.9800

48
[H-BBN]2
C 2.62839 -1.30337 -1.29493
C 1.79366 -0.00012 -1.30975
C 2.62770 1.30363 -1.29473
C 3.41707 1.56661 0.00004
H 1.93568 2.14249 -1.46021
H 3.32173 1.31859 -2.14713
C 2.62795 1.30341 1.29491
H 3.75965 2.60952 0.00010
H 4.33093 0.96567 -0.00011
C 1.79382 -0.00027 1.30983
H 1.93602 2.14229 1.46070
H 3.32214 1.31815 2.14718
C 2.62846 -1.30359 1.29472
C 3.41780 -1.56619 -0.00015
H 1.93687 -2.14280 1.46028
H 3.32260 -1.31823 2.14703
H 3.32251 -1.31780 -2.14728
H 1.93686 -2.14259 -1.46063
H 3.76085 -2.60895 -0.00025
H 4.33139 -0.96484 -0.00010
B 0.89580 -0.00030 0.00009
H 1.20330 -0.00017 -2.23547
H 1.20355 -0.00044 2.23561
H -3.32198 1.31862 2.14703
H -1.20357 -0.00019 2.23561
H -3.32281 -1.31777 2.14717
C -2.62784 1.30365 1.29471
H -1.93581 2.14249 1.46031
C -1.79384 -0.00013 1.30983
C -2.62859 -1.30336 1.29492
H -1.93709 -2.14260 1.46072
C -3.41702 1.56668 -0.00015
H -3.75952 2.60962 -0.00025
B -0.89581 -0.00032 0.00010
H -4.33093 0.96582 -0.00011
C -3.41782 -1.56621 0.00003
H -4.33143 -0.96490 -0.00012
H -3.76084 -2.60898 0.00009
C -2.62776 1.30342 -1.29493
C -1.79367 -0.00028 -1.30974
H -1.93579 2.14228 -1.46066
C -2.62833 -1.30358 -1.29474
H -1.93673 -2.14280 -1.46023
H -3.32187 1.31818 -2.14728
H -1.20330 -0.00046 -2.23546
H -3.32238 -1.31822 -2.14713
H -0.00001 0.97727 0.00018
H -0.00000 -0.97791 0.00018

2-methyl-thiophene, **2a**
B3PW91
SCF = -204.285929660
SCF (C6H5Cl) = -204.288401429
SCF (D3BJ) = -204.306656123
SCF (BS2) = -592.304637144
H (0 K) = -204.191145
H (298 K) = -204.184383
G (298 K) = -204.220876
Low freq. = 95.1333
Second freq. = 230.7174

12
2-methyl-thiophene, **2a**
C -0.78516 0.22412 -0.00000
C -0.01658 1.35682 0.00003
C 1.38631 1.10015 -0.00003
C 1.67832 -0.23296 -0.00001
S 0.23444 -1.19357 0.00001
C -2.27587 0.10684 -0.00001
H -0.44808 2.35278 0.00004
H 2.14408 1.87607 -0.00006
H 2.64664 -0.71446 -0.00002
H -2.64577 -0.42561 0.88336
H -2.64577 -0.42560 -0.88337
H -2.72422 1.10406 0.00000

3a
B3PW91
SCF = -541.744348265
SCF (C6H5Cl) = -541.746433804
SCF (D3BJ) = -541.819131582
SCF (BS2) = -929.857235639
H (0 K) = -541.447655
H (298 K) = -541.432357
G (298 K) = -541.489840
Low freq. = 31.8873
Second freq. = 45.0480

34
3a
C -1.54309 1.32836 0.00035
B -0.49408 0.14729 0.00007
C -1.21849 -1.25499 -0.00024
C -2.05010 -1.34862 -1.30614
C -2.99873 -0.16703 -1.56636
C -2.37194 1.21234 -1.30554
C -2.04993 -1.34909 1.30574
C -2.99886 -0.16782 1.56624
C -2.37237 1.21180 1.30588
H -1.07848 2.32363 0.00062
H -0.51987 -2.10379 -0.00044
H -2.62495 -2.28591 -1.31717
H -1.34364 -1.42260 -2.14543
H -3.34011 -0.20961 -2.60891
H -3.90381 -0.28095 -0.96308
H -1.70554 1.45872 -2.14464
H -3.16119 1.97807 -1.31682
H -1.34338 -1.42307 2.14495
H -2.62454 -2.28654 1.31664
H -3.90386 -0.28182 0.96285
H -3.34030 -0.21079 2.60874
H -1.70632 1.45818 2.14525
H -3.16184 1.97730 1.31707
H 5.12338 -1.12120 -0.88180
S 2.15221 -0.98960 -0.00018
C 3.15243 1.36821 0.00014
C 3.53898 0.04535 -0.00006
C 1.02409 0.35695 0.00005
C 4.93139 -0.50011 0.00008
C 1.74983 1.53676 0.00020
H 3.86639 2.18587 0.00023
H 5.65482 0.31947 -0.00164
H 1.26476 2.50767 0.00034
H 5.12428 -1.11824 0.88387

3a-[H-BBN]
B3PW91
SCF = -880.387552916
SCF (C6H5Cl) = -880.390331035
SCF (D3BJ) = -880.539003527
SCF (BS2) = -1268.59254027
H (0 K) = -879.867141
H (298 K) = -879.843684
G (298 K) = -879.917253
Low freq. = 44.1428
Second freq. = 47.4925

58
3a-[H-BBN]
H -0.88151 -5.08677 -1.04611
S 0.00007 -2.13028 -1.05755
C 0.00008 -0.91368 0.23508
C -0.00016 -1.61040 1.45165
C -0.00023 -4.86933 -0.43341
C -0.00030 -3.45108 0.03996
C -0.00030 -3.00754 1.35193
H 0.88389 -5.08806 -1.04157
H -0.00041 -1.08154 2.39872
H -0.00051 -3.68290 2.20087

H -0.00289 -5.55149 0.42015
C -2.24145 2.03640 -1.51364
C -1.87809 0.55078 -1.27973
C -3.10048 -0.36910 -1.06943
C -3.88493 -0.14268 0.23500
H -2.74286 -1.40817 -1.08390
H -3.79097 -0.28017 -1.92083
C -3.00682 0.05301 1.48287
H -4.55153 -0.99951 0.40035
H -4.55075 0.71765 0.11947
C -1.79631 0.99143 1.29406
H -2.62495 -0.92940 1.79680
H -3.63824 0.40687 2.31093
C -2.17424 2.47068 1.03931
C -2.86427 2.75815 -0.30597
H -1.24898 3.06230 1.09094
H -2.81196 2.84512 1.85344
H -2.92117 2.12926 -2.37325
H -1.32117 2.56409 -1.80310
H -2.84402 3.84041 -0.49022
H -3.92527 2.50115 -0.23556
B -0.94627 0.48695 0.02830
H -1.36118 0.20465 -2.18717
H -1.22029 0.97485 2.23056
H 3.63846 0.40639 2.31079
H 1.22060 0.97434 2.23074
H 2.81214 2.84472 1.85374
C 3.00700 0.05264 1.48270
H 2.62513 -0.92979 1.79652
C 1.79646 0.99112 1.29415
C 2.17435 2.47039 1.03961
H 1.24907 3.06199 1.09143
C 3.88507 -0.14285 0.23476
H 4.55169 -0.99969 0.40000
B 0.94639 0.48663 0.02842
H 4.55087 0.71750 0.11934
C 2.86425 2.75811 -0.30567
H 3.92527 2.50118 -0.23535
H 2.84391 3.84039 -0.48976
C 3.10058 -0.36909 -1.06967
C 1.87817 0.55079 -1.27969
H 2.74297 -1.40816 -1.08430
C 2.24140 2.03647 -1.51339
H 1.32106 2.56412 -1.80272
H 3.79104 -0.28001 -1.92109
H 1.36115 0.20481 -2.18713
H 2.92105 2.12951 -2.37303
H 0.00006 1.38549 -0.17549

3i
B3PW91
SCF = -740.440548814
SCF (C6H5Cl) = -740.445265875
SCF (D3BJ) = -740.531239675
SCF (BS2) = -740.668405488
H (0 K) = -740.080257
H (298 K) = -740.062839
G (298 K) = -740.125353
Low freq. = 14.3619
Second freq. = 30.8381

41
3i
N 2.41092 1.62035 -0.00005
C 1.05020 1.65332 -0.00000
H 0.54303 2.60959 -0.00004
C 0.49605 0.37940 0.00001
C 1.65006 -0.51106 0.00000
C 1.82002 -1.90397 0.00003
H 0.96035 -2.56520 0.00006
C 3.10291 -2.43748 0.00002
H 3.23238 -3.51630 0.00003
C 4.23874 -1.61060 -0.00002
H 5.22889 -2.05747 -0.00004
C 4.11144 -0.22728 -0.00005
H 4.98639 0.41654 -0.00010
C 2.81711 0.29463 -0.00003
C 3.29751 2.75994 0.00014
H 3.93641 2.75647 0.88986
H 3.93576 2.75737 -0.89006
H 2.70371 3.67542 0.00083
C -2.09201 1.29663 -0.00011
H -1.64725 2.30183 -0.00016
C -2.92135 1.17893 1.30415
H -2.25636 1.42958 2.14343
H -3.71620 1.93941 1.31569
C -3.53830 -0.20379 1.56685
H -4.44238 -0.32503 0.96325
H -3.87968 -0.24885 2.60950
C -2.57905 -1.37554 1.30544
H -1.87187 -1.44278 2.14479
H -3.14599 -2.31805 1.31773
C -1.74777 -1.27571 0.00020
H -1.05863 -2.12814 0.00029
C -2.57916 -1.37596 -1.30494
H -1.87201 -1.44381 -2.14427
H -3.14636 -2.31831 -1.31673
C -3.53808 -0.20405 -1.56698
H -4.44230 -0.32478 -0.96349

H -3.87927 -0.24945 -2.60967
C -2.92083 1.17860 -1.30470
H -3.71546 1.93930 -1.31690
H -2.25542 1.42868 -2.14383
B -1.01581 0.12912 0.00005

3i-[H-BBN]
B3PW91
SCF = -1079.08888821
SCF (C6H5Cl) = -1079.09460242
SCF (D3BJ) = -1079.25782221
SCF (BS2) = -1079.40905831
H (0 K) = -1078.504377
H (298 K) = -1078.478934
G (298 K) = -1078.556151
Low freq. = 41.7718
Second freq. = 42.5688

65
3i-[H-BBN]
N 2.41271 0.00187 1.61452
C 1.06996 0.00094 1.59224
H 0.51597 0.00051 2.52163
C 0.55247 0.00068 0.28615
C 1.75923 0.00159 -0.55648
C 1.99908 0.00191 -1.93752
H 1.17552 0.00140 -2.64128
C 3.30949 0.00292 -2.40349
H 3.49166 0.00316 -3.47436
C 4.40160 0.00363 -1.52280
H 5.41301 0.00443 -1.91820
C 4.20190 0.00336 -0.14765
H 5.04039 0.00397 0.54242
C 2.88244 0.00232 0.30087
C 3.25368 0.00237 2.79250
H 3.89043 -0.88744 2.80731
H 3.88774 0.89411 2.80860
H 2.62321 0.00077 3.68243
C -1.43318 1.78350 1.24633
H -1.48835 1.19766 2.17630
C -0.50723 2.98803 1.52106
H 0.44536 2.60030 1.91258
H -0.92370 3.61651 2.32237
C -0.20155 3.87387 0.30043
H 0.64898 4.52673 0.53855
H -1.04120 4.55318 0.12586
C 0.11206 3.09792 -0.99098
H 1.14081 2.71774 -0.93127
H 0.10203 3.80129 -1.83672
C -0.81438 1.89789 -1.28371
H -0.42068 1.39866 -2.17855
C -2.27480 2.28836 -1.61114
H -2.29969 2.98526 -2.46204
H -2.79249 1.38080 -1.95440
C -3.07439 2.89719 -0.44676
H -2.81208 3.95362 -0.33564
H -4.14125 2.89354 -0.70704
C -2.88804 2.17611 0.89909
H -3.48573 1.25330 0.88664
H -3.31760 2.79785 1.69864
C -1.42988 -1.78602 1.24631
H -1.48606 -1.20039 2.17635
C -0.50173 -2.98890 1.52084
H 0.45022 -2.59947 1.91224
H -0.91696 -3.61816 2.32217
C -0.19463 -3.87410 0.30013
H -1.03313 -4.55482 0.12554
H 0.65702 -4.52555 0.53815
C 0.11760 -3.09748 -0.99120
H 1.14568 -2.71550 -0.93147
H 0.10878 -3.80077 -1.83703
C -0.81096 -1.89907 -1.28377
H -0.41816 -1.39903 -2.17855
C -2.27070 -2.29208 -1.61121
H -2.78998 -1.38541 -1.95442
H -2.29438 -2.98898 -2.46215
C -3.06923 -2.90240 -0.44686
H -2.80515 -3.95840 -0.33591
H -4.13610 -2.90050 -0.70710
C -2.88406 -2.18121 0.89910
H -3.31242 -2.80387 1.69858
H -3.48341 -1.25949 0.88686
B -0.83420 0.94227 0.00945
H -1.72204 -0.00146 -0.26222
B -0.83254 -0.94359 0.00948

3j
B3PW91
SCF = -924.303209916
SCF (C6H5Cl) = -924.306097590
SCF (D3BJ) = -924.429532528
SCF (BS2) = -924.576685439
H (0 K) = -923.787890
H (298 K) = -923.764701
G (298 K) = -923.840899
Low freq. = 13.1715
Second freq. = 23.9640

57
3j
N -0.60602 1.89930 -0.00000
C -1.10580 0.58087 -0.00025
C 0.73951 1.87939 0.00009
H 1.29828 2.80724 0.00024
C 1.21473 0.56081 -0.00008
C 0.03233 -0.22645 -0.00031
H 0.00502 -1.30811 -0.00049
C -1.38159 3.12350 0.00012
H -2.01448 3.18528 -0.88875
H -0.69539 3.97261 -0.00045
C -2.91044 -1.44553 -0.00087
H -2.01882 -2.08499 -0.00153
C -3.68616 -1.76058 -1.30520
H -3.99006 -2.81749 -1.31526
H -2.98501 -1.64398 -2.14407
C -4.91456 -0.87639 -1.56673
H -5.75707 -1.22480 -0.96263
H -5.23360 -1.00673 -2.60913
C -4.67261 0.61769 -1.30451
H -5.63523 1.14948 -1.31350
H -4.09697 1.03061 -2.14603
C -3.90218 0.95184 0.00065
H -3.75453 2.03707 0.00114
B -2.56203 0.10223 -0.00020
H -2.01360 3.18573 0.88960
C 3.92534 1.09087 0.00046
H 3.64383 2.15363 0.00077
C 4.72270 0.83423 -1.30448
H 4.10913 1.19172 -2.14421
H 5.63323 1.45126 -1.31707
C 5.10030 -0.63305 -1.56589
H 5.97164 -0.90244 -0.96221
H 5.42997 -0.73396 -2.60834
C 3.96028 -1.63128 -1.30570
H 3.25214 -1.57995 -2.14536
H 4.36382 -2.65439 -1.31904
C 3.15731 -1.39428 -0.00057
H 2.32217 -2.10801 -0.00095
C 3.96023 -1.63257 1.30432
H 3.25194 -1.58258 2.14393
H 4.36422 -2.65551 1.31645
C 5.09975 -0.63413 1.56600
H 5.97148 -0.90250 0.96244
H 5.42900 -0.73595 2.60850
C 4.72169 0.83325 1.30587
H 5.63194 1.45067 1.31987
H 4.10726 1.18959 2.14547
C -3.68512 -1.76183 1.30380
H -3.98900 -2.81875 1.31309
H -2.98330 -1.64602 2.14222
C -4.91332 -0.87789 1.56713
H -5.75631 -1.22576 0.96338
H -5.23152 -1.00920 2.60966
C -4.67159 0.61643 1.30611
H -5.63421 1.14821 1.31633
H -4.09529 1.02858 2.14756
B 2.68136 0.11276 -0.00008

3j-[H-BBN]
B3PW91
SCF = -1262.95094768
SCF (C6H5Cl) = -1262.95470943
SCF (D3BJ) = -1263.15418415
SCF (BS2) = -1263.31667651
H (0 K) = -1262.211717
H (298 K) = -1262.180353
G (298 K) = -1262.272258
Low freq. = 17.6574
Second freq. = 20.1002

81
3j-[H-BBN]
N -1.83630 1.06543 -0.00000
C -0.53065 1.61679 -0.00000
C -1.77256 -0.26744 -0.00000
H -2.67667 -0.86229 -0.00000
C -0.43045 -0.70768 -0.00000
C 0.31361 0.51731 0.00000
H 1.39262 0.58178 0.00000
C -3.08663 1.80303 -0.00000
H -3.16209 2.43195 0.88978
H -3.91364 1.09109 -0.00000
C -1.11581 -2.75062 1.82449
H -2.05252 -2.90677 1.26860
C -0.60422 -4.15484 2.22398
H -0.56296 -4.76459 1.30983
H -1.33134 -4.65374 2.88167
C 0.77920 -4.18724 2.89741
H 0.67693 -3.92237 3.95396
H 1.14913 -5.22114 2.89714
C 1.83518 -3.28075 2.24032
H 2.20195 -3.77456 1.32878
H 2.70534 -3.20682 2.90919
C 1.34324 -1.86959 1.84113
H 2.16915 -1.39092 1.29540
C 0.99665 -0.95864 3.03863
H 0.81283 0.05199 2.64740
H 1.86182 -0.86708 3.71172

C -0.22881 -1.39204 3.86278
H 0.05542 -2.19810 4.54567
H -0.53167 -0.56065 4.51342
C -1.44132 -1.83481 3.02453
H -2.17437 -2.31852 3.68700
H -1.93982 -0.93442 2.63586
C 1.42932 3.49342 0.00000
H 2.10093 2.62591 0.00000
C 1.71696 4.27979 1.30525
H 2.76243 4.61973 1.31247
H 1.62721 3.57530 2.14446
C 0.79030 5.47616 1.56691
H 1.10816 6.33055 0.96300
H 0.90963 5.79885 2.60925
C -0.69426 5.18128 1.30617
H -1.26080 6.12349 1.31410
H -1.08626 4.59171 2.14800
C -1.00218 4.40020 -0.00000
H -2.08140 4.21413 -0.00000
B 0.02778 -2.05085 0.93746
H 0.34798 -2.92447 0.00000
B -0.10358 3.09566 -0.00000
H -3.16209 2.43195 -0.88978
C -1.11581 -2.75062 -1.82449
H -2.05252 -2.90677 -1.26860
C -0.60422 -4.15484 -2.22398
H -0.56296 -4.76459 -1.30983
H -1.33134 -4.65374 -2.88167
C 0.77920 -4.18724 -2.89741
H 0.67693 -3.92237 -3.95396
H 1.14913 -5.22114 -2.89714
C 1.83518 -3.28075 -2.24032
H 2.20195 -3.77456 -1.32878
H 2.70534 -3.20682 -2.90919
C 1.34324 -1.86959 -1.84113
H 2.16915 -1.39092 -1.29540
C 0.99665 -0.95864 -3.03863
H 0.81283 0.05199 -2.64740
H 1.86182 -0.86708 -3.71172
C -0.22881 -1.39204 -3.86278
H 0.05542 -2.19810 -4.54567
H -0.53167 -0.56065 -4.51342
C -1.44132 -1.83481 -3.02453
H -2.17437 -2.31852 -3.68700
H -1.93982 -0.93442 -2.63586
C 1.71696 4.27979 -1.30525
H 2.76243 4.61973 -1.31247
H 1.62721 3.57530 -2.14446
C 0.79030 5.47616 -1.56691
H 1.10816 6.33055 -0.96300
H 0.90963 5.79885 -2.60925
C -0.69426 5.18128 -1.30617
H -1.26080 6.12349 -1.31410
H -1.08626 4.59171 -2.14800
B 0.02778 -2.05085 -0.93746

NacNacZnH, **1**
B3PW91
SCF = -1466.70868636
SCF (C6H5Cl) = -1466.71954089
SCF (D3BJ) = -1466.89317166
SCF (BS2) = -3019.31239925
H (0 K) = -1466.064488
H (298 K) = -1466.026563
G (298 K) = -1466.134968
Low freq. = 15.7955
Second freq. = 18.8326

74
NacNacZnH, **1**
Zn -0.00000 0.00032 -0.80341
N 1.48069 -0.00008 0.51108
N -1.48068 -0.00011 0.51110
C -1.27298 -0.00048 1.82923
C 0.00002 -0.00062 2.42675
C 1.27301 -0.00046 1.82921
H 0.00003 -0.00092 3.51008
C -2.46228 -0.00080 2.76105
H -3.09338 -0.87735 2.58475
H -3.09369 0.87557 2.58499
H -2.14657 -0.00090 3.80530
C -2.81593 -0.00001 -0.00659
C -3.45158 -1.22802 -0.29758
C -3.45160 1.22812 -0.29703
C -4.72656 1.20082 -0.86985
C -5.36603 0.00023 -1.15424
C -4.72653 -1.20048 -0.87039
C -2.77110 2.56553 -0.04488
H -5.22429 2.13871 -1.10245
H -6.35672 0.00032 -1.60065
H -5.22425 -2.13828 -1.10341
C -2.77104 -2.56552 -0.04606
C -2.33557 3.20944 -1.37030
H -1.67766 2.54482 -1.93906
H -1.80121 4.14879 -1.18875
H -3.20349 3.43317 -2.00075
C -3.64429 3.53100 0.76775
H -1.86601 2.37213 0.53984
H -4.54419 3.82728 0.21800
H -3.08704 4.44585 0.99688

H -3.96705 3.08659 1.71483
C -2.33537 -3.20872 -1.37178
H -1.80097 -4.14814 -1.19068
H -1.67746 -2.54376 -1.94014
H -3.20323 -3.43216 -2.00240
C -3.64423 -3.53146 0.76601
H -1.86599 -2.37238 0.53883
H -4.54405 -3.82755 0.21601
H -3.96712 -3.08756 1.71328
H -3.08692 -4.44639 0.99474
C 2.46232 -0.00080 2.76101
H 2.14663 -0.00062 3.80526
H 3.09391 0.87540 2.58475
H 3.09324 -0.87752 2.58489
H 3.20348 3.43316 -2.00094
H 3.08695 4.44592 0.99665
H 1.80117 4.14879 -1.18900
C 3.64421 3.53105 0.76757
C 2.33555 3.20944 -1.37051
H 3.96692 3.08668 1.71468
H 1.67766 2.54479 -1.93927
C 2.77105 2.56556 -0.04507
H 1.86594 2.37216 0.53963
H 4.54411 -3.82753 0.21600
H 4.54413 3.82730 0.21784
C 3.64434 -3.53142 0.76606
H 3.96731 -3.08749 1.71329
C 2.81593 0.00002 -0.00663
C 2.77109 -2.56549 -0.04596
C 3.45156 1.22815 -0.29716
C 3.45160 -1.22799 -0.29757
C 4.72655 -1.20046 -0.87039
C 4.72651 1.20084 -0.87000
C 5.36601 0.00025 -1.15433
H 5.22429 -2.13825 -1.10337
H 5.22422 2.13873 -1.10266
H 1.86608 -2.37235 0.53898
H 6.35669 0.00034 -1.60075
H 3.08705 -4.44633 0.99486
H 3.20316 -3.43216 -2.00232
C 2.33534 -3.20872 -1.37164
H 1.80096 -4.14814 -1.19050
H 1.67738 -2.54378 -1.93998
H -0.00006 0.00081 -2.34038

10
B3PW91
SCF = -1669.81223588
SCF (C6H5Cl) = -1669.82254931
SCF (D3BJ) = -1670.03465283
SCF (BS2) = -3610.43032961
H (0 K) = -1669.089536
H (298 K) = -1669.045077
G (298 K) = -1669.168975
Low freq. = 16.1431
Second freq. = 18.4574

84
10
Zn -0.04311 0.07951 0.00003
N -1.59848 -1.13975 0.00025
N 1.36215 -1.29783 0.00015
C 1.08079 -2.60291 0.00029
C -0.22217 -3.13071 0.00042
C -1.46190 -2.46659 0.00040
H -0.27999 -4.21243 0.00054
C 2.21738 -3.59767 0.00026
H 2.85740 -3.45589 0.87635
H 2.85665 -3.45662 -0.87649
H 1.84493 -4.62296 0.00082
C 2.72496 -0.85378 0.00005
C 3.37509 -0.59944 1.22907
C 3.37501 -0.59968 -1.22906
C 4.67653 -0.09008 -1.20100
C 5.32796 0.16503 -0.00013
C 4.67661 -0.08985 1.20083
C 2.69490 -0.84027 -2.56940
H 5.18641 0.11455 -2.13890
H 6.33922 0.56263 -0.00021
H 5.18656 0.11495 2.13865
C 2.69510 -0.83983 2.56951
C 2.38128 0.48703 -3.27682
C 3.51691 -1.75880 -3.48477
H 4.46595 -1.29573 -3.77592
H 2.96249 -1.97373 -4.40479
H 3.75041 -2.71298 -3.00141
C 2.38180 0.48756 3.27691
H 1.85198 0.30654 4.21896
H 1.76153 1.13863 2.65369
H 3.30197 1.03478 3.51052
C 3.51707 -1.75846 3.48481
H 1.74191 -1.34119 2.37286
H 4.46626 -1.29557 3.77575
H 3.75027 -2.71274 3.00149

H 2.96277 -1.97320 4.40495
C -2.69907 -3.33311 0.00048
H -3.31939 -3.12372 -0.87628
H -3.31993 -3.12285 0.87664
H -2.43985 -4.39273 0.00106
H -3.27169 1.40765 -3.50512
H -3.24506 -1.60815 -4.41349
H -1.89507 0.54200 -4.20372
C -3.77558 -1.34506 -3.49191
C -2.41306 0.77020 -3.26543
H -4.10577 -2.27424 -3.01631
H -1.73625 1.35099 -2.63150
C -2.86499 -0.52284 -2.56913
H -1.96965 -1.12156 -2.37312
H -4.67142 -0.78332 3.78017
H -4.67201 -0.78504 -3.77956
C -3.77490 -1.34331 3.49277
H -4.10492 -2.27281 3.01768
C -2.90221 -0.54708 0.00019
C -2.86454 -0.52139 2.56949
C -3.51917 -0.21929 -1.22882
C -3.51896 -0.21859 1.22911
C -4.75610 0.43234 1.20093
C -4.75631 0.43166 -1.20079
C -5.37614 0.75666 0.00004
H -5.23888 0.69425 2.13900
H -5.23926 0.69304 -2.13891
H -1.96915 -1.12007 2.37364
H -6.33711 1.26394 -0.00002
H -3.24426 -1.60580 4.41445
H -3.27141 1.40949 3.50462
C -2.41272 0.77206 3.26510
H -1.89456 0.54438 4.20343
H -1.73609 1.35263 2.63079
H 3.30133 1.03440 -3.51055
H 1.76097 1.13802 -2.65356
H 1.85139 0.30589 -4.21880
H 1.74182 -1.34178 -2.37261
H 1.92490 5.92173 0.88234
S 1.42212 2.96962 -0.00024
C -0.03879 2.01108 -0.00026
C -1.11874 2.87180 -0.00054
C 1.28695 5.80256 -0.00098
C 0.57061 4.48876 -0.00078
C -0.78057 4.25541 -0.00083
H 1.92624 5.92074 -0.88346
H -2.14551 2.51777 -0.00058
H -1.51351 5.05714 -0.00111
H 0.56278 6.62229 -0.00199

NacNacZn[DMT], **[9]**+
B3PW91
SCF = -1871.32848514
SCF (C6H5Cl) = -1871.37991677
SCF (D3BJ) = -1871.58961374
SCF (BS2) = -3424.05319612
H (0 K) = -1870.484560
H (298 K) = -1870.436322
G (298 K) = -1870.567681
Low freq. = 10.7559
Second freq. = 13.6557

96
NacNacZn[DMT], **[9]**+
Zn -0.28248 -0.22290 -0.17866
N -2.00247 -0.56926 0.62904
N 0.92414 -1.40113 0.74696
C 0.40712 -2.18597 1.70724
C -0.94913 -2.21747 2.07160
C -2.05125 -1.47303 1.62069
H -1.17810 -2.91352 2.87002
C 1.31798 -3.08428 2.50923
H 2.21928 -3.36244 1.96275
H 0.78759 -3.98879 2.81357
H 1.63340 -2.56792 3.42264
C 2.33949 -1.39225 0.49832
C 3.19042 -0.63428 1.33317
C 2.84079 -2.09918 -0.62197
C 4.21211 -2.02190 -0.88732
C 5.06341 -1.27336 -0.08038
C 4.55253 -0.59053 1.01592
C 1.93797 -2.95656 -1.50214
H 4.62437 -2.55962 -1.73495
H 6.12574 -1.23184 -0.30316
H 5.22488 -0.01366 1.64460
C 2.68583 0.13617 2.54712
C 2.40776 -3.04796 -2.95944
H 2.64482 -2.06923 -3.39002
H 1.63065 -3.51209 -3.57455
H 3.30170 -3.67211 -3.05754
C 1.76769 -4.37385 -0.92980
H 0.94024 -2.49414 -1.50020
H 2.73884 -4.87282 -0.84591
H 1.13483 -4.97814 -1.58812
H 1.30533 -4.36583 0.05957
C 2.69504 1.65194 2.29892
H 2.30718 2.18351 3.17431
H 2.08670 1.92937 1.43273
H 3.71167 2.01395 2.11323
C 3.48222 -0.19867 3.81738

H 1.64599 -0.15492 2.72640
H 4.51115 0.17066 3.75938
H 3.53065 -1.27636 3.99962
H 3.01844 0.27366 4.68924
C -3.34794 -1.70123 2.35792
H -3.39330 -2.71772 2.75283
H -4.22193 -1.51747 1.73256
H -3.40821 -1.01471 3.21041
H -4.72356 -1.41884 -3.45936
H -4.15885 -3.71837 -1.63606
H -3.28440 -2.41754 -3.60281
C -4.42697 -2.79767 -1.10748
C -3.68342 -1.51314 -3.13246
H -4.29619 -2.97744 -0.03769
H -3.13761 -0.65310 -3.53560
C -3.57041 -1.62137 -1.60520
H -2.52627 -1.85899 -1.36335
H -4.39295 2.95831 3.12022
H -5.48978 -2.60404 -1.28725
C -3.71930 2.11759 3.31441
H -4.33906 1.24805 3.55236
C -3.18728 0.14139 0.23166
C -2.78919 1.84539 2.12273
C -3.92670 -0.32803 -0.88053
C -3.55104 1.32591 0.90956
C -4.65727 2.04157 0.43775
C -5.03329 0.41923 -1.29703
C -5.39471 1.59745 -0.65280
H -4.95186 2.95804 0.94138
H -5.62357 0.07408 -2.14056
H -2.07880 1.07200 2.43252
H -6.25554 2.16369 -0.99618
H -3.13217 2.37075 4.20294
H -2.62085 3.90952 1.42280
C -1.97428 3.10335 1.78579
H -1.44898 3.47165 2.67304
H -1.21810 2.90859 1.01651
H -1.64053 2.27728 -1.18668
C -1.30575 1.72289 -2.06263
H -1.19847 2.40351 -2.91434
H -2.06402 0.97529 -2.29089
H 4.82683 4.98932 -1.95090
C 4.39765 4.72222 -0.97797
C 2.40300 1.51036 -1.49012
C 1.08822 1.98412 -1.54758
C 3.45801 2.39505 -1.29983
C 0.85547 3.35116 -1.38313
C 3.24609 3.77052 -1.14808
C 1.92568 4.22252 -1.18021
N -0.01560 1.03220 -1.79538
H 2.62629 0.45195 -1.57713
H 4.46789 1.99693 -1.26112
H -0.14324 3.76765 -1.41565
H 1.71728 5.28177 -1.05565
H 4.08427 5.64988 -0.49248
H 5.19969 4.27863 -0.38123
C 0.28554 0.19336 -2.99479
H -0.54553 -0.49495 -3.16523
H 0.41008 0.83681 -3.87248
H 1.19539 -0.38276 -2.84434

TS1[CHZn]
B3PW91
SCF = -2075.60146632
SCF (C6H5Cl) = -2075.64714236
SCF (D3BJ) = -2075.91311696
SCF (BS2) = -4016.34101563
H (0 K) = -2074.661876
H (298 K) = -2074.607018
G (298 K) = -2074.751179
Low freq. = -25.4978
Second freq. = 15.6725

108
TS1[CHZn]
Zn 0.12277 -0.01632 0.01204
N 1.53591 -1.10382 -0.85209
N -1.15589 0.34513 -1.42642
C -0.61467 0.27485 -2.65718
C 0.64722 -0.26140 -2.95595
C 1.56096 -1.01085 -2.18536
H 0.89861 -0.22214 -4.01073
C -1.36180 0.81262 -3.85980
H -0.83474 1.69890 -4.23013
H -2.39033 1.09100 -3.63732
H -1.35792 0.08503 -4.67448
C -2.56956 0.59591 -1.29127
C -3.03612 1.88675 -0.94505
C -3.48310 -0.46814 -1.51339
C -4.85224 -0.20027 -1.40102
C -5.32201 1.06514 -1.07675
C -4.41603 2.09275 -0.84798
C -3.05578 -1.87806 -1.91358
H -5.56409 -1.00044 -1.58050
H -6.38975 1.25091 -1.00553
H -4.78885 3.08125 -0.59825
C -2.10064 3.06728 -0.72629
C -3.75578 -2.97475 -1.09322
C -3.31833 -2.14346 -3.40627
H -4.39015 -2.08462 -3.62375

H -2.97754 -3.14709 -3.68111
H -2.81160 -1.42626 -4.05364
C -2.54296 3.97492 0.43162
H -1.73941 4.67083 0.69393
H -2.81766 3.40679 1.32612
H -3.41007 4.58392 0.15688
C -1.94509 3.91552 -1.99913
H -1.10921 2.66516 -0.48536
H -2.91291 4.32341 -2.30973
H -1.54574 3.33615 -2.83368
H -1.26835 4.75720 -1.81749
C 2.61597 -1.72846 -2.99528
H 2.15340 -2.23387 -3.84676
H 3.17170 -2.45876 -2.40875
H 3.32443 -0.99940 -3.40183
H 0.67753 -5.73252 0.92102
H -0.33583 -4.78653 -1.61788
H -0.90928 -4.98042 0.83422
C 0.65122 -4.36661 -1.39727
C 0.14999 -4.77785 1.01780
H 0.88479 -3.63688 -2.17488
H 0.25654 -4.44795 2.05543
C 0.66863 -3.74537 0.00994
H -0.03651 -2.90268 -0.00309
H 6.41369 -0.93376 -0.93447
H 1.38358 -5.17800 -1.46422
C 5.55248 -0.52598 -1.47379
H 5.27238 -1.25547 -2.23678
C 2.48325 -1.93155 -0.14838
C 4.39869 -0.20715 -0.50500
C 2.04532 -3.18628 0.34781
C 3.80854 -1.48560 0.08307
C 4.65108 -2.29427 0.85707
C 2.93396 -3.94940 1.10988
C 4.22450 -3.50698 1.37666
H 5.66953 -1.96555 1.04400
H 2.61586 -4.91200 1.49582
H 3.61254 0.30916 -1.06577
H 4.89842 -4.11523 1.97282
H 5.88644 0.38607 -1.97962
H 5.71259 0.30873 1.16568
C 4.91472 0.76248 0.56886
H 5.32908 1.65807 0.09538
H 4.12799 1.08931 1.25195
H -4.82106 -3.03880 -1.33612
H -3.67705 -2.81748 -0.01445
H -3.31871 -3.95075 -1.32817
H -1.97526 -1.96551 -1.74681
H -3.99954 1.16435 4.66630
S -3.37372 -0.42521 2.10250
C -2.00076 -1.40575 1.69355
C -1.34871 -1.84201 2.82282
C -3.92919 0.08268 4.82148
C -3.04985 -0.57434 3.80526
C -1.93651 -1.35249 4.02083
H -1.86936 -1.73617 0.67128
H -4.94704 -0.32006 4.79066
H -0.50225 -2.51842 2.79717
H -1.56633 -1.58167 5.01453
H -3.53153 -0.08965 5.82451
H 2.05869 3.06508 3.06472
C 2.15643 3.19289 1.99089
H -1.00353 2.30922 1.78128
C -0.38363 1.67365 2.41017
H -0.02595 2.25715 3.26472
C 1.55967 2.29168 1.10275
N 0.76556 1.15866 1.61563
C 1.59061 0.28303 2.49642
H 1.97308 0.84517 3.35430
H 0.96072 -0.52838 2.86511
H 2.42489 -0.13353 1.93333
H -0.96871 0.83197 2.77944
H 3.94253 5.64253 -1.46007
H 3.34364 4.95815 2.21646
C 2.88767 4.27111 1.50826
C 3.83155 5.66933 -0.37354
H 4.83283 5.69806 0.06833
C 3.04728 4.49015 0.13261
H 3.33680 6.61099 -0.11097
C 2.44713 3.58150 -0.74084
C 1.71122 2.49479 -0.26565
H 2.55112 3.71813 -1.81371
H 1.26830 1.81852 -0.99507

INT-1[CHZn]
B3PW91
SCF = -2075.60194019
SCF (C6H5Cl) = -2075.64450393
SCF (D3BJ) = -2075.91594219
SCF (BS2) = -4016.34068856
H (0 K) = -2074.661969
H (298 K) = -2074.606347
G (298 K) = -2074.752979
Low freq. = 11.2828
Second freq. = 21.1629

108
INT-1[CHZn]
Zn 0.04581 -0.00374 0.19134
N 1.49336 -0.92851 -0.84824
N -1.32188 0.36152 -1.20757
C -0.84940 0.39215 -2.46157
C 0.44854 0.00986 -2.84251
C 1.44090 -0.73456 -2.17017
H 0.64353 0.13269 -3.90313
C -1.71954 0.85598 -3.61046
H -1.27568 1.75557 -4.05031
H -2.74086 1.08270 -3.30882
H -1.74289 0.10140 -4.40061
C -2.74123 0.45170 -0.97605
C -3.31932 1.67040 -0.54086
C -3.55186 -0.69820 -1.16921
C -4.92135 -0.60272 -0.89521
C -5.49365 0.58065 -0.45077
C -4.69367 1.70399 -0.28400
C -3.02746 -2.01920 -1.72459
H -5.55306 -1.47313 -1.04717
H -6.55989 0.63279 -0.25048
H -5.14974 2.63363 0.04172
C -2.51865 2.95855 -0.40910
C -3.43467 -3.23525 -0.87703
C -3.51321 -2.24187 -3.16799
H -4.60232 -2.35361 -3.19489
H -3.07545 -3.15578 -3.58305
H -3.25370 -1.41126 -3.82686
C -2.95977 3.82476 0.78112
H -2.22013 4.60926 0.97133
H -3.09411 3.24390 1.69941
H -3.90925 4.33060 0.57862
C -2.59337 3.80609 -1.69066
H -1.46648 2.68338 -0.27061
H -3.63066 4.08109 -1.90991
H -2.19765 3.27723 -2.55918
H -2.01840 4.73020 -1.56934
C 2.46430 -1.35134 -3.09846
H 1.96286 -2.03526 -3.78950
H 3.24872 -1.89623 -2.57628
H 2.92020 -0.56938 -3.71326
H 0.79013 -5.66594 0.49637
H 0.26059 -4.99863 -2.41462
H -0.76145 -5.12037 -0.12123
C 1.18432 -4.59716 -1.98467
C 0.18727 -4.77386 0.30038
H 1.66489 -3.96978 -2.73764
H -0.02690 -4.30277 1.26253
C 0.88571 -3.82632 -0.68619
H 0.19059 -3.01656 -0.93833
H 5.96965 -0.41615 -1.30938
H 1.85323 -5.44088 -1.78433
C 5.08529 0.22572 -1.38434
H 4.54849 -0.05179 -2.29122
C 2.48046 -1.82339 -0.28832
C 4.22068 0.10147 -0.11668
C 2.16045 -3.19723 -0.13284
C 3.75156 -1.33339 0.11488
C 4.64961 -2.22827 0.70808
C 3.09897 -4.03938 0.47435
C 4.33111 -3.56531 0.90192
H 5.62541 -1.86730 1.01813
H 2.86518 -5.09303 0.59478
H 3.33379 0.72856 -0.26150
H 5.04658 -4.23753 1.36648
H 5.43086 1.25809 -1.50300
H 6.05289 0.27125 1.06929
C 5.03469 0.67335 1.05863
H 5.12300 1.75917 0.95529
H 4.59537 0.45948 2.03680
H -4.51684 -3.39680 -0.90191
H -3.14641 -3.13930 0.17324
H -2.96884 -4.14214 -1.27594
H -1.93237 -1.96989 -1.74727
H -3.17887 0.35419 4.98370
S -2.67382 -0.99089 2.24758
C -1.18022 -1.60070 1.57775
C -0.29250 -1.93016 2.59114
C -2.90335 -0.70425 5.03803
C -2.07288 -1.12337 3.86730
C -0.78756 -1.62686 3.88030
H -1.19783 -2.02802 0.58020
H -3.83179 -1.28149 5.09485
H 0.66641 -2.40220 2.40141
H -0.23288 -1.79204 4.79777
H -2.34949 -0.86361 5.96593
H 1.80338 3.69544 2.72085
C 1.83253 3.70424 1.63526
H -1.13593 2.40080 1.86081
C -0.37981 1.89406 2.45496
H -0.02776 2.57342 3.23880
C 1.32766 2.63048 0.89335
N 0.74045 1.46436 1.57928
C 1.76177 0.80076 2.43095
H 2.20809 1.51181 3.13384
H 1.28186 0.00312 2.99862
H 2.54155 0.37622 1.80456
H -0.81461 1.01371 2.92904
H 3.13858 5.91890 -2.16910

H 2.76986 5.62724 1.58736
C 2.38370 4.80474 0.99061
C 3.04083 6.07670 -1.09240
H 4.03230 6.31461 -0.69380
C 2.45218 4.87369 -0.40798
H 2.41212 6.96126 -0.94042
C 1.94820 3.79289 -1.13333
C 1.39085 2.68320 -0.49562
H 1.98815 3.80877 -2.21911
H 1.01867 1.86919 -1.11325

TS2[CHZn]
B3PW91
SCF = -2075.58656011
SCF (C6H5Cl) = -2075.63541790
SCF (D3BJ) = -2075.88918829
SCF (BS2) = -4016.32612414
H (0 K) = -2074.648187
H (298 K) = -2074.592674
G (298 K) = -2074.742168
Low freq. = -52.0909
Second freq. = 11.2190

108
TS2[CHZn]
Zn 0.41314 -0.17774 0.27886
N 1.77757 0.62855 -0.87104
N -0.84379 -1.08609 -0.88724
C -0.79896 -0.64747 -2.15579
C 0.17288 0.23274 -2.67019
C 1.40579 0.68973 -2.16046
H 0.02544 0.47429 -3.71776
C -1.84367 -1.09713 -3.15001
H -2.55716 -0.27722 -3.29188
H -2.39566 -1.97296 -2.81132
H -1.39494 -1.31009 -4.12261
C -1.58500 -2.27901 -0.54764
C -2.86247 -2.20267 0.05525
C -0.99533 -3.54166 -0.81538
C -1.70128 -4.69870 -0.47053
C -2.95310 -4.63531 0.12491
C -3.52007 -3.39486 0.37942
C 0.35143 -3.71076 -1.50527
H -1.26423 -5.66955 -0.68489
H -3.48772 -5.54617 0.37816
H -4.50815 -3.34736 0.82673
C -3.59043 -0.89241 0.30016
C 1.28446 -4.64876 -0.72681
C 0.17962 -4.22429 -2.94444
H -0.28133 -5.21764 -2.95186
H 1.15099 -4.30355 -3.44378
H -0.45382 -3.56296 -3.54078
C -4.20917 -0.82238 1.70318
H -4.63627 0.17060 1.87548
H -3.47315 -1.03037 2.48701
H -5.02637 -1.54035 1.82419
C -4.69053 -0.67230 -0.75144
H -2.86721 -0.07614 0.19994
H -5.45866 -1.44999 -0.67676
H -4.29390 -0.69619 -1.76866
H -5.17044 0.29916 -0.60110
C 2.33560 1.26192 -3.20710
H 2.45148 0.55254 -4.03097
H 3.32073 1.50638 -2.81384
H 1.89220 2.16927 -3.63042
H 5.35750 -2.64820 0.27891
H 3.92091 -3.00265 -2.36674
H 3.88682 -3.59386 0.12518
C 4.19472 -1.97080 -2.12328
C 4.26592 -2.58721 0.32005
H 3.76441 -1.31713 -2.88654
H 3.99518 -2.31196 1.34497
C 3.71090 -1.59556 -0.71318
H 2.61752 -1.68360 -0.71610
H 4.00960 4.78892 -1.34770
H 5.28458 -1.88853 -2.19155
C 3.00147 4.37801 -1.46602
H 2.96815 3.86062 -2.42695
C 3.14191 0.91858 -0.48761
C 2.64414 3.45442 -0.28960
C 4.06675 -0.15257 -0.38643
C 3.56017 2.24357 -0.21101
C 4.89711 2.46532 0.13928
C 5.38821 0.12993 -0.02452
C 5.81052 1.42635 0.23327
H 5.22780 3.48096 0.33536
H 6.10491 -0.68272 0.04032
H 1.62456 3.08702 -0.44412
H 6.84415 1.62470 0.50094
H 2.30484 5.22170 -1.51293
H 3.64947 4.75362 1.15300
C 2.68006 4.26458 1.01606
H 1.92406 5.05603 1.00100
H 2.50539 3.63628 1.89459
H 0.89783 -5.67187 -0.69478
H 1.42541 -4.32400 0.30972
H 2.26588 -4.69457 -1.20903
H 0.83361 -2.72910 -1.56341
H -1.66485 -0.09732 5.29901
S -0.25802 -1.76096 3.09651

C 1.06533 -1.27476 2.05081
C 1.82539 -0.27516 2.67769
C -0.64299 -0.41674 5.52904
C 0.16848 -0.58775 4.28656
C 1.28863 0.13592 3.90828
H 1.48012 -2.03276 1.38766
H -0.71091 -1.35304 6.09166
H 2.74635 0.10877 2.24832
H 1.71209 0.92153 4.52393
H -0.18721 0.33660 6.17493
H -4.11996 2.88178 1.70642
C -3.80577 2.90028 0.66836
H -2.63679 1.10328 2.53062
C -1.94845 1.94552 2.59872
H -2.45721 2.77939 3.10988
C -2.48565 2.61179 0.31343
N -1.45483 2.30869 1.27327
C -0.54665 3.45383 1.41522
H -1.05351 4.31603 1.87808
H 0.30101 3.17104 2.04329
H -0.17486 3.76378 0.43984
H -1.09516 1.65553 3.21665
H -6.42220 3.72623 -2.30398
H -5.76439 3.45847 -0.00518
C -4.74504 3.23460 -0.31043
C -5.40826 3.69672 -2.71082
H -5.40413 2.99643 -3.55264
C -4.40317 3.30482 -1.66142
H -5.18643 4.69021 -3.11755
C -3.07673 3.00630 -2.00685
C -2.13793 2.66189 -1.04349
H -2.77569 3.04179 -3.05155
H -1.12200 2.41709 -1.34327

INT-2[CHZn]
B3PW91
SCF = -1670.21252981
SCF (C6H5Cl) = -1670.26421089
SCF (D3BJ) = -1670.44149696
SCF (BS2) = -3610.82592447
H (0 K) = -1669.477997
H (298 K) = -1669.433243
G (298 K) = -1669.558806
Low freq. = 9.7570
Second freq. = 17.1821

85
INT-2[CHZn]
Zn 0.07322 0.15342 -0.16610
N 1.65716 0.54262 0.83820
N -1.37567 0.65425 0.97697
C -1.06281 1.19790 2.16431
C 0.25073 1.41092 2.62088
C 1.49875 1.10745 2.04495
H 0.31295 1.87176 3.59908
C -2.17954 1.59410 3.09574
H -2.61627 0.70354 3.56104
H -2.98696 2.09801 2.56015
H -1.81516 2.24582 3.89043
C -2.74023 0.44178 0.58132
C -3.39443 -0.75094 0.96457
C -3.37431 1.39245 -0.25080
C -4.67014 1.11520 -0.69999
C -5.32178 -0.06043 -0.34736
C -4.68567 -0.97967 0.47972
C -2.71618 2.70777 -0.64602
H -5.17997 1.83785 -1.33127
H -6.32844 -0.25466 -0.70597
H -5.20637 -1.89058 0.76131
C -2.73509 -1.78864 1.86346
C -2.63223 2.88087 -2.16981
C -3.44039 3.91014 -0.01925
H -4.47113 3.98380 -0.38111
H -2.92918 4.84309 -0.27813
H -3.47718 3.84004 1.07158
C -2.28295 -3.01480 1.05597
H -1.79116 -3.74753 1.70427
H -1.57614 -2.73551 0.26581
H -3.13855 -3.50699 0.58050
C -3.63778 -2.21761 3.02865
H -1.83576 -1.33881 2.29639
H -4.51415 -2.77511 2.68302
H -3.99966 -1.35694 3.59923
H -3.08741 -2.87088 3.71318
C 2.71938 1.42374 2.87055
H 3.54641 1.77567 2.25094
H 3.06672 0.52112 3.38561
H 2.49342 2.17430 3.62908
H 3.72721 2.69857 -3.00304
H 3.28148 4.58857 -0.62963
H 2.22999 3.57807 -2.70523
C 3.75457 3.62986 -0.39332
C 2.76228 2.64612 -2.48912
H 3.84585 3.56004 0.69424
H 2.20157 1.82134 -2.94308
C 2.93466 2.46630 -0.97369

H 1.93991 2.51168 -0.51519
H 4.78583 -2.94651 2.37163
H 4.76658 3.64453 -0.81113
C 3.94407 -2.36285 2.75783
H 4.35836 -1.48392 3.26075
C 2.95655 0.23184 0.31066
C 2.96215 -1.97410 1.64276
C 3.55215 1.12105 -0.61478
C 3.57465 -0.99294 0.65200
C 4.78975 -1.31210 0.03703
C 4.77172 0.75269 -1.19352
C 5.38674 -0.45352 -0.87862
H 5.27964 -2.24973 0.28444
H 5.25148 1.42705 -1.89746
H 2.10351 -1.48667 2.11596
H 6.33367 -0.71953 -1.33921
H 3.43968 -2.97812 3.50972
H 3.25005 -3.76611 0.42710
C 2.43814 -3.23062 0.93102
H 1.98062 -3.91886 1.64934
H 1.68501 -2.98214 0.17528
H -3.62596 2.95294 -2.62297
H -2.11974 2.04127 -2.65250
H -2.09099 3.79842 -2.42267
H -1.69333 2.70349 -0.25246
H -1.88517 -4.28081 -2.04393
S -1.54883 -1.11466 -2.58895
C -0.03946 -0.26868 -2.24444
C 1.03891 -1.17326 -2.36215
C -1.50114 -3.88574 -2.99137
C -0.74051 -2.62207 -2.76138
C 0.63438 -2.49011 -2.60080
H 0.02449 0.77412 -2.55606
H -2.35341 -3.73527 -3.65870
H 2.07424 -0.85564 -2.27715
H 1.31443 -3.33108 -2.67356
H -0.84623 -4.64224 -3.42974

TS3[CHZn]
B3PW91
SCF = -2075.59548460
SCF (C6H5Cl) = -2075.64211720
SCF (D3BJ) = -2075.89378481
SCF (BS2) = -4016.33300298
H (0 K) = -2074.661001
H (298 K) = -2074.605076
G (298 K) = -2074.754995
Low freq. = -1078.5003
Second freq. = 13.8172

108
TS3[CHZn]
Zn 0.78867 0.28798 -0.22042
N 1.13550 2.19374 -0.52857
N 2.62345 -0.39070 -0.13360
C 3.65515 0.42714 -0.37431
C 3.52569 1.78639 -0.70491
C 2.39162 2.61161 -0.75101
H 4.46010 2.29533 -0.90923
C 5.05876 -0.11885 -0.29212
H 5.22535 -0.88269 -1.05712
H 5.23127 -0.60227 0.67354
H 5.79774 0.67148 -0.42637
C 2.86170 -1.76927 0.19250
C 2.98908 -2.72143 -0.84475
C 2.90238 -2.16157 1.54950
C 3.01367 -3.52396 1.84503
C 3.09769 -4.47680 0.83674
C 3.09643 -4.07087 -0.49330
C 2.85532 -1.15426 2.69064
H 3.04619 -3.84268 2.88360
H 3.18850 -5.53017 1.08658
H 3.19418 -4.81691 -1.27753
C 3.02830 -2.33039 -2.31645
C 1.57376 -1.29539 3.52394
C 4.09268 -1.25706 3.59532
H 4.13242 -2.21860 4.11741
H 4.07573 -0.47004 4.35656
H 5.02089 -1.15372 3.02549
C 1.75977 -2.77445 -3.05757
H 1.79627 -2.45778 -4.10566
H 0.85908 -2.35061 -2.60453
H 1.65797 -3.86534 -3.04451
C 4.27462 -2.88058 -3.02696
H 3.07174 -1.23785 -2.37285
H 4.25134 -3.97311 -3.09534
H 5.19921 -2.60569 -2.51015
H 4.33081 -2.49132 -4.04873
C 2.66575 4.07232 -1.03179
H 2.94498 4.58950 -0.10733
H 1.80084 4.58906 -1.44847
H 3.50694 4.16944 -1.72170
H -0.59072 3.43353 3.45264
H 2.17674 5.08115 3.16091
H 1.04594 2.93631 3.90563
C 1.37174 5.19726 2.42829
C 0.33743 2.99195 3.07260
H 1.74048 5.82999 1.61555
H 0.12504 1.96586 2.75199
C 0.91881 3.82144 1.91886

H 1.81029 3.29240 1.57077
H -0.11099 4.24441 -4.00181
H 0.56005 5.73844 2.92517
C 0.27742 3.24904 -3.76124
H 1.27744 3.36832 -3.33917
C 0.07555 3.15254 -0.42655
C -0.67031 2.51623 -2.79702
C -0.06068 3.92220 0.75390
C -0.84684 3.28417 -1.49308
C -1.90592 4.18637 -1.34607
C -1.14388 4.80363 0.84898
C -2.06127 4.93827 -0.18598
H -2.61600 4.31394 -2.15714
H -1.26361 5.40407 1.74637
H -0.19175 1.55876 -2.55099
H -2.88744 5.63784 -0.09616
H 0.37642 2.69037 -4.69793
H -2.43076 3.11030 -3.95732
C -1.99117 2.21115 -3.51351
H -1.81939 1.50712 -4.33395
H -2.74138 1.77868 -2.84207
H 1.50561 -2.28626 3.98544
H 0.67866 -1.16020 2.90724
H 1.55007 -0.55117 4.32742
H 2.85483 -0.15122 2.25111
H -0.59725 -5.23039 -0.81442
S -0.68034 -2.45063 0.50292
C -0.91138 -0.81734 -0.16112
C -1.52334 -0.99918 -1.42416
C -1.56862 -4.73080 -0.88508
C -1.41588 -3.24580 -0.83469
C -1.80501 -2.32357 -1.79068
H -1.82868 -0.19684 0.65675
H -2.18664 -5.09596 -0.05819
H -1.78057 -0.15497 -2.05626
H -2.28923 -2.61039 -2.71796
H -2.04146 -5.03167 -1.82255
H -7.53336 -3.77469 0.03326
H -2.12384 -1.21384 2.86401
H -3.22833 1.91005 0.28413
C -3.01909 1.64179 1.31747
C -2.39074 -0.15699 2.81258
C -7.45775 -2.78731 -0.42969
C -6.24920 -2.03816 0.06042
C -5.68887 -2.30089 1.31340
C -5.66133 -1.02753 -0.70884
C -4.56669 -0.30583 -0.24715
C -4.59328 -1.58477 1.79147
C -4.02242 -0.57422 1.01422
N -2.85675 0.17599 1.45642
H -6.06811 -0.79582 -1.68987
H -6.11965 -3.07689 1.94077
H -1.50324 0.43947 3.02628
H -2.08888 2.13137 1.60654
H -4.14631 0.46826 -0.87933
H -4.20855 -1.82615 2.77495
H -8.37774 -2.24169 -0.18840
H -7.43502 -2.92020 -1.51502
H -3.83615 1.99381 1.95503
H -3.15632 0.06777 3.56231

11

B3PW91
SCF = -1805.36202728
SCF (C6H5Cl) = -1805.37037399
SCF (D3BJ) = -1805.61775007
SCF (BS2) = -3358.06380135
H (0 K) = -1804.493848
H (298 K) = -1804.447047
G (298 K) = -1804.575136
Low freq. = 11.9809
Second freq. = 17.5060

98
11
Zn 0.00003 -0.14260 -0.00004
N -1.50398 -1.40487 -0.00973
N 1.50414 -1.40476 0.00988
C -2.45515 -3.66510 -0.02792
C -1.27685 -2.72070 -0.01045
C 0.00014 -3.31002 0.00022
C 1.27709 -2.72060 0.01083
C 2.45544 -3.66495 0.02859
C -2.84825 -0.90464 -0.00105
C -3.49713 -0.62723 -1.22548
C -4.77095 -0.05216 -1.18798
C -5.39912 0.23703 0.01729
C -4.75687 -0.05939 1.21324
C -3.48227 -0.63367 1.23310
C -2.85478 -0.92715 -2.57265
C -2.51194 0.36431 -3.32845
C -3.73260 -1.83963 -3.44204
C -2.83022 -0.94817 2.57282
C -3.68425 -1.90768 3.41565
C -2.52420 0.32935 3.36730
C 2.84837 -0.90445 0.00116
C 3.49721 -0.62676 1.22555
C 4.77099 -0.05164 1.18798
C 5.39920 0.23731 -0.01733
C 4.75700 -0.05937 -1.21324

C 3.48242 -0.63370 -1.23302
C 2.85482 -0.92641 2.57277
C 2.51203 0.36519 3.32834
C 3.73261 -1.83876 3.44232
C 2.83040 -0.94844 -2.57270
H 1.87691 -1.44717 -2.37082
C 3.68445 -1.90808 -3.41537
H 3.15799 -2.17594 -4.33791
H 3.91290 -2.83388 -2.87791
C 1.29748 2.99298 0.18489
C 1.46996 3.83952 -1.09486
C 0.22224 4.62085 -1.54491
C -1.09604 3.82977 -1.46840
C -1.29785 2.99273 -0.18525
C -1.47047 3.83946 1.09435
C -0.22289 4.62107 1.54427
C 1.09553 3.83019 1.46790
B -0.00011 2.05455 -0.00010
H -1.91551 -1.45698 -2.38323
H 0.00018 -4.39305 0.00030
H 1.91553 -1.45624 2.38344
H 0.13726 1.29436 -1.04279
H -0.13734 1.29447 1.04266
H -1.87672 -1.44691 2.37100
H 2.22563 2.41948 0.31773
H -5.27807 0.17469 -2.12225
H -4.66842 -1.34647 -3.72625
H -1.76597 3.15895 1.90535
H -1.93522 4.52805 -1.60848
H -2.30696 4.54558 0.97947
H 1.13445 3.14283 2.32535
H -2.22591 2.41906 -0.31800
H 5.27807 0.17543 2.12221
H -2.01313 0.13594 -4.27698
H -0.13283 5.53695 0.95220
H -5.25344 0.16121 2.15469
H 3.99468 -2.76814 2.92656
H -3.05498 -3.51660 -0.93089
H -0.37169 4.96389 2.57738
H 6.38742 0.68885 -0.02455
H -6.38735 0.68854 0.02445
H -1.13487 3.14226 -2.32574
H 0.13204 5.53681 -0.95300
H -3.99468 -2.76890 -2.92610
H -3.41549 0.94046 -3.55623
H 5.25359 0.16106 -2.15472
H 2.30633 4.54579 -0.98009
H -3.20777 -2.10369 -4.36656
H -3.15775 -2.17543 4.33821
H 1.93459 4.52863 1.60787
H 0.37099 4.96351 -2.57808
H -3.11964 -3.47689 0.81989
H -2.12785 -4.70494 0.00611
H 3.20778 -2.10264 4.36690
H 1.76557 3.15893 -1.90574
H 3.41560 0.94133 3.55607
H 2.12821 -4.70479 -0.00605
H -1.85061 1.00611 -2.74025
H -2.02571 0.08463 4.31191
H 1.85076 1.00693 2.74003
H 2.01317 0.13699 4.27690
H 4.66844 -1.34558 3.72644
H -3.91275 -2.83355 2.87834
H -4.63731 -1.45016 3.70233
H 3.12047 -3.47635 -0.81870
H 3.05467 -3.51679 0.93202
H -1.87389 1.00317 2.80321
H -3.44245 0.87652 3.60747
C 2.52437 0.32895 -3.36739
H 3.44261 0.87606 -3.60770
H 2.02580 0.08408 -4.31192
H 1.87411 1.00288 -2.80337
H 4.63754 -1.45062 -3.70207

TS1[DHC]
B3PW91
SCF = -1872.49082515
SCF (C6H5Cl) = -1872.54523528
SCF (D3BJ) = -1872.74140589
SCF (BS2) = -3425.21317622
H (0 K) = -1871.634813
H (298 K) = -1871.585201
G (298 K) = -1871.720690
Low freq. = -1141.1734
Second freq. = 8.9885

98
TS1[DHC]
Zn 1.55529 0.94310 0.86486
N 1.72461 -0.77650 -0.16764
N -0.24604 1.43167 0.10717
C -0.95779 0.64301 -0.65297
C -0.56892 -0.72331 -0.99649
C 0.78310 -1.26422 -0.93182
H -1.06072 -1.02585 -1.92453
C -2.26864 1.09713 -1.24086
H -2.08495 1.55903 -2.21791
H -2.75596 1.84565 -0.61416
H -2.94059 0.25137 -1.40085
C -0.67031 2.78545 0.37470

C -0.43470 3.79562 -0.58383
C -1.23076 3.08437 1.63471
C -1.59144 4.40852 1.89973
C -1.38992 5.41326 0.96224
C -0.80963 5.10363 -0.26115
C -1.45449 2.02843 2.70651
H -2.03309 4.65605 2.86097
H -1.67515 6.43647 1.18783
H -0.63599 5.89651 -0.98293
C 0.25977 3.53802 -1.91388
C -0.65577 2.33826 3.98103
H 0.41231 2.43718 3.76689
H -0.78567 1.53958 4.71936
H -0.99088 3.27144 4.44472
C -2.94755 1.85343 3.02322
H -1.08541 1.07300 2.31467
H -3.37455 2.77186 3.43832
H -3.09647 1.06263 3.76716
H -3.52830 1.60303 2.12843
C 1.67350 4.14114 -1.90520
H 2.19256 3.92225 -2.84421
H 2.27588 3.74836 -1.08008
H 1.63410 5.22940 -1.79204
C -0.54271 4.06631 -3.11178
H 0.36861 2.45550 -2.04344
H -0.60594 5.15893 -3.10418
H -1.56704 3.68057 -3.12359
H -0.05891 3.77726 -4.05038
C 1.04577 -2.46494 -1.80498
H 1.71290 -3.18082 -1.32088
H 1.54235 -2.13722 -2.72539
H 0.11730 -2.96074 -2.09458
H 3.68466 -2.40802 3.71584
H 1.34283 -4.35593 2.89008
H 1.97832 -2.21992 4.13669
C 2.12190 -4.13410 2.15236
C 2.72918 -2.00111 3.36944
H 1.83682 -4.59253 1.19916
H 2.84112 -0.91505 3.29605
C 2.32629 -2.61790 2.02095
H 1.36283 -2.17815 1.73398
H 5.16123 -0.74824 -3.69260
H 3.03434 -4.63421 2.49116
C 4.08793 -0.56329 -3.58450
H 3.57186 -1.50548 -3.79557
C 3.03249 -1.38674 -0.12590
C 3.74585 -0.01691 -2.19088
C 3.33448 -2.26465 0.93722
C 4.00407 -1.03026 -1.08507
C 5.27630 -1.59997 -0.97090
C 4.62217 -2.80430 1.00117
C 5.58749 -2.48264 0.05519
H 6.03998 -1.33882 -1.69781
H 4.87441 -3.48337 1.81074
H 2.67792 0.22996 -2.18900
H 6.58266 -2.91184 0.12302
H 3.80455 0.15955 -4.35630
H 5.59889 1.10414 -1.92131
C 4.51877 1.28311 -1.91606
H 4.30097 2.03282 -2.68343
H 4.26352 1.70689 -0.93936
H -2.57787 -0.80598 1.83292
C -2.17482 -1.81890 1.84488
H -2.75187 -2.43611 2.54030
H -1.13739 -1.78617 2.18326
H -7.68626 -3.32471 -2.34805
C -7.29073 -2.30574 -2.26356
C -3.56801 -2.67789 -1.53196
C -3.50383 -2.35936 -0.17105
C -4.78613 -2.64985 -2.20016
C -4.67264 -2.00935 0.50268
C -5.97298 -2.30514 -1.53930
C -5.88732 -1.98415 -0.18251
N -2.19741 -2.38168 0.48243
H -2.66990 -2.95093 -2.07877
H -4.81431 -2.90015 -3.25740
H -4.66361 -1.75648 1.55664
H -6.78794 -1.71023 0.36029
H -8.03805 -1.70581 -1.73858
H -7.18967 -1.91238 -3.27920
C -1.59888 -3.73899 0.48963
H -0.57499 -3.67421 0.86388
H -2.18214 -4.40322 1.13481
H -1.58842 -4.14930 -0.51917
H 2.46838 1.63131 1.87418
H -1.39253 -1.59230 -0.20022

INT-1[DHC]
B3PW91
SCF = -1872.50537218
SCF (C6H5Cl) = -1872.55991910
SCF (D3BJ) = -1872.74480964
SCF (BS2) = -3425.23073473
H (0 K) = -1871.646965
H (298 K) = -1871.595710
G (298 K) = -1871.738979
Low freq. = 8.7367
Second freq. = 9.8056

98
INT-1[DHC]

Zn	-2.07503	-0.00005	0.56013
N	-0.82996	-1.49155	-0.04302
N	-0.83005	1.49154	-0.04299
C	0.38063	1.33720	-0.45472
C	1.07364	0.00006	-0.49301
C	0.38066	-1.33710	-0.45491
H	1.80484	0.00001	0.33890
C	1.24995	2.46948	-0.90909
H	0.87784	3.43429	-0.56508
H	1.27456	2.48248	-2.00602
H	2.27765	2.31114	-0.56774
C	-1.44370	2.80251	-0.00242
C	-1.50211	3.46952	1.23798
C	-2.03841	3.32657	-1.16812
C	-2.67172	4.56931	-1.06514
C	-2.71981	5.26198	0.13811
C	-2.14190	4.71173	1.27561
C	-2.06317	2.58859	-2.49933
H	-3.13917	4.99895	-1.94631
H	-3.21398	6.22740	0.19090
H	-2.18999	5.25438	2.21521
C	-0.89720	2.90030	2.51365
C	-3.49065	2.13020	-2.83787
H	-3.91353	1.50343	-2.04610
H	-3.50070	1.55793	-3.77117
H	-4.15836	2.98803	-2.96638
C	-1.47586	3.42829	-3.64295
H	-1.44579	1.68731	-2.40811
H	-2.08858	4.31142	-3.84846
H	-1.43527	2.83985	-4.56519
H	-0.46375	3.77741	-3.41574
C	-1.95696	2.72258	3.61089
H	-1.51366	2.25259	4.49482
H	-2.78736	2.09727	3.26954
H	-2.37270	3.68496	3.92532
C	0.27404	3.76013	3.01264
H	-0.49292	1.90567	2.28788
H	-0.06037	4.76856	3.27655
H	1.05703	3.86078	2.25422
H	0.72113	3.31665	3.90832
C	1.24996	-2.46926	-0.90960
H	1.27421	-2.48224	-2.00653
H	0.87802	-3.43413	-0.56553
H	2.27775	-2.31087	-0.56854
H	-4.15800	-2.98736	-2.96651
H	-1.43491	-2.83969	-4.56555
H	-3.50005	-1.55737	-3.77130
C	-1.47566	-3.42825	-3.64339
C	-3.49006	-2.12970	-2.83804
H	-0.46365	-3.77771	-3.41626
H	-3.91269	-1.50287	-2.04618
C	-2.06266	-2.58850	-2.49965
H	-1.44503	-1.68740	-2.40840
H	-0.06008	-4.76862	3.27591
H	-2.08866	-4.31115	-3.84902
C	0.27403	-3.76002	3.01228
H	1.05706	-3.86025	2.25383
C	-1.44356	-2.80254	-0.00265
C	-0.89747	-2.90042	2.51349
C	-2.03804	-3.32658	-1.16849
C	-1.50220	-3.46960	1.23772
C	-2.14195	-4.71183	1.27517
C	-2.67133	-4.56934	-1.06567
C	-2.71962	-5.26206	0.13754
H	-2.19019	-5.25453	2.21475
H	-3.13860	-4.99897	-1.94694
H	-0.49347	-1.90564	2.28789
H	-3.21376	-6.22750	0.19020
H	0.72098	-3.31664	3.90807
H	-2.37265	-3.68575	3.92513
C	-1.95724	-2.72321	3.61080
H	-1.51403	-2.25321	4.49477
H	-2.78784	-2.09810	3.26957
H	5.46574	1.99092	-2.53123
C	6.26010	1.24307	-2.42500
H	7.05760	1.67692	-1.80356
H	6.66616	1.05994	-3.42060
H	4.21210	-0.00091	4.11697
C	3.40435	-0.00113	3.37502
C	4.85460	-1.20345	0.04213
C	5.16466	0.00022	-0.63180
C	4.26658	-1.19146	1.30405
C	4.85401	1.20343	0.04269
C	3.95407	-0.00068	1.97246
C	4.26599	1.19056	1.30459
N	5.73905	0.00064	-1.88801
H	5.10780	-2.15949	-0.40204
H	4.07629	-2.14350	1.79624
H	5.10677	2.15980	-0.40103
H	4.07523	2.14229	1.79722
H	2.79163	0.88437	3.57086
H	2.79237	-0.88721	3.57057
C	6.26067	-1.24133	-2.42554
H	6.66663	-1.05757	-3.42107
H	7.05839	-1.67506	-1.80430
H	5.46667	-1.98950	-2.53207
H	-3.45053	-0.00010	1.20691
H	1.73344	0.00014	-1.36979

INT-2[DHC]
B3PW91
SCF = -1467.10099131
SCF (C6H5Cl) = -1467.16102385
SCF (D3BJ) = -1467.28561512
SCF (BS2) = -3019.69831699
H (0 K) = -1466.445089
H (298 K) = -1466.406452
G (298 K) = -1466.517828
Low freq. = 10.4393
Second freq. = 14.4264

75
INT-2[DHC]
Zn -0.00001 -0.36975 -0.78734
N 1.49270 -0.05556 0.56720
N -1.49271 -0.05553 0.56721
C -1.34248 0.10025 1.83481
C 0.00001 0.01136 2.52052
C 1.34249 0.10022 1.83479
C -2.47879 0.35315 2.77988
H -3.43884 0.07664 2.34464
H -2.51070 1.42184 3.02626
H -2.32661 -0.18579 3.71987
C -2.80581 0.00731 -0.04164
C -3.44307 -1.20918 -0.35924
C -3.35745 1.26074 -0.37492
C -4.60249 1.26418 -1.01212
C -5.26941 0.08202 -1.30859
C -4.69034 -1.13936 -0.98609
C -2.64546 2.58275 -0.12381
H -5.05500 2.21346 -1.28304
H -6.23807 0.11243 -1.79818
H -5.21401 -2.05947 -1.22797
C -2.83908 -2.56865 -0.03532
C -2.18338 3.20781 -1.44998
H -1.53873 2.52776 -2.01634
H -1.62915 4.13404 -1.26706
H -3.03905 3.45204 -2.08736
C -3.51004 3.57112 0.67203
H -1.74627 2.38608 0.47210
H -4.39097 3.88338 0.10302
H -2.93783 4.47480 0.90467
H -3.86439 3.13959 1.61340
C -2.70005 -3.44348 -1.28947
H -2.19916 -4.38463 -1.04098
H -2.11902 -2.94014 -2.06796
H -3.67681 -3.69610 -1.71334
C -3.64162 -3.29312 1.05652
H -1.82764 -2.40907 0.36069
H -4.66219 -3.50189 0.72012
H -3.71324 -2.69925 1.97349
H -3.17307 -4.25027 1.30702
C 2.47881 0.35313 2.77985
H 3.43886 0.07662 2.34460
H 2.32665 -0.18579 3.71985
H 2.51073 1.42183 3.02622
H 3.03925 3.45221 -2.08704
H 2.93792 4.47461 0.90512
H 1.62933 4.13416 -1.26672
C 3.51011 3.57094 0.67239
C 2.18354 3.20793 -1.44973
H 3.86440 3.13929 1.61373
H 1.53889 2.52797 -2.01619
C 2.64554 2.58270 -0.12360
H 1.74631 2.38599 0.47224
H 4.66208 -3.50208 0.71985
H 4.39108 3.88325 0.10347
C 3.64152 -3.29330 1.05626
H 3.71315 -2.69950 1.97328
C 2.80581 0.00726 -0.04165
C 2.83900 -2.56871 -0.03552
C 3.35748 1.26069 -0.37484
C 3.44303 -1.20923 -0.35934
C 4.69030 -1.13941 -0.98618
C 4.60253 1.26414 -1.01203
C 5.26942 0.08198 -1.30859
H 5.21394 -2.05951 -1.22812
H 5.05508 2.21343 -1.28287
H 1.82756 -2.40913 0.36051
H 6.23808 0.11239 -1.79817
H 3.17293 -4.25045 1.30668
H 3.67669 -3.69605 -1.71363
C 2.69993 -3.44343 -1.28974
H 2.19901 -4.38459 -1.04132
H 2.11892 -2.94001 -2.06819
H -0.00002 -0.69928 -2.26969
H -0.00000 -0.94663 3.06722
H 0.00002 0.75524 3.32784

TS2[DHC]
B3PW91
SCF = -1872.49255360
SCF (C6H5Cl) = -1872.55022443
SCF (D3BJ) = -1872.74030828
SCF (BS2) = -3425.2172138
H (0 K) = -1871.633425
H (298 K) = -1871.583697
G (298 K) = -1871.720502
Low freq. = -26.6964
Second freq. = 4.6574

98
TS2[DHC]
Zn -0.66129 -0.93486 -0.77607
N -2.15926 -0.58992 0.60114
N 0.43813 -2.05131 0.56319
C -0.12763 -2.74970 1.48298
C -1.62453 -2.70135 1.71215
C -2.43545 -1.43472 1.52948
H -1.81718 -3.07835 2.72088
C 0.58885 -3.72519 2.36455
H 1.61119 -3.90531 2.03417
H 0.03766 -4.67177 2.39170
H 0.61107 -3.34490 3.39263
C 1.86580 -2.12157 0.33776
C 2.74642 -1.42337 1.19111
C 2.31902 -2.84434 -0.78707
C 3.69336 -2.85336 -1.03953
C 4.58361 -2.16759 -0.22156
C 4.10965 -1.46474 0.87838
C 1.38898 -3.66124 -1.67396
H 4.07209 -3.41468 -1.88827
H 5.64760 -2.18748 -0.43783
H 4.81179 -0.93545 1.51575
C 2.29984 -0.63542 2.41550
C 1.58734 -3.36924 -3.16615
H 1.46552 -2.30472 -3.38373
H 0.85090 -3.92250 -3.75736
H 2.57861 -3.67655 -3.51356
C 1.54245 -5.16336 -1.38344
H 0.35416 -3.38754 -1.43311
H 2.55470 -5.50584 -1.62168
H 0.84157 -5.74597 -1.99004
H 1.35678 -5.39773 -0.33015
C 2.60566 0.86106 2.26322
H 2.23469 1.41375 3.13243
H 2.15325 1.28388 1.36293
H 3.68350 1.03878 2.19785
C 2.93742 -1.18023 3.70394
H 1.21285 -0.73493 2.51607
H 4.02143 -1.02945 3.70293
H 2.75870 -2.25134 3.83747
H 2.53740 -0.65542 4.57756
C -3.60116 -1.31682 2.46189
H -4.14747 -2.26595 2.49615
H -4.27949 -0.51406 2.17539
H -3.23649 -1.12823 3.47871
H -4.94149 0.17775 -3.32105
H -5.69838 -2.31469 -1.62292
H -4.27998 -1.44233 -3.53089
C -5.53327 -1.38259 -1.07288
C -4.14637 -0.49865 -2.99264
H -5.57191 -1.60813 -0.00228
H -3.18784 -0.06944 -3.29683
C -4.19437 -0.74530 -1.47958
H -3.40375 -1.47106 -1.25272
H -2.94767 3.08490 3.63290
H -6.36921 -0.71175 -1.29605
C -2.45827 2.10587 3.62439
H -3.22353 1.35768 3.85160
C -2.98419 0.57765 0.37498
C -1.76785 1.84286 2.27620
C -3.92945 0.51884 -0.67202
C -2.77922 1.74263 1.14228
C -3.56327 2.86043 0.83449
C -4.68496 1.66694 -0.92536
C -4.50601 2.82941 -0.18489
H -3.43200 3.77113 1.41157
H -5.43102 1.64620 -1.71404
H -1.23874 0.88598 2.35485
H -5.10473 3.70948 -0.39976
H -1.72407 2.10043 4.43647
H -1.17184 3.91578 1.92800
C -0.71372 2.92407 1.99857
H 0.01744 2.96016 2.81194
H -0.17128 2.74148 1.06689
H -1.00394 2.84164 -1.39752
C -0.60838 2.49347 -2.35475
H -0.58050 3.34720 -3.05277
H -1.30486 1.75301 -2.75615
H 5.53589 5.38239 -0.42351
C 4.82314 5.02770 0.33071
C 2.96737 2.12567 -1.31541
C 1.68828 2.66053 -1.55523
C 3.95936 2.88534 -0.71069
C 1.45225 3.97864 -1.14053
C 3.73017 4.20500 -0.29729
C 2.45479 4.72378 -0.51702
N 0.68979 1.85972 -2.17674

H 3.18773 1.09944 -1.59274
H 4.93821 2.43823 -0.55082
H 0.49147 4.44970 -1.31371
H 2.23368 5.74454 -0.21335
H 4.42022 5.90825 0.83850
H 5.39382 4.44855 1.06417
C 1.11884 1.22707 -3.42586
H 0.35430 0.51407 -3.74537
H 1.27055 1.96576 -4.22889
H 2.05002 0.67785 -3.28764
H -0.71740 -0.91483 -2.29548
H -2.07283 -3.46046 1.04939

INT-3[DHC]
B3PW91
SCF = -1872.50327372
SCF (C6H5Cl) = -1872.55043390
SCF (D3BJ) = -1872.76852713
SCF (BS2) = -3425.22599203
H (0 K) = -1871.641710
H (298 K) = -1871.592571
G (298 K) = -1871.723949
Low freq. = 10.3033
Second freq. = 23.9621

98
INT-3[DHC]
Zn -0.39473 -0.87342 -0.99177
N -1.91115 -0.73926 0.50933
N 1.01963 -1.58765 0.45397
C 0.54036 -2.48239 1.24575
C -0.92495 -2.85503 1.15510
C -1.96700 -1.76164 1.28770
H -1.13698 -3.63549 1.88811
C 1.33363 -3.27641 2.23750
H 2.40422 -3.09009 2.16520
H 1.13844 -4.34468 2.09121
H 0.99465 -3.03482 3.25196
C 2.43184 -1.28761 0.47003
C 2.97729 -0.45918 1.47290
C 3.22668 -1.82102 -0.57222
C 4.58633 -1.49808 -0.58510
C 5.14612 -0.67811 0.38893
C 4.34573 -0.17084 1.40283
C 2.66115 -2.77551 -1.61599
H 5.21981 -1.90204 -1.36807
H 6.20613 -0.44347 0.36035
H 4.78976 0.46236 2.16561
C 2.16907 0.13211 2.62007
C 3.29046 -2.60523 -3.00414
H 3.29712 -1.56037 -3.32991
H 2.72694 -3.18622 -3.74041
H 4.32299 -2.96792 -3.03506
C 2.80563 -4.23567 -1.15434
H 1.58841 -2.57154 -1.71940
H 3.86118 -4.49881 -1.02781
H 2.37399 -4.91674 -1.89487
H 2.30442 -4.41742 -0.19919
C 2.16723 1.66661 2.57205
H 1.55460 2.07005 3.38494
H 1.77949 2.04190 1.62179
H 3.17834 2.06701 2.69895
C 2.68351 -0.34984 3.98644
H 1.12941 -0.19820 2.51783
H 3.69430 0.02198 4.18221
H 2.71861 -1.44045 4.05785
H 2.03853 0.02278 4.78893
C -3.05465 -2.03646 2.27981
H -3.57188 -2.96456 2.00871
H -3.78265 -1.22805 2.33701
H -2.61404 -2.20474 3.26903
H -5.31674 -0.78297 -2.78918
H -5.68964 -3.23983 -0.79305
H -4.64761 -2.41334 -2.91838
C -5.60804 -2.22640 -0.38712
C -4.47997 -1.42111 -2.48759
H -5.57846 -2.29716 0.70403
H -3.56718 -1.01914 -2.93474
C -4.36706 -1.52073 -0.95932
H -3.49593 -2.15031 -0.75078
H -2.75135 1.87469 3.79614
H -6.52628 -1.69339 -0.65358
C -1.88462 1.36348 3.36405
H -2.06193 0.28764 3.44556
C -3.00866 0.19365 0.44240
C -1.66582 1.81029 1.90857
C -4.15552 -0.15888 -0.30557
C -2.87325 1.46073 1.05085
C -3.91756 2.37586 0.89062
C -5.16171 0.80562 -0.43649
C -5.05041 2.05898 0.14883
H -3.84402 3.35364 1.35461
H -6.05313 0.56182 -1.00738
H -0.80673 1.25171 1.51339
H -5.84764 2.78741 0.03424
H -1.01177 1.60799 3.97822
H -2.05438 3.91196 2.38428
C -1.30476 3.29887 1.87363
H -0.35491 3.46354 2.38944
H -1.19691 3.66882 0.85082

H -1.92334 1.77451 -1.58621
C -1.45550 1.36843 -2.48384
H -1.47848 2.11991 -3.28304
H -2.02684 0.49981 -2.81509
H 4.16074 5.43297 -1.31573
C 3.49868 5.16610 -0.48362
C 2.10737 1.72621 -1.36828
C 0.78113 2.01442 -1.70742
C 2.97099 2.73917 -0.97007
C 0.34189 3.33663 -1.62170
C 2.54727 4.07209 -0.88512
C 1.21717 4.34268 -1.20790
N -0.07117 0.93298 -2.19947
H 2.47836 0.70675 -1.41390
H 3.99557 2.48196 -0.71566
H -0.67350 3.61207 -1.87994
H 0.84907 5.36388 -1.15095
H 2.96590 6.07231 -0.18454
H 4.13696 4.85703 0.34978
C 0.49443 0.40165 -3.47255
H -0.12629 -0.42672 -3.81926
H 0.51908 1.19483 -4.22945
H 1.50729 0.03491 -3.31255
H -0.80763 -1.99706 -2.01051
H -1.07404 -3.30104 0.15664

TS3[DHC]
B3PW91
SCF = -1872.47710950
SCF (C6H5Cl) = -1872.52430360
SCF (D3BJ) = -1872.74348646
SCF (BS2) = -3425.20230503
H (0 K) = -1871.619617
H (298 K) = -1871.571262
G (298 K) = -1871.701684
Low freq. = -980.5671
Second freq. = 8.8327

98
TS3[DHC]
Zn -0.31590 -0.59300 -0.78336
N -1.90208 -0.75740 0.45723
N 1.06391 -1.50048 0.38129
C 0.56402 -2.57733 0.93985
C -0.85192 -2.88498 0.82101
C -1.92912 -1.92802 1.04261
H -1.09181 -3.86875 1.21922
C 1.39663 -3.60071 1.66027
H 2.46682 -3.42574 1.55802
H 1.15379 -4.60141 1.28876
H 1.13886 -3.59096 2.72499
C 2.47910 -1.23680 0.46417
C 2.98378 -0.52797 1.57506
C 3.32902 -1.67148 -0.58100
C 4.69592 -1.39415 -0.47173
C 5.21373 -0.70853 0.62083
C 4.35977 -0.28037 1.62855
C 2.82686 -2.45172 -1.78987
H 5.36875 -1.73014 -1.25487
H 6.28000 -0.51268 0.68666
H 4.76764 0.25361 2.48224
C 2.10519 -0.03588 2.71578
C 3.42927 -1.95018 -3.11090
H 3.36177 -0.86204 -3.21390
H 2.91270 -2.40975 -3.95961
H 4.48705 -2.21626 -3.20161
C 3.09815 -3.95786 -1.63990
H 1.73945 -2.32173 -1.84881
H 4.17342 -4.15079 -1.56266
H 2.72074 -4.50435 -2.51041
H 2.62230 -4.37551 -0.74925
C 2.19151 1.48903 2.87293
H 1.51710 1.82782 3.66623
H 1.92350 2.00520 1.94657
H 3.20209 1.80617 3.15072
C 2.45650 -0.73187 4.04003
H 1.06593 -0.28273 2.47473
H 3.47297 -0.48254 4.36189
H 2.39718 -1.82106 3.95872
H 1.77279 -0.41295 4.83355
C -3.07240 -2.40676 1.89155
H -3.51012 -3.31343 1.46052
H -3.85226 -1.65366 2.00162
H -2.69500 -2.67847 2.88283
H -5.25452 -0.61654 -2.88727
H -5.66443 -3.23383 -1.08856
H -4.61076 -2.24595 -3.11595
C -5.61001 -2.24801 -0.61549
C -4.43524 -1.28909 -2.61397
H -5.62081 -2.38915 0.46843
H -3.50993 -0.87322 -3.02317
C -4.35619 -1.49544 -1.09368
H -3.48860 -2.13604 -0.90528
H -2.90266 1.55540 3.94546
H -6.52329 -1.70724 -0.88335
C -2.02937 1.07147 3.49539
H -2.22069 -0.00542 3.47595
C -3.03837 0.12387 0.45866
C -1.76670 1.63254 2.08766
C -4.17236 -0.18634 -0.33038

C -2.94798 1.33872 1.17562
C -4.00780 2.24434 1.07679
C -5.19781 0.76487 -0.39560
C -5.12196 1.96845 0.29164
H -3.96306 3.17970 1.62490
H -6.07755 0.55117 -0.99618
H -0.89534 1.10209 1.68212
H -5.93364 2.68694 0.22492
H -1.17082 1.25100 4.15091
H -2.17390 3.70128 2.68562
C -1.40554 3.11937 2.16650
H -0.47642 3.24752 2.72902
H -1.25906 3.55719 1.17459
H -1.98049 1.89587 -1.42038
C -1.57804 1.47084 -2.34019
H -1.61333 2.21623 -3.14270
H -2.19663 0.62228 -2.63056
H 4.21820 5.34565 -1.28237
C 3.53689 5.11348 -0.45550
C 2.02396 1.72331 -1.32336
C 0.71377 2.06219 -1.66966
C 2.92385 2.70493 -0.92656
C 0.32016 3.39863 -1.60345
C 2.54928 4.05354 -0.85924
C 1.23306 4.37321 -1.19709
N -0.18923 0.99450 -2.12880
H 2.35557 0.68922 -1.35164
H 3.93440 2.40981 -0.65837
H -0.68486 3.70956 -1.86113
H 0.90441 5.40806 -1.15077
H 3.03621 6.04220 -0.17107
H 4.15222 4.78635 0.38793
C 0.30138 0.44499 -3.42817
H -0.35905 -0.36513 -3.74666
H 0.30583 1.23620 -4.18624
H 1.31170 0.05542 -3.31597
H -0.76720 -2.19797 -1.45603
H -0.86423 -2.72851 -0.61467

INT-4[DHC]
B3PW91
SCF = -1805.75183276
SCF (C6H5Cl) = -1805.80670021
SCF (D3BJ) = -1806.00995453
SCF (BS2) = -3358.44743430
H (0 K) = -1804.871638
H (298 K) = -1804.824720
G (298 K) = -1804.952359
Low freq. = 8.7108
Second freq. = 23.9777

99
INT-4[DHC]
Zn 0.00007 -0.03727 -0.21716
N -1.51239 -1.35540 -0.46154
N 1.51263 -1.35522 -0.46149
C -2.45919 -3.59586 -0.81734
C -1.34396 -2.59617 -0.75751
C 0.00023 -3.18517 -1.11613
C 1.34436 -2.59600 -0.75753
C 2.45972 -3.59553 -0.81748
C -2.83329 -0.83299 -0.17134
C -3.48440 -0.10904 -1.19308
C -4.73728 0.43296 -0.89682
C -5.30893 0.28381 0.36236
C -4.63364 -0.41588 1.35330
C -3.38136 -0.99381 1.11631
C -2.88184 0.04547 -2.58350
C -3.17130 1.40943 -3.21971
C -3.34427 -1.08416 -3.51890
C -2.67962 -1.73258 2.24808
C -3.53457 -2.88441 2.79872
C -2.28246 -0.76946 3.37741
C 2.83348 -0.83266 -0.17133
C 3.38164 -0.99348 1.11627
C 4.63386 -0.41543 1.35324
C 5.30901 0.28442 0.36231
C 4.73728 0.43358 -0.89683
C 3.48445 -0.10856 -1.19307
C 2.68002 -1.73238 2.24804
C 2.28269 -0.76929 3.37735
C 3.53513 -2.88407 2.79869
C 2.88180 0.04595 -2.58344
H 1.78989 -0.03437 -2.48477
C 3.34411 -1.08371 -3.51886
H 2.89261 -0.97165 -4.50997
H 3.07961 -2.07548 -3.13891
C 1.31447 2.91919 0.57722
C 1.29332 4.07495 -0.44949
C -0.00006 4.90646 -0.48427
C -1.29344 4.07493 -0.45017
C -1.31513 2.91919 0.57656
C -1.29821 3.37899 2.05345
C -0.00083 4.06180 2.52219
C 1.29678 3.37896 2.05412
B -0.00027 2.03356 0.34033
H -1.78993 -0.03490 -2.48491
H 4.43185 -1.05966 -3.64146
H 1.75372 -2.17032 1.85711
H -0.00000 1.54470 -0.89971
H -0.00049 1.00307 1.16913

H -1.75325 -2.17037 1.85714
H 2.25058 2.36500 0.42358
H -5.27184 0.98661 -1.66151
H -4.43202 -1.06006 -3.64142
H -1.47362 2.49211 2.67976
H -2.14587 4.74778 -0.28124
H -2.14462 4.05222 2.24900
H 1.47182 2.49206 2.68051
H -2.25115 2.36499 0.42243
H 5.08501 -0.51699 2.33585
H -2.58817 1.52203 -4.13883
H -0.00073 5.10600 2.19819
H -5.08471 -0.51741 2.33594
H 3.85102 -3.57684 2.01256
H -3.43782 -3.11894 -0.85727
H -0.00111 4.10512 3.61874
H 6.28147 0.71983 0.57139
H -6.28143 0.71912 0.57147
H -1.44824 3.63883 -1.44818
H -0.00029 5.61973 0.34417
H -3.07979 -2.07594 -3.13897
H -4.22487 1.51460 -3.49710
H 5.27172 0.98736 -1.66149
H 2.14565 4.74781 -0.28008
H -2.89284 -0.97211 -4.51004
H -2.97249 -3.45078 3.54810
H 2.14311 4.05216 2.25014
H 0.00017 5.52337 -1.39180
H -2.41936 -4.22643 0.07963
H -2.33333 -4.26075 -1.67752
H 2.97315 -3.45051 3.54809
H 1.44867 3.63887 -1.44742
H 3.16566 -0.30760 3.83030
H 2.33395 -4.26033 -1.67774
H -2.91261 2.23228 -2.54837
H -1.74717 -1.30651 4.16716
H 1.63898 0.03642 3.01301
H 1.74747 -1.30641 4.16711
H 4.44052 -2.51018 3.28645
H -3.85038 -3.57720 2.01258
H -4.44000 -2.51064 3.28649
H 3.43829 -3.11849 -0.85734
H 2.41997 -4.22623 0.07941
H -1.63885 0.03636 3.01309
H -3.16549 -0.30791 3.83036
C 3.17128 1.40987 -3.21971
H 4.22483 1.51498 -3.49720
H 2.58806 1.52248 -4.13878
H 2.91269 2.23276 -2.54838
H 0.00030 -4.22389 -0.76450
H 0.00023 -3.29088 -2.21434

TS4[DHC]
B3PW91
SCF = -1805.68342483
SCF (C6H5Cl) = -1805.73312294
SCF (D3BJ) = -1805.93567766
SCF (BS2) = -3358.38044851
H (0 K) = -1804.811753
H (298 K) = -1804.763949
G (298 K) = -1804.897049
Low freq. = -940.1071
Second freq. = 13.0640

99
TS4[DHC]
Zn 0.07433 -0.31422 -0.88848
N -1.35240 -1.52323 -0.23916
N 1.67574 -1.28910 -0.24745
C 1.55406 -2.56219 -0.53940
C 0.28897 -3.08571 -1.03935
C -1.03661 -2.76368 -0.52601
H 0.36788 -4.12116 -1.36289
C 2.70692 -3.52398 -0.50619
H 3.63430 -3.04651 -0.19113
H 2.85106 -3.95799 -1.50189
H 2.47958 -4.35283 0.17181
C 2.94033 -0.69766 0.11055
C 3.25029 -0.50912 1.47319
C 3.81043 -0.26668 -0.91611
C 4.99846 0.36795 -0.53978
C 5.32640 0.55995 0.79665
C 4.45990 0.11821 1.78864
C 3.50630 -0.45204 -2.39657
H 5.68263 0.71060 -1.31070
H 6.25889 1.04792 1.06452
H 4.72794 0.26002 2.83141
C 2.34367 -0.99871 2.59174
C 3.15223 0.88790 -3.05895
H 2.29372 1.36404 -2.57134
H 2.90252 0.74415 -4.11524
H 3.99108 1.58957 -3.00359
C 4.65480 -1.14164 -3.14676
H 2.62811 -1.09924 -2.49034
H 5.55424 -0.51887 -3.17721
H 4.36299 -1.34151 -4.18273
H 4.92705 -2.09345 -2.68085
C 2.07152 0.08862 3.63922
H 1.36200 -0.27573 4.38839
H 1.65368 0.99230 3.18476

H 2.98193 0.37687 4.17381
C 2.92352 -2.25601 3.25942
H 1.38177 -1.27420 2.14437
H 3.88585 -2.03794 3.73433
H 3.08873 -3.06087 2.53698
H 2.24611 -2.62976 4.03436
C -2.02433 -3.89335 -0.47036
H -2.11521 -4.35226 -1.46111
H -3.00974 -3.56404 -0.14173
H -1.65945 -4.67118 0.20806
H -4.14392 0.82380 -3.08162
H -4.04690 -2.18754 -4.10841
H -2.95692 0.10689 -4.18139
C -4.34985 -1.98482 -3.07620
C -3.20309 0.26528 -3.12639
H -4.45396 -2.94294 -2.55824
H -2.42234 0.89906 -2.69089
C -3.31866 -1.07733 -2.38953
H -2.34653 -1.57396 -2.47375
H -3.39165 -2.46733 3.78956
H -5.34002 -1.52005 -3.11347
C -2.41175 -2.55431 3.30852
H -2.46117 -3.39661 2.61194
C -2.69100 -1.12633 0.11624
C -2.03193 -1.24630 2.59587
C -3.62691 -0.87248 -0.91211
C -3.01239 -0.93512 1.47575
C -4.29924 -0.48470 1.78760
C -4.89254 -0.40746 -0.53992
C -5.23163 -0.21444 0.79373
H -4.57573 -0.34684 2.82872
H -5.62885 -0.20239 -1.31174
H -1.04411 -1.39074 2.14348
H -6.22398 0.13876 1.05815
H -1.67919 -2.79878 4.08481
H -2.85383 0.07298 4.14096
C -1.91436 -0.09802 3.60628
H -1.15478 -0.33068 4.35864
H -1.63346 0.84196 3.12057
H -0.04201 1.40687 -0.75120
H 0.13278 -1.15523 -2.44767
H 0.20640 -2.07782 -2.11754
H -3.00966 4.49531 0.76259
H -2.48010 3.09864 1.67359
C -2.13362 3.86813 0.97147
H -2.50693 2.46752 -0.64187
H -1.37707 4.95119 2.67151
C -1.70298 3.13925 -0.32489
H -2.28405 4.67637 -1.75580
C -1.04780 4.71853 1.65211
C -1.38252 4.08843 -1.54195
H -0.96800 5.68714 1.15238
B -0.31967 2.48841 -0.14982
H -1.21452 3.46704 -2.43318
C 0.33872 4.05825 1.72683
H 0.33297 3.30627 2.52735
H -0.45394 5.85424 -0.69678
C -0.17562 5.01979 -1.34336
C 0.81467 3.35208 0.43295
H 1.08306 4.80645 2.02770
H 0.07986 5.47142 -2.30913
C 1.07307 4.31635 -0.78599
H 1.75520 2.82803 0.63164
H 1.54678 3.73545 -1.58921
H 1.81947 5.05851 -0.47567

INT-2B+
B3PW91
SCF = -1804.53312237
SCF (C6H5Cl) = -1804.58759082
SCF (D3BJ) = -1804.78136691
SCF (BS2) = -3357.22959397
H (0 K) = -1803.675593
H (298 K) = -1803.627816
G (298 K) = -1803.762518
Low freq. = 12.2139
Second freq. = 15.7430

97
INT-2B+
Zn 0.18239 -0.37053 0.02233
N -1.14120 -1.70690 0.19222
N 1.88201 -1.19491 0.12131
C -1.67551 -4.06865 0.54135
C -0.67502 -2.95638 0.36891
C 0.68922 -3.30281 0.40188
C 1.86142 -2.52875 0.28391
C 3.17794 -3.25666 0.35090
C -2.54961 -1.41987 0.14052
C -3.22121 -1.48110 -1.09969
C -4.56254 -1.08611 -1.13712
C -5.22152 -0.65591 0.00898
C -4.54817 -0.62494 1.22499
C -3.20644 -1.00722 1.31989
C -2.52729 -1.93557 -2.37672
C -2.15980 -0.73425 -3.26059
C -3.35503 -2.95519 -3.17075
C -2.48815 -0.95124 2.66087
C -3.31125 -1.55225 3.80780
C -2.06998 0.48783 2.99803

C 3.11662 -0.46106 0.02610
C 3.72126 0.02533 1.20562
C 4.86717 0.81599 1.07618
C 5.40357 1.11258 -0.17141
C 4.80128 0.61219 -1.31951
C 3.65344 -0.18353 -1.24943
C 3.15252 -0.25959 2.58888
C 2.42325 0.97340 3.14410
C 4.21885 -0.74577 3.57998
C 3.01390 -0.69979 -2.53063
H 2.26225 -1.44743 -2.25460
C 4.02233 -1.39457 -3.45596
H 3.50643 -1.83455 -4.31534
H 4.55984 -2.19511 -2.93878
C 0.05381 3.60774 0.55391
C 0.57765 4.28541 -0.76989
C -0.49322 4.54047 -1.84284
C -1.41545 3.33868 -2.10482
C -1.98487 2.63431 -0.81361
C -2.93988 3.51256 0.03337
C -2.29814 4.73352 0.71338
C -0.93779 4.46559 1.37872
B -0.67057 2.39396 -0.05387
H -1.59120 -2.42912 -2.09456
H 0.87214 -4.36057 0.54203
H 2.41246 -1.06127 2.49183
H 0.00626 1.31719 -0.18074
H -1.57293 -1.54706 2.57545
H 0.93329 3.38208 1.16492
H -5.10169 -1.12096 -2.07928
H -4.26441 -2.51035 -3.58688
H -3.37977 2.86166 0.79939
H -2.26928 3.64281 -2.72327
H -3.77733 3.84364 -0.59364
H -1.10096 3.94648 2.33218
H -2.52095 1.73832 -1.14148
H 5.34910 1.20447 1.96864
H -1.63868 -1.06163 -4.16618
H -2.20256 5.55023 -0.00634
H -5.07656 -0.30422 2.11795
H 4.77103 -1.60694 3.19145
H -2.29613 -4.17284 -0.35414
H -2.98935 5.10794 1.47732
H 6.29515 1.72801 -0.24820
H -6.26400 -0.35574 -0.04338
H -0.87048 2.59031 -2.69727
H -1.08905 5.41474 -1.57404
H -3.65612 -3.80442 -2.54975
H -3.05519 -0.18360 -3.56806
H 5.23205 0.84068 -2.29016
H 1.06721 5.22695 -0.49100
H -2.77046 -3.34161 -4.01154
H -2.70920 -1.59644 4.72080
H -0.46317 5.42085 1.63626
H 0.00875 4.80628 -2.78052
H -2.35641 -3.85064 1.36970
H -1.17990 -5.02014 0.73370
H 3.74981 -1.04276 4.52335
H 1.36960 3.65124 -1.19171
H 3.11038 1.81899 3.25451
H 3.03122 -4.33307 0.43903
H -1.50520 -0.02955 -2.73355
H -1.51493 0.52776 3.94083
H 1.61147 1.29620 2.48132
H 1.99081 0.76096 4.12743
H 4.94789 0.03659 3.81315
H -3.64479 -2.56747 3.57337
H -4.19921 -0.95398 4.03489
H 3.77684 -3.05296 -0.54177
H 3.76653 -2.91182 1.20667
H -1.42675 0.91667 2.21634
H -2.94491 1.13961 3.09113
C 2.28923 0.43246 -3.27459
H 2.99125 1.21667 -3.57686
H 1.79715 0.05526 -4.17705
H 1.52530 0.90630 -2.64573
H 4.76728 -0.69476 -3.84750

TS[2B+]
B3PW91
SCF = -2209.91626570
SCF (C6H5Cl) = -2209.96040586
SCF (D3BJ) = -2210.25059493
SCF (BS2) = -3762.73765328
H (0 K) = -2208.853244
H (298 K) = -2208.795376
G (298 K) = -2208.947454
Low freq. = -60.9846
Second freq. = 14.8545

120
TS[2B+]
Zn 0.08363 -0.10715 -0.40281
N 0.11633 1.68451 -1.14023
N 1.66421 -1.00073 -1.08218
C 0.47334 2.85437 -3.28914
C 0.64837 1.66853 -2.37122
C 1.36123 0.57757 -2.91981
C 1.94144 -0.56240 -2.32302
C 2.89907 -1.33420 -3.20158

C -0.25226 2.90944 -0.47905
C 0.74211 3.86915 -0.15323
C 0.35622 5.01549 0.55019
C -0.95715 5.22936 0.94220
C -1.91982 4.28417 0.61864
C -1.59723 3.12387 -0.09162
C 2.21957 3.73511 -0.49939
C 3.07568 3.66152 0.77294
C 2.71003 4.89317 -1.38465
C -2.73170 2.19514 -0.48668
C -3.56033 2.82981 -1.61586
C -3.63874 1.81519 0.68949
C 2.53108 -1.93703 -0.40783
C 2.11262 -3.27304 -0.20379
C 2.96769 -4.15836 0.45900
C 4.21588 -3.75562 0.91178
C 4.61249 -2.44022 0.71906
C 3.79109 -1.50466 0.07976
C 0.79515 -3.80371 -0.73411
C -0.02135 -4.54342 0.33371
C 1.02597 -4.70850 -1.95473
C 4.31408 -0.07931 -0.04597
H 3.50585 0.55180 -0.43162
C 5.49586 0.01020 -1.02550
H 5.82512 1.04881 -1.13416
H 5.24146 -0.36897 -2.01788
C -1.60587 -0.54516 3.22106
C -0.97822 -1.83928 3.84788
C 0.49142 -1.70383 4.27200
C 1.38633 -1.03360 3.21813
C 0.80293 0.27497 2.56407
C 0.63596 1.47243 3.53566
C -0.39439 1.27272 4.65749
C -1.72339 0.64928 4.20232
B -0.58920 -0.21863 2.09920
H 2.37216 2.80264 -1.05194
H 1.64344 0.71907 -3.95846
H 0.21066 -2.93991 -1.06431
H -0.93937 -0.58812 0.94274
H -2.29493 1.26731 -0.87339
H -2.60513 -0.80924 2.86017
H 1.11019 5.75701 0.79816
H 2.69770 5.84195 -0.83857
H 0.35058 2.34575 2.93536
H 2.35902 -0.78048 3.65837
H 1.61153 1.71463 3.97609
H -2.32604 1.42259 3.70932
H 1.50960 0.56990 1.77721
H 2.65503 -5.18859 0.60270
H 4.13321 3.54920 0.51406
H 0.04346 0.67435 5.46008
H -2.95293 4.45730 0.90500
H 1.55988 -4.18319 -2.75207
H 1.44023 3.26956 -3.58487
H -0.60547 2.24832 5.11053
H 4.87480 -4.46081 1.40971
H -1.22908 6.12851 1.48714
H 1.60409 -1.75358 2.41806
H 0.55614 -1.16282 5.21802
H 2.09327 5.02438 -2.27733
H 2.98090 4.57464 1.36922
H 5.58852 -2.12560 1.07638
H -1.59478 -2.13568 4.70603
H 3.74185 4.71603 -1.70539
H -4.36445 2.15789 -1.93220
H -2.30160 0.33587 5.08097
H 0.89139 -2.70337 4.47931
H -0.12645 3.64374 -2.83679
H -0.02201 2.51804 -4.20633
H 0.07660 -5.07296 -2.36151
H -1.06995 -2.65742 3.11989
H 0.47096 -5.46504 0.65898
H 2.32509 -1.82006 -3.99941
H 2.78664 2.81974 1.40814
H -4.36903 1.06453 0.37499
H -0.17861 -3.92556 1.22405
H -1.00193 -4.83079 -0.06150
H 1.62426 -5.58348 -1.68014
H -2.94374 3.06932 -2.48832
H -4.02066 3.76377 -1.27707
H 3.61485 -0.66651 -3.68635
H 3.44129 -2.10441 -2.65477
H -3.06536 1.39893 1.52322
H -4.19479 2.67719 1.07148
C 4.73753 0.48206 1.32030
H 5.57197 -0.08266 1.74692
H 5.06914 1.51887 1.22061
H 3.91776 0.45570 2.04429
H 6.34992 -0.57055 -0.66127
H -2.80578 -0.52937 -4.05846
H -0.62215 -2.41583 -3.21225
H -1.10752 -0.13907 -3.72171
C -1.62817 -2.50050 -2.79406
C -2.09776 -0.22787 -3.27136
H -1.63837 -3.29398 -2.04909
H -2.39441 0.74776 -2.88748
N -2.02276 -1.21397 -2.18841
H -2.32359 -2.78219 -3.59924
C -4.47556 -0.84862 -1.86163
C -3.25070 -1.37520 -1.44579

C -5.63282 -1.06703 -1.10955
C -5.61588 -1.82153 0.06416
C -3.22382 -2.12877 -0.26344
C -4.38166 -2.34931 0.47120
H -2.28405 -2.55917 0.07358
H -4.32893 -2.94703 1.37836
C -6.86966 -2.08229 0.85437
H -7.18942 -3.12573 0.75141
H -7.69489 -1.45026 0.51707
H -6.71735 -1.89611 1.92247
H -6.57205 -0.64607 -1.45987
H -4.55133 -0.27204 -2.77649

INT-1B+
B3PW91
SCF = -2209.91913587
SCF (C6H5Cl) = -2209.96254873
SCF (D3BJ) = -2210.25788086
SCF (BS2) = -3762.73985847
H (0 K) = -2208.855320
H (298 K) = -2208.797182
G (298 K) = -2208.949946
Low freq. = 10.4759
Second freq. = 15.4298

120
INT-1B+
Zn -0.17861 -0.21734 -0.73392
N 0.19416 1.68366 -1.16962
N 1.57398 -1.08705 -1.10025
C 0.81347 2.91607 -3.22977
C 0.83184 1.68948 -2.34787
C 1.49395 0.56301 -2.89661
C 1.98455 -0.61830 -2.29025
C 2.98615 -1.39163 -3.11962
C -0.09832 2.89478 -0.45396
C 0.94438 3.79074 -0.08762
C 0.62057 4.92973 0.65848
C -0.67569 5.20197 1.06640
C -1.68621 4.32067 0.71244
C -1.42853 3.17536 -0.04578
C 2.42030 3.60033 -0.41638
C 3.24572 3.45048 0.86913
C 2.98487 4.76493 -1.24843
C -2.62395 2.34849 -0.48343
C -3.34296 3.05091 -1.64752
C -3.61552 2.04725 0.64599
C 2.37692 -2.03976 -0.37362
C 1.89360 -3.35074 -0.13529
C 2.69580 -4.26860 0.54725
C 3.96451 -3.92897 0.99254
C 4.42375 -2.63850 0.78141
C 3.65375 -1.66884 0.12786
C 0.55828 -3.83114 -0.66362
C -0.26348 -4.61239 0.36933
C 0.75343 -4.66726 -1.93805
C 4.25321 -0.27043 0.03659
H 3.50223 0.40415 -0.38858
C 5.50241 -0.22910 -0.85909
H 5.87191 0.79754 -0.95078
H 5.31045 -0.61014 -1.86413
C -1.75664 -0.43512 3.17743
C -1.23448 -1.78100 3.79152
C 0.21565 -1.74467 4.29784
C 1.20557 -1.09623 3.31617
C 0.72462 0.24956 2.66364
C 0.58082 1.43373 3.65341
C -0.51022 1.26772 4.72236
C -1.85055 0.73463 4.19009
B -0.66500 -0.14435 2.11561
H 2.54405 2.67937 -0.99462
H 1.85589 0.71853 -3.90867
H -0.01107 -2.93538 -0.92966
H -0.91259 -0.44392 0.92223
H -2.25462 1.38240 -0.84573
H -2.75348 -0.63078 2.76780
H 1.41507 5.61801 0.93152
H 3.01735 5.68883 -0.66197
H 0.36999 2.33429 3.06218
H 2.16225 -0.90933 3.82089
H 1.54560 1.61532 4.14488
H -2.38302 1.55679 3.69455
H 1.46135 0.51788 1.89877
H 2.32570 -5.27637 0.71014
H 4.30058 3.28855 0.62633
H -0.14567 0.62360 5.52640
H -2.70754 4.53710 1.01029
H 1.27729 -4.10334 -2.71565
H 1.82479 3.24190 -3.48401
H -0.68689 2.24146 5.19428
H 4.58578 -4.65950 1.50203
H -0.89682 6.09313 1.64617
H 1.43132 -1.80749 2.51055
H 0.25576 -1.23563 5.26294
H 2.39134 4.96988 -2.14246
H 3.18865 4.35363 1.48490
H 5.41147 -2.36650 1.14130
H -1.91091 -2.07341 4.60522
H 4.01055 4.54473 -1.56236
H -4.18520 2.45216 -2.01050

H -2.48633 0.43393 5.03313
H 0.54411 -2.77185 4.49672
H 0.27549 3.74636 -2.77298
H 0.31856 2.65651 -4.17274
H -0.20470 -5.00888 -2.34561
H -1.32906 -2.56875 3.03018
H 0.19082 -5.57852 0.60864
H 2.47630 -1.76370 -4.01633
H 2.89854 2.61176 1.47734
H -4.38214 1.35016 0.29579
H -0.36868 -4.05806 1.30745
H -1.26539 -4.82513 -0.02017
H 1.35251 -5.55722 -1.71951
H -2.66758 3.25031 -2.48613
H -3.74032 4.01675 -1.31892
H 3.79811 -0.74808 -3.46530
H 3.40626 -2.24260 -2.58531
H -3.11873 1.59689 1.50955
H -4.12958 2.94932 0.99174
C 4.62125 0.25088 1.43562
H 5.38526 -0.37478 1.90657
H 5.02900 1.26264 1.37273
H 3.75579 0.27200 2.10361
H 6.31081 -0.83103 -0.43076
H -2.62650 -0.21447 -3.99771
H -0.43825 -2.14179 -3.23217
H -0.91782 0.15575 -3.69258
C -1.45861 -2.21303 -2.85093
C -1.90178 0.07970 -3.22940
H -1.53557 -3.05310 -2.16696
H -2.17962 1.04860 -2.82164
N -1.82351 -0.94412 -2.16102
H -2.14846 -2.38774 -3.68582
C -4.30490 -0.62392 -1.91544
C -3.09569 -1.14964 -1.46285
C -5.49115 -0.90554 -1.23344
C -5.51267 -1.71826 -0.09913
C -3.10261 -1.96311 -0.32445
C -4.28892 -2.24148 0.34234
H -2.17396 -2.39740 0.03674
H -4.26493 -2.88192 1.22045
C -6.79558 -2.04037 0.61697
H -7.07597 -3.08890 0.46483
H -7.62170 -1.42169 0.25857
H -6.70041 -1.88528 1.69637
H -6.42066 -0.48332 -1.60632
H -4.35135 0.00608 -2.79548

TS[1B+]
B3PW91
SCF = -2209.91346439
SCF (C6H5Cl) = -2209.96536924
SCF (D3BJ) = -2210.23870811
SCF (BS2) = -3762.73413937
H (0 K) = -2208.850176
H (298 K) = -2208.792566
G (298 K) = -2208.943305
Low freq. = -25.4313
Second freq. = 10.7398

120
TS[1B+]
Zn 0.51856 0.29026 -0.92315
N 2.33346 0.79412 -0.34032
N 0.70694 -1.63777 -1.31660
C 4.34048 1.13989 -1.76445
C 3.09105 0.38159 -1.37539
C 2.76011 -0.70784 -2.21445
C 1.84106 -1.77564 -2.00463
C 2.22789 -3.10745 -2.59242
C 2.87349 1.51930 0.76667
C 4.06037 1.08361 1.42607
C 4.52821 1.82238 2.51789
C 3.85969 2.93647 3.00092
C 2.68048 3.32512 2.38446
C 2.17313 2.64314 1.27635
C 4.84841 -0.18065 1.09004
C 4.75975 -1.19292 2.24207
C 6.32860 0.10761 0.78449
C 0.88589 3.18187 0.67734
C 1.08098 4.59979 0.11924
C -0.27816 3.14590 1.67736
C -0.05630 -2.78131 -0.87639
C -1.14320 -3.26075 -1.64545
C -1.90086 -4.32440 -1.14516
C -1.60138 -4.91983 0.07251
C -0.51223 -4.46193 0.80070
C 0.28140 -3.40132 0.34974
C -1.50454 -2.70937 -3.01449
C -2.92039 -2.11409 -3.02982
C -1.36960 -3.77283 -4.11628
C 1.48834 -3.01024 1.18997
H 1.99020 -2.16601 0.70590
C 2.49119 -4.17277 1.27267
H 3.38855 -3.86831 1.81914
H 2.79700 -4.51756 0.28035
C -3.87663 -0.43573 1.43731
C -4.25057 -1.90629 1.79877

C -3.96681 -2.30779 3.25369
C -2.58060 -1.87713 3.75855
C -2.15128 -0.42341 3.39025
C -2.98088 0.68229 4.09708
C -4.46249 0.74080 3.69968
C -4.70898 0.64036 2.18601
B -2.37233 -0.29826 1.84683
H 4.40762 -0.66120 0.21179
H 3.48414 -0.88950 -3.00243
H -0.79133 -1.91402 -3.24433
H -1.46185 -0.16997 1.04092
H 0.60135 2.54254 -0.16994
H -4.06429 -0.31682 0.36179
H 5.43842 1.49852 3.01393
H 6.86012 0.43327 1.68433
H -2.51437 1.65182 3.87185
H -2.52647 -2.00113 4.84855
H -2.89765 0.56019 5.18533
H -4.47308 1.61530 1.73644
H -1.10691 -0.30415 3.70359
H -2.73381 -4.70150 -1.73201
H 5.27670 -2.12091 1.97539
H -5.01699 -0.04322 4.22068
H 2.14220 4.18910 2.76370
H -0.36501 -4.20421 -4.14762
H 5.21438 0.48700 -1.81262
H -4.88931 1.68388 4.06276
H -2.20031 -5.74730 0.44142
H 4.24713 3.48753 3.85244
H -1.83261 -2.56324 3.34093
H -4.74664 -1.91048 3.90754
H 6.46197 0.88731 0.03083
H 5.22973 -0.80344 3.15074
H -0.25953 -4.94705 1.73923
H -5.31137 -2.07311 1.56729
H 6.82624 -0.80084 0.42929
H 0.16686 4.95613 -0.36523
H -5.77875 0.47428 1.99827
H -4.04747 -3.39850 3.33866
H 4.54836 1.96530 -1.08437
H 4.19484 1.54791 -2.77142
H -1.57786 -3.33404 -5.09790
H -3.68919 -2.57446 1.13081
H -3.67516 -2.88620 -2.85021
H 2.68628 -2.96394 -3.57454
H 3.72192 -1.43084 2.48304
H -1.20855 3.46047 1.19368
H -3.05406 -1.35134 -2.25531
H -3.14560 -1.66356 -4.00296
H -2.07651 -4.59512 -3.96749
H 1.90320 4.64274 -0.60290
H 1.32493 5.30162 0.92281
H 2.98053 -3.58524 -1.95588
H 1.38103 -3.78737 -2.68123
H -0.42150 2.14129 2.08713
H -0.09723 3.82016 2.52009
C 1.07269 -2.55688 2.59584
H 0.55184 -3.35424 3.13545
H 1.94762 -2.28483 3.19370
H 0.40912 -1.68871 2.55556
H 2.06157 -5.03172 1.79829
H 0.65615 2.93731 -3.83726
H 0.13714 0.05923 -3.91417
H 1.71093 1.65637 -3.21484
C -0.68352 0.71977 -3.62561
C 0.91759 2.35484 -2.94723
H -1.55172 0.12889 -3.34428
H 1.27048 3.02289 -2.16281
N -0.24890 1.56674 -2.47766
H -0.95234 1.36167 -4.47258
C -1.47069 3.75968 -2.37549
C -1.38379 2.40337 -2.06469
C -2.61001 4.48377 -2.02119
C -3.69061 3.88227 -1.37304
C -2.44741 1.79028 -1.39938
C -3.58345 2.51886 -1.06711
H -2.39794 0.73441 -1.14132
H -4.40269 2.01676 -0.56111
C -4.93390 4.65764 -1.03429
H -5.72177 4.46959 -1.77309
H -4.74524 5.73392 -1.02213
H -5.33171 4.37040 -0.05670
H -2.65431 5.54133 -2.26754
H -0.66818 4.27674 -2.88750

INT-3B+
B3PW91
SCF = -2008.82424340
SCF (C6H5Cl) = -2008.87646146
SCF (D3BJ) = -2009.12930949
SCF (BS2) = -3949.53238816
H (0 K) = -2007.869596
H (298 K) = -2007.816137
G (298 K) = -2007.959244
Low freq. = 10.4823
Second freq. = 15.6621

109
INT-3B+
Zn 0.58396 -0.51533 -0.02648
N 2.42691 -1.02188 0.22977
N -0.35476 -2.21424 -0.03461
C 4.13763 -2.73043 0.63891
C 2.71464 -2.32581 0.35049
C 1.77474 -3.36385 0.22921
C 0.38399 -3.33570 0.03925
C -0.28919 -4.67891 -0.11495
C 3.46642 -0.03464 0.35889
C 3.66251 0.58461 1.61387
C 4.57824 1.63748 1.69525
C 5.29611 2.05532 0.58055
C 5.13122 1.39676 -0.63183
C 4.22921 0.33633 -0.77094
C 2.92291 0.12527 2.86381
C 1.79541 1.09611 3.24087
C 3.86577 -0.09254 4.05535
C 4.13605 -0.38911 -2.10788
C 5.50381 -0.93146 -2.55408
C 3.54315 0.49652 -3.21161
C -1.78319 -2.32219 -0.13206
C -2.39469 -2.50312 -1.39320
C -3.78787 -2.62804 -1.44030
C -4.55628 -2.59359 -0.28321
C -3.94097 -2.39728 0.94944
C -2.55447 -2.24357 1.05429
C -1.59293 -2.56640 -2.68612
C -1.72742 -1.25917 -3.48122
C -1.97626 -3.76628 -3.56415
C -1.90978 -2.03016 2.42019
H -0.99495 -1.44112 2.26007
C -1.47670 -3.35368 3.07309
H -1.05566 -3.16782 4.06670
H -0.71828 -3.87525 2.48602
C -1.20185 2.45287 -1.68256
C -1.97523 3.79366 -1.74474
C -1.28335 4.97747 -1.05418
C -0.70484 4.65046 0.33240
C 0.02499 3.28699 0.46468
C 1.36744 3.25430 -0.30079
C 1.24532 3.34785 -1.82968
C 0.14245 2.46651 -2.43929
B -0.92100 2.14421 -0.13904
H 2.46033 -0.84211 2.63998
H 2.19556 -4.35876 0.30345
H -0.53678 -2.67816 -2.41994
H -1.83295 0.87658 1.43908
H 3.46527 -1.24563 -1.98154
H -1.83790 1.69925 -2.16648
H 4.73785 2.13349 2.64844
H 4.30314 0.84656 4.40888
H 1.88969 2.32381 -0.03945
H -0.02307 5.45454 0.64106
H 2.01852 4.06337 0.05572
H 0.50240 1.42601 -2.47725
H 0.26045 3.15643 1.53069
H -4.27525 -2.77740 -2.39951
H 1.25927 0.74469 4.12940
H 1.08609 4.38848 -2.12356
H 5.72151 1.70625 -1.48996
H -1.93807 -4.70917 -3.01024
H 4.21230 -3.80322 0.81786
H 2.20833 3.07347 -2.27528
H -5.63325 -2.72606 -0.33917
H 5.99965 2.87875 0.66227
H -1.52909 4.68395 1.05871
H -0.49566 5.36845 -1.70235
H 4.68933 -0.76607 3.79990
H 2.19214 2.09349 3.45880
H -4.55091 -2.36906 1.84695
H -2.17931 4.05409 -2.79225
H 3.31799 -0.53191 4.89542
H 5.39241 -1.54738 -3.45230
H -0.01586 2.75713 -3.48695
H -2.00174 5.80040 -0.95206
H 4.52130 -2.19827 1.51413
H 4.79380 -2.47184 -0.19679
H -1.29071 -3.84436 -4.41379
H -2.96203 3.64062 -1.28365
H -2.76888 -1.08027 -3.77031
H 0.33958 -5.47638 0.28314
H 1.07171 1.20757 2.42479
H 3.48098 -0.05639 -4.15488
H -1.39155 -0.39601 -2.89773
H -1.12870 -1.30011 -4.39711
H -2.98655 -3.66549 -3.97377
H 5.98059 -1.54297 -1.78192
H 6.19604 -0.11934 -2.79859
H -1.26426 -4.71547 0.37333
H -0.45507 -4.88489 -1.17836
H 2.53787 0.84159 -2.95616
H 4.16485 1.38100 -3.38681
C -2.79869 -1.25184 3.39952
H -3.63995 -1.85685 3.75279
H -2.21962 -0.96960 4.28431
H -3.21239 -0.33953 2.95720
H -2.33576 -4.02257 3.19215
C -4.76680 2.14518 0.59471

C -4.16369 2.97803 1.56148
C -2.82717 2.70974 1.72732
C -2.27598 1.69130 0.85588
S -3.67671 1.03351 -0.07242
C -6.18941 2.21518 0.15974
H -4.71785 3.73430 2.10647
H -2.20293 3.19245 2.47192
H -6.84858 2.20082 1.03405
H -6.46591 1.39567 -0.50520
H -6.36823 3.16200 -0.36376
H -0.18430 0.94858 -0.11259

INT-1[Zn-B]
B3PW91
SCF = -2347.06715562
SCF (C6H5Cl) = -2347.07658786
SCF (D3BJ) = -2347.46271669
SCF (BS2) = -4287.87383522
H (0 K) = -2345.898436
H (298 K) = -2345.837844
G (298 K) = -2345.989625
Low freq. = 19.7939
Second freq. = 25.8053

132
INT-1[Zn-B]
Zn -0.04943 -0.09649 0.26177
N 0.99124 -0.94741 -1.31795
N -0.71805 1.55872 -0.76370
C 0.16670 2.04352 -1.58733
C 1.47862 1.40331 -1.81325
C 1.58184 -0.05230 -2.06216
H 1.97795 1.92639 -2.63198
C -0.04566 3.32875 -2.33563
H 0.82320 3.97528 -2.18541
H -0.94794 3.85419 -2.02454
H -0.10357 3.11936 -3.40921
C -1.98849 2.21831 -0.56660
C -2.17058 3.05088 0.56192
C -3.04613 2.00434 -1.48032
C -4.28695 2.59182 -1.21172
C -4.49392 3.37520 -0.08512
C -3.43471 3.60731 0.78088
C -2.90369 1.20995 -2.77047
H -5.10485 2.43784 -1.91044
H -5.46709 3.81944 0.10471
H -3.58310 4.24863 1.64471
C -1.03821 3.45349 1.49403
C -3.90940 0.05465 -2.84408
C -3.06508 2.11632 -4.00279
H -4.08360 2.51460 -4.06537
H -2.87546 1.55124 -4.92214
H -2.38221 2.96958 -3.98116
C -1.38415 3.26474 2.97547
H -0.50725 3.47905 3.59451
H -1.70625 2.24353 3.18798
H -2.18171 3.94372 3.29653
C -0.64637 4.91926 1.23812
H -0.16815 2.82727 1.27214
H -1.47516 5.58993 1.49212
H -0.38468 5.09984 0.19143
H 0.21471 5.19898 1.85367
C 2.48286 -0.41202 -3.21302
H 2.55601 -1.48525 -3.38205
H 3.47930 -0.00590 -3.01218
H 2.12793 0.07486 -4.12662
H -2.03501 -3.87883 -4.12335
H -1.56540 -1.13702 -4.77711
H -3.10371 -2.64963 -3.46050
C -0.66810 -1.58492 -4.33661
C -2.20171 -3.23102 -3.25553
H 0.06823 -0.79036 -4.20534
H -2.39904 -3.86818 -2.39052
C -1.01310 -2.29087 -3.01508
H -1.33069 -1.52974 -2.29216
H 5.03713 -3.34226 -1.36992
H -0.26278 -2.30139 -5.05981
C 4.67692 -2.34914 -1.07815
H 4.54958 -1.76507 -1.99198
C 1.15778 -2.35833 -1.61321
C 3.37920 -2.47027 -0.25940
C 0.19318 -3.00909 -2.42308
C 2.26419 -3.07822 -1.09916
C 2.38153 -4.43580 -1.42225
C 0.37240 -4.36489 -2.71473
C 1.45484 -5.08106 -2.22343
H 3.23029 -4.99189 -1.03587
H -0.35411 -4.87240 -3.33948
H 3.06862 -1.46913 0.05565
H 1.57131 -6.13424 -2.46330
H 5.46036 -1.87150 -0.48089
H 4.16837 -4.24088 0.75877
C 3.68109 -3.29083 1.00395
H 4.36583 -2.73464 1.65149
H 2.77907 -3.51228 1.57591
H -4.94042 0.42303 -2.81931
H -3.77994 -0.64444 -2.01362
H -3.78821 -0.49410 -3.78400
H -1.89885 0.77673 -2.79977
H -6.22820 -1.54402 1.17857

S -3.29791 -0.51382 1.44956
C -1.77677 -1.39684 1.32520
C -2.00819 -2.52594 0.55353
C -5.64725 -1.52012 0.24978
C -4.18235 -1.67292 0.51294
C -3.34735 -2.68486 0.10588
H -5.87301 -0.57356 -0.25284
H -1.22742 -3.25441 0.35643
H -3.69790 -3.52982 -0.47769
H -6.00101 -2.33500 -0.38800
H 4.52766 2.42366 2.82473
H 2.28213 2.82200 2.17287
C 4.38226 2.59458 1.74970
H 3.98134 0.48540 1.57914
C 3.03490 3.30703 1.53596
H 3.12258 4.34196 1.90237
C 4.52194 1.25311 1.00830
H 5.19857 3.26223 1.45650
H 5.58119 0.95168 1.02252
H 1.75575 1.04573 0.46055
C 2.49586 3.28771 0.08669
B 2.42606 1.72649 -0.35621
H 1.50776 3.76529 0.12827
C 3.96887 1.22769 -0.43513
H 4.06510 0.18800 -0.77176
H 5.41969 3.84735 -0.35622
C 3.34172 4.12880 -0.89471
H 3.41995 5.16808 -0.54049
C 4.79060 2.07744 -1.42810
C 4.75102 3.59190 -1.18307
H 5.83994 1.74512 -1.44191
H 2.80910 4.19137 -1.85696
H 4.41398 1.89107 -2.44681
H 5.16387 4.11209 -2.05830
H 0.19183 -0.05179 1.93269
B -0.55011 -1.06832 2.35929
H -1.75512 0.31112 3.71974
H 0.86177 -2.74619 1.73244
C -1.03395 -0.51378 3.81337
C 0.47413 -2.29189 2.65780
H -2.70823 -1.85516 3.98205
H -1.00947 -3.85402 2.72743
H 2.23554 -1.06113 2.74452
H 0.53082 0.93470 4.06111
C -1.78346 -1.65294 4.53963
C -0.27254 -3.41429 3.41213
C 1.68921 -1.72808 3.42725
C 0.18219 0.03269 4.58405
C -1.00650 -2.97090 4.68825
C 1.36300 -0.94403 4.71033
H -2.10338 -1.31338 5.53643
H 0.42436 -4.22755 3.66627
H 2.39184 -2.53391 3.68645
H -0.11371 0.36300 5.59135
H -1.70511 -3.76279 4.99052
H 2.25704 -0.38580 5.01925
H -0.29385 -2.88811 5.51445
H 1.16995 -1.64651 5.52674

TS1[Zn-B]
B3PW91
SCF = -2347.06677160
SCF (C6H5Cl) = -2347.07754662
SCF (D3BJ) = -2347.46039354
SCF (BS2) = -4287.87368489
H (0 K) = -2345.898666
H (298 K) = -2345.838879
G (298 K) = -2345.988397
Low freq. = -29.9888
Second freq. = 21.5426

132
TS1[Zn-B]
Zn 0.13845 -0.00605 0.28597
N 1.01291 -0.94341 -1.34268
N -0.48720 1.68124 -0.73177
C 0.45241 2.11724 -1.51653
C 1.72323 1.37071 -1.70417
C 1.69513 -0.07814 -2.03887
H 2.30680 1.88325 -2.47290
C 0.37425 3.43494 -2.23133
H 1.28263 4.00674 -2.02062
H -0.49832 4.01908 -1.94044
H 0.35077 3.26446 -3.31306
C -1.70688 2.43092 -0.53351
C -1.85551 3.20858 0.63824
C -2.74756 2.36446 -1.48811
C -3.93511 3.05623 -1.22692
C -4.10882 3.79610 -0.06640
C -3.06808 3.87223 0.84785
C -2.64731 1.61062 -2.80571
H -4.73831 3.01614 -1.95753
H -5.04139 4.32268 0.11614
H -3.18863 4.47337 1.74430
C -0.73408 3.44005 1.63858
C -3.75928 0.56271 -2.94025
C -2.69107 2.57042 -4.00678
H -3.66410 3.06887 -4.07459
H -2.53737 2.02112 -4.94225
H -1.92877 3.35127 -3.94223

C -1.15787 3.15494 3.08379
H -0.29862 3.25846 3.75413
H -1.55412 2.14362 3.19383
H -1.92510 3.85690 3.42808
C -0.21340 4.88278 1.52121
H 0.09193 2.76409 1.39580
H -0.99193 5.59938 1.80634
H 0.09994 5.12536 0.50125
H 0.64447 5.03641 2.18390
C 2.57807 -0.46260 -3.19402
H 2.55590 -1.52872 -3.41480
H 3.60393 -0.16051 -2.95831
H 2.28675 0.09975 -4.08662
H -2.23826 -3.45266 -4.30485
H -1.50999 -0.73376 -4.83685
H -3.19715 -2.16011 -3.59578
C -0.66377 -1.28266 -4.41014
C -2.35349 -2.82878 -3.41148
H 0.14240 -0.56695 -4.23830
H -2.61523 -3.47770 -2.57300
C -1.08725 -2.01129 -3.12428
H -1.34157 -1.25918 -2.36772
H 4.81270 -3.69768 -1.38913
H -0.31852 -2.00120 -5.16194
C 4.53216 -2.69483 -1.04768
H 4.49831 -2.04682 -1.92652
C 1.04442 -2.34988 -1.70197
C 3.19382 -2.74153 -0.29033
C 0.04188 -2.86407 -2.56065
C 2.06498 -3.19518 -1.20420
C 2.07104 -4.53686 -1.60444
C 0.10750 -4.21192 -2.92743
C 1.11194 -5.04854 -2.46196
H 2.85552 -5.18868 -1.23207
H -0.64867 -4.61632 -3.59092
H 2.96209 -1.73477 0.07094
H 1.14069 -6.09223 -2.76216
H 5.32931 -2.33011 -0.39171
H 3.75935 -4.63297 0.65612
C 3.35307 -3.65451 0.93456
H 4.05234 -3.20427 1.64591
H 2.40389 -3.81697 1.44724
H -4.74859 1.03214 -2.93183
H -3.72648 -0.16738 -2.12694
H -3.66621 0.03127 -3.89319
H -1.68881 1.08255 -2.83027
H -6.57520 -0.95722 0.85103
S -3.55426 -0.34877 1.36543
C -2.15655 -1.40949 1.25117
C -2.47450 -2.44041 0.39046
C -5.93516 -0.95154 -0.03873
C -4.52177 -1.31437 0.29214
C -3.79614 -2.39468 -0.14074
H -6.00716 0.04621 -0.48571
H -1.77682 -3.24438 0.17480
H -4.21095 -3.14572 -0.80607
H -6.34898 -1.67048 -0.75181
H 4.56206 1.87761 3.13282
H 2.40237 2.50938 2.38425
C 4.49416 2.10890 2.06157
H 3.91324 0.05599 1.76473
C 3.23076 2.95095 1.81265
H 3.38847 3.95567 2.23444
C 4.55466 0.79440 1.26358
H 5.38240 2.71038 1.84490
H 5.57853 0.39366 1.32130
H 1.83247 0.86539 0.57074
C 2.77766 3.04724 0.33812
B 2.60347 1.53022 -0.20771
H 1.83515 3.61062 0.34684
C 4.09054 0.88585 -0.20806
H 4.11113 -0.13994 -0.59561
H 5.76091 3.35885 0.08093
C 3.75072 3.85081 -0.55436
H 3.89855 4.86104 -0.14372
C 5.04316 1.70196 -1.10961
C 5.12165 3.20164 -0.79231
H 6.05730 1.27582 -1.07830
H 3.28087 4.00476 -1.53853
H 4.71507 1.59369 -2.15582
H 5.62845 3.72059 -1.61727
H -0.11859 -0.17854 1.88729
B -0.91377 -1.22821 2.26195
H -1.98358 0.18671 3.69520
H 0.41283 -2.94211 1.54544
C -1.31479 -0.68546 3.74545
C 0.06079 -2.50352 2.49306
H -3.07379 -1.91798 3.87560
H -1.51922 -3.96889 2.50879
H 1.90290 -1.39663 2.62419
H 0.34636 0.64265 4.03865
C -2.13297 -1.80577 4.43059
C -0.75109 -3.61073 3.20563
C 1.31336 -2.05628 3.27887
C -0.06092 -0.25489 4.52599
C -1.44481 -3.17856 4.50834
C 1.05074 -1.31277 4.59933
H -2.41727 -1.49106 5.44619
H -0.10503 -4.47673 3.41638
H 1.96156 -2.91793 3.49525

H -0.32697 0.05332 5.54845
H -2.19346 -3.93505 4.77980
H 1.98309 -0.83186 4.92496
H -0.72269 -3.18397 5.33061
H 0.81529 -2.03755 5.38431

INT-2[Zn-B]
B3PW91
SCF = -2347.06825893
SCF (C6H5Cl) = -2347.08186405
SCF (D3BJ) = -2347.44824051
SCF (BS2) = -4287.87587588
H (0 K) = -2345.902567
H (298 K) = -2345.841312
G (298 K) = -2345.996846
Low freq. = 14.3165
Second freq. = 17.7516

132
INT-2[Zn-B]
Zn 0.26473 -0.02510 0.24258
N 0.90155 -1.16217 -1.37650
N 0.31440 1.81794 -0.73461
C 1.41686 1.91804 -1.41290
C 2.37469 0.77870 -1.51098
C 1.89102 -0.56060 -1.96504
H 3.18688 1.08231 -2.17681
C 1.86533 3.18940 -2.07306
H 2.87876 3.42461 -1.73359
H 1.20643 4.03024 -1.85825
H 1.92223 3.04096 -3.15678
C -0.60034 2.93290 -0.61911
C -0.60461 3.71117 0.56300
C -1.49746 3.23019 -1.67135
C -2.39445 4.29098 -1.50621
C -2.42600 5.04654 -0.34436
C -1.53030 4.75336 0.67310
C -1.54121 2.48070 -2.99382
H -3.08183 4.52791 -2.31330
H -3.13421 5.86321 -0.23695
H -1.53514 5.35571 1.57645
C 0.40564 3.54396 1.68651
C -2.93685 1.89875 -3.25240
C -1.12603 3.38029 -4.16990
H -1.84225 4.19693 -4.31064
H -1.09676 2.80407 -5.10124
H -0.14204 3.83208 -4.01906
C -0.24739 3.50300 3.07376
H 0.50157 3.25792 3.83317
H -1.04087 2.75490 3.12624
H -0.67835 4.47243 3.34621
C 1.43798 4.68386 1.63716
H 0.93694 2.59790 1.53764
H 0.95296 5.64736 1.83057
H 1.93090 4.75397 0.66300
H 2.21148 4.53690 2.39802
C 2.68051 -1.17249 -3.08712
H 2.35155 -2.18039 -3.33755
H 3.73832 -1.19365 -2.80444
H 2.60677 -0.53497 -3.97484
H -3.03658 -2.53305 -4.01244
H -1.21600 -0.33692 -5.30752
H -3.24763 -0.79435 -3.87388
C -0.71671 -1.20487 -4.86298
C -2.74434 -1.65505 -3.42770
H 0.36226 -1.02626 -4.89206
H -3.12542 -1.78054 -2.41090
C -1.22305 -1.45042 -3.43299
H -1.00654 -0.55693 -2.83794
H 3.45719 -5.08695 -0.98384
H -0.92336 -2.07018 -5.50256
C 3.49925 -4.03905 -0.66614
H 3.81857 -3.44726 -1.52876
C 0.48689 -2.48703 -1.79418
C 2.13591 -3.58559 -0.11844
C -0.50767 -2.62689 -2.78824
C 1.07281 -3.63226 -1.20386
C 0.68040 -4.89351 -1.66465
C -0.84701 -3.91379 -3.21841
C -0.25954 -5.04464 -2.67219
H 1.13463 -5.77533 -1.22252
H -1.59196 -4.02774 -3.99984
H 2.22979 -2.55147 0.22661
H -0.53840 -6.03443 -3.02257
H 4.27130 -3.95420 0.10558
H 1.78093 -5.51544 0.85304
C 1.75844 -4.44730 1.09492
H 2.47255 -4.28268 1.90804
H 0.76028 -4.20722 1.46629
H -3.68784 2.69176 -3.32974
H -3.24444 1.22273 -2.45038
H -2.95044 1.34980 -4.19925
H -0.83514 1.64573 -2.94319
H -6.99737 0.81875 -0.06850
S -4.02445 0.46297 0.84799
C -3.11595 -1.00414 1.16489
C -3.71347 -2.03712 0.47733
C -6.31768 0.39328 -0.81597
C -5.17786 -0.33911 -0.18084
C -4.86972 -1.67280 -0.27027

H -5.97548 1.21860 -1.45083
H -3.33224 -3.05294 0.52057
H -5.46170 -2.37295 -0.85301
H -6.89784 -0.29119 -1.44173
H 4.58702 0.09701 3.61547
H 2.88658 1.47320 2.70012
C 4.73369 0.37282 2.56290
H 3.51844 -1.33234 2.05641
C 3.87912 1.61341 2.24916
H 4.32156 2.48386 2.75746
C 4.43244 -0.85133 1.68027
H 5.79436 0.62999 2.48252
H 5.23847 -1.58921 1.81352
H 2.01361 0.19763 0.74211
C 3.66918 1.91252 0.74771
B 3.06062 0.57735 0.06359
H 2.98249 2.76692 0.69937
C 4.21435 -0.55167 0.18028
H 3.92884 -1.50241 -0.28467
H 6.58630 1.17291 0.82078
C 4.95974 2.35610 0.02066
H 5.39292 3.23617 0.51916
C 5.49325 -0.08947 -0.55434
C 6.04260 1.27708 -0.12233
H 6.28619 -0.84485 -0.44801
H 4.69327 2.69870 -0.99147
H 5.27882 -0.04371 -1.63390
H 6.79265 1.61266 -0.85128
H -0.75413 -0.45939 1.40128
B -1.90330 -1.02670 2.18846
H -2.27402 0.98044 3.17678
H -1.25162 -3.17811 1.87725
C -1.94950 -0.03437 3.45331
C -1.36065 -2.45227 2.69754
H -4.01696 -0.49768 3.84283
H -3.34144 -3.23830 3.02792
H 0.74650 -2.04319 2.59917
H 0.09229 0.62262 3.44377
C -3.06204 -0.60384 4.37423
C -2.45921 -3.01356 3.64012
C 0.01511 -2.30934 3.37683
C -0.57333 0.08198 4.13211
C -2.88854 -2.07698 4.78434
C 0.09809 -1.25261 4.48869
H -3.14870 0.01208 5.28205
H -2.13175 -3.97325 4.06824
H 0.34871 -3.27632 3.78257
H -0.63716 0.70373 5.03788
H -3.83715 -2.44126 5.20119
H 1.15471 -1.06840 4.72603
H -2.17069 -2.14710 5.60707
H -0.33570 -1.65179 5.41016

TS2[Zn-B]
B3PW91
SCF = -2347.06728514
SCF (C6H5Cl) = -2347.07933093
SCF (D3BJ) = -2347.44144282
SCF (BS2) = -4287.87466714
H (0 K) = -2345.902391
H (298 K) = -2345.841572
G (298 K) = -2345.996613
Low freq. = -52.7452
Second freq. = 10.7371

132
TS2[Zn-B]
Zn -0.25161 -0.07068 -0.22602
N -1.02181 -0.96698 1.50139
N -0.57099 1.89480 0.44367
C -1.74882 2.01359 0.97630
C -2.63420 0.83269 1.17474
C -2.11266 -0.37396 1.88149
H -3.53081 1.16947 1.70198
C -2.35315 3.33593 1.35318
H -3.33759 3.42128 0.88324
H -1.73481 4.18195 1.05424
H -2.51619 3.37128 2.43576
C 0.27102 3.04892 0.22922
C 0.36196 3.61228 -1.06550
C 1.01668 3.59866 1.29817
C 1.84884 4.69354 1.04134
C 1.96133 5.24361 -0.22651
C 1.21612 4.70156 -1.26322
C 0.96197 3.08309 2.72773
H 2.41832 5.12435 1.86017
H 2.61563 6.09269 -0.40297
H 1.28462 5.14015 -2.25436
C -0.48268 3.15335 -2.24275
C 2.35577 2.66760 3.21586
C 0.36239 4.12453 3.68682
H 1.00308 5.01032 3.75531
H 0.26850 3.70748 4.69559
H -0.62664 4.46136 3.36523
C 0.35763 2.87775 -3.49548
H -0.26876 2.44499 -4.28181
H 1.17211 2.18087 -3.28749
H 0.79555 3.79731 -3.89870
C -1.56389 4.20015 -2.56040
H -0.98604 2.22150 -1.96513

H -1.10607 5.13823 -2.89420
H -2.18425 4.42864 -1.68866
H -2.22187 3.84349 -3.35952
C -2.98094 -0.87758 3.00038
H -2.61287 -1.80417 3.43968
H -3.99533 -1.03359 2.61844
H -3.05447 -0.11089 3.77935
H 2.69742 -1.57659 4.68470
H 0.60617 0.63253 5.42203
H 2.80637 0.12817 4.27181
C 0.21609 -0.33256 5.08036
C 2.41024 -0.83257 3.93485
H -0.86910 -0.24124 4.97585
H 2.90320 -1.09133 2.99397
C 0.88640 -0.75679 3.76447
H 0.67419 0.01066 3.01263
H -3.21344 -5.14578 1.43929
H 0.40922 -1.06730 5.86996
C -3.30302 -4.15753 0.97427
H -3.77315 -3.49228 1.70423
C -0.56650 -2.17074 2.16358
C -1.92688 -3.64414 0.52076
C 0.32376 -2.07472 3.25688
C -1.00134 -3.43820 1.70842
C -0.57808 -4.57749 2.40116
C 0.70100 -3.24763 3.91887
C 0.25318 -4.49395 3.50752
H -0.91930 -5.55151 2.06256
H 1.36310 -3.17839 4.77658
H -2.06668 -2.67984 0.02322
H 0.55760 -5.39175 4.03813
H -3.97957 -4.25082 0.11849
H -1.27870 -5.63623 -0.11551
C -1.32655 -4.61412 -0.50680
H -1.94655 -4.63863 -1.40875
H -0.31635 -4.31848 -0.79710
H 3.04093 3.52159 3.23014
H 2.79222 1.89870 2.57303
H 2.30221 2.28020 4.23830
H 0.32139 2.19577 2.75132
H 6.95308 1.39153 0.61552
S 4.20661 0.60759 -0.66505
C 3.44391 -0.96563 -0.79543
C 3.96512 -1.78483 0.18421
C 6.17214 1.07544 1.31669
C 5.20055 0.13306 0.67904
C 4.95121 -1.17754 1.00744
H 5.68134 1.98222 1.68750
H 3.64606 -2.81600 0.30105
H 5.46813 -1.68753 1.81507
H 6.66247 0.58879 2.16461
H -4.23283 -0.83471 -3.95573
H -2.73458 0.80515 -3.12359
C -4.50986 -0.40737 -2.98286
H -3.24779 -1.90346 -2.08322
C -3.77733 0.93364 -2.80055
H -4.21830 1.67181 -3.48820
C -4.22707 -1.44607 -1.88300
H -5.58756 -0.22858 -3.04874
H -4.96405 -2.25960 -1.96892
H -1.99619 -0.06215 -0.89646
C -3.75133 1.48491 -1.35712
B -3.12973 0.32833 -0.40718
H -3.13119 2.39023 -1.38750
C -4.19311 -0.89331 -0.44076
H -3.89832 -1.73149 0.20196
H -6.58905 0.50566 -1.58625
C -5.13919 1.93096 -0.84261
H -5.57334 2.68209 -1.51964
C -5.57151 -0.42721 0.07918
C -6.15862 0.80332 -0.62592
H -6.29806 -1.25196 0.02473
H -5.00698 2.45027 0.11945
H -5.47907 -0.19221 1.15146
H -7.00362 1.18834 -0.03898
H 0.89392 -0.61079 -1.17020
B 2.44396 -1.30767 -1.95115
H 2.68259 0.57047 -3.18582
H 1.90097 -3.40617 -1.30553
C 2.46549 -0.49923 -3.32085
C 2.01695 -2.81627 -2.22613
H 4.59681 -0.81859 -3.49414
H 4.08310 -3.45027 -2.28092
H -0.11145 -2.58725 -2.34612
H 0.38395 -0.05271 -3.55882
C 3.69917 -1.09919 -4.06095
C 3.24128 -3.41131 -2.98416
C 0.69697 -2.90939 -3.01561
C 1.14643 -0.62350 -4.10437
C 3.68257 -2.62877 -4.23483
C 0.62408 -2.05545 -4.28974
H 3.80221 -0.62541 -5.04830
H 3.03040 -4.45329 -3.26744
H 0.47944 -3.95651 -3.27411
H 1.23902 -0.14341 -5.09003
H 4.68905 -2.96134 -4.52184
H -0.42025 -2.01132 -4.62671
H 3.04169 -2.89497 -5.07999
H 1.16521 -2.54954 -5.10202

INT-3[Zn-B]
B3PW91
SCF = -1805.33720766
SCF (C6H5Cl) = -1805.34680849
SCF (D3BJ) = -1805.60371980
SCF (BS2) = -3358.03414504
H (0 K) = -1804.470150
H (298 K) = -1804.424285
G (298 K) = -1804.546090
Low freq. = 10.3920
Second freq. = 28.7510

98
INT-3[Zn-B]
Zn -0.00647 -0.08736 -1.27997
N -1.48641 -0.57639 0.08006
N 1.53059 -0.42393 0.07146
C 1.29047 0.05932 1.25113
C -0.03115 0.65372 1.58292
C -1.28570 -0.07763 1.26055
H -0.04083 0.91022 2.64562
C 2.33154 0.14526 2.33003
H 2.34840 1.16243 2.73160
H 3.32623 -0.11863 1.97027
H 2.06069 -0.51903 3.15818
C 2.81263 -0.95265 -0.30438
C 3.62729 -0.19345 -1.17718
C 3.22519 -2.23359 0.12989
C 4.46350 -2.71786 -0.30544
C 5.27628 -1.98180 -1.15545
C 4.85056 -0.73235 -1.58508
C 2.38618 -3.13288 1.02582
H 4.79199 -3.69842 0.02857
H 6.23308 -2.37961 -1.48194
H 5.48309 -0.15209 -2.25124
C 3.25136 1.19498 -1.67150
C 2.03275 -4.44094 0.30385
C 3.08852 -3.44092 2.35750
H 4.00020 -4.02635 2.19704
H 2.43287 -4.02811 3.00999
H 3.37416 -2.53104 2.89233
C 3.10004 1.23166 -3.19893
H 2.79101 2.23114 -3.52369
H 2.34579 0.51731 -3.53944
H 4.04573 0.99745 -3.70055
C 4.26852 2.24557 -1.19989
H 2.28384 1.46474 -1.23710
H 5.25558 2.07403 -1.64356
H 4.38651 2.23317 -0.11195
H 3.94221 3.24864 -1.49256
C -2.31635 -0.11371 2.35284
H -3.27574 -0.50049 2.00888
H -2.45275 0.89618 2.75093
H -1.95374 -0.73396 3.18015
H -2.44966 -5.29293 -0.23076
H -1.71744 -4.45201 2.71733
H -0.83578 -5.27194 0.48127
C -2.48020 -3.87525 2.18260
C -1.57834 -4.65871 -0.03753
H -2.76084 -3.02237 2.80709
H -1.16349 -4.36233 -1.00539
C -1.95768 -3.43317 0.80735
H -1.04795 -2.84414 0.96454
H -5.51707 1.61977 -1.42396
H -3.36490 -4.51340 2.08234
C -4.54284 1.86925 -0.98913
H -4.63994 1.81030 0.09942
C -2.71956 -1.21853 -0.28641
C -3.43787 0.93882 -1.51086
C -2.96185 -2.56555 0.06383
C -3.65748 -0.49714 -1.06146
C -4.84578 -1.13385 -1.43061
C -4.17477 -3.14648 -0.32193
C -5.11731 -2.44333 -1.05830
H -5.57365 -0.58414 -2.02103
H -4.37723 -4.17715 -0.04333
H -2.49165 1.28973 -1.08880
H -6.05187 -2.91539 -1.34829
H -4.31927 2.90804 -1.25212
H -4.24987 0.71138 -3.52934
C -3.32361 1.03262 -3.03971
H -3.12868 2.06742 -3.34175
H -2.50617 0.41073 -3.41481
H 2.93391 -5.00989 0.05232
H 1.48948 -4.25127 -0.62578
H 1.41194 -5.07694 0.94315
H 1.44758 -2.61590 1.25106
H -0.28370 5.48726 -1.31810
H 1.26290 3.69548 -1.18723
C -0.24726 4.97741 -0.34595
H -1.66353 3.56871 -1.14616
C 1.08279 4.21257 -0.23436
H 1.90117 4.94098 -0.12528
C -1.50156 4.09897 -0.19722
H -0.27183 5.78158 0.39589
H -2.37682 4.75318 -0.06217
H -0.09919 1.67961 -0.51789
C 1.14815 3.15125 0.88740
B -0.10717 2.14506 0.67719
H 2.12175 2.65061 0.77401

C -1.44134 3.03530 0.92221
H -2.36922 2.45036 0.83503
H -0.23726 5.43352 2.27649
C 1.13139 3.75696 2.30894
H 1.95702 4.47454 2.43031
C -1.44021 3.63616 2.34557
C -0.18365 4.43594 2.72141
H -2.32580 4.27250 2.49430
H 1.33538 2.95494 3.03541
H -1.54715 2.81579 3.07258
H -0.17596 4.60599 3.80673
H -0.00881 -0.25820 -2.81118

TS3[Zn-B]
B3PW91
SCF = -1805.32755178
SCF (C6H5Cl) = -1805.33842436
SCF (D3BJ) = -1805.58782095
SCF (BS2) = -3358.02467847
H (0 K) = -1804.462369
H (298 K) = -1804.415752
G (298 K) = -1804.541882
Low freq. = -55.2793
Second freq. = 16.4114

98
TS3[Zn-B]
Zn -0.00082 -1.18367 -1.12447
N -1.48427 -0.63789 0.08125
N 1.48331 -0.63989 0.08125
C 1.27975 0.06932 1.18194
C 0.00030 0.51968 1.61224
C -1.27978 0.07106 1.18191
H 0.00062 1.01389 2.57760
C 2.45307 0.40709 2.06821
H 2.17260 1.11340 2.84925
H 3.28033 0.82396 1.48792
H 2.83122 -0.50235 2.54635
C 2.80077 -1.10492 -0.25170
C 3.58943 -0.36621 -1.16292
C 3.26169 -2.33625 0.26687
C 4.52255 -2.79623 -0.12527
C 5.31269 -2.07569 -1.01137
C 4.84046 -0.87454 -1.52521
C 2.41843 -3.20322 1.19051
H 4.88637 -3.74275 0.26615
H 6.28872 -2.45169 -1.30568
H 5.45411 -0.31745 -2.22787
C 3.10712 0.93577 -1.78355
C 1.96144 -4.47585 0.46103
C 3.14630 -3.56330 2.49344
H 4.02033 -4.19621 2.30619
H 2.47895 -4.11811 3.16185
H 3.49437 -2.67331 3.02731
C 2.70504 0.71950 -3.25103
H 2.31274 1.64651 -3.68390
H 1.93654 -0.05400 -3.34548
H 3.56682 0.40577 -3.85120
C 4.14312 2.06179 -1.66611
H 2.21231 1.25565 -1.24081
H 5.04282 1.85332 -2.25531
H 4.45514 2.21894 -0.62857
H 3.72310 3.00222 -2.03759
C -2.45260 0.41035 2.06825
H -3.28026 0.82610 1.48776
H -2.17168 1.11802 2.84788
H -2.83032 -0.49829 2.54828
H -2.82731 -5.08547 0.16904
H -2.48479 -4.11414 3.16252
H -1.32682 -5.08325 1.10772
C -3.15138 -3.55871 2.49386
C -1.96735 -4.47308 0.46178
H -3.49854 -2.66820 3.02744
H -1.40870 -4.23416 -0.44833
C -2.42285 -3.19975 1.19098
H -1.52476 -2.63421 1.46027
H -5.04061 1.85982 -2.25484
H -4.02609 -4.19067 2.30657
C -4.14039 2.06711 -1.66601
H -4.45177 2.22473 -0.62835
C -2.80231 -1.10126 -0.25169
C -3.10597 0.93967 -1.78380
C -3.26488 -2.33187 0.26708
C -3.58995 -0.36166 -1.16308
C -4.84161 -0.86843 -1.52538
C -4.52630 -2.79029 -0.12510
C -5.31543 -2.06888 -1.01140
H -5.45449 -0.31065 -2.22818
H -4.89138 -3.73626 0.26646
H -2.21059 1.25830 -1.24127
H -6.29192 -2.44367 -1.30573
H -3.71924 3.00694 -2.03770
H -3.56692 0.41043 -3.85136
C -2.70456 0.72292 -3.25139
H -2.31108 1.64940 -3.68430
H -1.93717 -0.05165 -3.34607
H 2.82067 -5.08928 0.16834
H 1.40325 -4.23607 -0.44914
H 1.32003 -5.08531 1.10676
H 1.52102 -2.63658 1.45976

H 0.00314 6.07946 -1.22350
H 1.45908 4.19872 -1.22396
C 0.00322 5.49120 -0.29649
H -1.45527 4.20071 -1.22257
C 1.29515 4.65615 -0.23879
H 2.15256 5.32327 -0.06787
C -1.28972 4.65781 -0.23751
H 0.00411 6.22866 0.51081
H -2.14609 5.32601 -0.06563
H 0.00112 2.02841 -0.58198
C 1.30420 3.51247 0.82331
B 0.00189 2.70884 0.41442
H 2.23783 2.95908 0.66205
C -1.29913 3.51398 0.82442
H -2.23353 2.96167 0.66390
H 0.00434 5.75391 2.36414
C 1.29218 4.01426 2.28010
H 2.14875 4.67979 2.46275
C -1.28521 4.01549 2.28129
C 0.00402 4.72222 2.72727
H -2.14102 4.68173 2.46495
H 1.45044 3.14753 2.93721
H -1.44347 3.14879 2.93843
H 0.00456 4.80553 3.82221
H -0.00134 -1.89281 -2.48633

TS4[Zn-B]
B3PW91
SCF = -2008.41672602
SCF (C6H5Cl) = -2008.42508014
SCF (D3BJ) = -2008.72206272
SCF (BS2) = -3949.12964388
H (0 K) = -2007.472257
H (298 K) = -2007.419797
G (298 K) = -2007.558195
Low freq. = -115.8181
Second freq. = 11.7279

108
TS4[Zn-B]
Zn -0.03838 -0.40700 -0.11300
N 1.38489 -1.77060 -0.48423
N -1.59782 -1.42595 -0.82937
C -1.41793 -2.58427 -1.47309
C -0.18219 -3.23054 -1.62466
C 1.09156 -2.89952 -1.13162
H -0.22399 -4.17056 -2.16364
C -2.59740 -3.28797 -2.11013
H -2.77194 -2.87863 -3.11107
H -3.51798 -3.15287 -1.54038
H -2.39825 -4.35571 -2.21928
C -2.93376 -0.96181 -0.58649
C -3.63211 -0.22295 -1.56688
C -3.52868 -1.24009 0.67068
C -4.81769 -0.75948 0.91627
C -5.50866 -0.01726 -0.03558
C -4.91510 0.24520 -1.26230
C -2.81220 -2.07536 1.72576
H -5.29021 -0.96526 1.87158
H -6.50812 0.35108 0.17949
H -5.45866 0.82191 -2.00617
C -3.04744 0.08022 -2.93856
C -3.20453 -1.71894 3.16377
C -3.02268 -3.58167 1.49816
H -4.08977 -3.83020 1.52605
H -2.52270 -4.15929 2.28378
H -2.62132 -3.91418 0.53853
C -2.74322 1.57520 -3.09110
H -2.28487 1.77830 -4.06553
H -2.05711 1.91602 -2.31339
H -3.65809 2.17420 -3.02043
C -3.96331 -0.39167 -4.07840
H -2.09702 -0.45662 -3.02394
H -4.89251 0.18752 -4.11054
H -4.23935 -1.44578 -3.97847
H -3.46493 -0.25982 -5.04499
C 2.15650 -3.94650 -1.38851
H 3.03390 -3.81472 -0.75516
H 2.48317 -3.89902 -2.43273
H 1.74518 -4.94571 -1.22432
H 2.94259 -2.19739 4.26599
H 1.11706 -4.15057 2.96776
H 1.18925 -2.08367 4.37138
C 1.87902 -3.75743 2.28551
C 2.05804 -1.76005 3.78915
H 1.65317 -4.13082 1.28440
H 2.12565 -0.67203 3.86739
C 1.90467 -2.22064 2.33666
H 0.92820 -1.86485 1.98537
H 5.61180 -1.51002 -3.01564
H 2.84831 -4.16854 2.58969
C 4.60497 -1.92731 -3.12462
H 4.63288 -2.95377 -2.74740
C 2.71214 -1.53418 0.00092
C 3.56001 -1.06921 -2.39369
C 2.96157 -1.66216 1.39135
C 3.74022 -1.12934 -0.88194
C 4.98751 -0.79475 -0.34211
C 4.22873 -1.32329 1.87358
C 5.23344 -0.87733 1.02109

H 5.78100 -0.46723 -1.00877
H 4.43572 -1.41026 2.93544
H 2.57052 -1.46895 -2.63379
H 6.20835 -0.60935 1.41909
H 4.38295 -1.96311 -4.19686
H 4.55492 0.85277 -2.69490
C 3.59751 0.36940 -2.91887
H 3.46101 0.38553 -4.00606
H 2.80575 0.96901 -2.46759
H -4.22514 -2.03951 3.40145
H -3.12950 -0.64376 3.34649
H -2.53834 -2.23108 3.86591
H -1.73837 -1.87607 1.62159
H -2.07403 3.49453 4.33429
S -1.52805 1.49384 1.99117
C -0.01153 1.07030 1.20618
C 1.00602 1.32863 2.11786
C -1.55694 2.56812 4.60860
C -0.77197 2.01529 3.46119
C 0.59173 1.86628 3.36326
H -2.31709 1.86181 4.95994
H 2.04594 1.12399 1.88283
H 1.27225 2.14609 4.16182
H -0.89152 2.79052 5.44723
H 0.25924 1.51346 -1.44242
B 0.48003 2.42075 -0.64850
H -1.60041 3.31275 -0.51937
H 2.73810 2.19280 -0.55231
C -0.55129 3.63098 -0.57759
C 1.97072 2.97732 -0.59777
H -0.55517 4.00657 1.53425
H 2.33297 3.38135 1.47901
H 2.01523 2.86908 -2.77001
H -0.74979 3.67153 -2.73444
C -0.27845 4.55487 0.62645
C 2.24027 3.96361 0.55531
C 2.10497 3.64718 -2.00098
C -0.37578 4.34296 -1.95098
C 1.17006 5.04704 0.77662
C 1.06730 4.73925 -2.30701
H -0.94620 5.42897 0.58742
H 3.21636 4.45105 0.41114
H 3.11618 4.06573 -2.11065
H -1.01747 5.23591 -1.98668
H 1.29666 5.46680 1.78368
H 1.11546 4.98249 -3.37703
H 1.34580 5.88650 0.09717
H 1.34029 5.66303 -1.79021

INT-4[Zn-B]
B3PW91
SCF = -2008.44135366
SCF (C6H5Cl) = -2008.45024422
SCF (D3BJ) = -2008.75406792
SCF (BS2) = -3949.15522380
H (0 K) = -2007.495377
H (298 K) = -2007.443220
G (298 K) = -2007.579205
Low freq. = 13.4267
Second freq. = 20.2449

108
INT-4[Zn-B]
Zn -0.03926 -0.27743 0.03228
N -1.46476 -1.65541 -0.28850
N 1.47758 -1.48004 0.54673
C 1.26006 -2.79579 0.64020
C 0.07075 -3.44916 0.28233
C -1.17493 -2.94883 -0.12639
H 0.10522 -4.52834 0.39221
C 2.33350 -3.72383 1.17620
H 1.89807 -4.37491 1.93978
H 3.17752 -3.18828 1.60908
H 2.70872 -4.37226 0.37862
C 2.79763 -0.96828 0.76288
C 3.05868 -0.18424 1.91331
C 3.81237 -1.20800 -0.19566
C 5.06251 -0.61049 -0.00134
C 5.32324 0.18377 1.10686
C 4.32843 0.38100 2.05825
C 3.61919 -2.10078 -1.41766
H 5.84872 -0.77990 -0.73280
H 6.30210 0.63687 1.23870
H 4.54572 0.98761 2.93096
C 2.00466 0.00436 2.99748
C 3.63029 -1.30633 -2.72945
C 4.67466 -3.21728 -1.47836
H 5.67225 -2.81325 -1.68207
H 4.43864 -3.91920 -2.28579
H 4.73538 -3.78072 -0.54294
C 2.24920 1.21926 3.89747
H 1.37810 1.38473 4.53861
H 2.41736 2.13270 3.32029
H 3.11083 1.06771 4.55793
C 1.86725 -1.25474 3.86962
H 1.03969 0.16141 2.49708
H 2.81625 -1.48151 4.36866
H 1.57355 -2.12912 3.28528
H 1.10815 -1.10039 4.64443

C -2.23906 -4.00989 -0.32424
H -3.10210 -3.64468 -0.87981
H -2.59351 -4.37090 0.64670
H -1.80995 -4.86773 -0.84930
H -3.74372 -0.06591 -4.48193
H -1.93202 -2.37565 -4.51289
H -2.01969 0.13889 -4.76217
C -2.55044 -2.34468 -3.60872
C -2.78772 0.12397 -3.98157
H -2.20429 -3.13824 -2.94260
H -2.84209 1.12114 -3.53344
C -2.46353 -0.95993 -2.94611
H -1.42380 -0.82402 -2.62651
H -5.15011 -2.41886 2.89959
H -3.58277 -2.57150 -3.89843
C -4.16645 -2.82944 2.64757
H -4.32567 -3.64718 1.93822
C -2.83953 -1.27359 -0.43127
C -3.23111 -1.74753 2.08328
C -3.33198 -0.88141 -1.69814
C -3.68432 -1.27236 0.70580
C -5.00433 -0.83592 0.55025
C -4.66284 -0.46528 -1.79686
C -5.49439 -0.43029 -0.68364
H -5.66042 -0.81612 1.41645
H -5.05641 -0.16108 -2.76179
H -2.23702 -2.19272 1.98198
H -6.52299 -0.09402 -0.78044
H -3.74636 -3.25144 3.56705
H -4.06197 -0.07581 3.21909
C -3.10534 -0.59349 3.08777
H -2.79425 -0.97443 4.06719
H -2.36901 0.14439 2.76045
H 4.59607 -0.81137 -2.88203
H 2.85142 -0.54051 -2.74264
H 3.46574 -1.97720 -3.58054
H 2.63666 -2.57501 -1.34589
H 4.11220 3.44994 -2.94261
S 2.22176 2.13099 -0.83263
C 0.51487 1.71365 -0.97262
C 0.24264 1.53312 -2.32522
C 3.83111 2.40765 -3.13078
C 2.49454 2.08694 -2.54097
C 1.33961 1.73489 -3.19937
H 4.62037 1.77478 -2.71240
H -0.76176 1.33077 -2.67768
H 1.28153 1.64576 -4.27975
H 3.81105 2.25496 -4.21328
H -0.76019 0.96156 0.99883
B -0.54612 2.03758 0.24999
H 0.96108 2.88829 1.73690
H -2.45183 1.86430 -1.00780
C -0.03612 3.13132 1.33962
C -2.00137 2.54539 -0.27028
H 0.93056 4.47168 -0.04165
H -1.21520 3.76111 -1.87351
H -3.20638 1.55815 1.19752
H -0.91405 2.14151 3.03262
C 0.08708 4.51422 0.65992
C -1.82224 3.91036 -0.97019
C -2.98032 2.59425 0.92115
C -1.00979 3.11789 2.53483
C -1.15524 4.99514 -0.10969
C -2.48830 3.34667 2.17268
H 0.36025 5.27522 1.40698
H -2.79636 4.28470 -1.32019
H -3.94078 3.03051 0.60696
H -0.71246 3.86640 3.28511
H -0.86822 5.83725 -0.75411
H -3.11302 3.05605 3.02873
H -1.88898 5.40795 0.58953
H -2.66288 4.41979 2.04791

TS5[Zn-B]
B3PW91
SCF = -2008.43843853
SCF (C6H5Cl) = -2008.45075601
SCF (D3BJ) = -2008.74676939
SCF (BS2) = -3949.15247335
H (0 K) = -2007.493833
H (298 K) = -2007.442106
G (298 K) = -2007.577830
Low freq. = -41.5502
Second freq. = 15.6048

108
TS5[Zn-B]
Zn 0.19371 -0.43965 -0.17044
N 1.69987 -1.56005 0.44989
N -1.20022 -1.80770 -0.40844
C -0.86857 -3.09913 -0.29063
C 0.37861 -3.57274 0.14847
C 1.54962 -2.88704 0.50967
H 0.45014 -4.65272 0.21500
C -1.87911 -4.17671 -0.62379
H -1.36771 -5.04567 -1.04446
H -2.63415 -3.82889 -1.32953
H -2.39968 -4.50934 0.27988
C -2.56073 -1.45418 -0.68973
C -2.88975 -0.92321 -1.95944

C -3.54286 -1.60167 0.31739
C -4.85047 -1.19893 0.02792
C -5.18947 -0.67462 -1.21229
C -4.21389 -0.54311 -2.19412
C -3.23193 -2.15759 1.70250
H -5.61737 -1.30073 0.79102
H -6.21256 -0.37099 -1.41685
H -4.48835 -0.13456 -3.16100
C -1.84754 -0.80414 -3.06440
C -3.17742 -1.04327 2.75493
C -4.22452 -3.24738 2.13476
H -5.22243 -2.83552 2.31957
H -3.89100 -3.71239 3.06872
H -4.33008 -4.03408 1.38093
C -2.17389 0.26728 -4.10906
H -1.31343 0.41462 -4.76896
H -2.41131 1.22918 -3.64646
H -3.01807 -0.02640 -4.74323
C -1.60873 -2.15287 -3.76242
H -0.90020 -0.51067 -2.59167
H -2.53489 -2.51693 -4.22142
H -1.25123 -2.91710 -3.06826
H -0.86014 -2.04571 -4.55527
C 2.70610 -3.76232 0.94255
H 3.36742 -3.25163 1.64373
H 3.30993 -4.04989 0.07543
H 2.33324 -4.67984 1.40286
H 3.25228 0.85153 4.57298
H 1.37327 -1.41684 4.60728
H 1.50682 1.07437 4.57072
C 2.12447 -1.51529 3.81584
C 2.38119 0.97511 3.91962
H 1.85218 -2.38090 3.20814
H 2.50346 1.91188 3.36746
C 2.19641 -0.22421 2.98440
H 1.22269 -0.10333 2.49037
H 5.79741 -2.21035 -2.27603
H 3.08972 -1.72172 4.29204
C 4.86720 -2.73979 -2.04427
H 5.08463 -3.44537 -1.23610
C 2.99502 -0.98982 0.67366
C 3.73693 -1.76777 -1.67381
C 3.25114 -0.31004 1.88797
C 3.98017 -1.07310 -0.33829
C 5.22008 -0.46921 -0.10535
C 4.51130 0.26673 2.06922
C 5.49053 0.19153 1.08506
H 5.98562 -0.51497 -0.87502
H 4.73052 0.79158 2.99320
H 2.81583 -2.35272 -1.59281
H 6.46084 0.65318 1.24575
H 4.59351 -3.31560 -2.93483
H 4.40245 -0.10863 -2.92741
C 3.52374 -0.75126 -2.80547
H 3.34871 -1.26710 -3.75640
H 2.66576 -0.10305 -2.60741
H -4.15167 -0.55298 2.85612
H -2.44740 -0.27365 2.49333
H -2.90789 -1.45598 3.73411
H -2.23768 -2.61197 1.67568
H -5.03018 3.51458 2.00839
S -2.67509 2.24056 0.40323
C -0.96422 2.14428 0.78499
C -0.82916 2.25444 2.15540
C -4.58708 2.61587 2.45265
C -3.15302 2.45097 2.06054
C -2.04658 2.42804 2.87129
H -5.19751 1.76238 2.13676
H 0.14404 2.26658 2.63535
H -2.10674 2.54105 3.94987
H -4.66985 2.70536 3.53966
H 0.46988 0.97010 -0.89311
B 0.16100 2.23774 -0.35880
H -1.21323 2.59341 -2.13888
H 1.97716 2.39433 1.01298
C -0.25353 2.96395 -1.74771
C 1.58790 2.85342 0.09192
H -1.34610 4.55017 -0.78551
H 0.69447 4.41406 1.28562
H 2.88669 1.54749 -1.00241
H 0.76617 1.63022 -3.08643
C -0.46143 4.46550 -1.42983
C 1.35089 4.35071 0.40834
C 2.65117 2.61945 -0.99835
C 0.81151 2.69951 -2.82844
C 0.71782 5.16506 -0.73261
C 2.25523 3.04657 -2.42388
H -0.70667 5.01224 -2.35312
H 2.29944 4.82609 0.70109
H 3.59179 3.12333 -0.72981
H 0.56523 3.23883 -3.75557
H 0.37133 6.12757 -0.33261
H 2.94725 2.58112 -3.13938
H 1.48473 5.42164 -1.46989
H 2.41200 4.12325 -2.53996

INT-5[Zn-B]
B3PW91
SCF = -2008.44021436
SCF (C6H5Cl) = -2008.45396055
SCF (D3BJ) = -2008.73944761
SCF (BS2) = -3949.15453354
H (0 K) = -2007.497972
H (298 K) = -2007.444665
G (298 K) = -2007.587216
Low freq. = 10.5091
Second freq. = 15.7513

108
INT-5[Zn-B]
Zn -0.40497 -0.52169 0.07590
N -2.12601 -1.24607 -0.55732
N 0.61255 -2.19843 0.22778
C 0.03451 -3.37188 -0.04666
C -1.28946 -3.52151 -0.49435
C -2.28102 -2.55992 -0.75014
H -1.59041 -4.54519 -0.68517
C 0.83230 -4.65107 0.07960
H 0.16634 -5.50226 0.23466
H 1.55235 -4.60355 0.89834
H 1.40060 -4.83589 -0.83826
C 2.00313 -2.15586 0.57117
C 2.37402 -1.94410 1.91945
C 2.97806 -2.27363 -0.44515
C 4.32567 -2.20594 -0.07943
C 4.70739 -2.02366 1.24364
C 3.73598 -1.88885 2.22912
C 2.60636 -2.42127 -1.91477
H 5.08950 -2.29427 -0.84709
H 5.76053 -1.97840 1.50738
H 4.04328 -1.73326 3.25839
C 1.32832 -1.80715 3.01842
C 2.76636 -1.08475 -2.65391
C 3.40286 -3.52429 -2.62542
H 4.46295 -3.26515 -2.71820
H 3.01841 -3.67308 -3.64014
H 3.34338 -4.48094 -2.09607
C 1.77244 -0.90759 4.17627
H 0.92569 -0.71540 4.84278
H 2.14754 0.05578 3.82016
H 2.55578 -1.37734 4.78151
C 0.88714 -3.17734 3.55726
H 0.44340 -1.33878 2.56704
H 1.74206 -3.71969 3.97677
H 0.44098 -3.79972 2.77750
H 0.14316 -3.05525 4.35237
C -3.60502 -3.09645 -1.24852
H -4.08861 -2.40553 -1.94148
H -4.29438 -3.24822 -0.41110
H -3.46519 -4.06041 -1.74163
H -2.69098 1.87548 -4.44687
H -1.55819 -0.84766 -4.70710
H -0.95993 1.56906 -4.34609
C -2.36268 -0.79862 -3.96496
C -1.87404 1.65578 -3.75034
H -2.40825 -1.76676 -3.46038
H -1.76175 2.51082 -3.07759
C -2.11438 0.35418 -2.97909
H -1.18478 0.12419 -2.44123
H -6.37487 -0.97297 2.00642
H -3.30688 -0.64968 -4.50098
C -5.60715 -1.71594 1.76573
H -5.97157 -2.30103 0.91525
C -3.22404 -0.35507 -0.78065
C -4.25063 -1.05965 1.47283
C -3.22390 0.45652 -1.94024
C -4.25305 -0.24904 0.18285
C -5.28471 0.66653 -0.04760
C -4.28440 1.34813 -2.12349
C -5.30947 1.45500 -1.19029
H -6.08029 0.76633 0.68566
H -4.30587 1.97708 -3.00767
H -3.51339 -1.86156 1.36976
H -6.12125 2.15906 -1.35085
H -5.52463 -2.38517 2.62878
H -4.49706 0.64172 2.81697
C -3.80740 -0.19516 2.66199
H -3.78220 -0.78767 3.58349
H -2.81055 0.22468 2.49989
H 3.81322 -0.76450 -2.65779
H 2.18820 -0.28394 -2.18379
H 2.43970 -1.18011 -3.69585
H 1.54849 -2.69503 -1.97193
H 6.16876 2.73721 -1.46758
S 3.43595 1.94355 -0.17870
C 1.80432 2.39883 -0.62896
C 1.80969 2.76474 -1.95852
C 5.53231 2.08330 -2.07489
C 4.08136 2.26760 -1.75960
C 3.07922 2.69415 -2.59504
H 5.86361 1.05340 -1.89870
H 0.90993 3.09966 -2.46564
H 3.25422 2.95580 -3.63464
H 5.71885 2.32006 -3.12638
H 0.02462 0.97938 0.36408
B 0.63410 2.46494 0.43012

H 1.81164 1.94765 2.29693
H -0.98070 3.28810 -0.93636
C 0.97817 2.59661 1.98923
C -0.64034 3.38047 0.10506
H 2.43414 4.14498 1.62202
H 0.61329 5.02315 -0.51855
H -2.22834 2.06444 0.69668
H -0.40996 1.17073 2.78950
C 1.47881 4.06003 2.15557
C -0.12409 4.83733 0.27267
C -1.83900 3.04305 1.01031
C -0.23276 2.25421 2.87458
C 0.52609 5.15082 1.63249
C -1.53266 2.99238 2.51627
H 1.70341 4.25789 3.21446
H -0.94731 5.54646 0.09827
H -2.66036 3.75468 0.83935
H -0.00024 2.43489 3.93479
H 1.08376 6.09321 1.54812
H -2.37242 2.51245 3.03605
H -0.25297 5.34687 2.37506
H -1.49671 4.00840 2.92000

TS6[Zn-B]
B3PW91
SCF = -2008.44017066
SCF (C6H5Cl) = -2008.45338085
SCF (D3BJ) = -2008.73706474
SCF (BS2) = -3949.15441244
H (0 K) = -2007.498240
H (298 K) = -2007.445621
G (298 K) = -2007.586474
Low freq. = -37.1765
Second freq. = 10.5604

108
TS6[Zn-B]
Zn -0.42194 -0.53272 0.06323
N -2.16680 -1.23466 -0.54684
N 0.54274 -2.24473 0.23367
C -0.06127 -3.40702 -0.02994
C -1.39103 -3.52982 -0.46831
C -2.36002 -2.54507 -0.72334
H -1.71822 -4.54732 -0.64862
C 0.71070 -4.70188 0.09842
H 0.02824 -5.54174 0.24290
H 1.42352 -4.67129 0.92444
H 1.28573 -4.89262 -0.81415
C 1.93284 -2.23072 0.57896
C 2.30699 -2.02891 1.92772
C 2.90699 -2.36457 -0.43629
C 4.25526 -2.32661 -0.06918
C 4.63924 -2.15737 1.25527
C 3.66950 -2.00420 2.23959
C 2.53110 -2.49550 -1.90623
H 5.01804 -2.42887 -0.83618
H 5.69278 -2.13645 1.52055
H 3.97832 -1.85840 3.27000
C 1.26422 -1.86945 3.02617
C 2.70357 -1.15290 -2.63167
C 3.31286 -3.60035 -2.62971
H 4.37597 -3.35364 -2.72225
H 2.92456 -3.73421 -3.64506
H 3.24194 -4.56137 -2.10975
C 1.70947 -0.93739 4.15789
H 0.86588 -0.73313 4.82503
H 2.07620 0.01845 3.77378
H 2.50005 -1.38527 4.77016
C 0.83317 -3.22825 3.60063
H 0.37560 -1.41820 2.56586
H 1.69212 -3.75449 4.03232
H 0.39079 -3.87299 2.83685
H 0.08908 -3.09162 4.39331
C -3.70411 -3.05131 -1.19952
H -4.17581 -2.35769 -1.89806
H -4.38836 -3.17013 -0.35251
H -3.59765 -4.02647 -1.67878
H -2.65343 1.89076 -4.44088
H -1.66882 -0.89433 -4.73503
H -0.94033 1.49096 -4.36564
C -2.46174 -0.80528 -3.98410
C -1.84108 1.62003 -3.75714
H -2.55511 -1.77211 -3.48328
H -1.67503 2.46053 -3.07725
C -2.14108 0.32584 -2.99422
H -1.22005 0.04225 -2.46830
H -6.36882 -0.79289 2.06911
H -3.40207 -0.60271 -4.50926
C -5.62694 -1.56246 1.83090
H -6.02066 -2.15123 0.99614
C -3.24077 -0.31560 -0.77058
C -4.25612 -0.95242 1.50683
C -3.23224 0.47802 -1.94247
C -4.25339 -0.16281 0.20408
C -5.26036 0.77943 -0.02772
C -4.26830 1.39773 -2.12711
C -5.27737 1.54998 -1.18292
H -6.04282 0.91452 0.71402
H -4.28276 2.01239 -3.02162
H -3.54510 -1.77786 1.40552

H -6.07034 2.27497 -1.34459
H -5.55160 -2.21883 2.70444
H -4.43085 0.77620 2.82703
C -3.76797 -0.08249 2.67423
H -3.74354 -0.66056 3.60491
H -2.76244 0.30573 2.48809
H 3.75383 -0.84386 -2.63532
H 2.13582 -0.35132 -2.15002
H 2.37214 -1.23242 -3.67343
H 1.47009 -2.75706 -1.96270
H 6.28390 2.55665 -1.45648
S 3.51230 1.90459 -0.16704
C 1.90578 2.42242 -0.63858
C 1.93500 2.76337 -1.97519
C 5.62294 1.91479 -2.05025
C 4.17995 2.16983 -1.74829
C 3.20328 2.62487 -2.60007
H 5.90752 0.87664 -1.84421
H 1.05313 3.12472 -2.49527
H 3.39698 2.85759 -3.64312
H 5.82465 2.11489 -3.10646
H 0.05207 0.95147 0.31456
B 0.73901 2.56116 0.40415
H 1.86769 1.99970 2.28386
H -0.83985 3.38747 -0.99538
C 1.05022 2.66306 1.96529
C -0.52197 3.47377 0.05325
H 2.53860 4.18978 1.62160
H 0.76633 5.10216 -0.54496
H -2.13699 2.17838 0.61355
H -0.37176 1.26103 2.74332
C 1.57398 4.11956 2.14071
C 0.01363 4.92460 0.23384
C -1.74210 3.15148 0.93514
C -0.18187 2.34134 2.82932
C 0.64544 5.22519 1.60544
C -1.46466 3.09705 2.44645
H 1.78570 4.31077 3.20327
H -0.79728 5.64444 0.04719
H -2.54937 3.87504 0.74837
H 0.03596 2.52296 3.89243
H 1.21745 6.15987 1.53301
H -2.32005 2.62728 2.94929
H -0.14294 5.42932 2.33561
H -1.42394 4.11251 2.85132

TS7[Zn-B]
B3PW91
SCF = -2347.02110248
SCF (C6H5Cl) = -2347.02926186
SCF (D3BJ) = -2347.40853511
SCF (BS2) = -4287.82824045
H (0 K) = -2345.855841
H (298 K) = -2345.794127
G (298 K) = -2345.951614
Low freq. = -111.6904
Second freq. = 15.3899

132
TS7[Zn-B]
Zn 0.65814 0.34775 -0.24212
N 0.40595 2.06083 -1.23527
N 2.41974 -0.21984 -1.08688
C 2.85758 0.41624 -2.17414
C 2.21707 1.51736 -2.77019
C 1.20211 2.34951 -2.27540
H 2.70927 1.89050 -3.66174
C 4.17575 0.04123 -2.82312
H 4.52267 -0.95226 -2.54266
H 4.94879 0.76082 -2.53426
H 4.07913 0.09496 -3.91113
C 3.32868 -1.11234 -0.42285
C 3.23128 -2.51056 -0.62372
C 4.31806 -0.57942 0.43731
C 5.20543 -1.45800 1.06795
C 5.13681 -2.82756 0.85865
C 4.15362 -3.34095 0.02053
C 4.46316 0.91054 0.71733
H 5.96827 -1.05574 1.72976
H 5.84285 -3.49449 1.34631
H 4.10063 -4.41368 -0.13301
C 2.16979 -3.12098 -1.52896
C 4.17458 1.22658 2.19215
C 5.85222 1.44131 0.33000
H 6.63584 1.00443 0.95869
H 5.89587 2.52819 0.46018
H 6.10366 1.21483 -0.70998
C 1.78645 -4.54838 -1.12228
H 0.91354 -4.87488 -1.69369
H 1.54218 -4.61724 -0.05817
H 2.59092 -5.26145 -1.33542
C 2.58586 -3.11306 -3.00934
H 1.27320 -2.49430 -1.43849
H 3.53183 -3.64884 -3.14954
H 2.70592 -2.10047 -3.39888
H 1.82477 -3.61295 -3.61836

C 1.11160 3.69589 -2.97278
H 0.12480 4.15072 -2.91300
H 1.39264 3.59160 -4.02318
H 1.81836 4.39619 -2.51570
H 1.02070 5.15161 2.68816
H 3.29970 4.52922 1.01696
H 2.00568 3.73144 3.02194
C 2.39665 4.63235 0.40507
C 1.11485 4.07864 2.48856
H 2.65152 4.32797 -0.61228
H 0.23946 3.58566 2.92191
C 1.25344 3.78686 0.98733
H 1.54330 2.73441 0.88244
H -2.00291 3.47248 -4.19933
H 2.12415 5.69360 0.38590
C -1.81216 2.44932 -3.85509
H -0.74853 2.23729 -3.97031
C -0.48107 3.09985 -0.79034
C -2.30566 2.24937 -2.41138
C -0.05819 3.97816 0.23443
C -1.75111 3.25631 -1.40254
C -2.52275 4.36399 -1.03521
C -0.87697 5.06375 0.56461
C -2.08795 5.27372 -0.07832
H -3.49062 4.51603 -1.50068
H -0.55737 5.75636 1.33765
H -1.96279 1.25875 -2.08651
H -2.70538 6.12937 0.18126
H -2.35165 1.76937 -4.52330
H -4.25122 3.13309 -2.89516
C -3.84003 2.22961 -2.43018
H -4.18982 1.38201 -3.02660
H -4.26655 2.13941 -1.42725
H 4.90432 0.74139 2.85005
H 3.18091 0.87893 2.48647
H 4.23540 2.30585 2.37306
H 3.72370 1.44430 0.11237
H 1.77686 -2.85945 5.17231
S 0.50106 -2.16849 1.72255
C 0.16544 -0.45406 1.50965
C 0.52981 0.16844 2.70454
C 1.45813 -3.18741 4.17914
C 1.04670 -2.00839 3.35508
C 1.00657 -0.68491 3.73022
H 0.63460 -3.89845 4.30778
H 0.46104 1.24304 2.83722
H 1.29746 -0.34371 4.71919
H 2.29134 -3.72866 3.71843
C -4.29486 -1.56311 2.57941
C -2.78218 -1.29464 2.84128
C -2.53951 -0.42034 4.09133
C -2.95174 1.05944 3.97454
H -1.47174 -0.46953 4.32221
H -3.05645 -0.86381 4.95548
C -2.62143 1.72348 2.62708
H -2.46040 1.62455 4.77797
H -4.02274 1.16235 4.17361
C -2.92041 0.86870 1.38067
H -1.55697 1.98125 2.60417
H -3.15863 2.67995 2.55783
C -4.44712 0.60913 1.17806
C -5.11694 -0.31553 2.21207
H -4.58577 0.17756 0.18100
H -4.97227 1.57538 1.16521
H -4.73364 -2.03754 3.46900
H -4.38062 -2.30164 1.77294
H -6.09068 -0.63525 1.81777
H -5.34616 0.24863 3.12006
B -2.32789 -0.58275 1.50789
H -2.31000 -2.26996 3.00872
H -2.57436 1.41996 0.49938
H -2.66071 -5.16415 -0.46166
H -0.83395 -3.49577 -0.50551
H -1.29842 -4.68230 -2.65068
C -2.89016 -4.08938 -0.40728
H -2.91103 -3.84305 0.66347
C -1.75818 -3.26546 -1.04764
C -1.48963 -3.60389 -2.54441
H -0.55882 -3.09950 -2.83723
C -4.28546 -3.85114 -1.00549
H -5.04080 -4.28347 -0.33559
B -2.10470 -1.71667 -1.08008
H -4.37904 -4.41526 -1.93848
C -2.58693 -3.18985 -3.54133
H -3.40272 -3.91795 -3.52313
H -2.17701 -3.23935 -4.55892
C -4.63243 -2.37124 -1.25279
C -3.49804 -1.48559 -1.80826
H -4.95618 -1.93249 -0.30122
C -3.15150 -1.78274 -3.29415
H -2.41361 -1.04109 -3.63204
H -5.51111 -2.31764 -1.91312
H -3.83950 -0.44510 -1.77137
H -4.04302 -1.63415 -3.92128
H -2.24843 -1.30168 0.47962
H -1.20720 -0.90786 -1.26755

INT-6[Zn-B]
B3PW91
SCF = -2347.05508736
SCF (C6H5Cl) = -2347.06488155
SCF (D3BJ) = -2347.44771730
SCF (BS2) = -4287.86307391
H (0 K) = -2345.889387
H (298 K) = -2345.827971
G (298 K) = -2345.982644
Low freq. = 11.4794
Second freq. = 25.9832

132
INT-6[Zn-B]
C -4.15814 0.01727 -0.82866
C -3.46515 -0.22491 0.38780
C -3.83195 -1.31297 1.21037
C -4.88186 -2.14143 0.79660
C -5.56560 -1.91517 -0.38836
C -5.20131 -0.83973 -1.18901
N -2.40473 0.68136 0.74669
C -2.75726 1.83868 1.33527
C -1.99848 3.01300 1.32470
C -0.82854 3.35462 0.60868
N 0.00319 2.48218 0.05032
C 1.22518 2.91292 -0.56730
C 2.31212 3.39809 0.20898
C 3.51992 3.67462 -0.44483
C 3.66866 3.50355 -1.81177
C 2.58357 3.07427 -2.56728
C 1.35453 2.78070 -1.97431
C 2.24456 3.70921 1.70412
C 0.16017 2.41565 -2.84496
C -3.16615 -1.61760 2.54343
C -3.83560 1.20278 -1.73143
Zn -0.48921 0.50157 0.22080
C 1.21961 0.11852 1.83786
C 0.50929 0.28670 3.07346
C 0.34839 -0.88454 3.76210
S 1.07049 -2.19853 2.86684
C 1.63378 -1.18470 1.57527
B 2.59018 -1.70106 0.39666
C -0.28446 -1.09264 5.10127
B 0.50687 -1.89215 -1.30709
H -2.46775 3.85633 1.81959
C -4.07541 1.92612 2.07611
C -0.60964 4.84743 0.48069
H -1.10870 -1.81223 5.06441
H 0.44016 -1.46406 5.83469
H 1.64086 0.94805 1.27662
H 0.15139 1.24367 3.44038
H -0.68288 -0.14543 5.47525
H 1.66411 -1.89961 -0.69488
H 0.03390 -0.71449 -0.87210
H 4.36142 4.03654 0.13955
H 2.69587 2.96831 -3.64083
H 4.61978 3.71670 -2.29135
H -5.17139 -2.97906 1.42540
H -5.74061 -0.66002 -2.11429
H -6.37884 -2.57083 -0.68715
C 3.28730 2.94508 2.53444
C 2.44041 5.21572 1.96314
H 1.25344 3.42448 2.07346
C -3.86721 0.84877 -3.22383
C -4.78510 2.38388 -1.46795
H -2.82064 1.53645 -1.48952
C 0.53604 1.85220 -4.21807
H -0.41476 1.64185 -2.31884
C -0.76276 3.63212 -3.02900
H -2.34500 -0.90502 2.67965
C -4.15123 -1.45264 3.71314
C -2.57927 -3.03603 2.56524
H 3.18100 3.20153 3.59429
H 3.18728 1.86332 2.44037
H 4.30607 3.21125 2.23464
H 2.26639 5.44538 3.02023
H 3.46723 5.51620 1.72886
H 1.77440 5.84027 1.36446
H -2.06962 -3.23101 3.51476
H -3.36438 -3.79234 2.45861
H -1.85872 -3.18694 1.75908
H -3.64187 -1.60389 4.67127
H -4.61510 -0.46287 3.72954
H -4.95921 -2.19006 3.65205
H -4.54789 3.22065 -2.13440
H -5.82499 2.09141 -1.65324
H -4.71618 2.74779 -0.44028
H -3.49335 1.69143 -3.81486
H -3.25131 -0.02508 -3.44883
H -4.88446 0.64397 -3.57476
H -0.35801 1.46572 -4.71607
H 0.95867 2.62478 -4.87045
H 1.25789 1.03479 -4.14375
H -1.61482 3.37480 -3.66726
H -1.15492 3.99803 -2.07775
H -0.21912 4.45327 -3.50956
H -4.87700 1.39118 1.56502
H -3.95753 1.46621 3.06348
H -4.37902 2.96378 2.22387
H -1.50999 5.30590 0.06064

H -0.45442 5.29509 1.46687
H 0.23888 5.09450 -0.15572
H 3.40238 -1.11223 -2.27251
C 4.21314 -1.12712 -1.53321
H 4.97068 -0.42162 -1.90628
H 3.16959 0.35280 -0.39905
H 4.95548 -2.89420 -2.52947
C 3.63053 -0.62558 -0.19747
C 4.82810 -2.53729 -1.49821
H 3.20683 -3.94933 -1.35366
C 4.01447 -3.58209 -0.71096
H 5.84290 -2.48070 -1.09301
C 4.70146 -0.39471 0.89909
H 5.46469 0.31007 0.53692
C 3.37926 -3.08933 0.60478
H 4.65629 -4.45439 -0.51572
H 4.21355 0.10167 1.74803
H 2.73097 -3.89863 0.97600
C 5.39505 -1.66137 1.42808
H 6.16794 -1.98259 0.72408
C 4.43594 -2.82874 1.71159
H 3.91189 -2.62908 2.65567
H 5.93420 -1.41113 2.35190
H 5.02387 -3.74277 1.88259
H 1.22621 -4.53264 -0.73820
C 0.33171 -4.42004 -1.36241
H -0.29147 -5.30236 -1.15282
H -0.54746 -3.22679 0.17843
H 1.48274 -5.25218 -2.99006
C -0.40491 -3.13856 -0.90693
C 0.74515 -4.45125 -2.84544
H 2.39177 -3.07848 -3.10825
C 1.33377 -3.13449 -3.38975
H -0.11529 -4.74667 -3.45329
C -1.79877 -2.97295 -1.54992
H -2.41078 -3.86801 -1.36528
C 0.65415 -1.83447 -2.89967
H 1.32216 -3.16947 -4.48963
H -2.31456 -2.15193 -1.03799
H 1.27916 -0.99885 -3.24130
C -1.79769 -2.67046 -3.05771
H -1.63833 -3.59091 -3.62691
C -0.75901 -1.62242 -3.48535
H -1.10809 -0.62965 -3.16509
H -2.79991 -2.32639 -3.34677
H -0.71437 -1.58583 -4.58427

TS0[Zn-B]
B3PW91
SCF = -2008.43044744
SCF (C6H5Cl) = -2008.44118183
SCF (D3BJ) = -2008.73038536
SCF (BS2) = -3949.14174501
H (0 K) = -2007.486540
H (298 K) = -2007.433520
G (298 K) = -2007.574429
Low freq. = -65.6891
Second freq. = 16.8860

108
TS0[Zn-B]
Zn 0.94573 0.15111 0.19910
N -0.24198 1.53119 -0.58915
N 0.12386 -1.41387 -0.68484
C -0.97323 -1.31856 -1.42056
C -1.71366 -0.11384 -1.59825
C -1.28881 1.22183 -1.33734
H -2.52090 -0.19080 -2.31878
C -1.47280 -2.53805 -2.15356
H -2.44990 -2.36172 -2.60207
H -1.53469 -3.40344 -1.48894
H -0.76952 -2.80264 -2.94991
C 0.83527 -2.65996 -0.58994
C 0.56720 -3.53292 0.48924
C 1.85264 -2.95611 -1.52531
C 2.58463 -4.13610 -1.36164
C 2.33454 -5.00291 -0.30567
C 1.33403 -4.69604 0.60801
C 2.20388 -2.02901 -2.68018
H 3.37115 -4.37416 -2.07303
H 2.91776 -5.91287 -0.19413
H 1.14234 -5.37297 1.43609
C -0.50963 -3.24453 1.52464
C 3.60194 -1.42185 -2.48759
C 2.10731 -2.73107 -4.04270
H 2.85040 -3.53026 -4.13616
H 2.29068 -2.01731 -4.85320
H 1.12186 -3.17942 -4.20462
C 0.10927 -2.84828 2.87384
H -0.67576 -2.60694 3.59908
H 0.76451 -1.97748 2.77823
H 0.70912 -3.66704 3.28725
C -1.47213 -4.42754 1.70185
H -1.09756 -2.39194 1.17143
H -0.97007 -5.29994 2.13420
H -1.91415 -4.73861 0.74972
H -2.28839 -4.15338 2.37802

C -2.07624 2.32728 -1.99475
H -2.32030 3.11949 -1.28263
H -2.99650 1.95148 -2.44073
H -1.47374 2.78777 -2.78471
H 3.66542 3.45034 -2.32034
H 1.66799 2.92014 -4.69579
H 3.49560 1.93453 -3.21562
C 1.32409 3.51219 -3.84073
C 3.10885 2.50764 -2.36604
H 0.25749 3.71618 -3.97867
H 3.32315 1.95205 -1.44834
C 1.60295 2.76634 -2.52767
H 1.10471 1.79298 -2.58887
H -2.19025 4.81942 2.50536
H 1.84877 4.47300 -3.87601
C -2.46926 3.87747 2.02053
H -2.99925 4.12575 1.09529
C 0.14902 2.90144 -0.40471
C -1.24646 2.98778 1.75593
C 1.04984 3.50038 -1.31462
C -0.29932 3.60422 0.73740
C 0.16576 4.90737 0.94053
C 1.47811 4.80804 -1.06556
C 1.04470 5.51217 0.05049
H -0.16787 5.45630 1.81699
H 2.17228 5.27826 -1.75722
H -1.61050 2.04024 1.34746
H 1.39274 6.52610 0.22785
H -3.17338 3.36563 2.68449
H -0.13808 3.58777 3.54002
C -0.51460 2.67201 3.06972
H -1.19381 2.18640 3.77910
H 0.33985 2.00840 2.90634
H 4.37142 -2.20170 -2.48974
H 3.67902 -0.88595 -1.53706
H 3.83426 -0.72265 -3.29821
H 1.48259 -1.20477 -2.68293
H 5.60795 -1.06514 4.14805
S 3.54802 -0.90932 1.80655
C 2.52758 0.40527 1.27449
C 3.08304 1.58948 1.71779
C 5.88504 -0.39417 3.32685
C 4.68400 0.13734 2.60981
C 4.28631 1.44166 2.46493
H 6.54771 -0.95422 2.65712
H 2.63562 2.55708 1.51058
H 4.84364 2.27422 2.88455
H 6.46173 0.43230 3.75191
H -5.72232 -0.75610 3.05061
H -3.82236 -1.96869 2.29594
C -5.53482 -0.69991 1.97026
H -4.18116 0.92644 2.38408
C -4.63369 -1.87898 1.56091
H -5.20820 -2.81458 1.62671
C -4.95172 0.68581 1.63919
H -6.51781 -0.80740 1.50338
H -5.73657 1.44732 1.75705
H -2.26516 -0.26395 0.96500
C -3.98217 -1.76325 0.14855
B -3.24168 -0.36341 0.26207
H -3.30287 -2.62030 0.06091
C -4.30244 0.81763 0.22720
H -3.85346 1.81829 0.19606
H -6.77944 -0.78329 -0.39023
C -4.99391 -1.84061 -1.01088
H -5.56772 -2.77758 -0.95620
C -5.31150 0.71416 -0.93281
C -5.97167 -0.66062 -1.11726
H -6.09667 1.47726 -0.82617
H -4.42880 -1.89945 -1.95196
H -4.78554 0.96570 -1.86483
H -6.46380 -0.69171 -2.09849

INT-0[Zn-B]
B3PW91
SCF = -2008.43672371
SCF (C6H5Cl) = -2008.44644793
SCF (D3BJ) = -2008.74062286
SCF (BS2) = -3949.14889906
H (0 K) = -2007.491309
H (298 K) = -2007.438894
G (298 K) = -2007.576440
Low freq. = 12.6461
Second freq. = 20.4873

108
INT-0[Zn-B]
Zn -0.01265 -0.09001 0.63364
N 1.57699 -0.11092 -0.71943
N -1.40851 -0.67402 -0.78244
C -1.26323 -0.04001 -1.90467
C -0.12953 0.90591 -2.11423
C 1.26493 0.46353 -1.83988
H -0.17662 1.27586 -3.14204
C -2.24530 -0.13479 -3.03787
H -2.57113 0.87355 -3.31325
H -3.11592 -0.74170 -2.78967
H -1.74934 -0.55714 -3.91892
C -2.54032 -1.53267 -0.53726
C -3.69354 -1.00434 0.08765

C	-2.47503	-2.90106	-0.87889
C	-3.60577	-3.69699	-0.66825
C	-4.76188	-3.18277	-0.09926
C	-4.78822	-1.85090	0.28866
C	-1.22743	-3.56062	-1.44460
H	-3.57299	-4.74520	-0.95189
H	-5.62920	-3.81868	0.05463
H	-5.68140	-1.44854	0.75852
C	-3.81006	0.43747	0.55305
C	-0.81999	-4.76924	-0.58806
C	-1.41563	-3.97370	-2.91183
H	-2.21303	-4.71842	-3.01123
H	-0.49690	-4.41674	-3.31193
H	-1.68310	-3.12049	-3.54253
C	-4.04318	0.51909	2.06875
H	-4.05994	1.56567	2.39148
H	-3.25200	0.00771	2.62307
H	-5.00189	0.07032	2.35187
C	-4.92093	1.18272	-0.20214
H	-2.86415	0.94335	0.34027
H	-5.90545	0.75585	0.01963
H	-4.77820	1.13775	-1.28638
H	-4.94175	2.23701	0.09214
C	2.27379	0.81389	-2.89523
H	3.29825	0.63283	-2.57009
H	2.15841	1.86875	-3.16043
H	2.07563	0.23511	-3.80395
H	3.56814	-4.45428	-1.35347
H	2.87582	-3.15227	-4.11104
H	2.01968	-4.58450	-2.19044
C	3.46190	-2.57397	-3.38820
C	2.59451	-3.97153	-1.48802
H	3.61430	-1.57214	-3.79806
H	2.08420	-3.98082	-0.52149
C	2.75350	-2.54231	-2.02455
H	1.74972	-2.13176	-2.17521
H	5.01009	2.64979	1.02854
H	4.44596	-3.04843	-3.30995
C	3.96834	2.71895	0.69548
H	3.97442	2.79744	-0.39605
C	2.92452	-0.51526	-0.41652
C	3.15420	1.50745	1.17797
C	3.48527	-1.66769	-1.01684
C	3.66491	0.22928	0.53193
C	4.95577	-0.19858	0.85567
C	4.78368	-2.04395	-0.65675
C	5.52042	-1.32376	0.27207
H	5.53060	0.37361	1.57831
H	5.22279	-2.92490	-1.11718
H	2.11705	1.66203	0.86341
H	6.52682	-1.63558	0.53709
H	3.54881	3.64619	1.09884
H	4.19148	1.34815	3.09762
C	3.16933	1.42725	2.71055
H	2.72575	2.33293	3.13725
H	2.59716	0.56864	3.06983
H	-1.59424	-5.54300	-0.59115
H	-0.64917	-4.47676	0.45242
H	0.09575	-5.22580	-0.97264
H	-0.41114	-2.83160	-1.40122
H	0.26044	0.60687	6.81925
S	-0.49249	0.46525	3.79473
C	0.08018	-0.57277	2.51496
C	0.61497	-1.70674	3.08897
C	-0.24516	-0.31583	6.51203
C	-0.01503	-0.62735	5.06657
C	0.56158	-1.74072	4.51424
H	-1.31039	-0.19624	6.74145
H	1.05204	-2.51032	2.50279
H	0.93820	-2.56424	5.11434
H	0.13963	-1.12911	7.13416
H	-1.07195	5.27231	1.31433
H	-2.20000	3.24308	0.84927
C	-0.94262	4.89893	0.28968
H	0.69789	3.71880	1.03443
C	-2.07137	3.90023	-0.02237
H	-3.01618	4.45647	-0.12394
C	0.47770	4.32506	0.14459
H	-1.04736	5.78175	-0.34859
H	1.19744	5.15799	0.15976
H	-0.36739	1.66856	0.08991
C	-1.83857	2.99572	-1.25306
B	-0.40829	2.25785	-1.05968
H	-2.68235	2.29308	-1.28071
C	0.70979	3.43121	-1.09481
H	1.73582	3.04637	-1.00380
H	-0.93227	5.67268	-2.25819
C	-1.87458	3.75572	-2.59789
H	-2.83483	4.28062	-2.71410
C	0.65146	4.20638	-2.43092
C	-0.73006	4.75515	-2.81790
H	1.37675	5.03404	-2.42459
H	-1.84524	3.01913	-3.41649
H	0.98212	3.53786	-3.24087
H	-0.71398	5.05989	-3.87324

TS0A[Zn-B]
B3PW91
SCF = -2347.03093824
SCF (C6H5Cl) = -2347.03959562
SCF (D3BJ) = -2347.41893817
SCF (BS2) = -4287.83721756
H (0 K) = -2345.864383
H (298 K) = -2345.803485
G (298 K) = -2345.957758
Low freq. = -89.1497
Second freq. = 15.7291

132
TS0A[Zn-B]
Zn 0.92919 0.06618 0.28677
N 0.24438 1.66545 -0.84902
N 0.54279 -1.40873 -1.09652
C -0.33394 -1.15071 -2.02422
C -1.19526 0.06862 -2.04624
C -0.58103 1.40262 -1.82132
H -1.64253 0.10521 -3.04277
C -0.53615 -2.07590 -3.19560
H -1.56829 -2.43174 -3.21973
H 0.13620 -2.93212 -3.17566
H -0.37668 -1.51330 -4.12180
C 1.26300 -2.66826 -1.10128
C 0.62530 -3.85969 -0.67536
C 2.61358 -2.70212 -1.52114
C 3.27146 -3.93432 -1.57877
C 2.64370 -5.11440 -1.21075
C 1.33809 -5.06194 -0.75133
C 3.38026 -1.46789 -1.96331
H 4.29962 -3.96641 -1.92365
H 3.17127 -6.06222 -1.27017
H 0.84757 -5.97929 -0.43927
C -0.78658 -3.93926 -0.11404
C 4.81898 -1.46265 -1.42885
C 3.36206 -1.33806 -3.49403
H 3.83440 -2.21057 -3.95913
H 3.91150 -0.44834 -3.81946
H 2.34192 -1.26797 -3.88426
C -0.75790 -4.51160 1.31148
H -1.76012 -4.50185 1.75058
H -0.09297 -3.93539 1.95909
H -0.40901 -5.54973 1.31666
C -1.70732 -4.80078 -0.99304
H -1.20420 -2.92919 -0.06064
H -1.37269 -5.84396 -1.00881
H -1.74112 -4.45178 -2.02841
H -2.72918 -4.78763 -0.60043
C -0.97295 2.45079 -2.82808
H -0.48664 3.40867 -2.65159
H -2.05599 2.58935 -2.80626
H -0.72103 2.09783 -3.83351
H 4.80527 3.03565 -1.86385
H 3.20037 1.97567 -4.37273
H 4.75748 1.39259 -2.48643
C 2.72110 2.71804 -3.72555
C 4.24911 2.09393 -1.81838
H 1.68948 2.84759 -4.06495
H 4.31359 1.71229 -0.79556
C 2.79036 2.27854 -2.25335
H 2.29007 1.30854 -2.17497
H -2.04333 5.74809 -0.11829
H 3.23831 3.67222 -3.87405
C -2.26946 4.67652 -0.15634
H -2.31845 4.38680 -1.20891
C 0.82128 2.99396 -0.73244
C -1.21908 3.86705 0.62336
C 2.05966 3.26753 -1.36059
C 0.15847 4.01225 -0.00493
C 0.77376 5.26519 0.10136
C 2.62417 4.53908 -1.21881
C 1.99845 5.53641 -0.48761
H 0.26599 6.05035 0.65319
H 3.57024 4.75185 -1.70775
H -1.51100 2.81311 0.58319
H 2.45268 6.51844 -0.39047
H -3.26332 4.52841 0.27876
H -1.04572 5.39267 2.18195
C -1.22172 4.31560 2.09259
H -2.19343 4.10856 2.54851
H -0.45312 3.80359 2.67807
H 5.43024 -2.24613 -1.88843
H 4.83425 -1.60487 -0.34483
H 5.30455 -0.51011 -1.65198
H 2.87809 -0.59116 -1.54038
H 4.67895 -1.41156 5.02508
S 3.10078 -1.16685 2.34001
C 2.39504 0.22397 1.55377
C 3.02880 1.35425 2.02756
C 5.18910 -0.84837 4.23520
C 4.22153 -0.22035 3.28194
C 4.04997 1.10804 2.99191
H 5.87268 -1.54077 3.73043
H 2.76470 2.35400 1.69553
H 4.64001 1.89048 3.46063
H 5.79419 -0.07434 4.71594
H -6.11150 -0.66193 0.69871
H -4.16450 -1.98545 0.46506

C -5.57089 -0.50965 -0.24571
H -4.41438 0.97913 0.79244
C -4.63551 -1.70906 -0.48440
H -5.24585 -2.57649 -0.78092
C -4.86675 0.85818 -0.19795
H -6.35191 -0.49422 -1.01146
H -5.62897 1.64845 -0.28112
H -2.27370 -0.19222 0.15865
C -3.50269 -1.47915 -1.50727
B -2.67997 -0.13909 -1.07100
H -2.88744 -2.38848 -1.47162
C -3.75087 1.07921 -1.24489
H -3.31979 2.06533 -1.01306
H -5.91794 -0.28945 -2.83593
C -4.04610 -1.37094 -2.95203
H -4.61933 -2.27577 -3.20574
C -4.31825 1.16480 -2.68201
C -4.91290 -0.13722 -3.23716
H -5.07876 1.95844 -2.74010
H -3.20619 -1.35423 -3.66158
H -3.52287 1.47814 -3.37301
H -5.04988 -0.03581 -4.32251
H -0.55441 -0.85727 1.29386
B -1.54557 -0.45775 1.89333
H -2.59632 -2.45059 2.06402
H -0.91461 1.68075 2.22458
C -2.42013 -1.53924 2.64642
C -1.45573 0.87610 2.73064
H -0.60264 -2.43484 3.42710
H 0.45891 0.24402 3.53392
H -3.32121 1.84462 2.29211
H -4.42584 -0.91277 2.21308
C -1.49238 -1.94021 3.83528
C -0.54442 0.44892 3.92658
C -2.82964 1.41076 3.17250
C -3.78922 -0.99311 3.09995
C -1.02718 -0.77429 4.72146
C -3.77060 0.37249 3.80600
H -2.00229 -2.69326 4.45352
H -0.43356 1.30631 4.60633
H -2.69875 2.24012 3.88236
H -4.28312 -1.72517 3.75596
H -0.20666 -1.12355 5.36174
H -4.79073 0.77838 3.81409
H -1.82779 -0.48546 5.40780
H -3.50908 0.24115 4.85988

INT-0A[Zn-B]
B3PW91
SCF = -2347.03384713
SCF (C6H5Cl) = -2347.04315987
SCF (D3BJ) = -2347.42695557
SCF (BS2) = -4287.83981259
H (0 K) = -2345.866005
H (298 K) = -2345.805000
G (298 K) = -2345.958788
Low freq. = 14.3877
Second freq. = 20.8647

132
INT-0A[Zn-B]
Zn 0.85826 0.13623 0.40638
N 0.06082 1.66409 -0.81007
N 0.68644 -1.40211 -1.00922
C -0.24022 -1.25683 -1.90434
C -1.23913 -0.12336 -1.90191
C -0.72340 1.28030 -1.76803
H -1.69557 -0.15754 -2.89596
C -0.37998 -2.21922 -3.05596
H -1.31061 -2.78442 -2.96862
H 0.44991 -2.92303 -3.10534
H -0.43805 -1.65854 -3.99437
C 1.51721 -2.59153 -1.01385
C 1.00165 -3.82205 -0.53600
C 2.84860 -2.51947 -1.48594
C 3.60285 -3.69448 -1.55530
C 3.09146 -4.91579 -1.14369
C 1.80870 -4.96218 -0.62369
C 3.49014 -1.22866 -1.96300
H 4.61569 -3.64761 -1.94074
H 3.69335 -5.81756 -1.21236
H 1.41234 -5.90925 -0.26948
C -0.36418 -4.00366 0.10902
C 4.96039 -1.12559 -1.53493
C 3.35474 -1.08124 -3.48594
H 3.84363 -1.91788 -3.99767
H 3.82702 -0.15633 -3.83344
H 2.30737 -1.06585 -3.80335
C -0.20525 -4.52120 1.54772
H -1.17477 -4.55214 2.05408
H 0.46431 -3.88477 2.13076
H 0.20608 -5.53630 1.55984
C -1.25559 -4.97182 -0.68494
H -0.86432 -3.03181 0.15906
H -0.83584 -5.98380 -0.68315
H -1.37467 -4.67071 -1.72928
H -2.25181 -5.02824 -0.23432

C -1.17327 2.22024 -2.85344
H -0.73853 3.21357 -2.75300
H -2.26239 2.30473 -2.84105
H -0.90071 1.80196 -3.82823
H 4.53499 3.37751 -1.76276
H 3.05551 2.11840 -4.25833
H 4.63385 1.72928 -2.35932
C 2.50285 2.82868 -3.63438
C 4.06276 2.39188 -1.70305
H 1.46755 2.85386 -3.98651
H 4.15451 2.03096 -0.67511
C 2.59729 2.43685 -2.14910
H 2.18241 1.42986 -2.04408
H -2.55321 5.65471 -0.51191
H 2.93089 3.82301 -3.80219
C -2.71562 4.57139 -0.53337
H -2.64667 4.25008 -1.57556
C 0.51153 3.04559 -0.74518
C -1.70534 3.84722 0.37403
C 1.76523 3.38810 -1.30566
C -0.28186 4.04537 -0.12783
C 0.24044 5.34041 -0.02565
C 2.23048 4.70009 -1.17353
C 1.48925 5.67413 -0.52377
H -0.36266 6.10839 0.44890
H 3.18994 4.96366 -1.60798
H -1.93989 2.77919 0.35131
H 1.86993 6.68704 -0.42790
H -3.73769 4.38081 -0.18923
H -1.78069 5.43442 1.87761
C -1.88033 4.34561 1.81672
H -2.87796 4.09041 2.18437
H -1.14507 3.90414 2.49427
H 5.59368 -1.84653 -2.06234
H 5.06789 -1.29197 -0.45965
H 5.35444 -0.13303 -1.76353
H 2.95779 -0.39912 -1.48553
H 5.10363 -0.97220 4.80955
S 3.30087 -0.87763 2.25766
C 2.42425 0.44520 1.52761
C 3.00043 1.62554 1.94971
C 5.50260 -0.37931 3.97841
C 4.41481 0.16183 3.10480
C 4.11319 1.46960 2.82823
H 6.19566 -1.02178 3.42293
H 2.62888 2.59895 1.64321
H 4.67307 2.30078 3.24765
H 6.08198 0.44435 4.40551
H -6.10117 -1.21528 0.77526
H -4.04380 -2.36388 0.65208
C -5.55442 -1.05299 -0.16343
H -4.59074 0.58614 0.83710
C -4.49899 -2.16214 -0.32145
H -5.00770 -3.09375 -0.61250
C -4.98996 0.37853 -0.15975
H -6.31265 -1.15000 -0.94565
H -5.82066 1.08544 -0.30488
H -2.33258 -0.34122 0.36965
C -3.35688 -1.85965 -1.31210
B -2.68155 -0.42026 -0.98295
H -2.65031 -2.69466 -1.22068
C -3.86685 0.67186 -1.18066
H -3.54076 1.70436 -0.98508
H -5.84126 -0.97408 -2.75906
C -3.86914 -1.86915 -2.77731
H -4.34612 -2.83746 -2.98976
C -4.39862 0.63769 -2.63657
C -4.84493 -0.74176 -3.14161
H -5.23360 1.34618 -2.74072
H -3.01951 -1.80830 -3.46987
H -3.62261 1.00420 -3.32223
H -4.95799 -0.70460 -4.23349
H -0.53662 -0.76084 1.18818
B -1.65914 -0.42533 1.63237
H -2.36694 -2.55139 2.03863
H -1.25896 1.78698 1.93763
C -2.26372 -1.59063 2.56089
C -1.64857 0.92939 2.49399
H -0.29400 -2.17496 3.22761
H 0.36907 0.62937 3.22153
H -3.65429 1.64008 2.15769
H -4.37652 -1.27997 2.27850
C -1.22017 -1.80855 3.68653
C -0.63786 0.69457 3.65139
C -3.05013 1.30290 3.00978
C -3.66126 -1.21660 3.10482
C -0.88010 -0.55728 4.50820
C -3.79642 0.17653 3.74483
H -1.56097 -2.60573 4.36426
H -0.62639 1.58022 4.30494
H -2.98290 2.16915 3.68426
H -3.98908 -1.97162 3.83542
H 0.02153 -0.75739 5.10214
H -4.86242 0.43441 3.80485
H -1.67081 -0.36775 5.23997
H -3.45562 0.13992 4.78334

TS0B[Zn-B]
B3PW91
SCF = -2347.04514223
SCF (C6H5Cl) = -2347.05413382
SCF (D3BJ) = -2347.43441115
SCF (BS2) = -4287.85095365
H (0 K) = -2345.878562
H (298 K) = -2345.817590
G (298 K) = -2345.971522
Low freq. = -114.5746
Second freq. = 20.0326

132
TS0B[Zn-B]
Zn -0.03092 -0.01564 -0.10627
N 0.20315 1.94571 0.65040
N -1.55815 -0.55919 1.23187
C -1.30301 -0.13846 2.43279
C -0.11321 0.70932 2.72590
C 0.12543 1.96080 1.94430
H -0.15039 0.97974 3.78469
C -2.11394 -0.53623 3.63307
H -1.44245 -0.95906 4.38655
H -2.89167 -1.26140 3.39528
H -2.57093 0.35248 4.08138
C -2.70330 -1.39750 0.96352
C -2.52121 -2.79000 0.79073
C -3.99429 -0.82968 0.84838
C -5.07364 -1.67117 0.55912
C -4.90712 -3.03563 0.37543
C -3.63681 -3.57952 0.49409
C -4.29231 0.65120 1.02448
H -6.06821 -1.24138 0.47841
H -5.76009 -3.66977 0.15053
H -3.50130 -4.64969 0.36819
C -1.18955 -3.49506 0.98766
C -4.95338 1.22653 -0.23510
C -5.18262 0.91591 2.24942
H -6.17146 0.46232 2.12185
H -5.32995 1.99232 2.39099
H -4.75268 0.50851 3.16821
C -0.81576 -4.38607 -0.20259
H 0.18014 -4.81271 -0.04846
H -0.79785 -3.82121 -1.13719
H -1.51474 -5.22143 -0.32033
C -1.21565 -4.32827 2.28020
H -0.40680 -2.73701 1.08966
H -1.94653 -5.14129 2.20309
H -1.48502 -3.72497 3.15251
H -0.23403 -4.77508 2.46850
C 0.33285 3.19812 2.77262
H 0.58860 4.07131 2.17286
H 1.12792 3.01147 3.50222
H -0.57639 3.40749 3.34705
H -2.76101 4.62517 -2.20372
H -3.76575 4.17848 0.73794
H -3.80552 3.31825 -1.66117
C -2.71848 4.46951 0.60234
C -2.76102 3.61595 -1.77962
H -2.22454 4.42172 1.57777
H -2.29301 2.94420 -2.50490
C -2.02799 3.56639 -0.43092
H -2.07473 2.53490 -0.06686
H 4.23908 4.37214 0.82437
H -2.70426 5.51605 0.27809
C 3.82476 3.42877 1.19775
H 3.19557 3.66453 2.06074
C 0.49665 3.14720 -0.09514
C 3.04395 2.70253 0.09071
C -0.56345 3.93737 -0.59357
C 1.83934 3.51575 -0.35422
C 2.08347 4.70808 -1.04435
C -0.25835 5.12551 -1.26545
C 1.05188 5.52280 -1.48595
H 3.11241 5.00222 -1.23073
H -1.07063 5.75033 -1.62495
H 2.68344 1.75071 0.49102
H 1.26652 6.45157 -2.00712
H 4.65901 2.81209 1.54776
H 4.47050 3.28597 -1.46697
C 3.98557 2.38342 -1.07932
H 4.77765 1.70392 -0.74852
H 3.45792 1.90535 -1.90799
H -5.92494 0.75794 -0.42300
H -4.32925 1.06672 -1.11816
H -5.12968 2.30084 -0.11972
H -3.34537 1.17899 1.17558
H -2.74473 -1.12152 -5.63734
S -1.67818 -0.96075 -2.68345
C -0.21910 -0.18473 -2.07902
C 0.09607 0.83514 -2.97080
C -2.84805 -0.15533 -5.13068
C -1.78536 0.03822 -4.09520
C -0.75762 0.95352 -4.09712
H -3.85282 -0.12068 -4.69590
H 0.93279 1.50557 -2.80430
H -0.61770 1.67973 -4.89215
H -2.78153 0.62952 -5.88917
H 4.40284 -2.56191 1.99430
H 2.07449 -2.81469 1.60907

C 3.75645 -1.99845 2.68014
H 3.74801 -0.42882 1.20696
C 2.34635 -2.61387 2.65465
H 2.38146 -3.59441 3.15435
C 3.81011 -0.50768 2.30129
H 4.20067 -2.14198 3.67005
H 4.79835 -0.10857 2.57823
H 1.19793 -0.49848 1.21219
C 1.23008 -1.73569 3.26271
B 1.26561 -0.30637 2.49994
H 0.29058 -2.28034 3.10276
C 2.68478 0.36941 2.89280
H 2.81377 1.36960 2.46264
H 3.46706 -1.32411 5.24052
C 1.36060 -1.54691 4.79200
H 1.38801 -2.52573 5.29426
C 2.78411 0.55782 4.42357
C 2.56163 -0.71107 5.25737
H 3.75813 0.99543 4.68985
H 0.44889 -1.05564 5.16606
H 2.03584 1.30481 4.73298
H 2.42176 -0.43289 6.31091
H 1.09279 -2.15943 -0.82244
B 1.44983 -1.81476 -1.92788
H 0.20400 -3.25819 -3.14893
H 3.07277 -0.36089 -1.30559
C 1.21252 -2.82600 -3.13165
C 2.88665 -1.14623 -2.05074
H 0.66048 -1.45037 -4.67995
H 2.65351 0.37934 -3.54491
H 3.60900 -2.59813 -0.61462
H 1.79806 -4.49792 -1.89634
C 1.46541 -2.17514 -4.50309
C 3.19666 -0.56803 -3.44498
C 3.81436 -2.33584 -1.66110
C 2.18438 -3.99510 -2.79265
C 2.81660 -1.45636 -4.64388
C 3.64820 -3.59450 -2.53010
H 1.38056 -2.92516 -5.30435
H 4.26371 -0.30837 -3.52003
H 4.86523 -2.01176 -1.69361
H 2.15450 -4.74565 -3.59679
H 2.79785 -0.83584 -5.55007
H 4.16357 -4.43315 -2.04278
H 3.60512 -2.19404 -4.82209
H 4.16976 -3.45143 -3.48086

TS0C[Zn-B]
B3PW91
SCF = -2347.05900256
SCF (C6H5Cl) = -2347.06696509
SCF (D3BJ) = -2347.44378434
SCF (BS2) = -4287.86682834
H (0 K) = -2345.892024
H (298 K) = -2345.830995
G (298 K) = -2345.986081
Low freq. = -40.2150
Second freq. = 15.9764

132
TS0C[Zn-B]
Zn 0.45340 0.15217 0.10231
N -0.59287 1.34881 -1.14263
N 0.16255 -1.61532 -0.82079
C -0.90282 -1.71079 -1.60611
C -1.78029 -0.63870 -1.91534
C -1.52975 0.75795 -1.87992
H -2.57369 -0.91791 -2.60187
C -1.24093 -3.01076 -2.30108
H -2.27659 -3.28368 -2.08157
H -0.58679 -3.82946 -2.00484
H -1.16712 -2.88084 -3.38525
C 1.10219 -2.70297 -0.73766
C 1.04711 -3.61687 0.34264
C 2.10516 -2.82813 -1.72889
C 3.06922 -3.83099 -1.58655
C 3.05336 -4.70404 -0.50757
C 2.03937 -4.59883 0.43521
C 2.15817 -1.95514 -2.97530
H 3.84187 -3.93343 -2.34431
H 3.81299 -5.47518 -0.41264
H 2.00742 -5.30381 1.26049
C -0.09767 -3.64416 1.34599
C 3.48168 -1.18902 -3.09148
C 1.92465 -2.78989 -4.24557
H 2.74224 -3.50127 -4.40625
H 1.87345 -2.14135 -5.12721
H 0.99641 -3.36514 -4.19217
C 0.36114 -3.88396 2.78996
H -0.48038 -3.74714 3.47621
H 1.15478 -3.19689 3.08912
H 0.72686 -4.90685 2.93295
C -1.10941 -4.73953 0.96464
H -0.60805 -2.67552 1.30439
H -0.63410 -5.72655 1.00274
H -1.51095 -4.60603 -0.04234
H -1.95094 -4.74588 1.66580

C	-2.42456	1.56728	-2.79440
H	-2.06462	2.58474	-2.94094
H	-3.42937	1.61359	-2.36456
H	-2.51418	1.07904	-3.76801
H	2.92310	3.39123	-4.11184
H	1.44098	1.01891	-4.83653
H	3.54866	1.79191	-3.72898
C	0.82065	1.70906	-4.25385
C	2.95500	2.62063	-3.33336
H	-0.12783	1.20945	-4.05027
H	3.48331	3.03087	-2.46968
C	1.55080	2.12724	-2.96645
H	1.66632	1.23242	-2.34224
H	-3.57762	5.04519	-0.51771
H	0.61541	2.58473	-4.87992
C	-3.59663	3.95265	-0.43582
H	-3.74715	3.55363	-1.44091
C	-0.32439	2.75594	-1.31305
C	-2.30495	3.43931	0.22617
C	0.74397	3.14230	-2.16550
C	-1.08879	3.74067	-0.64021
C	-0.74729	5.08759	-0.81739
C	1.03350	4.50254	-2.30497
C	0.30239	5.47474	-1.63411
H	-1.32949	5.84726	-0.30352
H	1.84679	4.80945	-2.95372
H	-2.37845	2.35320	0.34701
H	0.54710	6.52643	-1.75523
H	-4.46824	3.68395	0.17069
H	-2.26157	5.16192	1.57133
C	-2.19877	4.06926	1.62278
H	-3.02536	3.72674	2.25383
H	-1.26375	3.81030	2.11942
H	4.33462	-1.87396	-3.15250
H	3.63992	-0.52724	-2.23572
H	3.48951	-0.58093	-4.00260
H	1.35417	-1.21573	-2.90958
H	6.63623	-0.45338	0.57593
S	3.56074	-0.59947	0.98426
C	2.36897	0.69655	1.13137
C	2.89474	1.82040	0.50054
C	6.03795	-0.13313	-0.28409
C	4.70197	0.38983	0.14064
C	4.19179	1.65406	-0.04446
H	5.94287	-0.99331	-0.95518
H	2.35880	2.76440	0.47888
H	4.74989	2.44251	-0.53876
H	6.59536	0.64632	-0.81057
H	-6.06294	-1.19029	2.78726
H	-3.98481	-2.23101	2.27885
C	-5.82020	-1.24436	1.71811
H	-4.70038	0.58207	1.97571
C	-4.75765	-2.33816	1.50561
H	-5.21297	-3.32603	1.66696
C	-5.39116	0.15386	1.23670
H	-6.75243	-1.53451	1.22584
H	-6.26694	0.81868	1.21968
H	-2.53714	-0.51619	0.72710
C	-4.04689	-2.31501	0.11411
B	-3.50985	-0.83191	0.09281
H	-3.25517	-3.07075	0.16742
C	-4.68344	0.19293	-0.15637
H	-4.34933	1.22809	-0.29258
H	-6.90433	-1.77146	-0.67028
C	-4.97082	-2.66261	-1.06911
H	-5.42852	-3.65166	-0.92014
C	-5.59771	-0.17917	-1.33950
C	-6.07548	-1.63875	-1.37146
H	-6.47345	0.48570	-1.37073
H	-4.34621	-2.76092	-1.96903
H	-5.04418	0.01925	-2.26879
H	-6.50053	-1.85382	-2.36074
H	0.06488	0.10890	1.78731
B	1.17893	0.62303	2.28139
H	1.86540	-1.34063	3.20551
H	0.49970	2.79406	2.13861
C	1.53208	-0.34658	3.53722
C	0.75334	2.05551	2.91471
H	3.60808	0.20272	3.69551
H	2.73539	2.89631	2.99137
H	-1.33421	1.57135	3.08135
H	-0.45852	-1.11858	3.77021
C	2.71235	0.26061	4.32864
C	1.94060	2.64439	3.70644
C	-0.51765	1.83920	3.76676
C	0.25831	-0.55073	4.37986
C	2.53382	1.71933	4.78149
C	-0.42243	0.74667	4.84556
H	2.93125	-0.36141	5.20993
H	1.64875	3.59576	4.17670
H	-0.82540	2.77906	4.25016
H	0.47034	-1.17586	5.26068
H	3.50966	2.11550	5.09365
H	-1.43546	0.51144	5.19967
H	1.91156	1.75443	5.68086
H	0.10055	1.14022	5.72217

S8. References

1 M. Stender, R. J. Wright, B. E. Eichler, J. Prust, M. M. Olmstead, H. W. Roesky and P. P. Power, *J. Chem. Soc. Dalton Trans.,* 2001, 3465–3469.

2 M. Garçon, N. W. Mun, A. J. P. White and M. R. Crimmin, *Angew. Chem. Int. Ed.,* 2021, **60**, 6145–6153.

3 C. E. Radzewich, M. P. Coles and R. F. Jordan, *J. Am. Chem. Soc.,* 1998, **120**, 9384–9385.

4 M. E. Grundy, L. Sotorrios, M. K. Bisai, K. Yuan, S. A. Macgregor and M. J. Ingleson, *ACS Catal.,* 2023, **13**, 2286–2294.

5 M. K. Bisai, J. Łosiewicz, L. Sotorrios, G. S. Nichol, A. P. Dominey, M. J. Cowley, S. P. Thomas, S. A. Macgregor and M. J. Ingleson, *Angew. Chem. Int. Ed.,* 2024, **63**, e202404848.

6 D. Uraguchi, N. Kinoshita, T. Kizu and T. Ooi, *J. Am. Chem. Soc.,* 2015, **137**, 13768–13771.

7 H. Braunschweig and C. Hörl, *Chem. Commun.,* 2014, **50**, 10983–10985.

8 A. Prokofjevs, J. W. Kampf and E. Vedejs, *Angew. Chem. Int. Ed.,* 2011, **50**, 2098–2101.

9 J. Yu, G. Kehr, C. G. Daniliuc, C. Bannwarth, S. Grimme and G. Erker, *Org. Biomol. Chem.,* 2015, **13**, 5783–5792.

10 P. I. Jacob and H. C. Brown, *J. Org. Chem.,* 1977, **42**, 579–580.

11 M. Abarbri, J. Thibonnet, L. Bérillon, F. Dehmel, M. Rottländer and P. Knochel, *J. Org. Chem.,* 2000, **65**, 4618–4634.

12 C. Mi, B. B. Zhang, G. Zhang, A. Peng, Z. X. Wang, Q. Shi and H. Huang, *Chem. Eur. J.,* 2024, **30**, e202303857.

13 Y. Maegawa and S. Inagaki, *Dalton Trans.,* 2015, **44**, 13007–13016.

14 C. D. T. Nielsen and J. Burés, *Chem. Sci.,* 2019, **10**, 348–353.

15 M. J. Frisch, G. W. Trucks, H. B. Schlegel, G. E. Scuseria, M. A. Robb, J. R. Cheeseman, G. Scalmani, V. Barone, G. A. Petersson, H. Nakatsuji, X. Li, M. Caricato, A. V. Marenich, J. Bloino, B. G. Janesko, R. Gomperts, B. Mennucci, H. P. Hratchian, J. V. Ortiz, A. F. Izmaylov, J. L. Sonnenberg, D. Williams-Young, F. Ding, F. Lipparini, F. Egidi, J. Goings, B. Peng, A. Petrone, T. Henderson, D. Ranasinghe, V. G. Zakrzewski, J. Gao, N. Rega, G. Zheng, W. Liang, M. Hada, M. Ehara, K. Toyota, R. Fukuda, J. Hasegawa, M. Ishida, T. Nakajima, Y. Honda, O. Kitao, H. Nakai, T. Vreven, K. Throssell, J. J. A. Montgomery, J. E. Peralta, F. Ogliaro, M. J. Bearpark, J. J. Heyd, E. N. Brothers, K. N. Kudin, V. N. Staroverov, T. A. Keith, R. Kobayashi, J. Normand, K. Raghavachari, A. P. Rendell, J. C. Burant, S. S. Iyengar, J. Tomasi, M. Cossi, J. M. Millam, M. Klene, C. Adamo, R. Cammi, J. W. Ochterski, R. L. Martin, K. Morokuma, O. Farkas, J. B. Foresman and D. J. Fox, 2016.

16 A. D. Becke, *J. Chem. Phys.,* 1993, **98**, 5648–5652.

17 D. Andrae, U. Häußermann, M. Dolg, H. Stoll and H. Preuß, *Theor. Chim. Acta,* 1990, **77**, 123–141.

18 W. J. Hehre, R. Ditchfield and J. A. Pople, *J. Chem. Phys.,* 1972, **56**, 2257–2261.

19 P. C. Hariharan and J. A. Pople, *Theor. Chim. Acta,* 1973, **28**, 213–222.

20 A. Höllwarth, M. Böhme, S. Dapprich, A. W. Ehlers, A. Gobbi, V. Jonas, K. F. Köhler, R. Stegmann, A. Veldkamp and G. Frenking, *Chem. Phys. Lett.,* 1993, **208**, 237–240.

21 F. Weigend, *Phys. Chem. Chem. Phys.,* 2006, **8**, 1057–1065.

22 F. Weigend and R. Ahlrichs, *Phys. Chem. Chem. Phys.,* 2005, **7**, 3297–3305.

23 S. Grimme, J. Antony, S. Ehrlich and H. Krieg, *J. Chem. Phys.,* 2010, **132**, 154104.

24 J. Tomasi, B. Mennucci and R. Cammi, *Chem. Rev.,* 2005, **105**, 2999–3094.

25 A. D. Becke, *Phys. Rev. A,* 1988, **38**, 3098–3100.

26 J. P. Perdew, *Phys. Rev. B,* 1986, **33**, 8822–8824.

27 C. Lee, W. Yang and R. G. Parr, *Phys. Rev. B,* 1988, **37**, 785–789.
28 C. Adamo and V. Barone, *J. Chem. Phys.,* 1999, **110**, 6158–6170.
29 S. Grimme, S. Ehrlich and L. Goerigk, *J. Comput. Chem.,* 2011, **32**, 1456–1465.
30 S. Grimme, *J. Comput. Chem.,* 2006, **27**, 1787–1799.
31 Y. Zhao and D. G. Truhlar, *Theor. Chem. Acc.,* 2008, **120**, 215–241.
32 J. D. Chai and M. Head-Gordon, *Phys. Chem. Chem. Phys.,* 2008, **10**, 6615–6620.
33 J. Tao, J. P. Perdew, V. N. Staroverov and G. E. Scuseria, *Phys. Rev. Lett.,* 2003, **91**, 146401.
34 S. C. Liu, X. R. Zhu, D. Y. Liu and D. C. Fang, *Phys. Chem. Chem. Phys.,* 2023, **25**, 913–931.
35 J. Ariai and U. Gellrich, *Phys. Chem. Chem. Phys.,* 2023, **25**, 14005–14015.
36 P. Ríos, A. Rodríguez and J. López-Serrano, *ACS Catal.,* 2016, **6**, 5715–5723.
37 J. Kua, H. E. Krizner and D. O. De Haan, *J. Phys. Chem. A,* 2011, **115**, 1667–1675.
